# Does published research on non-communicable disease (NCD) in Arab countries reflect NCD disease burden?

**DOI:** 10.1371/journal.pone.0178401

**Published:** 2017-06-02

**Authors:** Abla M. Sibai, Neil V. Singh, Samer Jabbour, Shadi Saleh, Sawsan Abdulrahim, Farah Naja, Soha Yazbek

**Affiliations:** 1 Department of Epidemiology and Population Health, Faculty of Health Sciences, American University of Beirut, Beirut, Lebanon; 2 Brighton and Sussex Medical School, University of Sussex, Brighton BN1 9PX, United Kingdom; 3 Department of Health Management and Policy, Faculty of Health Sciences, American University of Beirut, Beirut, Lebanon; 4 Department of Health Promotions and Community Health, Faculty of Health Sciences, American University of Beirut, Beirut, Lebanon; 5 Department of Nutrition and Food Sciences, Faculty of Agriculture and Food Sciences, American University of Beirut, Beirut, Lebanon; 6 Medical Laboratory Sciences Program, Faculty of Health Sciences, American University of Beirut, Beirut, Lebanon; University of Oxford, UNITED KINGDOM

## Abstract

**Objectives:**

To review trends in non-communicable (NCD) research output in the Arab region, in terms of quantity and quality, study design, setting and focus. We also examined differences by time and place, and assessed gaps between research output and NCD burden.

**Methods:**

A scoping review of a total of 3,776 NCD-related reports published between 2000 and 2013 was conducted for seven Arab countries. Countries were selected to represent diverse socio-economic development levels in the region: Regression analyses were used to assess trends in publications over time and by country. Research gaps were assessed by examining the degree of match between proportionate literature coverage of the four main NCDs (CVD, cancer, DM, and COPD) and cause-specific proportional mortality rates (PMR).

**Results:**

The annual number of NCD publications rose nearly 5-fold during the study period, with higher income countries having the higher publication rates (per million populations) and the most rapid increases. The increase in the publication rate was particularly prominent for descriptive observational studies, while interventional studies and systematic reviews remained infrequent (slope coefficients = 13.484 and 0.883, respectively). Gap analysis showed a mismatch between cause-specific PMR burden and NCD research output, with a relative surplus of reports on cancer (pooled estimate +38.3%) and a relative deficit of reports on CVDs (pooled estimate -30.3%).

**Conclusion:**

The widening disparity between higher and lower-income countries and the discordance between research output and disease burden call for the need for ongoing collaboration among Arab academic institutions, funding agencies and researchers to guide country-specific and regional research agendas, support and conduct.

## Introduction

Non-communicable diseases (NCDs) are a major public health issue, responsible for 48% of healthy years of life lost and 63% of all deaths worldwide [1]. Although once seen as diseases of the Global North, almost three quarters of all NCD deaths now occur in low- and middle-income counties [2]. This trend is also true of the Arab World, where ischemic heart disease and diabetes top the list of the causes of death [3, 4]. The financial and social implications of disease and disability associated with NCDs, affecting people at the prime of their productive years, are major causes of impoverishment and barriers to socio-economic development, notably in resource-scarce settings.

The past two decades have seen growing international recognition of the importance of NCDs, with calls drawing attention to the need to intervene at the highest level. In 2000, the World Health Assembly adopted resolution WHA/53.17, endorsing a WHO Global Strategy for the prevention of NCDs and requesting Member States to develop national policy frameworks and promote community-based initiatives based on best available evidence [5].

A decade later, the WHO published two reports that outlined country profiles and capacities to respond to the NCD epidemic, tracking some of the achievements made and outlining challenges faced as countries strive to reach globally agreed targets[6,7]. In 2011, the UN held a high-level meeting on NCDs, setting out road-maps for disease surveillance, prevention and management. Academics also played a leading role, addressing the NCD crisis as “a development emergency in slow motion” [8] and called for translational research with more alignment between funding agencies’ priorities and disease burden [9]. Similarly, the Arab world responded to this growing epidemic. In September 2012, a regional conference was held in Saudi Arabia [10]. This was followed, a year later, by a meeting in Kuwait [11] which focused on the need for more directed relevant country-specific NCD data and locally-driven questions and scale-up intervention research that could feed into NCD prevention and control efforts, and inform policy reforms [3,12].

More recently, there has been a growing interest in the subject of research value, ever since the publication of the seminal paper on ‘Avoidable waste in the production and reporting of research evidence’ by Chalmers & Glasziou [13]. On the regional scene, Arab researchers have advocated for the need to transform the “broken cycle between research production and policy-making if we are to meet regional needs” [14]. Overall, the contributions of Arab nations to biomedical research remain relatively weak, and there are no regional platforms for collaboration to identify research priorities and draw the needed lessons [15–17]. Despite recommendations to reshape the NCD research agenda to guide a Global Strategy Action [18], there appears not to have been any systematic mapping of the NCD research landscape in the Arab world that would identify gaps and recognize opportunities.

Using scoping review methodology and framework [19], we take in this study a close look at NCD publications to survey the profile and focus of NCD research in selected Arab countries. Previous reviews of health research output in the region have relied on the less thorough bibliometric analysis and have either had broader scope (e.g. overall bio-medical research)[16,17] or were focused on one country [20–22]. More recent bibliometric studies examined research production in infectious diseases and nutrition [23, 24]. However, no previous study has examined the NCD research landscape in the Arab region, with a lens of examining ‘research waste’ or addressing gaps and strengths in research output. This paper maps NCD research published from 2000 to 2013 in seven Arab countries and aims to review trends in NCD research output, examine differences by time and place, describe the design, setting and focus of these studies, and assess gaps between research productivity and NCD priorities. Findings from this study have implications on funding allocation and research priority setting in the Arab region.

## Methods

### Inclusion criteria and search strategy

Publications were eligible for inclusion if their content addressed NCD and/or NCD risk factors; if the publication related to human health or health systems; if it was published in the period between January 2000 to December 2013, and if the study population pertained to one of the seven Arab countries, we selected. Countries were decided upon by the research team and were selected to represent various stages of demographic and epidemiological transitions and diverse socio-economic development levels in the region. These are categorised, based on the World Bank income group categorization into low-middle income countries (Sudan, Palestine and Morocco), upper-middle income countries (Iraq and Lebanon), and high-income countries (Bahrain and Kuwait).

A scoping review was used instead of a systematic review because of the broad explanatory nature of the study objectives and research questions. Scoping studies are emerging evidence-mapping tools that aim to summarize and evaluate the quantity, quality and focus of published studies on a broad topic, thus providing a review of a large body of literature across a wide range of study designs [19, 25]. A scoping review does not exclude publications based on the inappropriateness of the research methods or the value of research, nor does it focus on assessing the results of individual studies. Rather, scoping reviews allow mapping of a large scope of research output and of various methods and quality to examine research gaps and opportunities and highlight areas for further in-depth analysis.

In this study, we searched Medline (via PubMed) for all reports of NCD research meeting the above criteria and published between 1^st^ January 2000 and 31^st^ December 2013. We consulted professional librarians in developing our search strategy and the exportation of retrieved records. The search was iterative, with steps being repeated when necessary [19]. The initial search retrieved a total of 9,162 citations, from which 1,212 duplicates were removed, leaving 7,950 unique citations for screening. There was no restriction on publications included (primary research articles, reviews, meta-analyses and commentaries) but these needed to have appeared in peer reviewed academic journals. Of the 7,950 citations, 3,466 were excluded because reading the abstract indicated that they were ineligible (e.g. studies conducted in countries other than those selected or non-human research). A further 708 records were excluded after reading the full text because they did not fully meet our inclusion criteria. Selection of reports involved simultaneous scrutiny by independent reviewers. Disagreements were resolved through joint discussion with AMS and SY. This yielded a final list of 3,776 articles for inclusion in our analysis. The complete search strategy and the list of papers are available as supplementary files on Plos One online. Further details on the search strategy have been published elsewhere [26].

### Data extraction and classification of key characteristics

Article identifiers—including journal, year of publication, authors’ names and affiliations—and abstracts and full-texts (when available) were downloaded. Information on study design, focus (risk factors and outcomes addressed), and study setting were independently extracted using a standardized data abstraction form. *Study design* was classified, when applicable, into case reports or case series, observational studies (cross-sectional, cohort or case-control studies), intervention studies (clinical or population/community-based trials), and reviews (which included literature reviews, systematic reviews and meta-analyses).

*NCD risk factors* included a long list of characteristics and behaviours, which we later grouped into four main domains: social and structural characteristics (e.g. socio-economic variables, health system), behaviours and lifestyles (e.g. tobacco and alcohol use, nutrition/diet, salt intake, physical activity), physiologic factors (e.g. obesity, anthropometric measures, diabetes, hypertension, cholesterol indicators), and other factors such as (trace elements or infectious aetiology). *NCD outcomes* focused on the WHO classification of the four prominent conditions, namely cardiovascular diseases (CVDs), cancers, chronic obstructive pulmonary disease (COPD) and type-2 diabetes (DM). In these categories, papers examining multiple risk factors or more than one disease outcome were counted more than once. The *study setting* was described according to whether the study was laboratory-based (molecular, cellular), hospital/clinic-based and patient-oriented, or community/population-based. Other features were also noted and recorded, including whether the publication had a public health focus/orientation (as opposed to clinical) and whether the authors’ list included collaborations with non-academics such as employees of governmental or non-governmental agencies. Data extraction and coding were independently performed by three teams, each made up of two trained research assistants. Discrepancies were resolved by consensus and discussion with a third party reviewer (AMS and the Project Co-ordinator). All information were recorded in a database and transferred later into a statistical package for analysis.

### Analyses

Characteristics of the reports were described using numbers and percentages. Time trends in quantity were assessed for all the reports and then stratified by country and study type. Regression analyses covering the 14-year study time were also conducted, and slope coefficients for time trends were estimated. Research gaps were assessed by examining the degree of discordance between proportionate literature coverage of the four main NCDs (CVD, cancer, DM, and COPD) and disease burden, as represented by cause-specific proportional mortality rates (PMR) [6]. Bar diagrams presenting differentials between disease coverage and NCD PMRs were plotted and contrasted for each country and for the pooled sample. In an ideal scenario, with a good match between research reporting activity and NCD burden, one would expect the bars to be small and to approach zero. Differentials above the null indicate relative research surplus and differentials below the null indicate relative research deficit. All analyses were conducted using Excel and SPSS 22.0.1.

## Results

Table 1 shows the absolute and relative distribution of the reports of NCD research and provides estimates of the rate of publication per million populations in each of the seven countries. Results show a wide range in the rate of NCD research output, with Kuwait (276.3 publications per million populations), Lebanon (214.3), and Bahrain (151.0) topping the list (referred to hereafter as ‘high-publishing countries’), followed by Palestine (37.5), Morocco (34.2), Iraq (8.0), and Sudan (4.6) (referred to hereafter as ‘low-publishing countries’). The most frequent category of the articles (41.4%) was population-based studies of observational data. Case reports and case series made up 24.6% and laboratory-based studies 15.7% of our study sample. Reports of intervention studies were overall rare, with, randomised trials, meta-analyses and reviews together accounting for only 14.3% of the study data. These patterns were overall comparable across countries except for Morocco, being an outlier with 50.5% of its reports were case reports or case series.

**Table 1:** Descriptive analysis of NCD papers published from 2000 until 2013 in selected Arab countries.

Countries (listed by GDP/capita)		Lower Middle Income Countries						Upper Middle Income Countries				High Income Countries				Pooled		
		Sudan		Palestine		Morocco		Iraq		Lebanon		Bahrain		Kuwait				
Total population		37,195,000		4,550,368		32,521,000		32,778,000		4,647,000		1,318,000		3,250,000				
NCD Proportionate Mortality Rate		53.9%		77.8%		74.5%		62.0%		89.9%		75.0		75.5%				
		Count	%	Count	%	Count	%	Count	%	Count	%	Count	%	Count	%		Count	%
**Total (crude) no. of NCD publications**		169	4.5	157	4.2	1103	29.2	260	6.9	996	26.4	196	5.2	895	23.7	**Total**	**3776**	**100.0**
**NCD publication rate (per million population)**		4.6		37.5		34.2		8.0		214.3		151.0		276.3		**Average**	**142.2**	
**Study Design**	**Observational**	102	60.4	102	68.0	305	28.2	139	56.7	347	36.8	119	61.0	409	45.8		1523	41.4
	**Case report/ Series**	22	13.0	2	1.3	546	50.5	25	10.2	177	18.8	17	8.7	117	13.1		906	24.6
	**Lab studies**	16	9.5	17	11.3	123	11.4	39	15.9	138	14.6	16	8.2	227	25.4		576	15.7
	**Reviews**	18	10.7	13	8.7	72	6.7	12	4.9	170	18.0	37	19.0	96	10.8		418	11.4
	**Interventional**	6	3.6	1	0.7	8	0.7	18	7.3	52	5.5	0	0.0	23	2.6		108	2.9
	**Others**	5	3.0	15	10.0	27	2.5	12	4.9	59	6.3	6	3.1	21	2.3		145	3.9
		169	100	150	100	1081	100	245	100	943	100	195	100	893	100	**Total**	3676	**100.0**
**Risk Factor**	**Physiologic**	33	22.3	57	24.7	145	21.9	54	25.2	167	27.5	82	29.4	267	26.0		805	25.4
	**Social/ Structural**	39	26.4	64	27.7	144	21.8	52	24.3	159	26.2	69	24.7	213	20.7		740	23.3
	**Behaviors**	44	29.7	34	14.7	103	15.6	37	17.3	164	27.0	49	17.6	128	12.5		559	17.6
	**Others**	32	21.6	76	32.9	270	40.8	71	33.2	118	19.4	79	28.3	420	40.9		1066	33.6
		148	100.0	231	100.0	662	100.0	214	100.0	608	100.0	279	100.0	1028	100.0	Total*	**3170**	**100.0**
**Outcome**	**Cancer**	95	53.1	29	14.6	723	59.0	103	38.3	449	42.4	37	14.5	337	30.7		1773	41.4
	**CVD**	34	19.0	60	30.2	176	14.4	65	24.2	282	26.6	81	31.8	261	23.8		959	22.4
	**DM**	14	7.8	22	11.1	73	6.0	23	8.6	68	6.4	27	10.6	82	7.5		309	7.2
	**COPD**	12	6.7	19	9.5	52	4.2	16	5.9	79	7.5	8	3.1	65	5.9		251	5.9
	**Others**	24	13.4	69	34.7	201	16.4	62	23.0	182	17.2	102	40.0	351	32.0		991	23.1
	** **	179	100.0	199	100.0	1225	100.0	269	100.0	1060	100.0	255	100.0	1096	100.0	Total*	**4283**	**100.0**
**Study Setting**	**Hospital/ Clinic based**	87	51.5	56	37.1	777	73.0	157	64.9	447	47.5	92	47.9	436	49.0		2052	56.2
	**Community based**	44	26.0	64	42.4	79	7.4	34	14.0	163	17.3	36	18.8	116	13.0		536	14.7
	**Laboratory based**	13	7.7	15	9.9	96	9.0	31	12.8	120	12.7	12	6.3	200	22.5		487	13.3
	**Others**	25	14.8	16	10.6	112	10.5	20	8.3	212	22.5	52	27.1	138	15.5		575	15.8
		169	100.0	151	100.0	1064	100.0	242	100.0	942	100.0	192	100.0	890	100.0	**Total**	**3650**	**100.0**
**Other features**	**% with public health focus**	77	45.6	104	66.2	212	19.2	66	25.4	287	28.8	91	46.4	288	32.2	**Total**	1125	29.8
	**% collaboration with govt./NGOs co-author)**	31	18.3	18	11.5	53	4.8	17	6.5	59	5.9	27	13.8	86	9.6	**Total**	291	7.7

* The total number do not add to the total (crude) count, because some papers may fit into more than one category, while others may not fit into any category or may be 'not applicable'.

Physiologic factors, including metabolic syndrome and its components, featured in 25.4% of the articles, followed by research addressing social and structural factors (23.3%). Behavioural factors were the focus in a smaller proportion (17.6%). Most of the articles focused on cancer (41.4%) or CVD (22.4%), with many fewer tackling diabetes (7.2%) or COPD (5.9%).

Table 1 also shows that the most common study setting was the hospital/clinic (56.2%) and that less than a third of the reports had a public health focus (29.8%). The exception to this pattern was Palestine, where community-based studies outnumbered hospital-based studies (42.4% vs. 37.1%), and where two-thirds (66.2%) of the papers had a public health focus. Overall, only 7.7% of the total publications involved collaborations with co-authors from governmental or non-governmental agencies.

**Fig 1:**
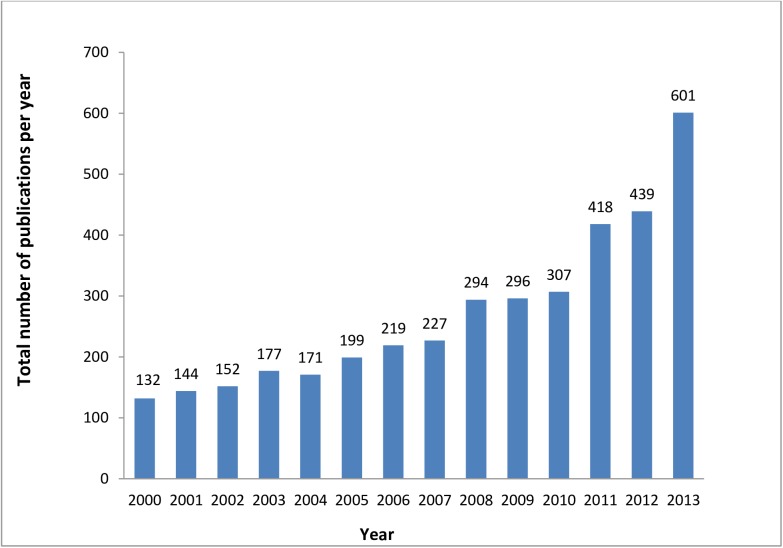
Trend in quantity (number of publications per year) of NCD reports in selected Arab countries between 2000–2013.

**Fig 2:**
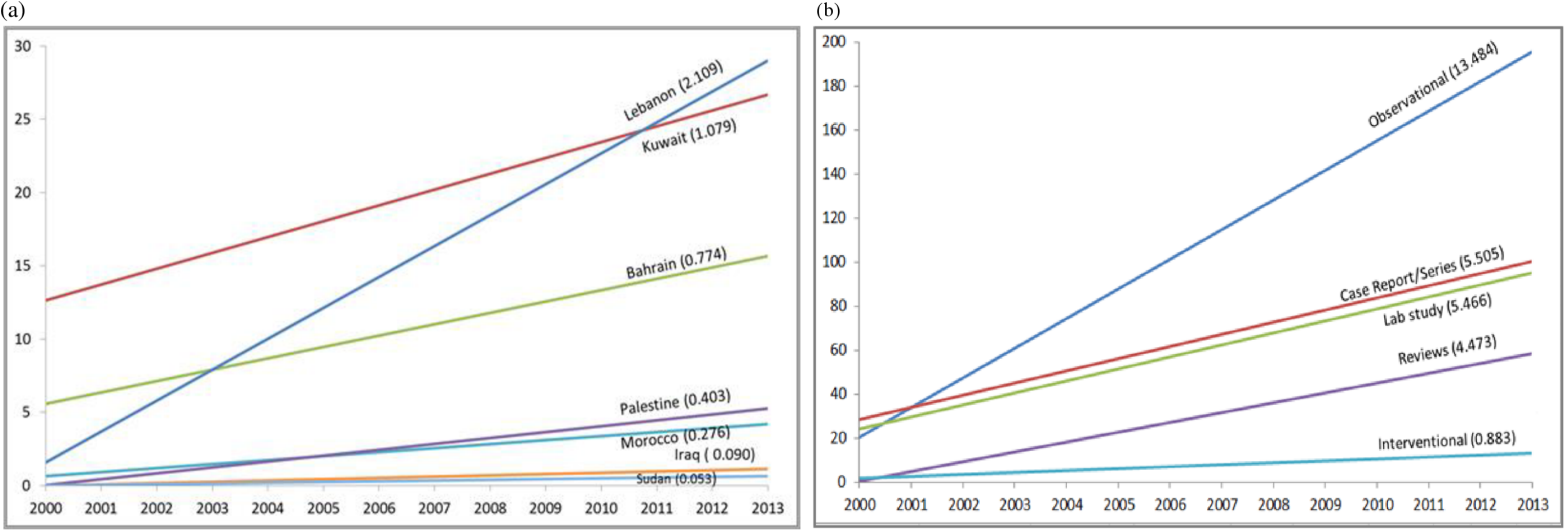
Time trends in NCD publication rate between 2000 and 2013, by (a) country and (b) study type*. *Slopes (regression coefficients) are presented in parentheses

Fig 1 shows that the annual number of NCD research reports increased nearly 5-fold, from 132 in 2000 to 601 in 2013. Yet, the growth in the publication rate varied by country, with Lebanon increasing 8-fold over the period (slope coefficient = 2.109) (Fig 2A). For the remaining countries, the rates were higher in the high-income countries (slope coefficient = 1.079 in Kuwait and 0.774 in Bahrain) compared to those in low-middle income countries (Palestine, Morocco, Iraq and Sudan; range = 0.053 to 0.403). The result was a widening in the gap between reporting rates in ‘high publishing’ countries and the ‘low publishing’ countries over time. Fig 2B shows that time trends in annual publication rates were markedly different for the different study types, with observational studies increasing steeply (slope coefficient = 13.484), intervention studies increasing only gradually (slope coefficient = 0.883), and case reports, case slides, laboratory studies, and reviews increasing at intermediate rates (slope coefficients of 5.505, 5.466, and 4.473 respectively).

**Fig 3:**
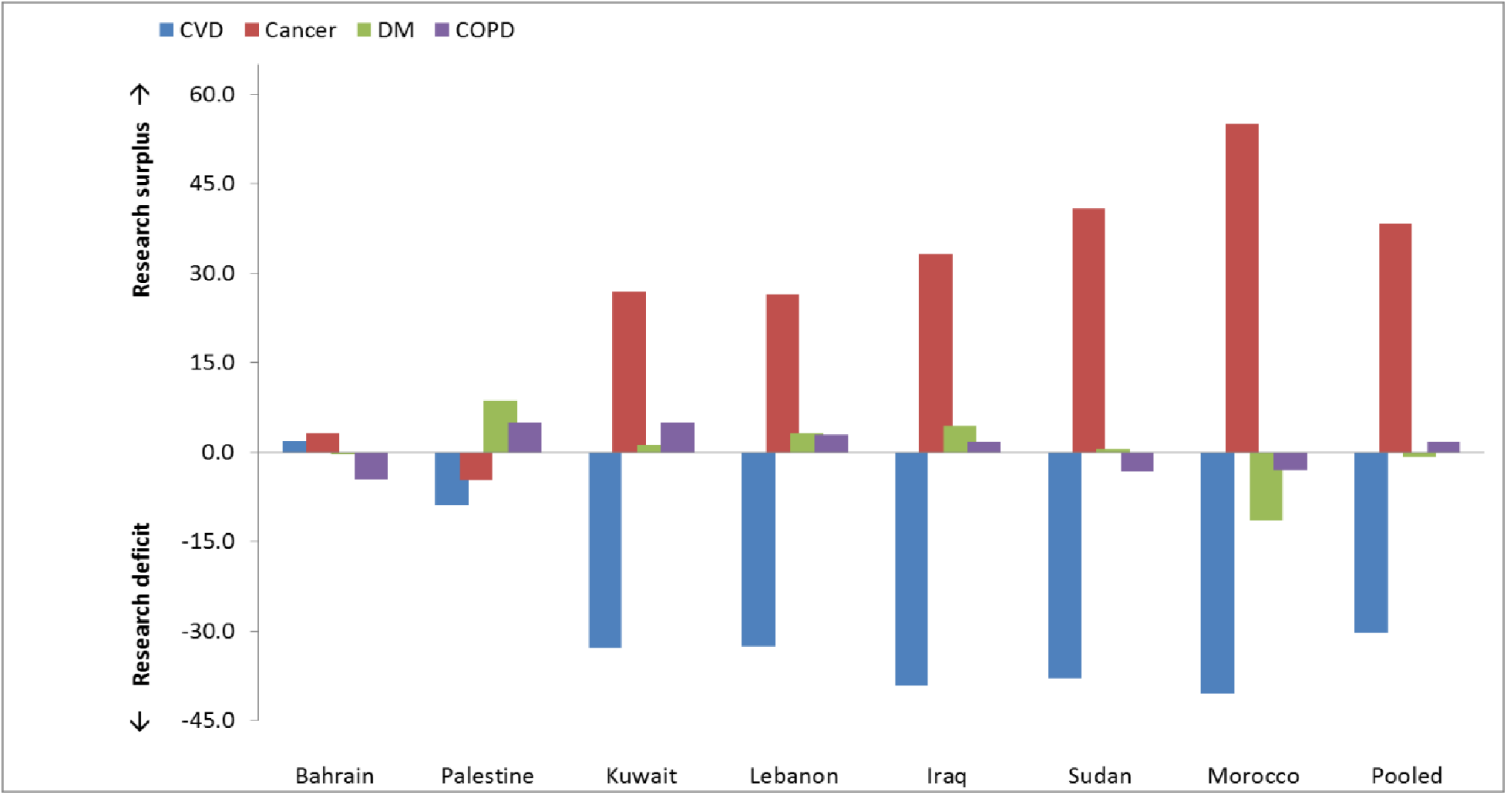
Over- or under-representation of the four major NCDs in the literature compared to proportionate mortality rates (countries presented in order of increasing gaps). (Data on proportionate mortality was sourced from http://apps.who.int/gho/data/node.main.A864?lang=en for all countries except Palestine, for which data was retrieved from the Occupied Palestinian territory STEPS survey 2010–2011. http://www.emro.who.int/pse/programmes/ncds-pal.html, http://www.abudis.net/chronic_diseases_in_palestine.htm)

Fig 3 compares country-specific NCD burden as indicated in cause-specific proportional mortality rates (PMRs) for the four major groups of NCDs (CVD, cancers, DM, and COPD), and research publications addressing these. This gap analysis shows that publications from Bahrain and Palestine matched their cause-specific PMR burden, but there were large disparities in the other countries, with a relative ‘surplus’ of research publications on cancer (pooled estimate 38.3%) and a relative ‘deficit’ of research publications on CVDs (pooled estimate -30.3%).

## Discussion

To our knowledge, this study is the first to document and characterize NCD health research publications in the Arab region. Findings showed an overall increase in NCD publication rate for the past few years, yet the increase was mainly the result of a rise in observational studies, notably cross-sectional, and was characterised by a relative dearth of high-level evidence output such as intervention studies and systematic reviews. Also, there was a discordance between the focus of the research and disease burden, with a relative surplus of publications on cancer and a relative deficit in CVD research.

These findings need to be considered in light of certain limitations. First, we surveyed NCD research production in only seven out of the 22 countries in the EMRO region. However, countries were selected to represent a range of geographic regions, GDPs, socio-political contexts and epidemiologic transitions, thus providing a reasonable representation of the region as a whole. Second, our search strategy was restricted to PubMed, so we may have excluded reports published in some regional journals, particularly those written in Arabic, and others not indexed in PubMed. Yet, the search was systematic and reproducible across countries; hence, our comparative assessments between countries remain valid to conclude. Last, because of our focus on GDP as an indicator of the level of economy and categorization of countries, our interpretation of study findings overlooked additional geopolitical particularities and other factors bound to play a large role in constraining academic output and excellence in some countries. For example, we did not account for the impact of such broader forces as economic sanctions in Iraq, debt repayments in Sudan, or the Israeli-Palestinian conflict on research funding and output.

With these limitations acknowledged, we believe our findings are of importance. The present study has shown that, overall, Arab countries have been increasingly productive in NCD research, with an almost 5-fold rise in the numbers of reports published between 2000 and 2013. The rise in NCD literature partly reflects the proliferation of academic institutions in the region in recent years, with a current estimate of 400 universities compared to 174 a decade ago [27]. Also, the rise is likely to be the result of recent increases in NCD research funding for the region, although this remains meagre compared to other regions [28]. Overall funding for scientific research in Arab countries is among the lowest in the world [29], with spending on research and development at just 0.15% of GDP, compared, for example, to that in Africa (0.3%) or to the world average of 1.4% [30].

Despite the growth in NCD research output over the years, our study revealed several areas of concern. First, high-income countries remained more prolific than lower income countries throughout the study period. More worryingly, the disparity in publication rate between the ‘high-publishing’ and the ‘lower-publishing’ countries at baseline have widened over time. Only Lebanon bucked this trend, starting as a low-publishing country in 2000 but becoming the most highly publishing country by 2013. Compared to other countries in the study, Lebanon is known to host the largest number of universities when weighted to its population size.

Second, the majority of the increase in NCD literature rate was accounted for by lower-evidence research (e.g., descriptive observational studies), with a relative dearth of systematic reviews and controlled intervention studies. This may reflect the lack of self-sustaining scientific infrastructure with high quality training or is the consequence of academic reward systems that incentivise quantity over quality, encouraging researchers to reach for the low-hanging fruit of observational studies rather than investing time and effort in interventional trials. In their recent review of the burden of NCD in the Arab region, Rahim and colleagues [3] acknowledge the lack of implementation research and note that the dearth of studies that evaluate intervention programs and monitor population-based policies ‘is particularly disturbing’. Furthermore, and notwithstanding the limitations of the h-index, as a measure of the quality of publications, El-Idrissi et al. have noted the relatively weak impact of the medical research output in Arab countries, with h-ratings being half that of Iran, almost one fourth that of Turkey, and less than 4.5% of that of the US [15]. Although there are some current initiatives to assess directly the quality of research in at least one of the countries (Palestine) included in our sample, there has been no systematic assessment of the quality of reports of NCD research specifically. This is a deficiency that we intend to address in further studies.

Third, our gap analysis has shown that CVD remained an understudied topic of interest to researchers and that there may be an unwarranted excess in research on cancer, when compared to its disease burden. In an earlier analysis focused on the genetic component in NCD publications in the Arab region using the same data set as ours, Jamaluddine and colleagues note the lack of alignment of the knowledge produced with globally identified priorities and with the burden of heritable diseases of relevance to the region, and call for focused research agendas to include community genetics [26]. In a recent review addressing research priorities, Chalmers et al [31] note that funders have primary responsibility for reducing waste and increasing research value by demanding that proposals be justified by systematic reviews of existing evidence and by so-called ‘research-on-research’, as illustrated by our study. Our findings corroborate some evidence from the global health literature, noting a lack of alignment between donor priorities and patterns of development assistance for health, taking account of recipients’ disease burden, particularly from NCDs [32–34]. The domination of reports of cancer research in the Arab literature may have been additionally facilitated by the availability of cancer registries and hence easy access to secondary data sources.

Fourth, there were relatively high proportion of reports based on hospital/clinic study samples and a relatively low proportion of those that are community-based or have a public health orientation. Thus, there appears to be an over-emphasis on clinical and disease-centred approaches to research as opposed to upstream population based studies. It is worth noting that the two countries where publications matched national proportionate NCD burden with the smallest relative surpluses and deficits (Bahrain and Palestine) were also countries with higher-than-average proportions of population-based research, and higher proportions of papers with public health orientation.

## Conclusion

The landscape of NCD research production in the Arab world is not an even plain; rather, there are towering mountains of research in some higher income countries whilst there are gaping voids, with unmet needs, in others. The widening disparity between higher and lower-income countries and the discordance between research output and disease burden may imply a misdirection of research funds and gaps in knowledge production between and within countries in the region. Rashad and colleagues note that the extent to which national governments contribute to research funding in the Arab region fluctuates between countries, and investments, when available, are often not appropriately aligned with priority health problems [35]. While these observations and our findings are appealing for inducing change, this paper raises more questions than answers and shortcomings cannot be tackled simply by redirecting research funding or injecting cash from existing sources. What is needed are strategic interventions and broader changes. There is a need for ongoing collaboration among Arab academic institutions, funding agencies and researchers across various disciplines to scrutinize NCD research support, conduct and production, in order to identify overlaps, synergies and opportunities. Such data are crucial to guide country-specific and regional funding agendas and flows and to advise on policy frameworks and channels for ‘reducing research waste and increasing research value’.

## Supporting information

S1 File Search terms. (PDF)

S2 File Dataset. (DOCX)
