# Supplementary material for: Does published research on non-communicable disease (NCD) in Arab countries reflect NCD disease burden?
Source: PLoS One. 2017 Jun 2;12(6):e0178401. doi: 10.1371/journal.pone.0178401 (PMC5456081; doi:10.1371/journal.pone.0178401)
Supplement: S1 File — (PDF) [file pone.0178401.s001.pdf]

NCD Scoping project

- (Coronary artery disease OR myocardial infarction OR angina pectoris OR peripheral vascular disease OR peripheral arterial disease OR atherosclerosis OR thrombosis OR hypertension OR primary hypertension OR essential hypertension OR secondary hypertension) AND Country

- (Stroke OR cerebrovascular accident OR ischemic stroke OR hemorrhagic stroke OR vascular dementia OR Transient ischemic attack) AND Country

- (Metabolic syndrome OR Dyslipidemia OR Hyperlipidemia OR Combined Hyperlipidemia OR Hyperlipoproteinemia OR Hyperchylomicronemia OR Hypertriglyceridemia OR Hypercholesterolemia OR Familial Hypercholesterolemia OR lipoprotein (a)) AND Country

- (Type 1 diabetes mellitus OR Type 2 diabetes mellitus OR hyperglycemia OR high blood glucose OR glucose impairment OR Secondary diabetes OR gestational diabetes OR Fasting blood glucose OR hypoglycemia OR hyperinsulinemia OR HBA1c OR Diabetic complications OR diabetic nephropathy OR diabetic retinopathy OR diabetic ketoacidosis OR diabetic septic foot OR diabetic neuropathy OR non ketotic diabetic coma) AND Country

- (Chronic obstructive pulmonary disease OR chronic bronchitis OR bronchiectasis OR emphysema OR asthma OR lung fibrosis OR pulmonary fibrosis OR lung hyperinflation OR dyspnea OR bronchodilators OR pulmonary function) AND Country

  (Cancer OR neoplasm OR malignancy OR tumor OR radiotherapy OR chemotherapy OR biopsy OR tumor markers OR palliative care)AND Country

- (Tobacco OR smoking OR narghile OR hubble-bubble OR hookah OR water pipe OR cigarette smoking OR nicotine OR Alcohol OR drinking habits) AND Country

- (Nutrition OR diet OR obesity OR anti- oxidant activity OR BMI OR overweight OR dietary patterns OR macronutrients OR micronutrients OR eating habits OR food security OR Physical inactivity OR physical activity OR sedentary lifestyle OR exercise OR lack of exercise) AND Country

- (Health policy OR policy OR quality indicators OR primary health care OR healthcare OR cost effectiveness OR cost-benefit OR cost OR human resources OR financial) AND (cardiovascular disease OR hypertension OR diabetes mellitus OR cancer OR asthma OR chronic obstructive pulmonary disease OR obesity OR hyperlipidemia) AND Country

- (Risk factors OR prevention OR public health OR prevalence OR incidence OR primary health care) AND Kuwait AND (cardiovascular disease OR hypertension OR diabetes mellitus OR cancer OR asthma OR chronic obstructive pulmonary disease OR obesity OR hyperlipidemia)

- (Genetic polymorphism OR gene polymorphism OR genetic pedigree OR SNP OR GWAS OR genotype OR gene variants OR mutation OR risk allele OR genetic risk factors OR genetic analysis OR Candidate gene OR gene) AND (cardiovascular disease OR hypertension OR diabetes mellitus OR cancer OR asthma OR chronic obstructive pulmonary disease OR obesity OR hyperlipidemia) AND Country

Country

- exp morocco/ or exp south sudan/ or exp sudan/ or exp bahrain/ or exp iraq/ or exp kuwait/ or exp lebanon/
- (palestin* or Gaza* or (west adj bank)).tw.
  (morocc* or sudan* or Bahrain* or Iraq* or Kuwait* or lebanon or lebanese).tw.
