# Supplementary material for: Does published research on non-communicable disease (NCD) in Arab countries reflect NCD disease burden?
Source: PLoS One. 2017 Jun 2;12(6):e0178401. doi: 10.1371/journal.pone.0178401 (PMC5456081; doi:10.1371/journal.pone.0178401)
Supplement: S2 File — (DOCX) [file pone.0178401.s002.docx]

| 99 | Nutrition and Health | Musaiger AO | 2000 |  | http://www.ncbi.nlm.nih.gov/pubmed/10840814 | | | |
| --- | --- | --- | --- | --- | --- | --- | --- | --- |
| 136 | Annals of Saudi Medicine | Modebe O | 2000 |  | http://www.ncbi.nlm.nih.gov/pubmed/17322719 | | | |
| 94 | International Journal of Clinical Pharmacology Therapy | Jassim Al Khaja KA | 2000 | 0 | http://www.ncbi.nlm.nih.gov/pubmed/11125870 | | | |
| 96 | Nicotine and Tobacco Research | Hamadeh RR | 2000 | 0 | http://www.ncbi.nlm.nih.gov/pubmed/11072442 | | | |
| 97 | International Journal of Obesity and Related Metabolic Disorders | Musaiger AO | 2000 | 0 | http://www.ncbi.nlm.nih.gov/pubmed/11033976 | | | |
| 98 | Annals of Human Biology | Musaiger AO | 2000 | 0 | http://www.ncbi.nlm.nih.gov/pubmed/11023120 | | | |
| 101 | Neurosciences | Al-Jishi AA | 2000 | 0 | http://www.ncbi.nlm.nih.gov/pubmed/24276659 | | | |
| 83 | Eastern Mediterranean health journal | Hamadeh RR | 2000 | 0 | http://www.ncbi.nlm.nih.gov/pubmed/12197332 | | | |
| 181 | Annals of Saudi Medicine | Al-Mahroos F | 2000 | 0 | http://www.ncbi.nlm.nih.gov/pubmed/17322704 | | | |
| 200 | Eastern Mediterranean health journal | Hamadeh RR | 2000 | 0 | http://www.ncbi.nlm.nih.gov/pubmed/11370329 | | | |
| 195 | Nutrition and Health | Musaiger AO | 2000 | 0 | http://www.ncbi.nlm.nih.gov/pubmed/10840809 | | | |
| 128 | Saudi Journal of Kidney Disease and Transplantation | Jamsheer NS | 2000 | 0 | http://www.ncbi.nlm.nih.gov.ezproxy.aub.edu.lb/pubmed/18209315 | | | |
| 88 | Eastern Mediterranean health journal | al-Mahroos R | 2000 | 0 | http://www.ncbi.nlm.nih.gov/pubmed/11794072 | | | |
| 100 | International Journal of Epidemiology | Al-Mahroos F | 2000 | 0 | http://www.ncbi.nlm.nih.gov/pubmed/10750606 | | | |
| 95 | International Journal of Food Sciences and Nutrition | Musaiger AO | 2000 | 0 | http://www.ncbi.nlm.nih.gov/pubmed/11103306 | | | |
| 176 | Journal of oral pathology and medicine | Abdullah BH | 2000 | 0 | http://www.ncbi.nlm.nih.gov/pubmed/10972350 | | | |
| 213 | Journal of obstetrics and gynaecology | Khunda SS | 2000 | 0 | http://www.ncbi.nlm.nih.gov/pubmed/15512636 | | | |
| 258 | Eastern Mediterranean Health Journal | Abdel-Barry JA | 2000 | 0 | http://www.ncbi.nlm.nih.gov/pubmed/11370345 | | | |
| 167 | Eastern Mediterranean Health Journal | al-Alwan NA | 2000 | 0 | http://www.ncbi.nlm.nih.gov/pubmed/?term=DNA+proliferative+index+as+a+marker+in+Iraqi+aneuploid+mammary+carcinoma. | | | |
| 177 | Journal of Ethnopharmacology | Abdel-Hassan IA | 2000 | 0 | http://www.ncbi.nlm.nih.gov/pubmed/?term=The+hypoglycaemic+and+antihyperglycaemic+effect+of+citrullus+colocynthis+fruit+aqueous+extract+in+normal+and+alloxan+diabetic+rabbits. | | | |
| 174 | Saudi Medical Journal | Al-Waiz MM | 2000 | 0 | http://www.ncbi.nlm.nih.gov/pubmed/?term=Squamous+cell+carcinoma+complicating+prurigo+nodularis. | | | |
| 171 | Indian Journal of Cancer | Yahya HI | 2000 | 0 | http://www.ncbi.nlm.nih.gov/pubmed/?term=Acute+Lympoblastic+Leukaemia+in+seventy+Iraqi+adults%3A+clinical+and+haematological+findings+and+outcome+of+therapy. | | | |
| 172 | Eastern Mediterranean Health Journal | al-Alwan NA | 2000 | 0 | http://www.ncbi.nlm.nih.gov/pubmed/?term=Assessment+of+response+to+tamoxifen+among+Iraqi+patients+with+advanced+breast+cancer. | | | |
| 173 | Eastern Mediterranean Health Journal | Taha AS | 2000 | 0 | http://www.ncbi.nlm.nih.gov/pubmed/?term=Flexible+fibreoptic+bronchoscopy+in+Basra%2C+Iraq%3A+a+20-month+experience. | | | |
| 599 | Cytopathology | Hombal SM | 2000 | 0 | http://www.ncbi.nlm.nih.gov.ezproxy.aub.edu.lb/pubmed/10714378 | | | |
| 895 | Singapore Medical Journal | Khadadah ME | 2000 | 0 | http://www.ncbi.nlm.nih.gov/pubmed/11063170 | | | |
| 110 | European Journal of Clinical Nutrition | Akanji AO | 2000 | 0 | http://www.ncbi.nlm.nih.gov/pubmed/10878654 | | | |
| 172 | Human Heredity | Haider MZ | 2000 | 0 | http://www.ncbi.nlm.nih.gov/pubmed/?term=High+frequency+of+HLA-DQB1+non-Asp(57)+alleles+in+Kuwaiti+children+with+insulin-dependent+diabetes+mellitus | | | |
| 180 | Scandinavian Journal of Clinical and Laboratory Investigation | Mojiminiyi OA | 2000 | 0 | http://www.ncbi.nlm.nih.gov/pubmed/?term=Evaluation+of+serum+cystatin+C+and+chromogranin+A+as+markers+of+nephropathy+in+patients+with+type+2+diabetes+mellitus | | | |
| 189 | Respiratory Medicine | Mousa K | 2000 | 0 | http://www.ncbi.nlm.nih.gov.ezproxy.aub.edu.lb/pubmed/?term=Technetium+99mTc-DTPA+clearance+in+the+evaluation+of+pulmonary+involvement+in+patients+with+diabetes+mellitus | | | |
| 258 | British Journal of Oral and Maxillofacial Surgery | Morris RE | 2000 | 0 | http://ac.els-cdn.com/S0266435699902474/1-s2.0-S0266435699902474-main.pdf?_tid=7c63c20e-2540-11e3-9501-00000aacb360&acdnat=1380044644_ae1ae809fb821c8a81c4897f5b334c78 | | | |
| 285 | Diagnostic Cytopathology | Abdulla M | 2000 | 0 | http://www.ncbi.nlm.nih.gov/pubmed/10820531 | | | |
| 343 | Diabetes Research and Clinical Practice | Abdella N | 2000 | 0 | http://www.ncbi.nlm.nih.gov/pubmed/11106832 | | | |
| 387 | Acta Diabetologica | Al-Adsani A | 2000 | 0 | http://www.ncbi.nlm.nih.gov/pubmed/11450506 | | | |
| 574 | Allergy | Ezeamuzie CI | 2000 | 0 | http://www.ncbi.nlm.nih.gov.ezproxy.aub.edu.lb/pubmed/?term=Asthma+in+the+desert%3A+spectrum+of+the+sensitizing+aeroallergens. | | | |
| 575 | International Archives of Allergy and Immunology | Ezeamuzie CI | 2000 | 0 | http://www.ncbi.nlm.nih.gov.ezproxy.aub.edu.lb/pubmed/?term=IgE-mediated+sensitization+to+mould+allergens+among+patients+with+allergic+respiratory+diseases+in+a+desert+environment. | | | |
| 587 | International Journal of Cardiology | Hayat NH | 2000 | 0 | http://www.ncbi.nlm.nih.gov.ezproxy.aub.edu.lb/pubmed/?term=Abolition+of+exercise+induced+positive+U-wave+after+coronary+angioplasty%3A+clinical+implication. | | | |
| 598 | International Archives of Allergy and Immunology | Hijazi Z | 2000 | 0 | http://www.ncbi.nlm.nih.gov.ezproxy.aub.edu.lb/pubmed/?term=Interleukin-4+gene+promoter+polymorphism+%5BC590T%5D+and+asthma+in+Kuwaiti+Arabs. | | | |
| 629 | The Journal of Asthma | Khadadah M | 2000 | 0 | http://www.ncbi.nlm.nih.gov.ezproxy.aub.edu.lb/pubmed/?term=The+association+of+skin+test+reactivity%2C+total+serum+IgE+levels%2C+and+peripheral+blood+eosinophilia+with+asthma+in+Kuwait. | | | |
| 630 | Singapore Medical Journal | Khadadah ME | 2000 | 0 | http://www.ncbi.nlm.nih.gov.ezproxy.aub.edu.lb/pubmed/?term=Clinical+features+and+outcome+of+management+of+severe+acute+asthma+(status+asthmaticus)+in+the+intensive+care+unit+of+a+tertiary+medical+center. | | | |
| 655 | Bulletin of the world health organization | Memon A | 2000 | 0 | http://www.ncbi.nlm.nih.gov.ezproxy.aub.edu.lb/pubmed/11143190 | | | |
| 697 | American journal of hematology | Raghupathy R | 2000 | 0 | http://www.ncbi.nlm.nih.gov/pubmed/?term=Tumor+necrosis+factor-alpha+is+undetectable+in+the+plasma+of+SS+patients+with+elevated+Hb+F | | | |
| 860 | Annals of Clinical Biochemistry | Akanji AO | 2000 | 0 | http://www.ncbi.nlm.nih.gov/pubmed/10817252 | | | |
| 268 | Australasian Radiology | Sheikh M | 2000 | 0 | http://www.ncbi.nlm.nih.gov/pubmed/?term=Fine+needle+aspiration+cytology+(FNAC)+was+performed+under+ultrasound+and+CT+guidance+in+120+cases | | | |
| 298 | Prostaglandins, Leukotriences, and Essential Fatty Acids | Ali M | 2000 | 0 | http://www.ncbi.nlm.nih.gov/pmc/articles/PMC1671000/ | | | |
| 97 | Prostaglandins, Leukotrienes, and Essential Fatty Acids | Ali M | 2000 | 0 | http://www.ncbi.nlm.nih.gov/pubmed/?term=ffect+of+allicin+from+garlic+powder+on+serum+lipids+and+blood+pressure+in+rats+++++++fed+with+a+high+cholesterol+diet | | | |
| 158 | Surgery | Bitar MS | 2000 | 0 | http://www.ncbi.nlm.nih.gov/pubmed/?term=Insulin+and+glucocorticoid-dependent+suppression+of+the+IGF-I+system+in+diabetic+wounds | | | |
| 197 | Pediatrics International | Nandakumaran M | 2000 | 0 | http://www.ncbi.nlm.nih.gov/pubmed/?term=Kinetics+of+palmitic+acid+transport+in+insulin-dependent+diabetic+pregnancies%3A+in+vitro+study | | | |
| 244 | European Journal of Cell Biology | Krajcí D | 2000 | 0 | http://ac.els-cdn.com/S0171933504700412/1-s2.0-S0171933504700412-main.pdf?_tid=1e97d1fe-24ed-11e3-b7d5-00000aab0f27&acdnat=1380008839_4b8d61c8cb4dfbd16d93a2d6bf80d3bb | | | |
| 245 | In Vivo | Lincoln DT | 2000 | 0 | http://www.ncbi.nlm.nih.gov.ezproxy.aub.edu.lb/pubmed/?term=Growth+hormone+(GH)+plays+a+crucial+role+in+stimulating+and+controlling+the+growth%2C+metabolism+and+differentiation+of+many+mammalian | | | |
| 273 | Oncology reports | Temmim L | 2000 | 0 | http://www.ncbi.nlm.nih.gov/pubmed/?term=-+Our+immunohistochemical+results+clearly+demonstrated+the+occurrence+of+growth+hormone+receptors+(GH-R)+in+the+tumour+cells+of+lipomas+and+liposarcomas. | | | |
| 364 | Annals of Clinical Biochemistry | Akanji AO | 2000 | 0 | http://www.ncbi.nlm.nih.gov/pubmed/10817252 | | | |
| 394 | Molecular and Cellular Biochemistry | Al-Bader A | 2000 | 0 | http://www.ncbi.nlm.nih.gov/pubmed/10939622 | | | |
| 644 | Japanese Journal of Pharmacology | Mahmoud F | 2000 | 0 | http://www.ncbi.nlm.nih.gov.ezproxy.aub.edu.lb/pubmed/?term=In+vitro+effects+of+Ginkgolide+B+on+lymphocyte+activation+in+atopic+asthma%3A+comparison+with+cyclosporin+A. | | | |
| 737 | Prostaglandins, Leukotrienes, and Essential Fatty Acids | Thomson M | 2000 | 0 | http://www.ncbi.nlm.nih.gov.ezproxy.aub.edu.lb/pubmed/?term=Thromboxane-B(2)+levels+in+serum+of+rabbits+receiving+a+single+intravenous+dose+of+aqueous+extract+of+garlic+and+onion | | | |
| 878 | Pharmacol Biochem Behav | Bitar MS | 2000 | 0 | http://www.ncbi.nlm.nih.gov/pubmed/11124388 | | | |
| 887 | International Journal of Immunopharmacology | Ezeamuzie CI | 2000 | 0 | http://www.ncbi.nlm.nih.gov/pubmed/10785548 | | | |
| 99 | Diabetes Research and Clinical Practice | Abdella N | 2000 | 0 | http://www.ncbi.nlm.nih.gov/pubmed/10936670 | | | |
| 511 | Annals of Allergy, Asthma and Immunology | Behbehani NA | 2000 | 0 | http://www.ncbi.nlm.nih.gov/pubmed/10923606 | | | |
| 327 | Acta Neurologica Belgica | Deleu D | 2000 | 0 | http://www.ncbi.nlm.nih.gov/pubmed/10934562 | | | |
| 91 | Eastern Medditerranian Health Journal | Saleh EA | 2000 | 0 | http://www.ncbi.nlm.nih.gov/pubmed/?term=Hypertension+and+its+determinants+among+primary-school+children+in+Kuwait%3A+an+++++++epidemiological+study | | | |
| 98 | Acta Cardiologica | al-Adsani A | 2000 | 0 | http://www.ncbi.nlm.nih.gov/pubmed/10707754 | | | |
| 423 | International Journal of Food Sciences and Nutrition | Al-Isa AN | 2000 | 0 | http://www.ncbi.nlm.nih.gov/pubmed/11027033 | | | |
| 549 | Medicinski Arhiv | Dobardzi? A | 2000 | 1 | http://www.ncbi.nlm.nih.gov/pubmed/10934832 | | | |
| 286 | Acta Cytologica | Abdulla M | 2000 | 0 | http://www.ncbi.nlm.nih.gov/pubmed/10934957 | | | |
| 262 | Neoplasma | Novotny L | 2000 | 0 | http://www.ncbi.nlm.nih.gov/pubmed/10870680 | | | |
| 232 | Analytical and Quantitative Cytology and Histology | Francis IM | 2000 | 0 | http://www.ncbi.nlm.nih.gov.ezproxy.aub.edu.lb/pubmed/?term=To+compare+manual+to+image+analysis+estimation+of+proliferating+cell+nuclear+antigen+(PCNA)+expression+in+paraffin+sections+of+breast+carcinomas | | | |
| 319 | The International Journal of Biological Markers | Behbehani AI | 2000 | 0 | http://www.ncbi.nlm.nih.gov/pubmed/10763141 | | | |
| 51 | J Med Liban | Ammar W | 2000 |  | Not Found | |  |  |
| 76 | J Med Liban | Azar S T | 2000 |  | Not Found | |  |  |
| 602 | Gastrointestinal Endoscopy | El-Khalil T | 2000 |  | http://www.ncbi.nlm.nih.gov/pubmed/?term=Sigmoid+lipoma+mimicking+carcinoma%3A+case+report+with+review+of+diagnosis+and+management. | | | |
| 608 | J Med Liban | Frem GJ | 2000 |  | http://www.ncbi.nlm.nih.gov//pubmed/11503534 | | | |
| 636 | J Med Liban | Jazra C | 2000 |  | http://www.ncbi.nlm.nih.gov/pubmed/11503527 | | | |
| 660 | J Med Liban | Maroun T | 2000 |  | http://www.ncbi.nlm.nih.gov.ezproxy.aub.edu.lb/pubmed/11503528 | | | |
| 107 | J Med Liban | Bulbul M A | 2000 | 0 | http://www.ncbi.nlm.nih.gov/pubmed/?term=The+value+of+free+and+total+prostate+specific+antigen+in+identifying+patients+for+prostatic+biopsy+and+its+relationship+to+Gleason+score+and+number+of+positive+cores | | | |
| 973 | J Nucl Cardiol | Dakik HA | 2000 | 0 | http://www.ncbi.nlm.nih.gov/pubmed/10958284 | | | |
| 975 | Clinical cardiology | Dakik HA | 2000 | 0 | http://www.ncbi.nlm.nih.gov/pubmed/10894446 | | | |
| 537 | Eastern Mediterranean health journal | Yusef J I | 2000 | 0 | http://www.ncbi.nlm.nih.gov/pubmed/?term=Management+of+diabetes+mellitus+and+hypertension+at+UNRWA+primary+health+care+facilities+in+Lebanon | | | |
| 570 | J Clin Endocrinol Metab | Azar ST | 2000 | 0 | http://www.ncbi.nlm.nih.gov/pubmed/11134127 | | | |
| 641 | European Addiction Research | Karam E | 2000 | 0 | http://www.ncbi.nlm.nih.gov/pubmed/?term=Use+and+abuse+of+licit+and+illicit+substances%3A+prevalence+and+risk+factors+among+students+in+Lebanon. | | | |
| 301 | The Urologic clinics of North America | Jabbour M E | 2000 | 0 | http://www.ncbi.nlm.nih.gov/pubmed/?term=jabbour+2000+Primary+percutaneous+approach+to+upper+urinary+tract+transitional+cell+carcinoma | | | |
| 569 | J Med Liban | Azar S | 2000 | 0 | http://www.ncbi.nlm.nih.gov/pubmed/11503533 | | | |
| 578 | J Med Liban | Birbari AE | 2000 | 0 | http://www.ncbi.nlm.nih.gov/pubmed/12489586 | | | |
| 579 | J Med Liban | Birbari AE | 2000 | 0 | http://www.ncbi.nlm.nih.gov/pubmed/11028155 | | | |
| 91 | Leukemia | Bazarbachi A | 2000 | 0 | http://www.ncbi.nlm.nih.gov/pubmed/?term=Evidence+against+a+direct+cytotoxic+effect+of+alpha+interferon+and+zidovudine+in+HTLV-I+associated+adult+T+cell+leukemia%2Flymphoma | | | |
| 577 | Eur J Pharm Sci | Bikhazi AB | 2000 | 0 | http://www.ncbi.nlm.nih.gov/pubmed/11033073 | | | |
| 611 | Phytomedicine | Gali-Muhtasib H | 2000 | 0 | http://www.ncbi.nlm.nih.gov/pubmed/?term=Chemopreventive+effects+of+sage+oil+on+skin+papillomas+in+mice. | | | |
| 981 | Nutr Cancer | Gali-Muhtasib HU | 2000 | 0 | http://www.ncbi.nlm.nih.gov/pubmed/10965522 | | | |
| 67 | J Med Liban | Attieh E | 2000 | 1 | http://www.ncbi.nlm.nih.gov/pubmed/11268568 | | | |
| 276 | J Med Liban | Hamzeh F | 2000 | 0 | http://www.ncbi.nlm.nih.gov/pubmed/?term=Febrile+neutropenia+in+cancer+patients+in+a+tertiary+care+medical+center+in+Lebanon%3A+microbial+spectrum+and+outcome | | | |
| 55 | Current hypertension reports | Aoun S | 2000 | 0 | http://www.ncbi.nlm.nih.gov/pubmed/10995524 | | | |
| 217 | Journal français d'ophtalmologie | El Mansouri Y | 2000 | 1 | http://www.ncbi.nlm.nih.gov/pubmed/10733353 | | | |
| 1036 | Annales de Cardiologie et d'Angéiologie | Benyass A | 2000 | 1 | http://www.ncbi.nlm.nih.gov/pubmed/12555478 | | | |
| 1120 | Progrès en urologie | el Khader K | 2000 | 1 | http://www.ncbi.nlm.nih.gov/pubmed/11217568 | | | |
| 1121 | Progrès en urologie | Tazi K | 2000 | 1 | http://www.ncbi.nlm.nih.gov/pubmed/11217563 | | | |
| 1122 | European journal of gynaecological oncology | Mansouri H | 2000 | 1 | http://www.ncbi.nlm.nih.gov/pubmed/11214624 | | | |
| 1125 | Revue de laryngologie - otologie - rhinologie | Sahraoui S | 2000 | 1 | http://www.ncbi.nlm.nih.gov/pubmed/11109881 | | | |
| 1126 | Journal de radiologie | Cherkaoui M | 2000 | 1 | http://www.ncbi.nlm.nih.gov/pubmed/11104982 | | | |
| 1127 | Archives de pédiatrie | Zafad S | 2000 | 1 | http://www.ncbi.nlm.nih.gov/pubmed/11075263 | | | |
| 1128 | Progrès en urologie | Tazi K | 2000 | 1 | http://www.ncbi.nlm.nih.gov/pubmed/11064906 | | | |
| 1130 | Journal de radiologie | El Otmany A | 2000 | 1 | http://www.ncbi.nlm.nih.gov/pubmed/10992100 | | | |
| 1131 | Annales d'Urologie | Zrara I | 2000 | 1 | http://www.ncbi.nlm.nih.gov/pubmed/10953800 | | | |
| 1132 | Annales d'Urologie | Joual A | 2000 | 1 | http://www.ncbi.nlm.nih.gov/pubmed/10953794 | | | |
| 1133 | Progrès en urologie | Tazi K | 2000 | 1 | http://www.ncbi.nlm.nih.gov/pubmed/10951945 | | | |
| 1134 | Progrès en urologie | Benchekroun A | 2000 | 1 | http://www.ncbi.nlm.nih.gov/pubmed/10951940 | | | |
| 1136 | Progrès en urologie | Benchekroun A | 2000 | 1 | http://www.ncbi.nlm.nih.gov/pubmed/10857136 | | | |
| 1137 | Archives des maladies du coeur et des vaisseaux | Bennis A | 2000 | 1 | http://www.ncbi.nlm.nih.gov/pubmed/10816809 | | | |
| 1138 | Annales d'Urologie | Beddouch A | 2000 | 1 | http://www.ncbi.nlm.nih.gov/pubmed/10763424 | | | |
| 1139 | Annales d'Urologie | Debbagh A | 2000 | 1 | http://www.ncbi.nlm.nih.gov/pubmed/10763420 | | | |
| 1141 | Journal français d'ophtalmologie | Boutimzine N | 2000 | 0 | http://www.ncbi.nlm.nih.gov/pubmed/10705121 | | | |
| 201 | Revue des maladies respiratoires | El biaze M | 2000 | 1 | http://www.ncbi.nlm.nih.gov/pubmed/10951962 | | | |
| 341 | Genetic testing | Kreiss Y | 2000 | 0 | http://www.ncbi.nlm.nih.gov/pubmed/11216667 | | | |
| 1123 | Cancer radiothérapie | Sahraoui S | 2000 | 1 | http://www.ncbi.nlm.nih.gov/pubmed/11191849 | | | |
| 1124 | Diabetes and Metabolism | Chadli A | 2000 | 1 | http://www.ncbi.nlm.nih.gov/pubmed/11173722 | | | |
| 1037 | Annales de Cardiologie et d'Angéiologie | Tazi-MÃ©zalek Z | 2000 | 1 | http://www.ncbi.nlm.nih.gov/pubmed/12555320 | | | |
| 19 | Atherosclerosis | Adlouni A | 2000 | 0 | http://www.ncbi.nlm.nih.gov/pubmed/10998472 | | | |
| 107 | Atherosclerosis | Bennani-Kabchi N | 2000 | 0 | http://www.ncbi.nlm.nih.gov/pubmed/10781635 | | | |
| 322 | Journal of Ethnopharmacology | Jaouhari JT | 2000 | 0 | http://www.ncbi.nlm.nih.gov/pubmed/10661879 | | | |
| 324 | Journal of Ethnopharmacology | Jouad H | 2000 | 0 | http://www.ncbi.nlm.nih.gov/pubmed/10904160 | | | |
| 1135 | Annales Pharmaceutiques Françaises | Bennani-Kabchi N | 2000 | 1 | http://www.ncbi.nlm.nih.gov/pubmed/10915976 | | | |
| 1140 | International journal of Cancer | Dardari R | 2000 | 1 | http://www.ncbi.nlm.nih.gov/pubmed/10728597 | | | |
| 68 | Public Health Rev | Sha'ban DA | 2000 |  |  |  |  |  |
| 65 | Eastern Mediterranean health journal = La revue de sante de la Medoterranee orientale = al-Majallah | Husseini A | 2000 | 0 | http://www.ncbi.nlm.nih.gov/pubmed/12197326 | | | |
| 69 | scandinavian journal of clinical & laboratory investigation | Husseini A | 2000 | 0 | http://www.ncbi.nlm.nih.gov/pubmed/11129061 | | | |
| 70 | J Ethnopharmacol | Ali-Shtayeh MS | 2000 | 0 | http://www.ncbi.nlm.nih.gov/pubmed/11025160 | | | |
| 130 | The Journal of asthma | Hasan MM | 2000 | 0 | http://www.ncbi.nlm.nih.gov.ezproxy.aub.edu.lb/pubmed/10883746 | | | |
| 131 | Diabetic medicine: a journal of the British Diabetic Association | Husseini A | 2000 | 0 | http://www.ncbi.nlm.nih.gov.ezproxy.aub.edu.lb/pubmed/11110509 | | | |
| 158 | Public Health Rev | Abdeen Z | 2000 | 0 | http://www.ncbi.nlm.nih.gov/pubmed/11411275 | | | |
| 97 | Lancet | Kark JD | 2000 | 0 | http://www.ncbi.nlm.nih.gov.ezproxy.aub.edu.lb/pubmed/11052588 | | | |
| 15 | Saudi medical journal | Ahmed A M | 2000 | 0 | http://www.ncbi.nlm.nih.gov/pubmed/?term=Diabetic+autonomic+neuropathy+Saudi+medical+journal+Ahmed%2C+A.+M. | | | |
| 68 | Eastern Mediterranean health journal | Elzubier A G | 2000 | 0 | http://www.ncbi.nlm.nih.gov/pubmed/11370321 | | | |
| 86 | Anticancer research | Loro L L | 2000 | 0 | http://www.ncbi.nlm.nih.gov/pubmed/?term=Apoptosis+and+expression+of+Bax+and+Bcl-2+in+snuff-+and+non-snuff+associated+oral+squamous+cell+carcinomas | | | |
| 90 | Saudi Medical Journal | Al-Mannai A | 2001 | 0 | http://www.ncbi.nlm.nih.gov/pubmed/11590455 | | | |
| 91 | Pharmacoepidemiology Drug Safety | Jassim al Khaja KA | 2001 | 0 | http://www.ncbi.nlm.nih.gov/pubmed/11501335 | | | |
| 92 | Annals of Human Biology | Musaiger AO | 2001 | 0 | http://www.ncbi.nlm.nih.gov/pubmed/11393341 | | | |
| 93 | Journal of Clinical Pharmacy and Therapeutics | Jassim Al Khaja KA | 2001 | 0 | http://www.ncbi.nlm.nih.gov/pubmed/11286605 | | | |
| 87 | Fundamental and Clinical Pharmacology | Kassab S | 2001 | 0 | http://www.ncbi.nlm.nih.gov/pubmed/11860525 | | | |
| 89 | Annals of Pharmacotherapy | Al Khaja KA | 2001 | 0 | http://www.ncbi.nlm.nih.gov/pubmed/11724081 | | | |
| 140 | Annals of Saudi Medicine | Al-Mahroos F | 2001 | 0 | http://www.ncbi.nlm.nih.gov/pubmed/?term=Obesity+among+adult+Bahraini+population%3A+impact+of+physical+activity+and+educational+level. | | | |
| 216 | British Journal of Radiololgy | Mould RF | 2001 |  | http://www.ncbi.nlm.nih.gov/pubmed/11511490 | | | |
| 164 | Eastern Mediterranean Health Journal | Al-Alwan NA | 2001 | 0 | http://www.ncbi.nlm.nih.gov/pubmed/12596958 | | | |
| 175 | Saudi Medical Journal | Al-Hashimi AH | 2001 | 0 | http://www.ncbi.nlm.nih.gov/pubmed/?term=Lucigenin+chemiluminesence.+A+new+approach+to+study+the+redox+activity+of+Ehrlich+ascetic+tumor+cells. | | | |
| 894 | Oncol Rep | Luqmani YA | 2001 |  | http://www.ncbi.nlm.nih.gov/pubmed/11496304 | | | |
| 284 | Surgical Endoscopy | Abbas M | 2001 | 0 | http://www.ncbi.nlm.nih.gov/pubmed/11727152 | | | |
| 869 | Postgraduate Medical Journal | Al-Adsani A | 2001 | 0 | http://www.ncbi.nlm.nih.gov/pubmed/11161092 | | | |
| 883 | Diagnostic Cytopathology | Das DK | 2001 | 0 | http://www.ncbi.nlm.nih.gov/pubmed/11466810 | | | |
| 88 | Japanese Heart Journal | Zubaid M | 2001 | 0 | https://www.jstage.jst.go.jp/article/jhj/42/6/42_6_669/_pdf | | | |
| 92 | Journal of Diabetes and its Complications | Abdella NA | 2001 | 0 | http://www.ncbi.nlm.nih.gov/pubmed/11522503 | | | |
| 128 | Diabetes Educator | Al-Jasem LI | 2001 | 0 | http://www.ncbi.nlm.nih.gov/pubmed/11912800 | | | |
| 129 | Medical Principles and Practice | Al-Khabbaz AK | 2001 | 0 | http://www.ncbi.nlm.nih.gov/pubmed/?term=Diabetes+mellitus+and+periodontal+health%3A+dentists'+knowledge | | | |
| 144 | Pediatrics International | Alsaeid M | 2001 | 0 | http://www.ncbi.nlm.nih.gov/pubmed/?term=Impact+of+glycemic+control+on+serum+lipoprotein+(a)+in+Arab+children+with+type+1+diabetes | | | |
| 213 | Renal Failure | Varghese K | 2001 | 0 | http://www.ncbi.nlm.nih.gov/pubmed/11725913 | | | |
| 274 | The Breast | Temmim L | 2001 | 0 | http://ac.els-cdn.com/S0960977600902236/1-s2.0-S0960977600902236-main.pdf?_tid=913624c8-3a86-11e3-8082-00000aab0f6c&acdnat=1382383718_fc715220b048e66bae1aa0101f789c3c | | | |
| 306 | Transplantation Proceedings | Al Mousawi MS | 2001 | 0 | http://www.ncbi.nlm.nih.gov/pubmed/11498169 | | | |
| 586 | Clincal cardiology | Hayat N | 2001 | 0 | http://www.ncbi.nlm.nih.gov.ezproxy.aub.edu.lb/pubmed/?term=Transmyocardial+laser+revascularization%3A+is+the+enthusiasm+justified%3F | | | |
| 588 | Clincal cardiology | Hayat NJ | 2001 | 0 | http://www.ncbi.nlm.nih.gov.ezproxy.aub.edu.lb/pubmed/?term=Staged+revascularization+in+critically+ill+patients+with+coronary+artery+disease. | | | |
| 597 | Journal of Tropical Pediatrics | Hijazi Z | 2001 | 0 | http://www.ncbi.nlm.nih.gov.ezproxy.aub.edu.lb/pubmed/?term=Influence+of+consanguinity+and+IgE+receptor+genotypes+on+clinical+manifestations+of+asthma+in+Kuwaiti+children. | | | |
| 699 | Nutrition | Ramadan J | 2001 | 0 | http://ac.els-cdn.com/S0899900700005499/1-s2.0-S0899900700005499-main.pdf?_tid=1a040f72-52bf-11e3-8155-00000aab0f01&acdnat=1385046828_63895275359b81c5b0aa384ddf7f812d | | | |
| 253 | Clinical otolaryngology and allied sciences | McDermott AL | 2001 | 0 | http://www.ncbi.nlm.nih.gov.ezproxy.aub.edu.lb/pubmed/11309046 | | | |
| 891 | Neoplasma | Novotny L | 2001 | 0 | http://www.ncbi.nlm.nih.gov/pubmed/11712672 | | | |
| 90 | Hormone and Metabolic Research | Bitar MS | 2001 | 0 | http://www.ncbi.nlm.nih.gov/pubmed/?term=Co-administration+of+etomoxir+and+RU-486+mitigates+insulin+resistance+in+hepatic++++++++and+muscular+tissues+of+STZ-induced+diabetic+rats | | | |
| 93 | Phytotherapy Research | Gibbon S | 2001 | 0 | http://www.ncbi.nlm.nih.gov/pubmed/?term=Antihypertensive+effect+of+an+aqueous+extract+of+Zygophyllum+coccineum+L.+in+++++++rats | | | |
| 95 | Kidney and Blood Pressure Research | Al-Qattan KK | 2001 | 0 | http://www.ncbi.nlm.nih.gov/pubmed/?term=Different+levels+of+hypertension+induce+opposite+diuretic+behaviors+from+the+++++++nonclipped+kidney+in+the+rat+two-kidney%2C+one-clip+model | | | |
| 96 | Prostaglandins, Leukotrienes and Essential Fatty Acids | Al-Qattan KK | 2001 | 0 | http://www.ncbi.nlm.nih.gov/pubmed/?term=Thromboxane-B2%2C+prostaglandin-E2+and+hypertension+in+the+rat+2-kidney+1-clip+++++++model%3A+a+possible+mechanism+of+the+garlic+induced+hypotension | | | |
| 231 | International journal of Cancer | Ford CHG | 2001 | 0 | http://onlinelibrary.wiley.com.ezproxy.aub.edu.lb/doi/10.1002/ijc.1262/pdf | | | |
| 309 | The Journal of Pathology | Al Mulla F | 2001 | 0 | http://www.ncbi.nlm.nih.gov/pubmed/11745690 | | | |
| 573 | Biochemical pharmacology | Ezeamuzie CI | 2001 | 0 | http://www.sciencedirect.com.ezproxy.aub.edu.lb/science/article/pii/S000629520100613X?np=y | | | |
| 230 | International journal of hematology | El-sonbaty MR | 2001 | 0 | http://www.ncbi.nlm.nih.gov.ezproxy.aub.edu.lb/pubmed/?term=Both+non-Hodgkin's+lymphoma+(NHL)+in+pregnancy+and+acute+spontaneous+tumor-lysis+(ASTL)+syndrome+are+rare. | | | |
| 295 | Postgraduate Medical Journal | Al Adsani N | 2001 | 0 | http://www.ncbi.nlm.nih.gov/pmc/articles/PMC1741912/ | | | |
| 89 | Renal Failure | Varghese K | 2001 | 0 | http://www.ncbi.nlm.nih.gov/pubmed/11777319 | | | |
| 94 | International Journal of Food Sciences and Nutrition | Jackson RT | 2001 | 0 | http://www.ncbi.nlm.nih.gov/pubmed/11474894 | | | |
| 228 | Acta Cardiologica | Zubaid M | 2001 | 0 | http://www.ncbi.nlm.nih.gov/pubmed/?term=Thrombolytic+therapy+in+acute+myocardial+infarction%3A+practice+pattern+at+an+Arab+Middle+Eastern+centre | | | |
| 289 | Journal of Clinical Pharmacy and Therapeutics | Abiaka C | 2001 | 0 | http://www.ncbi.nlm.nih.gov/pubmed/11493368 | | | |
| 290 | Cancer Detection and Prevention | Abiaka C | 2001 | 0 | http://www.ncbi.nlm.nih.gov/pubmed/11425266 | | | |
| 318 | Acta Oncologica | Awadh Behebehani N | 2001 | 0 | http://www.ncbi.nlm.nih.gov/pubmed/11207008 | | | |
| 331 | Analytical and Quantitative Cytology and Histology | Dey P | 2001 | 0 | http://www.ncbi.nlm.nih.gov/pubmed/11233740 | | | |
| 349 | Annals of Allergy, Asthma and Immunology | Abul AT | 2001 | 0 | http://www.ncbi.nlm.nih.gov/pubmed/11345294 | | | |
| 272 | Anticancer Research | Temmim L | 2001 | 0 | http://www.ncbi.nlm.nih.gov/pubmed/?term=The+mutation+of+the+p53+gene+is+a+common+phenomenon+in+numerous+human+tumors+including+breast+cancer | | | |
| 38 | J Med Liban | Akel M M | 2001 |  | http://www.ncbi.nlm.nih.gov/pubmed/12243421 | | | |
| 97 | European radiology | Birjawi G A | 2001 |  | Not Found | |  |  |
| 272 | Journal of medical ethics | Hamadeh G N | 2001 |  | Not Found | |  |  |
| 494 | Otolaryngology--head and neck surgery | Shreif J A | 2001 |  | Abstract Not Found | |  |  |
| 26 | European journal of obstetrics, gynecology, and reproductive biology | Abu-Musa A | 2001 | 0 | http://www.ncbi.nlm.nih.gov/pubmed/11728665 | | | |
| 123 | Thyroid | Checrallah A | 2001 | 0 | http://www.ncbi.nlm.nih.gov/pubmed/11575860 | | | |
| 206 | J Med Liban | Farah M | 2001 | 0 | http://www.ncbi.nlm.nih.gov/pubmed/?term=Primary+mediastinal+liposarcoma%3A+a+case+report+and+review+of+the+literature+Farah+2001 | | | |
| 477 | Catheterization and cardiovascular interventions | Sarkis A | 2001 | 0 | http://www.ncbi.nlm.nih.gov/pubmed/?term=Gadolinium-based+coronarography+in+a+patient+with+renal+failure%3A+first+clinical+report | | | |
| 502 | J Med Liban | Slaba S | 2001 | 0 | http://www.ncbi.nlm.nih.gov/pubmed/?term=Slaba+2001+Imaging+of+an+exceptional+tumor%3A+myxoid+chondrosarcoma+of+the+jugular+foramen | | | |
| 972 | J Nucl Cardiol | Dakik HA | 2001 | 0 | http://www.ncbi.nlm.nih.gov/pubmed/11725269 | | | |
| 31 | Journal of the American Geriatrics Society | Abyad A | 2001 | 0 | http://www.ncbi.nlm.nih.gov/pubmed/?term=Health+care+for+older+persons%3A+a+country+profile--Lebanon | | | |
| 108 | The Canadian journal of urology | Bulbul M A | 2001 | 0 | http://www.ncbi.nlm.nih.gov/pubmed/?term=The+value+of+cystoscopy%2C+prostate+biopsy+and+frozen-section+urethral+biopsy+prior+to+orthotopic+neobladder+substitution | | | |
| 326 | J Med Liban | Kattan J | 2001 | 0 | http://www.ncbi.nlm.nih.gov/pubmed/?term=kattan%2CJ+20011+Mesothelioma--asbestos+in+Lebanon%3A+a+problem+to+be+considered | | | |
| 495 | Journal of epidemiology and community health | Sibai A M | 2001 | 0 | http://www.ncbi.nlm.nih.gov/pubmed/?term=Non-communicable+disease+mortality+rates+using+the+verbal+autopsy+in+a+cohort+of+middle+aged+and+older+populations+in+Beirut+during+wartime%2C+1983-93 | | | |
| 503 | Journal of epidemiology and community health | Sleilaty G | 2001 | 0 | http://www.ncbi.nlm.nih.gov/pubmed/?term=Postoperative+oral+amiodarone+versus+oral+bisoprolol+as+prophylaxis+against+atrial+fibrillation+after+coronary+artery+bypass+graft+surgery%3A+a+prospective+randomized+trial | | | |
| 571 | Eastern Mediterranean health journal | Baddoura R | 2001 | 0 | http://www.ncbi.nlm.nih.gov/pubmed/15332785 | | | |
| 711 | Aust N Z J Public Health | Tamim H | 2001 | 0 | http://www.ncbi.nlm.nih.gov/pubmed/11357917 | | | |
| 731 | J Thromb Thrombolysis | Irani-Hakime N | 2001 | 0 | http://www.ncbi.nlm.nih.gov/pubmed/?term=Factor+V+R506Q+mutation-Leiden%3A+an+independent+risk+factor+for+venous+thrombosis+but+not+coronary+artery+disease. | | | |
| 942 | Am J Epidemiol | Sibai AM | 2001 | 0 | http://www.ncbi.nlm.nih.gov/pubmed/11447045 | | | |
| 1005 | Arch Mal Coeur Vaiss | Kossaify A | 2001 | 0 | http://www.ncbi.nlm.nih.gov/pubmed/11828921 | | | |
| 523 | J Med Liban | Usta J | 2001 | 0 | http://www.ncbi.nlm.nih.gov/pubmed/?term=2001+usta+Asthma+in+primary+care | | | |
| 957 | Am Heart J | Azar RR | 2001 | 0 | http://www.ncbi.nlm.nih.gov/pubmed/11376298 | | | |
| 970 | J Med Liban | Dakik HA | 2001 | 0 | http://www.ncbi.nlm.nih.gov/pubmed/12243422 | | | |
| 90 | Virus research | Bazarbachi A | 2001 | 0 | http://www.ncbi.nlm.nih.gov/pubmed/?term=Treatment+of+adult+T-cell+leukaemia%2Flymphoma%3A+current+strategy+and+future+perspectives | | | |
| 152 | The hematology journal | Darwiche N | 2001 | 0 | http://www.ncbi.nlm.nih.gov/pubmed/?term=Retinoic+acid+dramatically+enhances+the+arsenic+trioxide-induced+cell+cycle+arrest+and+apoptosis+in+retinoic+acid+receptor+alpha-positive+human+T-cell+lymphotropic+virus+type-I-transformed+cells | | | |
| 235 | Oncology reports | Gali-Muhtasib H U | 2001 | 0 | http://www.ncbi.nlm.nih.gov/pubmed/11295102 | | | |
| 588 | Cell Mol Biol (Noisy-le-grand) | Chehade F | 2001 | 0 | http://www.ncbi.nlm.nih.gov/pubmed/11441960 | | | |
| 626 | Carcinogenesis | Hatoum A | 2001 | 0 | http://www.ncbi.nlm.nih.gov/pubmed/?term=Overexpression+of+retinoic+acid+receptors+alpha+and+gamma+into+neoplastic+epidermal+cells+causes+retinoic+acid-induced+growth+arrest+and+apoptosis. | | | |
| 980 | Nutr Cancer | Gali-Muhtasib HU | 2001 | 0 | http://www.ncbi.nlm.nih.gov/pubmed/11588891 | | | |
| 64 | J Med Liban | Ashkar K | 2001 | 0 | http://www.ncbi.nlm.nih.gov/pubmed/?term=Cancer+screening+for+the+primary+care+physician+Ashkar+2001 | | | |
| 1 | The Canadian Nurse | | 2001 |  | http://www.ncbi.nlm.nih.gov/pubmed/11865600 | | | |
| 385 | J Urol | Mansouri H | 2001 | 0 | http://www.ncbi.nlm.nih.gov/pubmed/11342954 | | | |
| 386 | Journal of Clinical Oncology | Mansouri H | 2001 | 0 | http://www.ncbi.nlm.nih.gov/pubmed/11331337 | | | |
| 387 | Journal of Clinical Oncology | Mansouri H | 2001 | 0 | http://www.ncbi.nlm.nih.gov/pubmed/11387369 | | | |
| 1035 | Annales de Cardiologie et d'Angéiologie | Belmadani K | 2001 | 1 | http://www.ncbi.nlm.nih.gov/pubmed/12555596 | | | |
| 1072 | Revue de pneumologie clinique | Mahassini N | 2001 | 1 | http://www.ncbi.nlm.nih.gov/pubmed/11924152 | | | |
| 1073 | Journal of neuroradiology | Taleb A | 2001 | 1 | http://www.ncbi.nlm.nih.gov/pubmed/11924146 | | | |
| 1078 | Journal of neuroradiology | Boumdin H | 2001 | 1 | http://www.ncbi.nlm.nih.gov/pubmed/11894527 | | | |
| 1080 | Progrès en urologie | Touiti D | 2001 | 1 | http://www.ncbi.nlm.nih.gov/pubmed/11859671 | | | |
| 1081 | Progrès en urologie | Ameur A | 2001 | 1 | http://www.ncbi.nlm.nih.gov/pubmed/11859670 | | | |
| 1082 | Progrès en urologie | Dakir M | 2001 | 1 | http://www.ncbi.nlm.nih.gov/pubmed/11859663 | | | |
| 1086 | Revue de laryngologie - otologie - rhinologie | el Kohen A | 2001 | 1 | http://www.ncbi.nlm.nih.gov/pubmed/11799865 | | | |
| 1087 | Annales d'Urologie | Debbagh A | 2001 | 1 | http://www.ncbi.nlm.nih.gov/pubmed/11774771 | | | |
| 1088 | Progrès en urologie | Dakir M | 2001 | 1 | http://www.ncbi.nlm.nih.gov/pubmed/11761694 | | | |
| 1089 | Progrès en urologie | Benchekroun A | 2001 | 1 | http://www.ncbi.nlm.nih.gov/pubmed/11761691 | | | |
| 1090 | Progrès en urologie | Khaleq K | 2001 | 1 | http://www.ncbi.nlm.nih.gov/pubmed/11761690 | | | |
| 1091 | Archives des maladies du coeur et des vaisseaux | Nour-Eddine M | 2001 | 1 | http://www.ncbi.nlm.nih.gov/pubmed/11725718 | | | |
| 1093 | Annales d'Urologie | Kasmaoui E | 2001 | 1 | http://www.ncbi.nlm.nih.gov/pubmed/11675969 | | | |
| 1094 | Annales d'Urologie | Benchekroun A | 2001 | 1 | http://www.ncbi.nlm.nih.gov/pubmed/11675968 | | | |
| 1095 | Annales d'Urologie | Karmouni T | 2001 | 1 | http://www.ncbi.nlm.nih.gov/pubmed/11675964 | | | |
| 1096 | Annales d'Urologie | Tahri A | 2001 | 1 | http://www.ncbi.nlm.nih.gov/pubmed/11675960 | | | |
| 1097 | Annales d'Urologie | Fekak H | 2001 | 1 | http://www.ncbi.nlm.nih.gov/pubmed/11675959 | | | |
| 1098 | Progrès en urologie | Louafy L | 2001 | 1 | http://www.ncbi.nlm.nih.gov/pubmed/11512467 | | | |
| 1099 | Annales d'Urologie | Benchekroun A | 2001 | 1 | http://www.ncbi.nlm.nih.gov/pubmed/11496601 | | | |
| 1101 | Annales de Chirugie | Raiss M | 2001 | 1 | http://www.ncbi.nlm.nih.gov/pubmed/11447798 | | | |
| 1102 | Revue de stomatologie et de chirurgie maxillo-faciale | Oujilal A | 2001 | 1 | http://www.ncbi.nlm.nih.gov/pubmed/11446141 | | | |
| 1103 | Journal de radiologie | Ousehal A | 2001 | 1 | http://www.ncbi.nlm.nih.gov/pubmed/11428215 | | | |
| 1105 | Gynécologie, obstétrique & fertilité | Zamiati S | 2001 | 1 | http://www.ncbi.nlm.nih.gov/pubmed/11406935 | | | |
| 1106 | Progrès en urologie | Benchekroun A | 2001 | 1 | http://www.ncbi.nlm.nih.gov/pubmed/11400501 | | | |
| 1107 | Progrès en urologie | el Khader K | 2001 | 1 | http://www.ncbi.nlm.nih.gov/pubmed/11400495 | | | |
| 1108 | Chirurgie de la main | Essadki B | 2001 | 1 | http://www.ncbi.nlm.nih.gov/pubmed/11386177 | | | |
| 1109 | Cancer radiothérapie | Sahraoui S | 2001 | 1 | http://www.ncbi.nlm.nih.gov/pubmed/11355580 | | | |
| 1110 | Annales d'Urologie | Sahraoui S | 2001 | 1 | http://www.ncbi.nlm.nih.gov/pubmed/11355283 | | | |
| 1111 | Annales d'Urologie | Benchekroun A | 2001 | 1 | http://www.ncbi.nlm.nih.gov/pubmed/11355278 | | | |
| 1113 | Annales de Dermatologie et de Vénéréologie | El Haouri M | 2001 | 1 | http://www.ncbi.nlm.nih.gov/pubmed/11319392 | | | |
| 1114 | Progrès en urologie | Tazi K | 2001 | 1 | http://www.ncbi.nlm.nih.gov/pubmed/11296655 | | | |
| 1115 | Neurochirurgie | Akhaddar A | 2001 | 1 | http://www.ncbi.nlm.nih.gov/pubmed/11283455 | | | |
| 1116 | Annales d'Urologie | Tahri A | 2001 | 1 | http://www.ncbi.nlm.nih.gov/pubmed/11233327 | | | |
| 1117 | Annales d'Urologie | Fekkak H | 2001 | 1 | http://www.ncbi.nlm.nih.gov/pubmed/11233322 | | | |
| 1118 | Annales d'Urologie | Dahami Z | 2001 | 1 | http://www.ncbi.nlm.nih.gov/pubmed/11233315 | | | |
| 46 | J Neurosurg Sci | Amarti A | 2001 | 0 | http://www.ncbi.nlm.nih.gov/pubmed/11731741 | | | |
| 172 | International Journal of Cancer | Dardari R | 2001 | 0 | http://www.ncbi.nlm.nih.gov/pubmed/11291059 | | | |
| 309 | Anticancer Research | Iscovich J | 2001 | 0 | http://www.ncbi.nlm.nih.gov/pubmed/11396230 | | | |
| 325 | Journal of Ethnopharmacology | Jouad H | 2001 | 0 | http://www.ncbi.nlm.nih.gov/pubmed/11535361 | | | |
| 482 | Biochemical pharmacology | Squali Houssaïni FZ | 2001 | 0 | http://www.ncbi.nlm.nih.gov/pubmed/11325213 | | | |
| 1079 | Revue des maladies respiratoires | Yazidi AA | 2001 | 1 | http://www.ncbi.nlm.nih.gov/pubmed/11887770 | | | |
| 1092 | International Journal of Tuberculosis and Lung Disease | Laraqui CH | 2001 | 1 | http://www.ncbi.nlm.nih.gov/pubmed/11718134 | | | |
| 1100 | Gynécologie, obstétrique & fertilité | Yahyaoui O | 2001 | 1 | http://www.ncbi.nlm.nih.gov/pubmed/11462957 | | | |
| 331 | Diabetes and Metabolism | Kadiri A | 2001 | 0 | http://www.ncbi.nlm.nih.gov/pubmed/11547222 | | | |
| 61 | Monaldi Arch Chest Dis | Bartal M | 2001 | 0 | http://www.ncbi.nlm.nih.gov/pubmed/11980288 | | | |
| 171 | Journal of Clinical Microbiology | Dardari R | 2001 | 0 | http://www.ncbi.nlm.nih.gov/pubmed/11526145 | | | |
| 326 | Journal of Ethnopharmacology | Jouad H | 2001 | 0 | http://www.ncbi.nlm.nih.gov/pubmed/11390130 | | | |
| 1104 | Annales Pharmaceutiques Françaises | Rhiouani H | 2001 | 1 | http://www.ncbi.nlm.nih.gov/pubmed/11427823 | | | |
| 1112 | Clin Exp Hypertens | El Bardai S | 2001 | 1 | http://www.ncbi.nlm.nih.gov/pubmed/11349824 | | | |
| 1119 | Journal de radiologie | Ousehal A | 2001 | 1 | http://www.ncbi.nlm.nih.gov/pubmed/11223626 | | | |
| 402 | Journal of Nutrition | Mokhtar N | 2001 | 0 | http://www.ncbi.nlm.nih.gov/pubmed/11238780 | | | |
| 271 | Diabetes care | Haddad PS | 2001 | 0 | http://www.ncbi.nlm.nih.gov/pubmed/11289496 | | | |
| 510 | Urol Ann | Tazi K | 2001 | 0 | http://www.ncbi.nlm.nih.gov/pubmed/11233325 | | | |
| 117 | Journal of Ethnopharmacology | Benwahhoud M | 2001 | 0 | http://www.ncbi.nlm.nih.gov/pubmed/11378278 | | | |
| 62 | Eastern Mediterranean health journal = La revue de sante de la Medoterranee orientale = al-Majallah | Abdul-Rahim HF | 2001 | 0 | http://www.ncbi.nlm.nih.gov/pubmed/12596954 | | | |
| 74 | International journal of obesity | Abdul-Rahim HF | 2001 | 0 | http://www.ncbi.nlm.nih.gov.ezproxy.aub.edu.lb/pubmed/11753598 | | | |
| 116 | European journal of clinical nutrition | Stene LC | 2001 | 0 | http://www.ncbi.nlm.nih.gov.ezproxy.aub.edu.lb/pubmed/11528498 | | | |
| 124 | Diabetes care | Abdul-Rahim HF | 2001 | 0 | http://www.ncbi.nlm.nih.gov/pubmed/11213878 | | | |
| 161 | The Israel Medical Association journal: IMAJ | Darwish H | 2001 | 0 | http://www.ncbi.nlm.nih.gov/pubmed/11519397 | | | |
| 28 | Saudi medical journal | Ahmed M E | 2001 | 0 | http://www.ncbi.nlm.nih.gov/pubmed/?term=A+calculus+within+an+anal+fistula+tract+in+a+diabetic+patient | | | |
| 102 | Transactions of the Royal Society of Tropical Medicine and Hygiene | Omer R E | 2001 | 0 | http://www.ncbi.nlm.nih.gov.ezproxy.aub.edu.lb/pubmed/?term=The+role+of+hepatitis+B+and+hepatitis+C+viral+infections+in+the+incidence+of+hepatocellular+carcinoma+in+Sudan | | | |
| 103 | Cancer Causes and Control | Omer R E | 2001 | 0 | http://www.ncbi.nlm.nih.gov/pubmed/?term=Peanut+butter+intake%2C+GSTM1+genotype+and+hepatocellular+carcinoma%3A+a+case-control+study+in+Sudan | | | |
| 62 | Seminars in oncology | el-Mawla N G | 2001 | 0 | http://www.ncbi.nlm.nih.gov/pubmed/?term=Bladder+cancer+in+Africa%3A+update | | | |
| 84 | The Canadian Journal of Urology | Hasan WA | 2002 | 0 | http://www.ncbi.nlm.nih.gov/pubmed/12121583 | | | |
| 133 | Saudi Journal of Kidney Disease and Transplantation | Radhi A | 2002 | 0 | http://www.ncbi.nlm.nih.gov/pubmed/17660676 | | | |
| 81 | Journal of Postgraduate Medicine | Sequeira RP | 2002 | 0 | http://www.ncbi.nlm.nih.gov/pubmed/12432188 | | | |
| 82 | Journal of Evaluation in Clinical Practice | Sequeira RP | 2002 | 0 | http://www.ncbi.nlm.nih.gov/pubmed/12421390 | | | |
| 86 | Journal of Evaluation in Clinical Practice | Al Khaja KA | 2002 | 0 | http://www.ncbi.nlm.nih.gov/pubmed/11882098 | | | |
| 80 | Archives of Dermatology | Al Mahroos M | 2002 | 0 | http://www.ncbi.nlm.nih.gov/pubmed/12437454 | | | |
| 79 | Medical Principles and Practice | Musaiger AO | 2002 | 0 | http://www.ncbi.nlm.nih.gov/pubmed/12444306 | | | |
| 85 | Journal of Biosocial Sciences | Musaiger AO | 2002 | 0 | http://www.ncbi.nlm.nih.gov/pubmed/11926459 | | | |
| 142 | Food Nutr Bull | Tawfeek H | 2002 | 0 | http://www.ncbi.nlm.nih.gov/pubmed/?term=Relationship+between+waist+circumference+and+blood+pressure+among+the+population+in+Baghdad%2C+Iraq. | | | |
| 157 | Eastern Mediterranean Health Journal | Salem MB | 2002 | 0 | http://www.ncbi.nlm.nih.gov/pubmed/?term=Prevalence+of+wheeze+among+preschool+children+in+Basra+governonate%2C+southern+Iraq. | | | |
| 168 | Saudi Medical Journal | Al-Ali MA | 2002 | 0 | http://www.ncbi.nlm.nih.gov/pubmed/12070550 | | | |
| 169 | Journal of the Pakistan Medical Association | al-Naama LM | 2002 | 0 | http://www.ncbi.nlm.nih.gov/pubmed/11963582 | | | |
| 160 | Eastern Mediterranean Health Journal | Garib BT | 2002 | 0 | http://www.ncbi.nlm.nih.gov/pubmed/?term=Flow+cytometric+DNA+analysis+of+oral+squamous+cell+carcinoma+in+Iraqi+patients. | | | |
| 165 | Bollettino chimico farmaceutico | Khallow KI | 2002 | 0 | http://www.ncbi.nlm.nih.gov/pubmed/?term=Preparation%2C+characterization+%26+anticancer+evaluation+of+potassium+N-(p-anisole)-alpha-(2-xanthatophenyl)nitrone. | | | |
| 166 | Saudi Medical Journal | Yasseen AA | 2002 | 0 | http://www.ncbi.nlm.nih.gov/pubmed/?term=Total+cellular+ribose+nucleic+acid+analysis+of+multi-drug+resistant+cancer+cells. | | | |
| 170 | Journal of oral pathology and medicine | Abdullah BH | 2002 | 0 | http://www.ncbi.nlm.nih.gov/pubmed/11896823 | | | |
| 238 | Scandinavian Journal of Urology and Nephrology | Kehinde EO | 2002 | 0 | http://informahealthcare.com.ezproxy.aub.edu.lb/doi/pdf/10.1080/003655902320131938 | | | |
| 297 | International journal of gynaecology and obstetrics | Al Azemi | 2002 | 0 | http://www.ncbi.nlm.nih.gov/pubmed/12399095 | | | |
| 299 | Medical Principles and Practice | Ali Y | 2002 | 0 | http://www.ncbi.nlm.nih.gov/pubmed/12138299 | | | |
| 83 | Medical Principles and Practice | Rashed W | 2002 | 0 | http://www.ncbi.nlm.nih.gov/pubmed/?term=atient+characteristics+and+practice+patterns+in+the+treatment+of+acute+++++++myocardial+infa7ion+in+Kuwait%3A+a+pilot+study | | | |
| 84 | Medical Principles and Practice | Al-Attar AT | 2002 | 0 | http://www.ncbi.nlm.nih.gov/pubmed/12424409 | | | |
| 85 | Indian Heart Journal | Suresh CG | 2002 | 0 | http://www.ncbi.nlm.nih.gov/pubmed/?term=Racial+variation+in+risk+factors+and+occurrence+of+acute+myocardial+infa7ion%3A+++++++comparison+between+Arab+and+South+Asian+men+in+Kuwait. | | | |
| 86 | International Journal of Obesity and Related Metabolic Disorders | Olusi SO | 2002 | 0 | http://www.ncbi.nlm.nih.gov/pubmed/?term=Obesity+is+an+independent+risk+factor+for+plasma+lipid+peroxidation+and+depletion+++++++of+erythrocyte+cytoprotectic+enzymes+in+humans | | | |
| 101 | Acta Diabetologica | Abdella NA | 2002 | 0 | http://www.ncbi.nlm.nih.gov/pubmed/12486492 | | | |
| 105 | Diabetes Research and Clinical Practice | Abdul-Rasoul M | 2002 | 0 | http://www.ncbi.nlm.nih.gov/pubmed/?term=Incidence+and+seasonal+variation+of+Type+1+diabetes+in+children+in+Farwania+area%2C+Kuwait+(1995-1999) | | | |
| 109 | Clinica Chimica Acta | Akanji AO | 2002 | 0 | http://www.ncbi.nlm.nih.gov/pubmed/11814472 | | | |
| 200 | BMC Endocrine Disorders | Parrapil A | 2002 | 0 | http://www.ncbi.nlm.nih.gov/pubmed/?term=Diagnostic+criteria+for+diabetes+revisited%3A+making+use+of+combined+criteria | | | |
| 206 | Diabetic Medicine | Shaltout AA | 2002 | 0 | http://onlinelibrary.wiley.com/doi/10.1046/j.1464-5491.2002.00703.x/pdf | | | |
| 208 | Angiology | Sheikh M | 2002 | 0 | http://www.ncbi.nlm.nih.gov/pubmed/11865839 | | | |
| 211 | Medical Principles and Practice | Thomas CS | 2002 | 0 | http://www.ncbi.nlm.nih.gov.ezproxy.aub.edu.lb/pubmed/12444312?report=abstract | | | |
| 235 | Australasian Radiology | Hanna RM | 2002 | 0 | http://onlinelibrary.wiley.com.ezproxy.aub.edu.lb/doi/10.1046/j.1440-1673.2002.01054.x/full | | | |
| 255 | International journal of Cancer | Memon A | 2002 | 0 | http://www.ncbi.nlm.nih.gov.ezproxy.aub.edu.lb/pubmed/11774247 | | | |
| 256 | British journal of cancer | Memon A | 2002 | 0 | http://www.ncbi.nlm.nih.gov.ezproxy.aub.edu.lb/pubmed/?term=We+conducted+a+population-based+study+of+313+case-control+pairs+in+Kuwait+to+examine+the+aetiology+of+thyroid+cancer | | | |
| 313 | Medical Principles and Practice | Al Shemmari SH | 2002 | 0 | http://www.ncbi.nlm.nih.gov/pubmed/12138297 | | | |
| 411 | Medical Principles and Practice | Al-Harbi K | 2002 | 0 | http://www.ncbi.nlm.nih.gov/pubmed/12116689 | | | |
| 595 | The Journal of Asthma | Hijazi Z | 2002 | 0 | http://www.ncbi.nlm.nih.gov.ezproxy.aub.edu.lb/pubmed/?term=Characteristics+of+asthmatic+children+in+Kuwait. | | | |
| 596 | Medical Principles and Practice | Hijazi Z | 2002 | 0 | http://www.ncbi.nlm.nih.gov.ezproxy.aub.edu.lb/pubmed/?term=Hospital+management+of+children+with+acute+asthma+exacerbations+in+Kuwait%3A+adherence+to+international+guidelines. | | | |
| 607 | European Journal of Clinical Nutrition | Jackson RT | 2002 | 0 | http://www.ncbi.nlm.nih.gov.ezproxy.aub.edu.lb/pubmed/12122545 | | | |
| 639 | Environmental Health Perspectives | Lange JL | 2002 | 0 | http://www.ncbi.nlm.nih.gov.ezproxy.aub.edu.lb/pubmed/?term=Exposures+to+the+Kuwait+oil+fires+and+their+association+with+asthma+and+bronchitis+among+gulf+war+veterans. | | | |
| 642 | oncology reports | Luqmani YA | 2002 | 0 | http://www.ncbi.nlm.nih.gov.ezproxy.aub.edu.lb/pubmed/11836619 | | | |
| 703 | Clinical rheumatology | Refai TM | 2002 | 0 | http://www.ncbi.nlm.nih.gov/pubmed/12447627 | | | |
| 733 | Medical Principles and Practice | Thomas CS | 2002 | 0 | http://www.ncbi.nlm.nih.gov.ezproxy.aub.edu.lb/pubmed/?term=Angiographic+comparison+of+coronary+artery+disease+in+Arab+women+with+and+without+type+II+diabetes+mellitus | | | |
| 87 | Current medical Research and Opinion | Sharma JN | 2002 | 0 | http://www.ncbi.nlm.nih.gov/pubmed/11999140 | | | |
| 100 | Medical Principles and Practice | Abdella NA | 2002 | 0 | http://www.ncbi.nlm.nih.gov/pubmed/12444313 | | | |
| 108 | Medical Principles and Practice | Akanji AO | 2002 | 0 | http://www.ncbi.nlm.nih.gov/pubmed/12444310 | | | |
| 237 | Medical Principles and Practice | Joseph BK | 2002 | 0 | http://www.ncbi.nlm.nih.gov.ezproxy.aub.edu.lb/pubmed/?term=-+Researchers+in+oral+cancer+agree+that+the+early+diagnosis+of+oral+carcinoma+greatly+increases+the+probability+of+cure+with+minimum+impairment+and+deformity. | | | |
| 890 | Current Medicinal Chemistry | Novotny L | 2002 | 0 | http://www.ncbi.nlm.nih.gov/pubmed/11966441 | | | |
| 226 | Autonomic and Autacoid Pharmacology | Yousif MH | 2002 | 0 | http://www.ncbi.nlm.nih.gov/pubmed/?term=Endothelium-dependent+relaxation+in+isolated+renal+arteries+of+diabetic+rabbits | | | |
| 249 | Oncology reports | Luqmani YA | 2002 | 0 | http://www.ncbi.nlm.nih.gov.ezproxy.aub.edu.lb/pubmed/?term=Cytosol+of+primary+breast+cancers+from+217+women+of+predominantly+Arab+ethnicity+were+assayed+for+uPA%2C+tPA%2C+PAI-1+and+a+subset+for+ER%2C+PR+and+pS2 | | | |
| 300 | European Journal of Cancer Prevention | Al Khalaf M | 2002 | 0 | http://www.ncbi.nlm.nih.gov/pubmed/12394246 | | | |
| 335 | Medical Principles and Practice | Junaid TA | 2002 | 0 | http://www.ncbi.nlm.nih.gov/pubmed/12444304 | | | |
| 647 | Transplantation proceedings | Mahmoud FF | 2002 | 0 | http://www.ncbi.nlm.nih.gov.ezproxy.aub.edu.lb/pubmed/12431672 | | | |
| 181 | Diabetes Research and Clinical Practice | Mojiminiyi OA | 2002 | 0 | http://www.ncbi.nlm.nih.gov/pubmed/12161055 | | | |
| 229 | East African Medical Journal | Elshebiny YH | 2002 | 0 | http://www.ncbi.nlm.nih.gov.ezproxy.aub.edu.lb/pubmed/?term=-+A+case+of+leiomyoma+of+urinary+bladder%2C+a+rare+benign+tumour%2C+is+presented.+The+patient+was+a+42+year+old+female+who+presented+with+dysuria+and+frequency+of+micturition | | | |
| 287 | Journal of Clinical Laboratory Analysis | Abiaka C | 2002 | 0 | http://www.ncbi.nlm.nih.gov/pubmed/12112388 | | | |
| 265 | international Journal of Clinical Practices | Qassem AA | 2002 | 0 | http://www.ncbi.nlm.nih.gov/pubmed/?term=To+analyse+preferences+for+disclosure+of+cancer+diagnosis+and+prognosis+among+physicians+practising+in+Kuwait | | | |
| 288 | Journal of Clinical Laboratory Analysis | Abiaka C | 2002 | 0 | http://www.ncbi.nlm.nih.gov/pubmed/11793433 | | | |
| 346 | Asia Pacific Journal of Clinical Nutrition | Abiaka C | 2002 | 0 | http://www.ncbi.nlm.nih.gov/pubmed/11890634 | | | |
| 508 | International Journal of Biological Markers | Behbehani AI | 2002 | 0 | http://www.ncbi.nlm.nih.gov/pubmed/11936590 | | | |
| 242 | Pathology Oncology Research | KhoursheedM. | 2002 | 0 | http://por.hu/2002/8/3/0170/0170a.pdf | | | |
| 752 | Vascular Pharmacology | Yousif MH | 2002 | 0 | http://www.ncbi.nlm.nih.gov/pubmed/14567066 | | | |
| 143 | Journal of pain and symptom management | Daher M | 2002 |  | Not Found | |  |  |
| 105 | J Med Liban | Bulbul M A | 2002 | 0 | http://www.ncbi.nlm.nih.gov/pubmed/12841318 | | | |
| 127 | Hemoglobin | Chehal A | 2002 | 0 | http://www.ncbi.nlm.nih.gov/pubmed/?term=Beta-thalassemia+intermedia+and+non-Hodgkin's+lymphoma | | | |
| 128 | European journal of gynaecological oncology | Chehal A | 2002 | 0 | http://www.ncbi.nlm.nih.gov/pubmed/12556107 | | | |
| 245 | Journal of pediatric orthopaedics | Ghanem I B | 2002 | 0 | http://www.ncbi.nlm.nih.gov/pubmed/?term=Rib+fixation+for+humeral+lengthening+following+interscapulothoracic+resection+for+malignant+tumor | | | |
| 971 | BMJ | Dakik HA | 2002 | 0 | http://www.ncbi.nlm.nih.gov/pubmed/11777802 | | | |
| 181 | J Med Liban | El Saghir N S | 2002 | 0 | http://www.ncbi.nlm.nih.gov/pubmed/?term=Age+distribution+of+breast+cancer+in+Lebanon%3A+increased+percentages+and+age+adjusted+incidence+rates+of+younger-aged+groups+at+presentation | | | |
| 259 | J Med Liban | Ghoussoub K | 2002 | 0 | http://www.ncbi.nlm.nih.gov/pubmed/?term=Two-year+follow-up+study+of+postoperative+rehabilitation%2C+return+to+work%2C+and+athletic+activities+of+111+Lebanese+patients+after+coronary+bypass%5D | | | |
| 667 | European journal of gynaecological oncology | Mroueh AM | 2002 | 0 | http://www.ncbi.nlm.nih.gov.ezproxy.aub.edu.lb/pubmed/12440818 | | | |
| 53 | J Med Liban | Aoun E | 2002 | 0 | http://www.ncbi.nlm.nih.gov/pubmed/12841311 | | | |
| 230 | Current cancer drug targets | Gali-Muhtasib H | 2002 | 0 | http://www.ncbi.nlm.nih.gov/pubmed/?term=Modulating+cell+cycle%3A+current+applications+and+prospects+for+future+drug+development | | | |
| 307 | International journal of cardiology | Jabbour S | 2002 | 0 | http://www.ncbi.nlm.nih.gov/pubmed/?term=Cardiovascular+disease+and+the+global+tobacco+epidemic%3A+a+wake-up+call+for+cardiologists | | | |
| 1021 | Saudi journal of kidney diseases and transplantation | Mallat SG | 2002 | 0 | http://www.ncbi.nlm.nih.gov/pubmed/18209429 | | | |
| 158 | Molecular Carcinogenesis | Diab-Assef M | 2002 | 0 | http://www.ncbi.nlm.nih.gov/pubmed/11933073 | | | |
| 196 | Blood | El-Sabban M E | 2002 | 0 | http://www.ncbi.nlm.nih.gov/pubmed/?term=Human+T-cell+lymphotropic+virus+type+1-transformed+cells+induce+angiogenesis+and+establish+functional+gap+junctions+with+endothelial+cells | | | |
| 311 | J Med Liban | Jamaleddine G | 2002 | 1 | http://www.ncbi.nlm.nih.gov/pubmed/?term=Cancer+patients+and+critical+care+medicine+jamaleddine+2002 | | | |
| 732 | Molecular Carcinogenesis | Maalouf S | 2002 | 0 | http://www.ncbi.nlm.nih.gov/pubmed/?term=Protective+effect+of+vitamin+E+on+ultraviolet+B+light-induced+damage+in+keratinocytes. | | | |
| 710 | Diagn Microbiol Infect Dis | Tamim H | 2002 | 0 | http://www.ncbi.nlm.nih.gov/pubmed/12151187 | | | |
| 252 | J Med Liban | Ghosn M | 2002 | 0 | http://www.ncbi.nlm.nih.gov/pubmed/?term=Outcome+of+cancer+patients+admitted+to+the+intensive+care+unit+(ICU)+Ghosn+2002 | | | |
| 234 | J Med Liban | Gali-Muhtasib H U | 2002 | 0 | http://www.ncbi.nlm.nih.gov/pubmed/15298478 | | | |
| 1063 | Archives de pédiatrie | Radouane B | 2002 |  | http://www.ncbi.nlm.nih.gov/pubmed/12108322 | | | |
| 65 | Rev Chir Orthop Reparatrice Appar Mot | Belaabidia B | 2002 | 1 | http://www.ncbi.nlm.nih.gov/pubmed/12399719 | | | |
| 66 | J Gynecol Obstet Biol Reprod (Paris) | Belaabidia B | 2002 | 1 | http://www.ncbi.nlm.nih.gov/pubmed/11976575 | | | |
| 82 | Annales d'Urologie | Benchekroun A | 2002 | 0 | http://www.ncbi.nlm.nih.gov/pubmed/11859574 | | | |
| 1030 | Med Trop (Mars) | Hachi H | 2002 | 1 | http://www.ncbi.nlm.nih.gov/pubmed/12616948 | | | |
| 1033 | Annales de Pathologie | Cherradi N | 2002 | 1 | http://www.ncbi.nlm.nih.gov/pubmed/12594389 | | | |
| 1034 | Bulletin de la Société belge d'ophtalmologie | Charif CM | 2002 | 1 | http://www.ncbi.nlm.nih.gov/pubmed/12564314 | | | |
| 1038 | Progrès en urologie | Bakkali H | 2002 | 1 | http://www.ncbi.nlm.nih.gov/pubmed/12545639 | | | |
| 1039 | Cancer radiothérapie | Tayeb K | 2002 | 1 | http://www.ncbi.nlm.nih.gov/pubmed/12504775 | | | |
| 1040 | Cancer radiothérapie | SaÃ¢di I | 2002 | 1 | http://www.ncbi.nlm.nih.gov/pubmed/12504774 | | | |
| 1041 | Cancer radiothérapie | Bakkali H | 2002 | 1 | http://www.ncbi.nlm.nih.gov/pubmed/12504773 | | | |
| 1043 | Revue de pneumologie clinique | Smahi M | 2002 | 1 | http://www.ncbi.nlm.nih.gov/pubmed/12486800 | | | |
| 1044 | Annales d'Urologie | Rabii R | 2002 | 1 | http://www.ncbi.nlm.nih.gov/pubmed/12481625 | | | |
| 1045 | Annales d'Urologie | Benchekroun A | 2002 | 1 | http://www.ncbi.nlm.nih.gov/pubmed/12481622 | | | |
| 1046 | Gynécologie, obstétrique & fertilité | Zrara I | 2002 | 1 | http://www.ncbi.nlm.nih.gov/pubmed/12478984 | | | |
| 1047 | Progrès en urologie | Ameur A | 2002 | 1 | http://www.ncbi.nlm.nih.gov/pubmed/12463137 | | | |
| 1048 | Gynécologie, obstétrique & fertilité | Hachi H | 2002 | 1 | http://www.ncbi.nlm.nih.gov/pubmed/12448366 | | | |
| 1049 | Annales de Biologie Clinique | Ouzzif Z | 2002 | 1 | http://www.ncbi.nlm.nih.gov/pubmed/12446237 | | | |
| 1051 | Cancer radiothérapie | Hadadi K | 2002 | 1 | http://www.ncbi.nlm.nih.gov/pubmed/12412365 | | | |
| 1052 | Cancer radiothérapie | Sifat H | 2002 | 1 | http://www.ncbi.nlm.nih.gov/pubmed/12412362 | | | |
| 1053 | Presse médicale | Faraj Z | 2002 | 1 | http://www.ncbi.nlm.nih.gov/pubmed/12378975 | | | |
| 1056 | Gynécologie, obstétrique & fertilité | Benchakroun N | 2002 | 1 | http://www.ncbi.nlm.nih.gov/pubmed/12199042 | | | |
| 1057 | Annales d'Endocrinologie | Ibnou Soufyane N | 2002 | 1 | http://www.ncbi.nlm.nih.gov/pubmed/12193880 | | | |
| 1058 | Progrès en urologie | Benchekroun A | 2002 | 1 | http://www.ncbi.nlm.nih.gov/pubmed/12189758 | | | |
| 1059 | Progrès en urologie | Benchekroun A | 2002 | 1 | http://www.ncbi.nlm.nih.gov/pubmed/12189755 | | | |
| 1060 | Annales d'Urologie | Benchekroun A | 2002 | 1 | http://www.ncbi.nlm.nih.gov/pubmed/12162196 | | | |
| 1061 | Annales d'Urologie | Ghadouane M | 2002 | 1 | http://www.ncbi.nlm.nih.gov/pubmed/12162187 | | | |
| 1062 | Acta Orthopaedica Belgica | Raissouni Z | 2002 | 1 | http://www.ncbi.nlm.nih.gov/pubmed/12152380 | | | |
| 1064 | Journal de gynécologie, obstétrique et biologie de la reproduction | BenÃ¢aboud I | 2002 | 1 | http://www.ncbi.nlm.nih.gov/pubmed/12058141 | | | |
| 1065 | Annales d'Urologie | Benchekroun A | 2002 | 1 | http://www.ncbi.nlm.nih.gov/pubmed/12056096 | | | |
| 1066 | Annales d'Urologie | Ameur A | 2002 | 1 | http://www.ncbi.nlm.nih.gov/pubmed/12056086 | | | |
| 1068 | Revue de stomatologie et de chirurgie maxillo-faciale | Mahtar M | 2002 | 1 | http://www.ncbi.nlm.nih.gov/pubmed/11997740 | | | |
| 1069 | Neurochirurgie | Fadli M | 2002 | 1 | http://www.ncbi.nlm.nih.gov/pubmed/11972150 | | | |
| 1070 | Annales d'Urologie | Ameur A | 2002 | 1 | http://www.ncbi.nlm.nih.gov/pubmed/11969052 | | | |
| 1075 | Archives des maladies du coeur et des vaisseaux | Ztot S | 2002 | 1 | http://www.ncbi.nlm.nih.gov/pubmed/11901891 | | | |
| 1076 | Cancer radiothérapie | el Omari-Alaoui H | 2002 | 1 | http://www.ncbi.nlm.nih.gov/pubmed/11899679 | | | |
| 1077 | Revue médicale de Liège | el Absi M | 2002 | 1 | http://www.ncbi.nlm.nih.gov/pubmed/11899491 | | | |
| 1083 | Annales d'Urologie | Zannoud M | 2002 | 1 | http://www.ncbi.nlm.nih.gov/pubmed/11859580 | | | |
| 1084 | Annales de Chirugie | Banani A | 2002 | 1 | http://www.ncbi.nlm.nih.gov/pubmed/11833308 | | | |
| 116 | Revue des maladies respiratoires | Yazidi A A | 2002 | 1 | http://www.ncbi.nlm.nih.gov/pubmed/12417860 | | | |
| 179 | Annales d'Endocrinologie | Diouri A | 2002 | 1 | http://www.ncbi.nlm.nih.gov/pubmed/12527849 | | | |
| 223 | Cancer Radiothérapie | El Omari-Alaoui H | 2002 | 0 | http://www.ncbi.nlm.nih.gov/pubmed/12504771 | | | |
| 274 | Annales de dermatologie et de vénéréologie | Hali F | 2002 | 0 | http://www.ncbi.nlm.nih.gov/pubmed/12124511 | | | |
| 360 | Annales de biologie clinique | Laraqui A | 2002 | 1 | http://www.ncbi.nlm.nih.gov/pubmed/12368140 | | | |
| 1067 | Gynécologie, obstétrique & fertilité | Sahraoui S | 2002 | 1 | http://www.ncbi.nlm.nih.gov/pubmed/12043504 | | | |
| 1042 | Annales Françaises d'Anesthésie et de Réanimation | Zeggwagh AA | 2002 | 1 | http://www.ncbi.nlm.nih.gov/pubmed/12494803 | | | |
| 97 | Public health nutrition | Benjelloun S | 2002 | 0 | http://www.ncbi.nlm.nih.gov/pubmed/12027276 | | | |
| 175 | Journal of Biomechanics | Derouich M | 2002 | 0 | http://www.ncbi.nlm.nih.gov/pubmed/12052393 | | | |
| 1071 | Annales de Biologie Clinique | Mikdame M | 2002 | 1 | http://www.ncbi.nlm.nih.gov/pubmed/11937438 | | | |
| 212 | Journal of ethnopharmacology | El Hilay J | 2002 | 0 | http://www.ncbi.nlm.nih.gov/pubmed/12007699 | | | |
| 279 | Clinical genetics | Harich N | 2002 | 0 | http://www.ncbi.nlm.nih.gov/pubmed/12220441 | | | |
| 328 | Journal of Ethnopharmacology | Jouad H | 2002 | 0 | http://www.ncbi.nlm.nih.gov/pubmed/12127236 | | | |
| 348 | Annales de biologie clinique | Lahlali-Kacemi N | 2002 | 1 | http://www.ncbi.nlm.nih.gov/pubmed/11830396 | | | |
| 712 | Journal of Ethnopharmacology | El Hilaly J | 2002 | 0 | http://www.ncbi.nlm.nih.gov/pubmed/12007699 | | | |
| 1050 | Thérapie | Benajiba N | 2002 | 1 | http://www.ncbi.nlm.nih.gov/pubmed/12422535 | | | |
| 1054 | Phytotherapy research | Ziyyat A | 2002 | 1 | http://www.ncbi.nlm.nih.gov/pubmed/12237817 | | | |
| 1055 | Phytotherapy research | Legssyer A | 2002 | 0 | http://www.ncbi.nlm.nih.gov/pubmed/12237804 | | | |
| 100 | Med Trop (Mars) | Benkiran L | 2002 | 1 | http://www.ncbi.nlm.nih.gov/pubmed/12038184 | | | |
| 191 | Journal of Ethnopharmacology | Eddouks M | 2002 | 0 | http://www.ncbi.nlm.nih.gov/pubmed/12241983 | | | |
| 464 | Cancer Causes and Control | Sasco AJ | 2002 | 0 | http://www.ncbi.nlm.nih.gov/pubmed/12296508 | | | |
| 66 | Eur Respir J | El-Sharif N | 2002 | 0 | http://www.ncbi.nlm.nih.gov/pubmed/12108852 | | | |
| 120 | Addiction (Abigdon, England) | WHO ASSIST Working Group | 2002 | 0 | http://www.ncbi.nlm.nih.gov.ezproxy.aub.edu.lb/pubmed/12199834 | | | |
| 148 | The Science of the total environment | Safi JM | 2002 | 0 | http://www.ncbi.nlm.nih.gov.ezproxy.aub.edu.lb/pubmed/11846176 | | | |
| 129 | Annals of oncology | Darwish H | 2002 | 0 | http://www.ncbi.nlm.nih.gov.ezproxy.aub.edu.lb/pubmed/12196377 | | | |
| 116 | Journal of stroke and cerebrovascular diseases | Sokrab T E | 2002 | 0 | http://www.ncbi.nlm.nih.gov/pubmed/?term=Acute+stroke+type%2C+risk+factors%2C+and+early+outcome+in+a+developing+country%3A+a+view+from+Sudan+using+a+hospital-based+sample | | | |
| 6 | Metabolism: clinical and experimental | Abdelgadir M | 2002 | 0 | http://www.ncbi.nlm.nih.gov/pubmed/?term=Reduced+leptin+concentrations+in+subjects+with+type+2+diabetes+mellitus+in+Sudan | | | |
| 91 | Breast Cancer Research and Treatment | Masri M A | 2002 | 0 | http://www.ncbi.nlm.nih.gov/pubmed/?term=Minor+role+for+BRCA2+(exon11)+and+p53+(exon+5-9)+among+Sudanese+breast+cancer+patients | | | |
| 14 | Saudi medical journal | Ahmed A M | 2002 | 0 | https://www.ncbi.nlm.nih.gov.ezproxy.aub.edu.lb/pubmed/?term=History+of+diabetes+mellitus+Ahmed%2C+A.+M+Saudi+medical+journal | | | |
| 97 | Medical principles and practice | Musaiger A O | 2002 | 0 | http://www.ncbi.nlm.nih.gov/pubmed/12444306 | | | |
| 127 | Breast Cancer Research and Treatment | Masri MA | 2002 | 0 | http://www.ncbi.nlm.nih.gov/pubmed/22552770 | | | |
| 78 | Anticancer research | Ibrahim S O | 2002 | 0 | http://www.ncbi.nlm.nih.gov/pubmed/12168821 | | | |
| 186 | Obstet Gynecol | Rajab KE | 2003 | 0 | http://www.ncbi.nlm.nih.gov/pubmed/14607041 | | | |
| 70 | Journal of Evaluation in Clinical Practice | Sequeira RP | 2003 | 0 | http://www.ncbi.nlm.nih.gov/pubmed/14758963 | | | |
| 73 | Annals of Pharmacotherapy | Al Khaja KA | 2003 | 0 | http://www.ncbi.nlm.nih.gov/pubmed/14519051 | | | |
| 74 | Saudi Medical Journal | Alnasir FA | 2003 | 0 | http://www.ncbi.nlm.nih.gov/pubmed/12973475 | | | |
| 75 | Journal for Scientific Research Medical Sciences | Rajab KE | 2003 | 0 | http://www.ncbi.nlm.nih.gov/pubmed/24019728 | | | |
| 76 | Annals of Human Biology | Al-Sendi AM | 2003 | 0 | http://www.ncbi.nlm.nih.gov/pubmed/12881137 | | | |
| 77 | Pharmacoepidemiology Drug Safety | al Khaja KA | 2003 | 0 | http://www.ncbi.nlm.nih.gov/pubmed/12733473 | | | |
| 78 | European Journal of Clinical Nutrition | Al-Sendi AM | 2003 | 0 | http://www.ncbi.nlm.nih.gov/pubmed/12627185 | | | |
| 196 | Nutrition and Health | Musaiger AO | 2003 | 0 | http://www.ncbi.nlm.nih.gov/pubmed/14653506 | | | |
| 59 | Eastern Mediterranean health journal | Al-Haddad N | 2003 | 0 | http://www.ncbi.nlm.nih.gov/pubmed/15562736 | | | |
| 72 | British Journal of Nutrition | Al-Sendi AM | 2003 | 0 | http://www.ncbi.nlm.nih.gov/pubmed/14552329 | | | |
| 55 | Eastern Mediterranean health journal | Al-Hilli F | 2003 | 0 | http://www.ncbi.nlm.nih.gov/pubmed/15751930 | | | |
| 163 | Saudi Medical Journal | Al-Waiz M | 2003 |  | http://www.ncbi.nlm.nih.gov/pubmed/?term=An+upsurge+of+new+cases+of+Kaposi's+sarcoma+in+Iraqi+patients. | | | |
| 136 | Annals of Saudi Medicine | Saeed AK | 2003 | 0 | http://www.ncbi.nlm.nih.gov/pubmed/?term=Type+2+diabetes+and+its+association+with+hypertension+and+depression+in+an+Iraqi+population. | | | |
| 162 | Saudi Medical Journal | Mula-Abed WA | 2003 | 0 | http://www.ncbi.nlm.nih.gov/pubmed/?term=Performance+indicators+and+validity+of+serum+fructosamine+assay+as+a+diagnostic+test+in+a+screening+program+for+diabetes+mellitus. | | | |
| 585 | East African Medical Journal | Hanna RM | 2003 | 0 | http://www.ncbi.nlm.nih.gov.ezproxy.aub.edu.lb/pubmed/?term=Giant+fat+containing+breast+masses%3A+report+of+six+cases. | | | |
| 590 | Indian Journal of Otolaryngology and Head and Neck Surgery | Hazarika P | 2003 | 0 | http://www.ncbi.nlm.nih.gov.ezproxy.aub.edu.lb/pubmed/?term=Solitary+malignant+schwanoma+of+para+pharyngeal+space-a+case+report+and+review+of+literature. | | | |
| 76 | Medical Principles and Practice | Al-Shammri S | 2003 | 0 | http://www.ncbi.nlm.nih.gov/pubmed/?term=Risk+factors%2C+subtypes+and+outcome+of+ischaemic+stroke+in+Kuwait--a+++++++hospital-based+study | | | |
| 78 | Journal of Biosocial Sciences | Al-Kandari YY | 2003 | 0 | http://www.ncbi.nlm.nih.gov/pubmed/?term=Religiosity+and+its+relation+to+blood+pressure+among+selected+Kuwaitis | | | |
| 80 | Angiology | Thomas CS | 2003 | 0 | http://www.ncbi.nlm.nih.gov/pubmed/?term=Disease+of+proximal+part+of+vertebral+artery+in+patients+with+coronary+artery+++++++disease | | | |
| 82 | Angiology | Thomas CS | 2003 | 0 | http://www.ncbi.nlm.nih.gov/pubmed/?term=Extent+and+severity+of+atherosclerotic+vascular+disease+in+patients+undergoing+++++++coronary+angiography--the+Kuwait+Vascular+Study | | | |
| 115 | Journal of Diabetes and its Complications | Al Sabti K | 2003 | 0 | http://www.ncbi.nlm.nih.gov.ezproxy.aub.edu.lb/pubmed/?term=Efficacy+and+reliability+of+fundus+digital+camera+as+a+screening+tool+for+diabetic+retinopathy+in+Kuwait | | | |
| 179 | Journal of Diabetes and its Complications | Mojiminiyi OA | 2003 | 0 | http://www.ncbi.nlm.nih.gov/pubmed/?term=Evaluation+of+cystatin+C+and+beta-2+microglobulin+as+markers+of+renal+function+in+patients+with+type+2+diabetes+mellitus | | | |
| 199 | Annals of Saudi Medicine | Olusi SO | 2003 | 0 | http://www.ncbi.nlm.nih.gov/pubmed/?term=Baseline+population+survey+data+on+the+prevalence+of+risk+factors+for+coronary+artery+disease+among+Kuwaitis+aged+15+years+and+older | | | |
| 233 | Medical Principles and Practice | Gupta R | 2003 | 0 | http://www.karger.com.ezproxy.aub.edu.lb/Article/Pdf/70756 | | | |
| 250 | Pathology Oncology Research | Makar RR | 2003 | 0 | http://por.hu/2003/9/3/0159/0159a.pdf | | | |
| 314 | Clinical Lymphoma | Al Shemmari SH | 2003 | 0 | http://www.ncbi.nlm.nih.gov/pubmed/14556681 | | | |
| 391 | Occupational Medicine | Al-Asi T | 2003 | 0 | http://www.ncbi.nlm.nih.gov/pubmed/14581639 | | | |
| 422 | Nutrition and Health | Al-Isa AN | 2003 | 0 | http://www.ncbi.nlm.nih.gov/pubmed/14703151 | | | |
| 683 | Biological Trace Element Research | Olusi S | 2003 | 0 | http://www.ncbi.nlm.nih.gov.ezproxy.aub.edu.lb/pubmed/12719608 | | | |
| 684 | Hormone Research in Paediatrics | Olusi S | 2003 | 0 | http://www.ncbi.nlm.nih.gov.ezproxy.aub.edu.lb/pubmed/?term=Relations+of+serum+interleukin+18+levels+to+serum+lipid+and+glucose+concentrations+in+an+apparently+healthy+adult+population. | | | |
| 698 | American Journal of Human Biology | Ramadan J | 2003 | 0 | http://onlinelibrary.wiley.com/doi/10.1002/ajhb.10190/pdf | | | |
| 899 | J Obstet Gynaecol | Mahmoud F | 2003 | 0 | http://www.ncbi.nlm.nih.gov/pubmed/12623476 | | | |
| 75 | Journal of Clinical Pharamcology | Sharma JN | 2003 | 0 | http://www.ncbi.nlm.nih.gov/pubmed/?term=oes+the+kinin+system+mediate+in+cardiovascular+abnormalities%3F+An+overview | | | |
| 275 | Current Cancer Drug Targets | Thomson M | 2003 | 0 | http://www.ncbi.nlm.nih.gov/pubmed/?term=Garlic+%5BAllium+sativum%5D+is+among+the+oldest+of+all+cultivated+plants.+It+has+been+used+as+a+medicinal+agent+for+thousands+of+years | | | |
| 719 | IDrugs | Sharma JN | 2003 | 0 | http://www.ncbi.nlm.nih.gov.ezproxy.aub.edu.lb/pubmed/12811681 | | | |
| 736 | Current Cancer Drug Targets | Thomson M | 2003 | 0 | http://www.ncbi.nlm.nih.gov.ezproxy.aub.edu.lb/pubmed/?term=Garlic+%5BAllium+sativum%5D%3A+a+review+of+its+potential+use+as+an+anti-cancer+agent | | | |
| 74 | Brain Research | Al-Sarraf H | 2003 | 0 | http://www.ncbi.nlm.nih.gov/pubmed/14642643 | | | |
| 77 | Prostaglandins, Leukotrienes, and Essential Fatty Acids | Al-Qattan KK | 2003 | 0 | http://www.ncbi.nlm.nih.gov/pubmed/?term=Mechanism+of+garlic+(Allium+sativum)+induced+reduction+of+hypertension+in+2K-1C+++++++rats%3A+a+possible+mediation+of+Na%2FH+exchanger+isoform-1 | | | |
| 79 | Brain Research | Al-Sarraf H | 2003 | 0 | http://www.ncbi.nlm.nih.gov/pubmed/12763606 | | | |
| 81 | International Immunopharmacology | Sharma JN | 2003 | 0 | http://www.ncbi.nlm.nih.gov/pubmed/?term=Evaluation+of+tissue+kallikrein+activity+on+survival+time+after+acute+coronary+++++++artery+ligation+in+hypertensive+rats | | | |
| 214 | Pharmacological Research | Yousif MH | 2003 | 0 | http://www.ncbi.nlm.nih.gov/pubmed/?term=Diabetes+differentially+modulated+receptor-+and+non-receptor-mediated+relaxation+in+rat+renal+artery | | | |
| 221 | Autonomic and Autacoid Pharmacology | Yousif MH | 2003 | 0 | http://www.ncbi.nlm.nih.gov/pubmed/?term=Inhibition+of+calcium%2Fcalmodulin-dependent+protein+kinase+II+normalizes+diabetes-induced+abnormal+vascular+reactivity+in+the+rat+perfused+mesenteric+vascular+bed | | | |
| 248 | International journal of molecular medicine | Luqmani YA | 2003 | 0 | http://www.ncbi.nlm.nih.gov.ezproxy.aub.edu.lb/pubmed/?term=Microsatellite+instability+(MSI)+and+loss+of+heterozygosity+(LOH)+was+investigated+in+paired+tumour+and+normal+tissue+DNA+from+108+predominantly+premenopausal+breast+cancer+patients+(under+age+45+years+at+presentation)+for+25+simple+repeat+loci+interspersed+across+11+chromosomes.+MSI+was+observed+at+a+single+locus+in+69+(64%25)+patients%3 | | | |
| 281 | Carcinogenesis | El Mowafy AM | 2003 | 0 | http://carcin.oxfordjournals.org/content/24/5/869.long | | | |
| 282 | Tumour Biology | Abaza MS | 2003 | 0 | http://www.ncbi.nlm.nih.gov/pubmed/15001837 | | | |
| 301 | The Journal of Endocrinology | Al Khalaf M | 2003 | 0 | http://www.ncbi.nlm.nih.gov/pubmed/14529565 | | | |
| 304 | Archives of Biochemistry and Biophysics | Al Maghrebi M | 2003 | 0 | http://linkedlifedata.com/resource/pubmed/id/12921788 | | | |
| 308 | Molecular Pathology | Al Mulla F | 2003 | 0 | http://www.ncbi.nlm.nih.gov/pubmed/12890742 | | | |
| 524 | International Journal of Tuberculosis and Lung Disease | Chan-Yeung M | 2003 | 0 | http://www.ncbi.nlm.nih.gov/pubmed/12757038 | | | |
| 640 | Anticancer Research | Lincoln DT | 2003 | 0 | http://www.ncbi.nlm.nih.gov.ezproxy.aub.edu.lb/pubmed/?term=The+thioredoxin-thioredoxin+reductase+system%3A+over-expression+in+human+cancer. | | | |
| 728 | Family Medicine | Sorkhou I | 2003 | 0 | http://www.ncbi.nlm.nih.gov.ezproxy.aub.edu.lb/pubmed/12861451 | | | |
| 512 | International Journal of Tuberculosis and Lung Disease | Behbehani NA | 2003 | 0 | http://www.ncbi.nlm.nih.gov/pubmed/12757041 | | | |
| 543 | Nutrition | Dashti HM | 2003 | 0 | http://www.ncbi.nlm.nih.gov/pubmed/14559328 | | | |
| 243 | The Surgeon | Khoursheed M | 2003 | 0 | http://www.ncbi.nlm.nih.gov.ezproxy.aub.edu.lb/pubmed/?term=To+investigate+the+expression+of+E-cadherin%2C+a+calcium-dependent+cell-cell+adhesion+molecule+in+colorectal+carcinoma | | | |
| 280 | Medical Principles and Practice | Vembu P | 2003 | 0 | http://www.ncbi.nlm.nih.gov/pubmed/?term=-+OBJECTIVE%3A+To+report+a+case+of+severe+Guillain-Barre+syndrome+in+a+32-year+old+female+patient+diagnosed+with+acute+lymphoblastic+leukaemia+who+was+on+chemotherapy | | | |
| 333 | East African Medical Journal | Elshebiny YH | 2003 | 0 | http://www.ncbi.nlm.nih.gov/pubmed/12635765 | | | |
| 865 | Acta Paediatrica | Alkhalaf M | 2003 | 0 | http://www.ncbi.nlm.nih.gov/pubmed/12839302 | | | |
| 241 | BJU international | Kehinde EO | 2003 | 0 | http://www.ncbi.nlm.nih.gov.ezproxy.aub.edu.lb/pubmed/?term=%3A+To+investigate+the+common+causes+of+total+serum+prostate-specific+antigen+(PSA)+values+of%3E+10+ng%2FmL+in+an+Arab+population%2C+as+in+the+USA+and+Europe+the+risk+of+prostate+cancer+is+considered+high+in+men+with+such+PSA+levels. | | | |
| 345 | Journal of Clinical Laboratory Analysis | Abiaka C | 2003 | 0 | http://www.ncbi.nlm.nih.gov/pubmed/12640629 | | | |
| 667 | Pediatric Hematology-Oncology | Mottl H | 2003 | 0 | http://www.ncbi.nlm.nih.gov/pubmed/?term=High+survival+rate+in+childhood+non-Hodgkin+lymphoma+without+CNS+involvement%3A+results+of+BFM+95+study+in+Kuwait. | | | |
| 263 | Hematology (amsterdam, Netherlands) | Novotny L | 2003 | 0 | http://www.ncbi.nlm.nih.gov/pubmed/12745645 | | | |
| 320 | Environment International | Bem H | 2003 | 0 | http://www.ncbi.nlm.nih.gov/pubmed/14664872 | | | |
| 332 | Carcinogenesis | El Mowafy AM | 2003 | 0 | http://www.ncbi.nlm.nih.gov/pubmed/12771030 | | | |
| 527 | Neoplasma | Cojocel C | 2003 | 0 | http://www.ncbi.nlm.nih.gov/pubmed/12740644 | | | |
| 84 | Chemistry & biology | Ballew N | 2003 |  | Not Found | |  |  |
| 137 | Medical and pediatric oncology | Dabbous I A | 2003 |  | Not Found | |  |  |
| 462 | Molecular immunology | Salamoun W | 2003 |  | Abstract Not Found | |  |  |
| 518 | Southern medical journal | Tohfe M | 2003 |  | Abstract Not Found | |  |  |
| 116 | J Med Liban | Chalhoub-Hachem B R | 2003 | 0 | http://www.ncbi.nlm.nih.gov/pubmed/15298165 | | | |
| 130 | European journal of gynaecological oncology | Chehal A | 2003 | 0 | http://www.ncbi.nlm.nih.gov/pubmed/14658598 | | | |
| 131 | Clinical and laboratory haematology | Chehal A | 2003 | 0 | http://www.ncbi.nlm.nih.gov/pubmed/?term=IgM+myeloma+and+Waldenstrom's+macroglobulinemia%3A+a+distinct+clinical+feature%2C+histology%2C+immunophenotype%2C+and+chromosomal+abnormality | | | |
| 269 | European journal of haematology | Haidar J H | 2003 | 0 | http://www.ncbi.nlm.nih.gov/pubmed/?term=Signet+ring-like+light+chain+myeloma+with+systemic+spread | | | |
| 270 | European journal of haematology | Haidar J H | 2003 | 0 | http://www.ncbi.nlm.nih.gov/pubmed/12694172 | | | |
| 364 | Dermatology online journal | Mourad Y A | 2003 | 0 | http://www.ncbi.nlm.nih.gov/pubmed/?term=Nail+toxicity+related+to+taxanes+mourad+2003 | | | |
| 465 | Diagnostic cytopathology | Salem Shabb N | 2003 | 0 | http://www.ncbi.nlm.nih.gov/pubmed/?term=Clear+cell+sarcoma+(malignant+melanoma+of+soft+parts)%3A+fine-needle+aspiration+cytology+of+a+highly+pigmented+tumor | | | |
| 489 | International journal of gynecological cancer | Shamseddine A | 2003 | 0 | http://www.ncbi.nlm.nih.gov/pubmed/?term=Cure+of+metastatic+uterine+carcinosarcoma+to+lungs%3A+a+case+report | | | |
| 689 | International Journal of Surgical Pathology | Saad AG | 2003 | 0 | http://www.ncbi.nlm.nih.gov.ezproxy.aub.edu.lb/pubmed/?term=Leiomyoma+of+the+urethra%3A+report+of+3+cases+of+a+rare+entity. | | | |
| 39 | Molecular immunology | Akoum R | 2003 | 0 | http://www.ncbi.nlm.nih.gov/pubmed/?term=Results+of+radiation+therapy+for+thymoma+based+on+a+review+of+27+patients | | | |
| 103 | Molecular immunology | Brihi E | 2003 | 0 | http://www.ncbi.nlm.nih.gov/pubmed/12835089 | | | |
| 316 | Medical principles and practice | Kalaajieh W K | 2003 | 0 | http://www.ncbi.nlm.nih.gov/pubmed/?term=Response+to+cisplatin-etoposide+treatment+and+survival+in+patients+with+small-cell+lung+cancer+in+North+Lebanon | | | |
| 386 | Health education & behavior | Nakkash R | 2003 | 0 | http://www.ncbi.nlm.nih.gov/pubmed/?term=The+development+of+a+feasible+community-specific+cardiovascular+disease+prevention+program%3A+triangulation+of+methods+and+sources | | | |
| 460 | The European respiratory journal | Salameh P R | 2003 | 0 | http://www.ncbi.nlm.nih.gov/pubmed/?term=2003+Respiratory+symptoms+in+children+and+exposure+to+pesticides+salameh | | | |
| 500 | Gerontology | Sibai A M | 2003 | 0 | http://www.ncbi.nlm.nih.gov/pubmed/?term=Variations+in+nutritional+status+of+elderly+men+and+women+according+to+place+of+residence | | | |
| 665 | Microsurgery | Moucharafieh RS | 2003 | 0 | http://www.ncbi.nlm.nih.gov.ezproxy.aub.edu.lb/pubmed/12833328 | | | |
| 712 | J Asthma | Tamim H | 2003 | 0 | http://www.ncbi.nlm.nih.gov/pubmed/14529107 | | | |
| 713 | Addiction | Tamim H | 2003 | 0 | http://www.ncbi.nlm.nih.gov/pubmed/12814499 | | | |
| 941 | Obesity reviews | Sibai AM | 2003 | 0 | http://www.ncbi.nlm.nih.gov/pubmed/14627756 | | | |
| 969 | International journal of cardiology | Dakik HA | 2003 | 0 | http://www.ncbi.nlm.nih.gov/pubmed/14659861 | | | |
| 147 | Journal of nuclear cardiology | Dakik H A | 2003 | 0 | http://www.ncbi.nlm.nih.gov/pubmed/12673175 | | | |
| 236 | Journal of endocrinological investigation | Gannage-Yared M H | 2003 | 0 | http://www.ncbi.nlm.nih.gov/pubmed/?term=Effects+of+a+short-term+calcium+and+vitamin+D+treatment+on+serum+cytokines%2C+bone+markers%2C+insulin+and+lipid+concentrations+in+healthy+post-menopausal+women | | | |
| 955 | Am J Cardiol | Azar RR | 2003 | 0 | http://www.ncbi.nlm.nih.gov/pubmed/12860235 | | | |
| 184 | The Biochemical journal | El-Assaad W | 2003 | 0 | http://www.ncbi.nlm.nih.gov/pubmed/?term=Ceramide+and+glutathione+define+two+independently+regulated+pathways+of+cell+death+initiated+by+p53+in+Molt-4+leukaemia+cells | | | |
| 305 | International journal of cardiology | Jabbour S | 2003 | 0 | http://www.ncbi.nlm.nih.gov/pubmed/?term=Information+and+communication+technology+in+cardiovascular+disease+prevention+in+developing+countries%3A+hype+and+hope.+Report+of+the+International+Collaboration+on+Information+Use+in+Cardiovascular+Health+Promotio | | | |
| 395 | Blood | Nasr R | 2003 | 0 | http://www.ncbi.nlm.nih.gov.ezproxy.aub.edu.lb/pubmed/12560223 | | | |
| 590 | Food and chemical toxicology | Daher CF | 2003 | 0 | http://www.ncbi.nlm.nih.gov/pubmed/12963008 | | | |
| 677 | Prostaglandins, Leukotrienes and Essential Fatty Acids | Nemr R | 2003 | 0 | http://www.ncbi.nlm.nih.gov.ezproxy.aub.edu.lb/pubmed/?term=Effects+of+nicotine+on+thromboxane%2Fprostacyclin+balance+in+myocardial+ischemia. | | | |
| 705 | Food and chemical toxicology | Shihadeh A | 2003 | 0 | http://www.ncbi.nlm.nih.gov/pubmed/12453738 | | | |
| 36 | Education for health | Afifi Soweid R | 2003 | 0 | http://www.ncbi.nlm.nih.gov/pubmed/?term=Changes+in+health-related+attitude+and+self-reported+behaviour+of+undergraduate+students+at+the+American+university+of+Beirut+following+a+health+awareness+course | | | |
| 480 | European journal of gynaecological oncology | Seoud M | 2003 | 0 | http://www.ncbi.nlm.nih.gov/pubmed/?term=Short+duration+neoadjuvant+chemotherapy+followed+by+radiotherapy+for+advanced+carcinoma+of+the+cervix%3A+results+and+prognostic+variables | | | |
| 214 | Annales de biologie clinique | El Kassimi B | 2003 | 1 | http://www.ncbi.nlm.nih.gov/pubmed/12825551 | | | |
| 746 | Revue de Pneumologie Clinique | Rguibi M | 2003 |  | http://www.ncbi.nlm.nih.gov/pubmed/14707927 | | | |
| 749 | La Presse Médicale | Rguibi M | 2003 |  | http://www.ncbi.nlm.nih.gov/pubmed/12733389 | | | |
| 38 | Journal of neuroradiology | Akhaddar A | 2003 | 0 | http://www.ncbi.nlm.nih.gov/pubmed/12717299 | | | |
| 159 | Journal français d'ophtalmologie | Charif Chefchaouni M | 2003 | 1 | http://www.ncbi.nlm.nih.gov/pubmed/12746604 | | | |
| 318 | Annales de Pathologie | Jabri L | 2003 | 1 | http://www.ncbi.nlm.nih.gov/pubmed/12743502 | | | |
| 320 | Scandinavian journal of urology and nephrology | Janane A | 2003 | 0 | http://www.ncbi.nlm.nih.gov/pubmed/12745734 | | | |
| 431 | Cancer Radiothérapie | Omari-Alaoui H | 2003 | 0 | http://www.ncbi.nlm.nih.gov/pubmed/14522353 | | | |
| 747 | Joint Bone Spine | Rguibi M | 2003 | 0 | http://www.ncbi.nlm.nih.gov/pubmed/14563474 | | | |
| 748 | Revue de Pneumologie Clinique | Rguibi M | 2003 | 1 | http://www.ncbi.nlm.nih.gov/pubmed/13130205 | | | |
| 994 | Revue de stomatologie et de chirurgie maxillo-faciale | Boulaich M | 2003 | 1 | http://www.ncbi.nlm.nih.gov/pubmed/14968099 | | | |
| 995 | Revue de pneumologie clinique | Regragui A | 2003 | 1 | http://www.ncbi.nlm.nih.gov/pubmed/14745343 | | | |
| 997 | Revue de pneumologie clinique | Soualhi M | 2003 | 1 | http://www.ncbi.nlm.nih.gov/pubmed/14699295 | | | |
| 998 | Journal français d'ophtalmologie | Souhail H | 2003 | 1 | http://www.ncbi.nlm.nih.gov/pubmed/14691403 | | | |
| 999 | Annales d'Urologie | Ameur A | 2003 | 1 | http://www.ncbi.nlm.nih.gov/pubmed/14606314 | | | |
| 1000 | Annales de Chirugie | Benchekroun A | 2003 | 1 | http://www.ncbi.nlm.nih.gov/pubmed/14559310 | | | |
| 1001 | Cancer radiothérapie | Amaoui B | 2003 | 1 | http://www.ncbi.nlm.nih.gov/pubmed/14522352 | | | |
| 1002 | Journal de radiologie | Chat L | 2003 | 1 | http://www.ncbi.nlm.nih.gov/pubmed/13677829 | | | |
| 1004 | Annales d'Urologie | Soualy K | 2003 | 1 | http://www.ncbi.nlm.nih.gov/pubmed/12951708 | | | |
| 1005 | Progrès en urologie | el Mejjad A | 2003 | 1 | http://www.ncbi.nlm.nih.gov/pubmed/12940210 | | | |
| 1006 | Revue de stomatologie et de chirurgie maxillo-faciale | Boulaich M | 2003 | 1 | http://www.ncbi.nlm.nih.gov/pubmed/12931069 | | | |
| 1007 | Bulletin de la Société belge d'ophtalmologie | Moutaouakil A | 2003 | 1 | http://www.ncbi.nlm.nih.gov/pubmed/12879721 | | | |
| 1008 | Annales d'Urologie | Benchekroun A | 2003 | 1 | http://www.ncbi.nlm.nih.gov/pubmed/12872605 | | | |
| 1010 | Annales de Chirugie | Alaoui OA | 2003 | 1 | http://www.ncbi.nlm.nih.gov/pubmed/12853025 | | | |
| 1011 | Cancer radiothérapie | SaÃ¢di I | 2003 | 1 | http://www.ncbi.nlm.nih.gov/pubmed/12834776 | | | |
| 1012 | Cancer radiothérapie | SaÃ¢di I | 2003 | 1 | http://www.ncbi.nlm.nih.gov/pubmed/12834775 | | | |
| 1013 | La Revue de médecine interne | Mikdame M | 2003 | 1 | http://www.ncbi.nlm.nih.gov/pubmed/12829219 | | | |
| 1014 | La Revue de médecine interne | Ennibi K | 2003 | 1 | http://www.ncbi.nlm.nih.gov/pubmed/12829218 | | | |
| 1015 | Bulletin de la Société belge d'ophtalmologie | Hajji Z | 2003 | 1 | http://www.ncbi.nlm.nih.gov/pubmed/12784574 | | | |
| 1016 | Progrès en urologie | el Malki HO | 2003 | 1 | http://www.ncbi.nlm.nih.gov/pubmed/12765067 | | | |
| 1017 | Revue de stomatologie et de chirurgie maxillo-faciale | Mahmal L | 2003 | 1 | http://www.ncbi.nlm.nih.gov/pubmed/12750630 | | | |
| 1018 | Revue de stomatologie et de chirurgie maxillo-faciale | El Gbouri H | 2003 | 1 | http://www.ncbi.nlm.nih.gov/pubmed/12750629 | | | |
| 1019 | Neurochirurgie | El Khorassani M | 2003 | 1 | http://www.ncbi.nlm.nih.gov/pubmed/12746729 | | | |
| 1021 | Annales d'Urologie | Ghadouane M | 2003 | 1 | http://www.ncbi.nlm.nih.gov/pubmed/12741197 | | | |
| 1022 | Annales d'Urologie | Janane A | 2003 | 1 | http://www.ncbi.nlm.nih.gov/pubmed/12741192 | | | |
| 1023 | Neurochirurgie | Kanouni L | 2003 | 1 | http://www.ncbi.nlm.nih.gov/pubmed/12736579 | | | |
| 1024 | Gynécologie, obstétrique & fertilité | Kably A | 2003 | 1 | http://www.ncbi.nlm.nih.gov/pubmed/12718987 | | | |
| 1026 | Journal de radiologie | Nassar I | 2003 | 1 | http://www.ncbi.nlm.nih.gov/pubmed/12717290 | | | |
| 1028 | Annales d'Urologie | Benchekroun A | 2003 | 1 | http://www.ncbi.nlm.nih.gov/pubmed/12701322 | | | |
| 1029 | Annales d'Urologie | Benchekroun A | 2003 | 1 | http://www.ncbi.nlm.nih.gov/pubmed/12701313 | | | |
| 1032 | Annales de Chirugie | Chouhou L | 2003 | 1 | http://www.ncbi.nlm.nih.gov/pubmed/12600328 | | | |
| 59 | Sante Publique | Balafrej A | 2003 | 1 | http://www.ncbi.nlm.nih.gov/pubmed/12784491 | | | |
| 143 | Genes and Immunity | Bougbis L | 2003 | 0 | http://www.ncbi.nlm.nih.gov/pubmed/12618861 | | | |
| 155 | Bulletin de la Société de pathologie exotique | Chakib A | 2003 | 1 | http://www.ncbi.nlm.nih.gov/pubmed/12836521 | | | |
| 221 | journal of human genetics | El Messal M | 2003 | 0 | http://www.ncbi.nlm.nih.gov/pubmed/12730724 | | | |
| 711 | Journal of Ethnopharmacology | El-Hilaly J | 2003 | 0 | http://www.ncbi.nlm.nih.gov/pubmed/12738079 | | | |
| 1027 | Presse médicale | Benamar L | 2003 | 1 | http://www.ncbi.nlm.nih.gov/pubmed/12714902 | | | |
| 1031 | Journal français d'ophtalmologie | Hajji Z | 2003 | 1 | http://www.ncbi.nlm.nih.gov/pubmed/12610409 | | | |
| 406 | International Urology and Nephrology | Moudouni S M | 2003 | 0 | http://www.ncbi.nlm.nih.gov/pubmed/15072484 | | | |
| 260 | Asia pacific journal of clinical nutrition | Galal O | 2003 | 0 | http://www.ncbi.nlm.nih.gov/pubmed/14505998 | | | |
| 754 | International Journal of Tuberculosis and Lung Disease | Nejjari C | 2003 | 0 | http://www.ncbi.nlm.nih.gov/pubmed/12661835 | | | |
| 1025 | Revue de pneumologie clinique | Bentaleb F | 2003 | 1 | http://www.ncbi.nlm.nih.gov/pubmed/12717323 | | | |
| 125 | Journal of Ethnopharmacology | Berrougui H | 2003 | 0 | http://www.ncbi.nlm.nih.gov/pubmed/14522427 | | | |
| 142 | Tissue Antigens | Bougbis L | 2003 | 0 | http://www.ncbi.nlm.nih.gov/pubmed/12622777 | | | |
| 186 | Phytomedicine | Eddouks M | 2003 | 0 | http://www.ncbi.nlm.nih.gov/pubmed/13678249 | | | |
| 327 | Journal of Ethnopharmacology | Jouad H | 2003 | 0 | http://www.ncbi.nlm.nih.gov/pubmed/12787953 | | | |
| 337 | European journal of cancer prevention | Khallouki F | 2003 | 0 | http://www.ncbi.nlm.nih.gov/pubmed/12548113 | | | |
| 374 | Journal of Ethnopharmacology | Maghrani M | 2003 | 0 | http://www.ncbi.nlm.nih.gov/pubmed/12787950 | | | |
| 400 | J Environ Radioact | Misdaq M A | 2003 | 0 | http://www.ncbi.nlm.nih.gov/pubmed/12691719 | | | |
| 1009 | Annales d'Urologie | Lezrek M | 2003 | 1 | http://www.ncbi.nlm.nih.gov/pubmed/12872603 | | | |
| 1020 | Neurochirurgie | Regragui A | 2003 | 1 | http://www.ncbi.nlm.nih.gov/pubmed/12746721 | | | |
| 511 | J Hypertens | Tazi MA | 2003 | 0 | http://www.ncbi.nlm.nih.gov/pubmed/12714863 | | | |
| 50 | J Clin Virol | Amrani M | 2003 | 0 | http://www.ncbi.nlm.nih.gov/pubmed/12878093 | | | |
| 525 | Journal of nephrology | Salvadori M | 2003 | 0 | http://www.ncbi.nlm.nih.gov/pubmed/14733407 | | | |
| 98 | Thérapie | Benkhalti F | 2003 | 1 | http://www.ncbi.nlm.nih.gov/pubmed/12942853 | | | |
| 130 | Fitoterapia | Bnouham M | 2003 | 0 | http://www.ncbi.nlm.nih.gov/pubmed/14630172 | | | |
| 61 | Int J Environ Health Res | Al-Khatib I | 2003 | 0 | http://www.ncbi.nlm.nih.gov/pubmed/14594698 | | | |
| 63 | Clin Exp Allergy | El-Sharif N | 2003 | 0 | http://www.ncbi.nlm.nih.gov/pubmed/12580909 | | | |
| 64 | Ann Allergy Asthma Immunol | El-Sharif NA | 2003 | 0 | http://www.ncbi.nlm.nih.gov/pubmed/12546340 | | | |
| 123 | International journal of obesity | Abdul-Rahim HF | 2003 | 0 | http://www.ncbi.nlm.nih.gov/pubmed/12532166 | | | |
| 132 | Medical science monitor: international medical journal of experimental and clinical research | Husseini A | 2003 | 0 | http://www.ncbi.nlm.nih.gov.ezproxy.aub.edu.lb/pubmed/12761454 | | | |
| 108 | Journal of nephrology | Sabeel A I | 2003 | 0 | https://www.ncbi.nlm.nih.gov.ezproxy.aub.edu.lb/pubmed/?term=Kaposi's+sarcoma+in+Sudanese+renal+transplant+recipients%3A+a+report+from+a+single+center | | | |
| 21 | Anticancer research | Ahmed H G | 2003 | 0 | https://www.ncbi.nlm.nih.gov.ezproxy.aub.edu.lb/pubmed/?term=Study+of+oral+epithelial+atypia+among+Sudanese+tobacco+users+by+exfoliative+cytology | | | |
| 84 | Saudi medical journal | Kheir M M | 2003 | 0 | https://www.ncbi.nlm.nih.gov.ezproxy.aub.edu.lb/pubmed/?term=Hypertension+in+type+2+diabetic+patients+Saudi+medical+journal+Kheir%2C+M.+M. | | | |
| 158 | Diabetes Research and Clinical Practice | Abdelgadir M | 2003 | 0 | http://www.ncbi.nlm.nih.gov/pubmed/12639764 | | | |
| 171 | Anticancer Research | Ibrahim SO | 2003 | 0 | http://www.ncbi.nlm.nih.gov/pubmed/14981901 | | | |
| 77 | Oral oncology | Ibrahim S O | 2003 | 0 | https://www.ncbi.nlm.nih.gov.ezproxy.aub.edu.lb/pubmed/?term=Gene+expression+profile+in+oral+squamous+cell+carcinomas+and+matching+normal+oral+mucosal+tissues+from+black+Africans+and+white+Caucasians%3A+the+case+of+the+Sudan+vs.+Norway | | | |
| 60 | Journal of Biosocial Sciences | Musaiger AO | 2004 | 0 | http://www.ncbi.nlm.nih.gov/pubmed/15535459 | | | |
| 62 | Journal of Thrombosis Thrombolysis | Almawi WY | 2004 | 0 | http://www.ncbi.nlm.nih.gov/pubmed/15353918 | | | |
| 64 | Transplantation Proceedings | Al-Hermi B | 2004 | 0 | http://www.ncbi.nlm.nih.gov/pubmed/15350485 | | | |
| 66 | Nutrition and Health | Musaiger AO | 2004 | 0 | http://www.ncbi.nlm.nih.gov/pubmed/15174736 | | | |
| 68 | Clinical and Diagnostic Laboratory Immunology | Al-Harbi EM | 2004 | 0 | http://www.ncbi.nlm.nih.gov/pubmed/15013978 | | | |
| 69 | Saudi Medical Journal | Shome DK | 2004 | 0 | http://www.ncbi.nlm.nih.gov/pubmed/14968210 | | | |
| 71 | Journal of Evaluation in Clinical Practice | Sequeira RP | 2004 | 0 | http://www.ncbi.nlm.nih.gov/pubmed/14731158 | | | |
| 150 | Eastern Mediterranean health journal | Musaiger AO | 2004 | 0 | http://www.ncbi.nlm.nih.gov/pubmed/16335765 | | | |
| 65 | Child Care Health and Development | Al-Sendi AM | 2004 | 0 | http://www.ncbi.nlm.nih.gov/pubmed/15191428 | | | |
| 56 | Annals of Saudi Medicine | Alnasir FA | 2004 | 0 | http://www.ncbi.nlm.nih.gov/pubmed/15646163 | | | |
| 58 | Aging clinical and experimental research | Al Khaja KA | 2004 | 0 | http://www.ncbi.nlm.nih.gov/pubmed/15575127 | | | |
| 63 | Transplantation Proceedings | Almawi WY | 2004 | 0 | http://www.ncbi.nlm.nih.gov/pubmed/15350494 | | | |
| 67 | Saudi Medical Journal | Behbehani NN | 2004 | 0 | http://www.ncbi.nlm.nih.gov/pubmed/15138525 | | | |
| 151 | Eastern Mediterranean health journal | Alnasir FA | 2004 | 0 | http://www.ncbi.nlm.nih.gov/pubmed/?term=Schoolteachers'+knowledge+of+common+health+problems+in+Bahrain. | | | |
| 61 | Saudi Medical Journal | Hamadeh RR | 2004 | 0 | http://www.ncbi.nlm.nih.gov/pubmed/15448757 | | | |
| 161 | Nutrition Metabolism and Cardiovasc Diseases | Al-Tamer YY | 2004 |  | http://www.ncbi.nlm.nih.gov/pubmed/?term=Lipid+components+and+fatty+acid+composition+of+Iraqi+subjects+who+smoke+and+consume+dairy+products. | | | |
| 264 | European Journal of Clinical Nutrition | Al-Tamer YY | 2004 | 0 | http://www.ncbi.nlm.nih.gov/pubmed/15054424 | | | |
| 214 | Neurosciences | Al-Mahdawi AM | 2004 | 0 | http://www.ncbi.nlm.nih.gov/pubmed/23377243 | | | |
| 156 | Saudi Medical Journal | Abdul-Rahman MM | 2004 | 0 | http://www.ncbi.nlm.nih.gov/pubmed/15711663 | | | |
| 158 | Electromyography and clinical neurophysiology | Al-Azzawi LM | 2004 | 0 | http://www.ncbi.nlm.nih.gov/pubmed/?term=The+usefulness+of+the+brainstem+auditory+evoked+potential+in+the+early+diagnosis+of+cranial+nerve+neuropathy | | | |
| 159 | Electromyography and clinical neurophysiology | Al-Azzawi LM | 2004 | 0 | http://www.ncbi.nlm.nih.gov/pubmed/?term=The+usefulness+of+the+blink+reflex+in+the+early+diagnosis+of+cranial+nerve+neuropathy+associated+with+diabetes+mellitus. | | | |
| 234 | Diagnostic Cytopathology | Haji BE | 2004 | 0 | http://onlinelibrary.wiley.com.ezproxy.aub.edu.lb/doi/10.1002/dc.20063/pdf | | | |
| 251 | Medical Principles and Practice | mallik AA | 2004 | 0 | http://www.karger.com.ezproxy.aub.edu.lb/Article/Pdf/81926 | | | |
| 283 | APMIS | Abbas M | 2004 | 0 | http://www.ncbi.nlm.nih.gov/pubmed/15511276 | | | |
| 305 | Medical Principles and Practice | Al Meshan MK | 2004 | 0 | http://www.ncbi.nlm.nih.gov/pubmed/15316266 | | | |
| 310 | Pediatric Dermatology | Al Saleh QA | 2004 | 0 | http://www.ncbi.nlm.nih.gov/pubmed/15461762 | | | |
| 882 | Medical Principles and Practice | Das DK | 2004 | 0 | http://www.ncbi.nlm.nih.gov/pubmed/14755143 | | | |
| 72 | Medical Principles and Practice | Zubaid M | 2004 | 0 | http://www.karger.com/Article/Pdf/75630 | | | |
| 73 | Medical Principles and Practice | Shorkhou El | 2004 | 0 | http://www.ncbi.nlm.nih.gov/pubmed/?term=Prevalence+of+metabolic+syndrome+among+hypertensive+patients+attending+a+primary++++++++care+clinic+in+Kuwait | | | |
| 143 | Pediatric Diabetes | Alsaeid M | 2004 | 0 | http://www.ncbi.nlm.nih.gov/pubmed/?term=Angiotensin-converting+enzyme+gene+polymorphism+and+lipid+profiles+in+Kuwaiti+children+with+type+1+diabetes | | | |
| 190 | Annals of Nutrition Metabolism | Moussa MA | 2004 | 0 | http://www.ncbi.nlm.nih.gov.ezproxy.aub.edu.lb/pubmed/?term=Lipoprotein(a)+and+other+cardiovascular+metabolic+risk+factors+in+Kuwaiti+children+with+type-1+diabetes | | | |
| 196 | Medical Principles and Practice | Muller HP | 2004 | 0 | http://www.ncbi.nlm.nih.gov/pubmed/?term=Screening+of+elevated+glucose+levels+in+gingival+crevice+blood+using+a+novel%2C+sensitive+self-monitoring+device | | | |
| 210 | Acta Cardiologica | Thalib L | 2004 | 0 | http://www.ncbi.nlm.nih.gov/pubmed/?term=Diabetes+mellitus+as+a+contributor+to+the+in-hospital+mortality+after+acute+myocardial+infa7ion+in+Kuwait | | | |
| 252 | Diagnostic Cytopathology | Mallik MK | 2004 | 0 | http://onlinelibrary.wiley.com.ezproxy.aub.edu.lb/doi/10.1002/dc.20022/pdf | | | |
| 254 | European Journal of Cancer | Memon A | 2004 | 0 | http://www.ncbi.nlm.nih.gov.ezproxy.aub.edu.lb/pubmed/?term=In+a+population-based+study+of+313+case-control+pairs+in+Kuwait%2C+we+evaluated+whether+a+family+history+of+benign+thyroid+disease+(BTD)+and+thyroid+or+other+cancers+was+associated+with+an+increased+risk+of+thyroid+cancer%2C+the+second+most+common+neoplasm+among+women+in+this+and+several+other+Arab+countries+in+the+Gulf+region | | | |
| 267 | Medical Principles and Practice | Shah Syed GM | 2004 | 0 | http://www.ncbi.nlm.nih.gov/pubmed/?term=To+investigate+the+correlation+between+gallium-67+(67Ga)+uptake+and+histological+subtypes+of+Hodgkin's+disease+(HD)+in+paediatric+patients | | | |
| 296 | Medical Principles and Practice | Al Azemi | 2004 | 0 | http://www.ncbi.nlm.nih.gov/pubmed/14657616 | | | |
| 311 | Molecular and Cellular Biochemisty | Al Sayer H | 2004 | 0 | http://www.ncbi.nlm.nih.gov/pubmed/15228079 | | | |
| 312 | Medical Principles and Practice | Al Shemmari SH | 2004 | 0 | http://www.ncbi.nlm.nih.gov/pubmed/15181324 | | | |
| 386 | Acta Diabetologica | Al-Adsani A | 2004 | 0 | http://www.ncbi.nlm.nih.gov/pubmed/15666581 | | | |
| 400 | Annals of Allergy, Asthma and Immunology | Al-Dowaisan A | 2004 | 0 | http://www.ncbi.nlm.nih.gov/pubmed/14989397 | | | |
| 421 | Nutrition and Health | Al-Isa AN | 2004 | 0 | http://www.ncbi.nlm.nih.gov/pubmed/15615328 | | | |
| 452 | Clinica Chimica Acta | Al-Rashidi M | 2004 | 0 | http://www.ncbi.nlm.nih.gov/pubmed/15149883 | | | |
| 462 | Annals of Nutrition and metabolism | Al-Shayji IA | 2004 | 0 | http://www.ncbi.nlm.nih.gov/pubmed/14639040 | | | |
| 594 | Archives of disease in childhood | Hijazi Z | 2004 | 0 | http://www.ncbi.nlm.nih.gov.ezproxy.aub.edu.lb/pubmed/14977697 | | | |
| 868 | Medical Principles and Practice | Al-Shemmari SH | 2004 | 0 | http://www.ncbi.nlm.nih.gov/pubmed/14755141 | | | |
| 270 | Leukemia and Lymphoma | Temmim L | 2004 | 0 | http://www.ncbi.nlm.nih.gov/pubmed/?term=Kuwait+was+chosen+by+the+International+Lymphoma+Study+Group+(ILSG)+as+one+of+the+sites+attending+in+the+project+on+%22Clinical+characteristics+and+pathological+classification+of+non+Hodgkin's+lymphoma+(NHL)+in+the+developing+countries | | | |
| 529 | Indian Journal of Pathology and Microbiology | Das DK | 2004 | 0 | http://www.ncbi.nlm.nih.gov/pubmed/16295413 | | | |
| 69 | Family Medicine | Al-Shaibani H | 2004 | 0 | http://www.ncbi.nlm.nih.gov/pubmed/?term=Prevalence+of+insulin+resistance+syndrome+in+a+primary+health+care+center+in+++++++Kuwait | | | |
| 71 | Pharmacology | Sharma JN | 2004 | 0 | http://www.ncbi.nlm.nih.gov/pubmed/?term=Tissue+kallikrein+increases+duration+of+survival+after+prolonged+coronary+artery++++++++ligation+in+hypertensive+rats | | | |
| 162 | Hormone and Metabolic Research | Bitar MS | 2004 | 0 | http://www.ncbi.nlm.nih.gov.ezproxy.aub.edu.lb/pubmed/?term=Alpha-lipoic+acid+mitigates+insulin+resistance+in+Goto-Kakizaki+rats | | | |
| 167 | Journal of Biomedical Science | Dhaunsi GS | 2004 | 0 | http://www.ncbi.nlm.nih.gov.ezproxy.aub.edu.lb/pubmed/?term=Antioxidants+attenuate+diabetes-induced+activation+of+peroxisomal+functions+in+the+rat+kidney | | | |
| 220 | Medical Principles and Practice | Yousif MH | 2004 | 0 | http://www.ncbi.nlm.nih.gov/pubmed/?term=nhibition+of+Ras-GTPase+improves+diabetes-induced+abnormal+vascular+reactivity+in+the+rat+perfused+mesenteric+vascular+bed | | | |
| 236 | Cytopathology | Joqai S | 2004 | 0 | http://www.ncbi.nlm.nih.gov.ezproxy.aub.edu.lb/pubmed/?term=The+cytological+diagnosis+of+classical+papillary+carcinoma+is+easily+established+based+on+the+characteristic+architectural+and+nuclear+features | | | |
| 328 | Journal of Clinical Pathology | Dey P | 2004 | 0 | http://www.ncbi.nlm.nih.gov/pubmed/15563660 | | | |
| 515 | Molecular and Cellular Biochemistry | Benter IF | 2004 | 0 | http://www.ncbi.nlm.nih.gov/pubmed/15124905 | | | |
| 525 | Biological Trace Element Research | Chen MD | 2004 | 0 | http://www.ncbi.nlm.nih.gov/pubmed/15258323 | | | |
| 646 | Journal of Pharmacological Sciences | Mahmoud FF | 2004 | 0 | http://www.ncbi.nlm.nih.gov.ezproxy.aub.edu.lb//pubmed/14978350 | | | |
| 194 | Metabolism | Moussa MA | 2004 | 0 | http://www.ncbi.nlm.nih.gov.ezproxy.aub.edu.lb/pubmed/?term=Association+of+serum+sialic+acid+with+cardiovascular+metabolic+risk+factors+in+Kuwaiti+children+and+adolescents+with+type+1+diabetes | | | |
| 316 | Saudi Medical Journal | Andejani AA | 2004 | 0 | http://www.ncbi.nlm.nih.gov/pubmed/15573182 | | | |
| 420 | European Journal of Clinical Nutrition | Al-Isa AN | 2004 | 0 | http://www.ncbi.nlm.nih.gov/pubmed/15054423 | | | |
| 461 | Annals of Saudi Medicine | Al-Shammari S | 2004 | 0 | http://www.ncbi.nlm.nih.gov/pubmed/15573849 | | | |
| 513 | Saudi Medical Journal | Behbehani NN | 2004 | 0 | http://www.ncbi.nlm.nih.gov/pubmed/15138525 | | | |
| 568 | Eastern Medditerranian Health Journal | El-Shazly M | 2004 | 0 | http://www.ncbi.nlm.nih.gov/pubmed/16201708 | | | |
| 279 | Rheumatology International | Uppal SS | 2004 | 0 | http://www.ncbi.nlm.nih.gov/pubmed/?term=Focal+myositis%2C+the+much+rarer+clinical+and+pathological+variant+of+polymyositis%2C+is+generally+a+benign+inflammatory+pseudotumor | | | |
| 294 | International journal of gynaecology and obstetrics | Al Bahar S | 2004 | 0 | http://www.ncbi.nlm.nih.gov/pubmed/15145269 | | | |
| 67 | Saudi Medical Journal | Jamal ST | 2004 | 0 | http://www.ncbi.nlm.nih.gov/pubmed/?term=Headache+and+blood+pressure+in+primary+health+care+setting+in+Kuwait | | | |
| 70 | Canadian Journal of Cardiology | Zubaid M | 2004 | 0 | http://www.ncbi.nlm.nih.gov/pubmed/15229759 | | | |
| 269 | European Journal of Gastroenterology and Hepatology | Siddique I | 2004 | 0 | http://www.ncbi.nlm.nih.gov/pubmed/?term=-+OBJECTIVE%3A+To+assess+the+prognostic+ability+of+the+Cancer+of+the+Liver+Italian+Programme+(CLIP)+score+and+compare+it+with+the+Okuda+system+in+patients+with+hepatocellular+carcinoma+from+the+Middle+East%2C+where+the+majority | | | |
| 271 | Oncology reports | Temmim L | 2004 | 0 | http://www.ncbi.nlm.nih.gov/pubmed/?term=A+total+of+935+patients+with+extranodal+non-Hodgkin+lymphoma+(NHL)+diagnosed+in+the+period+between+January+1985+and+December+2000+in+Kuwait+Cancer+Center%2C+serving+the+whole+population+of+Kuwait%2C+were+used+to+describe+the+clinicopathological+and+epidemiological+features+of+extranodal+lymphomas+in+Kuwait. | | | |
| 276 | Medical Principles and Practice | Thotathil ZS | 2004 | 0 | http://www.ncbi.nlm.nih.gov/pubmed/?term=OBJECTIVE%3A+Primary+tumors+of+the+trachea+are+extremely+rare.+Treatment+methods+vary+considerably+and+few+studies+have+sought+to+provide+adequate+guidelines | | | |
| 292 | Indian Journal of Cancer | Al Bahar S | 2004 | 0 | http://www.ncbi.nlm.nih.gov/pubmed/15472411 | | | |
| 324 | Acta Cytologica | Das DK | 2004 | 0 | http://www.ncbi.nlm.nih.gov/pubmed/15192947 | | | |
| 348 | Medical Principles and Practice | Abul A | 2004 | 0 | http://www.ncbi.nlm.nih.gov/pubmed/14755139 | | | |
| 506 | Journal of the Egyptian Public Health Association | Badr Hel-S | 2004 | 0 | http://www.ncbi.nlm.nih.gov/pubmed/17265610 | | | |
| 266 | Medical Principles and Practice | Shah Syed GM | 2004 | 0 | http://www.ncbi.nlm.nih.gov/pubmed/?term=OBJECTIVES%3A+To+evaluate+the+role+of+iodine-131+metoiodobenzylguanidine+(iodine-131+MIBG)+scanning+in+the+management+of+paediatric+patients+with+neuroblastoma | | | |
| 278 | Clinical Lymphoma | Tuli MM | 2004 | 0 | http://www.ncbi.nlm.nih.gov/pubmed/?term=The+aim+of+this+study+was+to+determine+whether+gallium+(Ga)-67+scintigraphy+can+monitor+the+treatment+response+rates+and+predict+the+long-term+clinical+outcome+in+patients+with+lymphoma | | | |
| 68 | The investigational Drugs | Sharma JN | 2004 | 0 | http://www.ncbi.nlm.nih.gov/pubmed/15478018 | | | |
| 291 | The Journal of Urology | Abuzallouf S | 2004 | 0 | http://www.ncbi.nlm.nih.gov/pubmed/15126770 | | | |
| 751 | IDrugs | Sharma JN | 2004 | 0 | http://www.ncbi.nlm.nih.gov/pubmed/15478018 | | | |
| 247 | International Journal of Oncology | Luqmani YA | 2004 | 0 | http://www.ncbi.nlm.nih.gov/pubmed/15289882 | | | |
| 260 | Blood cells, molecules and diseases | Naresh KN | 2004 | 0 | http://ac.els-cdn.com/S1079979604001573/1-s2.0-S1079979604001573-main.pdf?_tid=445744fc-3a68-11e3-8ec6-00000aacb35e&acdnat=1382370705_2bd5682212e61d65c48a4edf0a45b880 | | | |
| 293 | Neoplasma | Al Bahar S | 2004 | 0 | http://www.ncbi.nlm.nih.gov/pubmed/15254677 | | | |
| 323 | Diagnostic Cytopathology | Das DK | 2004 | 0 | http://www.ncbi.nlm.nih.gov/pubmed/15540187 | | | |
| 187 | Hemoglobin | Elhajj I | 2004 |  | Not Found | |  |  |
| 509 | Journal of the pancreas | Soweid A | 2004 |  | Abstract Not Found | |  |  |
| 513 | Journal of the American College of Nutrition | Tamim H | 2004 |  | Not Found | |  |  |
| 948 | Anaesthesia | Abchee AB | 2004 |  | http://www.ncbi.nlm.nih.gov/pubmed/14725527 | | | |
| 52 | Am J of neuroradiology | Anis N | 2004 | 0 | http://www.ncbi.nlm.nih.gov/pubmed/?term=Use+of+radio-frequency+ablation+for+the+palliative+treatment+of+sacral+chordoma | | | |
| 66 | Gynecologic oncology | Atallah D | 2004 | 0 | http://www.ncbi.nlm.nih.gov/pubmed/?term=Malignant+female+adnexal+tumor+of+probable+wolffian+origin+relapsing+after+pregnancy | | | |
| 129 | Breast | Chehal A | 2004 | 0 | http://www.ncbi.nlm.nih.gov/pubmed/?term=Cisplatin+plus+vinorelbine+(PVn)+as+a+salvage+regimen+for+refractory+breast+cancer | | | |
| 226 | Cancer genetics and cytogenetics | Farra C | 2004 | 0 | http://www.ncbi.nlm.nih.gov/pubmed/?term=Complex+translocation+(8%3B12%3B21)%3A+a+new+variant+of+t(8%3B21)+in+acute+myeloid+leukemia | | | |
| 345 | J Med Liban | Loutfi R | 2004 | 0 | http://www.ncbi.nlm.nih.gov/pubmed/?term=Spontaneous+pneumothorax+following+chemotherapy+for+metastatic+germ+cell+tumor%3A+a+case+report | | | |
| 365 | Annals of hematology | Mourad Y A | 2004 | 0 | http://www.ncbi.nlm.nih.gov/pubmed/?term=Successful+treatment+of+B-cell+prolymphocytic+leukemia+with+monoclonal+anti-CD20+antibody | | | |
| 946 | Am J Med Genet A | Shamseddine A | 2004 | 0 | http://www.ncbi.nlm.nih.gov/pubmed/14994233 | | | |
| 60 | Health care for women international | Arevian M | 2004 | 0 | http://www.ncbi.nlm.nih.gov/pubmed/?term=Risk+factors+for+coronary+artery+disease+(CAD)+in+Lebanese-Armenian+women | | | |
| 80 | Kidney international | Bahous S A | 2004 | 0 | http://www.ncbi.nlm.nih.gov/pubmed/15458442 | | | |
| 146 | The Canadian journal of cardiology | Dakik H A | 2004 | 0 | http://www.ncbi.nlm.nih.gov/pubmed/?term=Acute+myocardial+infarction%3A+clinical+characteristics%2C+management+and+outcome+in+a+university+medical+centre+in+a+developing+Middle+Eastern+country | | | |
| 376 | Journal of pediatric hematology/oncology | Muwakkit S A | 2004 | 0 | http://www.ncbi.nlm.nih.gov/pubmed/?term=Clinical+presentation+and+treatment+outcome+of+children+with+Burkitt+lymphoma+in+Lebanon%3A+a+single+institution's+experience | | | |
| 475 | Diabetes Care | Salti I | 2004 | 0 | http://www.ncbi.nlm.nih.gov/pubmed/?term=A+population-based+study+of+diabetes+and+its+characteristics+during+the+fasting+month+of+Ramadan+in+13+countries%3A+results+of+the+epidemiology+of+diabetes+and+Ramadan+1422%2F2001+(EPIDIAR)+study++2004 | | | |
| 584 | Nicotine Tob Res | Chaaya M | 2004 | 0 | http://www.ncbi.nlm.nih.gov/pubmed/15203779 | | | |
| 642 | Drug and Alcohol Dependence | Karam E | 2004 | 0 | http://www.ncbi.nlm.nih.gov/pubmed/?term=Alcohol+use+among+university+students+in+Lebanon%3A+prevalence%2C+trends+and+covariates.+The+IDRAC+University+Substance+Use+Monitoring+Study+(1991+and+1999). | | | |
| 662 | The Journal of clinical endocrinology and metabolism | Medlej R | 2004 | 0 | http://www.ncbi.nlm.nih.gov.ezproxy.aub.edu.lb/pubmed/15070927 | | | |
| 704 | Pharmacol Biochem Behav | Shihadeh A | 2004 | 0 | http://www.ncbi.nlm.nih.gov/pubmed/15388286 | | | |
| 485 | J Med Liban | Shamseddine A | 2004 | 0 | http://www.ncbi.nlm.nih.gov/pubmed/?term=Cisplatin+and+vinorelbine+(PVn)+for+the+treatment+of+advanced+breast+cancer%3A+10+years+of+experience | | | |
| 488 | Annals of epidemiology | Shamseddine A | 2004 | 0 | http://www.ncbi.nlm.nih.gov/pubmed/?term=Cancer+incidence+in+postwar+Lebanon%3A+findings+from+the+first+national+population-based+registry | | | |
| 496 | Bulletin of the World Health Organization | Sibai A M | 2004 | 0 | http://www.ncbi.nlm.nih.gov/pubmed/?term=Population+ageing+in+Lebanon%3A+current+status%2C+future+prospects+and+implications+for+policy | | | |
| 925 | Eastern Mediterranean health journal | Hwalla N | 2004 | 0 | http://www.ncbi.nlm.nih.gov/pubmed/16335757 | | | |
| 951 | Lancet Oncol | Bazarbachi A | 2004 | 0 | http://www.ncbi.nlm.nih.gov/pubmed/15522654 | | | |
| 153 | Leukemia | Darwiche N | 2004 | 0 | http://www.ncbi.nlm.nih.gov/pubmed/?term=N-(4-hydroxyphenyl)retinamide+induces+growth+arrest+and+apoptosis+in+HTLV-I-transformed+cells | | | |
| 280 | Chemico-biological interactions | Harakeh S | 2004 | 0 | http://www.ncbi.nlm.nih.gov/pubmed/?term=Inhibition+of+proliferation+and+induction+of+apoptosis+by+2-benzoyl-3-phenyl-6%2C7-dichloroquinoxaline+1%2C4-dioxide+in+adult+T-cell+leukemia+cells | | | |
| 304 | Bulletin of the World Health Organization | Jabbour S | 2004 | 0 | http://www.ncbi.nlm.nih.gov/pubmed/?term=Religion-based+tobacco+control+interventions%3A+how+should+WHO+proceed%3F | | | |
| 306 | Current controlled trials in cardiovascular medicine | Jabbour S | 2004 | 0 | http://www.ncbi.nlm.nih.gov/pubmed/?term=Unanswered+ethical+and+scientific+questions+for+trials+of+invasive+interventions+for+coronary+disease%3A+The+case+of+single+vessel+disease | | | |
| 315 | The Anatolian journal of cardiology | Jazra C | 2004 | 0 | http://www.ncbi.nlm.nih.gov/pubmed/?term=Some+aspects+of+cardiology+practice+in+Lebanon+jazra+2004 | | | |
| 553 | Clin Genet | Abifadel M | 2004 | 0 | http://www.ncbi.nlm.nih.gov/pubmed/14984478 | | | |
| 610 | Anti-cancer drugs | Gali-Muhtasib H | 2004 | 0 | http://www.ncbi.nlm.nih.gov/pubmed/?term=Molecular+pathway+for+thymoquinone-induced+cell-cycle+arrest+and+apoptosis+in+neoplastic+keratinocytes. | | | |
| 639 | Molecular Carcinogenesis | Kabbout M | 2004 | 0 | http://www.ncbi.nlm.nih.gov/pubmed/?term=Stage-specific+effect+of+N-(4-hydroxyphenyl)retinamide+on+cell+growth+in+squamous+cell+carcinogenesis. | | | |
| 979 | International journal of oncology | Gali-Muhtasib H | 2004 | 0 | http://www.ncbi.nlm.nih.gov/pubmed/15067333 | | | |
| 559 | Am J Ophthalmol | Al-Haddad CE | 2004 | 0 | http://www.ncbi.nlm.nih.gov/pubmed/15183815 | | | |
| 176 | Anti-cancer drugs | El Saghir N S | 2004 | 0 | http://www.ncbi.nlm.nih.gov/pubmed/?term=Docetaxel+extravasation+into+the+normal+breast+during+breast+cancer+treatment | | | |
| 735 | J Med Liban | Medlej-Hashim M | 2004 | 1 | http://www.ncbi.nlm.nih.gov/pubmed/?term=%5Bvon+Hippel-Lindau+syndrome%3A+molecular+diagnosis+of+two+Lebanese+families+and+analysis+of+the+genotype-phenotype+correlation%5D. | | | |
| 727 | J Med Liban | Zoughaib SS | 2004 | 0 | http://www.ncbi.nlm.nih.gov/pubmed/16432971 | | | |
| 393 | Clinical breast cancer | Nasr F L | 2004 | 0 | http://www.ncbi.nlm.nih.gov/pubmed/?term=Gemcitabine+plus+carboplatin+combination+therapy+as+second-line+treatment+in+patients+with+relapsed+breast+cancer | | | |
| 231 | International journal of oncology | Gali-Muhtasib H | 2004 | 0 | http://www.ncbi.nlm.nih.gov/pubmed/?term=Thymoquinone+extracted+from+black+seed+triggers+apoptotic+cell+death+in+human+colorectal+cancer+cells+via+a+p53-dependent+mechanism | | | |
| 2 | Diabetes & vascular disease research | | 2004 |  | http://www.ncbi.nlm.nih.gov/pubmed/16370094 | | | |
| 745 | Progrès en Urologie | Sbitti Y | 2004 |  | http://www.ncbi.nlm.nih.gov/pubmed/15217144 | | | |
| 36 | Surgical neurology | Akhaddar A | 2004 | 0 | http://www.ncbi.nlm.nih.gov/pubmed/15031083 | | | |
| 48 | Joint Bone Spine | Amine B | 2004 | 0 | http://www.ncbi.nlm.nih.gov/pubmed/15288860 | | | |
| 330 | Journal de radiologie | Kacemi L | 2004 | 1 | http://www.ncbi.nlm.nih.gov/pubmed/15094640 | | | |
| 973 | Progrès en urologie | Rabii R | 2004 | 1 | http://www.ncbi.nlm.nih.gov/pubmed/15776911 | | | |
| 974 | Progrès en urologie | Rifki Jai S | 2004 | 1 | http://www.ncbi.nlm.nih.gov/pubmed/15776910 | | | |
| 975 | Progrès en urologie | Tazi H | 2004 | 1 | http://www.ncbi.nlm.nih.gov/pubmed/15751422 | | | |
| 977 | Revue de laryngologie - otologie - rhinologie | Hallaoui Y | 2004 | 1 | http://www.ncbi.nlm.nih.gov/pubmed/15712693 | | | |
| 979 | Cancer radiothérapie | BenJelloun H | 2004 | 1 | http://www.ncbi.nlm.nih.gov/pubmed/15619383 | | | |
| 980 | Presse médicale | El-Idrissi F | 2004 | 1 | http://www.ncbi.nlm.nih.gov/pubmed/15523244 | | | |
| 981 | Revue médicale de Liège | Maliki M | 2004 | 1 | http://www.ncbi.nlm.nih.gov/pubmed/15493159 | | | |
| 982 | Progrès en urologie | Moufid K | 2004 | 1 | http://www.ncbi.nlm.nih.gov/pubmed/15373190 | | | |
| 985 | Journal de radiologie | Radouane B | 2004 | 1 | http://www.ncbi.nlm.nih.gov/pubmed/15243381 | | | |
| 986 | Archives de pédiatrie | El Kababri M | 2004 | 1 | http://www.ncbi.nlm.nih.gov/pubmed/15234374 | | | |
| 987 | Archives des maladies du coeur et des vaisseaux | El Mahi O | 2004 | 1 | http://www.ncbi.nlm.nih.gov/pubmed/15182080 | | | |
| 988 | Journal français d'ophtalmologie | Dahreddine M | 2004 | 1 | http://www.ncbi.nlm.nih.gov/pubmed/15179308 | | | |
| 989 | Gynécologie, obstétrique & fertilité | Kanouni L | 2004 | 1 | http://www.ncbi.nlm.nih.gov/pubmed/15123098 | | | |
| 990 | Revue de pneumologie clinique | Soualhi M | 2004 | 1 | http://www.ncbi.nlm.nih.gov/pubmed/15107669 | | | |
| 991 | Progrès en urologie | El Khader K | 2004 | 1 | http://www.ncbi.nlm.nih.gov/pubmed/15098760 | | | |
| 992 | Progrès en urologie | Rabii R | 2004 | 1 | http://www.ncbi.nlm.nih.gov/pubmed/15098759 | | | |
| 996 | Archives de pédiatrie | El Kababri M | 2004 | 1 | http://www.ncbi.nlm.nih.gov/pubmed/14700757 | | | |
| 67 | Public health nutrition | Belahsen R | 2004 | 0 | http://www.ncbi.nlm.nih.gov/pubmed/15153258 | | | |
| 111 | Annales de Biologie Clinique | Bennouar N | 2004 | 1 | http://www.ncbi.nlm.nih.gov/pubmed/15217762 | | | |
| 984 | Annales de Cardiologie et d'Angéiologie | El Honsali I | 2004 | 1 | http://www.ncbi.nlm.nih.gov/pubmed/15291169 | | | |
| 993 | Annales de Cardiologie et d'Angéiologie | Abir-Khalil S | 2004 | 1 | http://www.ncbi.nlm.nih.gov/pubmed/15038524 | | | |
| 84 | Saudi Medical Journal | Benchekroun M T | 2004 | 0 | http://www.ncbi.nlm.nih.gov/pubmed/15494846 | | | |
| 200 | Clinical and experimental Hypertension | El Bardai S | 2004 | 0 | http://www.ncbi.nlm.nih.gov/pubmed/15554450 | | | |
| 363 | Journal of Ethnopharmacology | Lemhadri A | 2004 | 0 | http://www.ncbi.nlm.nih.gov/pubmed/15138008 | | | |
| 364 | Journal of Ethnopharmacology | Lemhadri A | 2004 | 0 | http://www.ncbi.nlm.nih.gov/pubmed/15231045 | | | |
| 373 | Journal of Ethnopharmacology | Maghrani M | 2004 | 0 | http://www.ncbi.nlm.nih.gov/pubmed/15013197 | | | |
| 375 | Journal of Ethnopharmacology | Maghrani M | 2004 | 0 | http://www.ncbi.nlm.nih.gov/pubmed/15013198 | | | |
| 377 | Journal of Ethnopharmacology | Maghrani M | 2004 | 0 | http://www.ncbi.nlm.nih.gov/pubmed/15120454 | | | |
| 708 | Journal of Ethnopharmacology | Eddouks M | 2004 | 0 | http://www.ncbi.nlm.nih.gov/pubmed/15261976 | | | |
| 710 | Journal of Ethnopharmacology | El-Hilaly J | 2004 | 0 | http://www.ncbi.nlm.nih.gov/pubmed/15182907 | | | |
| 978 | Neoplasma | Aboudkhil S | 2004 | 0 | http://www.ncbi.nlm.nih.gov/pubmed/15640941 | | | |
| 452 | American Journal of Human Biology | Rguibi M | 2004 | 0 | http://www.ncbi.nlm.nih.gov/pubmed/15368608 | | | |
| 453 | Ethnicity and Disease | Rguibi M | 2004 | 0 | http://www.ncbi.nlm.nih.gov/pubmed/15724774 | | | |
| 474 | International Journal of Cancer | Smith JS | 2004 | 0 | http://www.ncbi.nlm.nih.gov/pubmed/15221973 | | | |
| 389 | J Nurs Scholarsh | McCarthy P | 2004 | 0 | http://www.ncbi.nlm.nih.gov/pubmed/15098413 | | | |
| 182 | Clinical Nutrition | Drissi A | 2004 | 0 | http://www.ncbi.nlm.nih.gov/pubmed/15380909 | | | |
| 124 | The British Journal of Nutrition | Berrougui H | 2004 | 0 | http://www.ncbi.nlm.nih.gov/pubmed/15613254 | | | |
| 187 | Journal of Ethnopharmacology | Eddouks M | 2004 | 0 | http://www.ncbi.nlm.nih.gov/pubmed/15261975 | | | |
| 59 | Saudi Med J | Baune BT | 2004 | 0 | http://www.ncbi.nlm.nih.gov/pubmed/15573202 | | | |
| 60 | Allergy | El Sharif N | 2004 | 0 | http://www.ncbi.nlm.nih.gov/pubmed/15147447 | | | |
| 108 | American journal of surgery | Nissan A | 2004 | 0 | http://www.ncbi.nlm.nih.gov.ezproxy.aub.edu.lb/pubmed/15219486 | | | |
| 101 | Nutrition and cancer | Omer R E | 2004 | 0 | http://www.ncbi.nlm.nih.gov/pubmed/?term=Population-attributable+risk+of+dietary+aflatoxins+and+hepatitis+B+virus+infection+with+respect+to+hepatocellular+carcinoma | | | |
| 148 | Saudi Medical Journal | Al-Haddad NM | 2005 |  | http://www.ncbi.nlm.nih.gov/pubmed/16380798 | | | |
| 49 | Saudi Medical Journal | Rajab KE | 2005 | 0 | http://www.ncbi.nlm.nih.gov/pubmed/16047069 | | | |
| 51 | Saudi Medical Journal | Rajab KE | 2005 | 0 | http://www.ncbi.nlm.nih.gov/pubmed/15983689 | | | |
| 52 | Diabetes Research and Clinical Practice | Al Khaja KA | 2005 | 0 | http://www.ncbi.nlm.nih.gov/pubmed/15890429 | | | |
| 53 | Journal of Evaluation in Clinical Practice | Al Khaja KA | 2005 | 0 | http://www.ncbi.nlm.nih.gov/pubmed/15813710 | | | |
| 47 | Clinical Microbiology and Infection | Senok AC | 2005 | 0 | http://www.ncbi.nlm.nih.gov/pubmed/16307549 | | | |
| 197 | International Journal of Food Sciences and Nutrition | Musaiger AO | 2005 | 0 | http://www.ncbi.nlm.nih.gov/pubmed/16096134 | | | |
| 48 | Saudi Medical Journal | Al Suwaidi J | 2005 | 0 | http://www.ncbi.nlm.nih.gov/pubmed/16228059 | | | |
| 50 | The Journal of clinical endocrinology and metabolism | Al-Jenaidi FA | 2005 | 0 | http://www.ncbi.nlm.nih.gov/pubmed/15985473 | | | |
| 54 | Saudi Medical Journal | Al-Hermi BE | 2005 | 0 | http://www.ncbi.nlm.nih.gov/pubmed/15770309 | | | |
| 57 | Clinical and Diagnostic Laboratory Immunology | Motala AA | 2005 | 0 | http://www.ncbi.nlm.nih.gov/pubmed/15643010 | | | |
| 146 | Saudi Medical Journal | Al-Hilli F | 2005 |  | http://www.ncbi.nlm.nih.gov/pubmed/16311687 | | | |
| 147 | Saudi Medical Journal | Sharquie KE | 2005 |  | http://www.ncbi.nlm.nih.gov/pubmed/?term=The+frequency+of+skin+diseases+in+obese+children+and+adult+Iraqi+population. | | | |
| 151 | Saudi Medical Journal | Hasso NM | 2005 |  | http://www.ncbi.nlm.nih.gov/pubmed/?term=Effect+of+antioxidant+serum+levels+of+myocardial+ischemia+markers+in+patients+with+ischemic+heart+disease+after+treadmill+exercise+testing. | | | |
| 154 | Saudi Medical Journal | Sharquie KE | 2005 |  | http://www.ncbi.nlm.nih.gov/pubmed/?term=New+intralesional+therapy+for+basal+cell+carcinoma+by+2%25+zinc+sulphate+solution. | | | |
| 211 | Ethnicity and Disease | Rice VH | 2005 |  | http://www.ncbi.nlm.nih.gov/pubmed/?term=Predictors+of+tobacco+use+among+Lebanese%2C+Yemeni%2C+and+Iraqi+adolescents%2C+14-18+years+of+age. | | | |
| 148 | Lancet | Khoshnaw AI | 2005 | 0 | http://www.ncbi.nlm.nih.gov/pubmed/16291065 | | | |
| 144 | Eastern Mediterranean Health Journal | Al-Thamiri D | 2005 | 0 | http://www.ncbi.nlm.nih.gov/pubmed/?term=Asthma+prevalence+and+severity+among+primary-school+children+in+Baghdad. | | | |
| 149 | Annals of Saudi Medicine | Lafta RK | 2005 | 0 | http://www.ncbi.nlm.nih.gov/pubmed/?term=Childhood+obesity+in+Iraq%3A+prevalence+and+possible+risk+factors. | | | |
| 153 | Saudi Medical Journal | Al-Kubaisy W | 2005 | 0 | http://www.ncbi.nlm.nih.gov/pubmed/15806220 | | | |
| 155 | Saudi Medical Journal | Hashim AR | 2005 | 0 | http://www.ncbi.nlm.nih.gov/pubmed/15756352 | | | |
| 210 | Saudi Medical Journal | Al-Timimi DJ | 2005 | 0 | http://www.ncbi.nlm.nih.gov/pubmed/?term=Zinc+deficiency+among+a+healthy+population+in+Baghdad%2C+Iraq. | | | |
| 212 | Neurosciences | Al-Mahdawi AM | 2005 | 0 | http://www.ncbi.nlm.nih.gov/pubmed/?term=Transient+ischemic+attack+and+ischemic+stroke%2C+risk+factors+and+preventive+roles+of+the+first+contact+physicians. | | | |
| 257 | Medical Principles and Practice | Mittal R | 2005 | 0 | http://www.karger.com.ezproxy.aub.edu.lb/Article/Pdf/84641 | | | |
| 325 | Diagnostic Cytopathology | Das DK | 2005 | 0 | http://www.ncbi.nlm.nih.gov/pubmed/16078258 | | | |
| 584 | Cytopathology | Haji BE | 2005 | 0 | http://www.ncbi.nlm.nih.gov.ezproxy.aub.edu.lb/pubmed/?term=Cytomorphological+features+of+metastatic+mammary+lobular+carcinoma+in+cervicovaginal+smears%3A+report+of+a+case+and+review+of+literature. | | | |
| 602 | Journal of the European Academy of Dermatology and Venereology | Hussein K | 2005 | 0 | http://www.ncbi.nlm.nih.gov.ezproxy.aub.edu.lb/pubmed/?term=Sweet's+syndrome+(acute+febrile+neutrophilic+dermatosis)+associated+with+adenocarcinoma+of+prostate+and+transitional+cell+carcinoma+of+urinary+bladder. | | | |
| 881 | Acta Cytologica | Das DK | 2005 | 0 | http://www.ncbi.nlm.nih.gov/pubmed/15717758 | | | |
| 66 | Diabetic Medicine | Abdella NA | 2005 | 0 | http://www.ncbi.nlm.nih.gov/pubmed/15717875 | | | |
| 102 | International Journal of Urology | Abdul-Halim H | 2005 | 0 | http://www.ncbi.nlm.nih.gov/pubmed/?term=-+Severe+emphysematous+pyelonephritis+in+diabetic+patients%3A+diagnosis+and+aspects+of+surgical+management | | | |
| 107 | Journal of Diabetes and its Complications | Abdulrazak A | 2005 | 0 | http://www.ncbi.nlm.nih.gov/pubmed/15866058 | | | |
| 134 | Medical Principles and Practice | AlKhawari HA | 2005 | 0 | http://www.ncbi.nlm.nih.gov/pubmed/?term=Evaluating+diabetic+foot+infection+with+magnetic+resonance+imaging%3A+Kuwait+experience | | | |
| 239 | Prostate Cancer and Prostatic Diseases | Kehinde EO | 2005 | 0 | http://www.nature.com.ezproxy.aub.edu.lb/pcan/journal/v8/n1/full/4500783a.html | | | |
| 240 | BJU international | Kehinde EO | 2005 | 0 | http://www.ncbi.nlm.nih.gov.ezproxy.aub.edu.lb/pubmed/?term=To+determine+age-specific+reference+ranges+for+serum+prostate-specific+antigen+(PSA)+concentration+and+prostate+volumes+in+a+population+of+healthy+Arab+men. | | | |
| 259 | Journal of the Egyptian National Cancer Institute | Motawy M | 2005 | 0 | http://www.nci.edu.eg/Journal/june%202004/CAN_3.PDF | | | |
| 321 | Gerontology | Das DK | 2005 | 0 | www.ncbi.nlm.nih.gov/pubmed/15832039 | | | |
| 467 | Psychological Reports | Alansari B | 2005 | 0 | http://www.ncbi.nlm.nih.gov/pubmed/16173370 | | | |
| 504 | Eastern Medditerranian Health Journal | Badr HE | 2005 | 0 | http://www.ncbi.nlm.nih.gov/pubmed/16532682 | | | |
| 505 | International Journal of Behavioral Medicine | Badr HE | 2005 | 0 | http://www.ncbi.nlm.nih.gov/pubmed/16262546 | | | |
| 674 | Transplantation Proceedings | Nampoory MR | 2005 | 0 | http://www.ncbi.nlm.nih.gov.ezproxy.aub.edu.lb/pubmed/16213300 | | | |
| 687 | Medical Principles and Practice | Onadeko BO | 2005 | 0 | http://www.karger.com/Article/Pdf/81921 | | | |
| 712 | Transplantation Proceedings | Samhan M | 2005 | 0 | http://www.ncbi.nlm.nih.gov.ezproxy.aub.edu.lb/pubmed/16213307 | | | |
| 898 | American Journal of Reproductive Immunology | Mahmoud F | 2005 | 0 | http://www.ncbi.nlm.nih.gov/pubmed/15667522 | | | |
| 722 | Journal of Clinical Ultrasound | Sheikh M | 2005 | 0 | http://www.ncbi.nlm.nih.gov.ezproxy.aub.edu.lb/pubmed/?term=Patients'+tolerance+and+early+complications+of+transrectal+sonographically+guided+prostate+biopsy%3A+prospective+study+of+300+patients | | | |
| 723 | International Urology and Nephrology | Sheikh M | 2005 | 0 | http://www.ncbi.nlm.nih.gov.ezproxy.aub.edu.lb/pubmed/?term=Utility+of+volume+adjusted+prostate+specific+antigen+density+in+the+diagnosis+of+prostate+cancer+in+Arab+men | | | |
| 60 | Scientific World Journal | Sharma JN | 2005 | 0 | http://www.ncbi.nlm.nih.gov/pubmed/16113940 | | | |
| 246 | Medical Principles and Practice | Luqmani YA | 2005 | 0 | http://www.karger.com.ezproxy.aub.edu.lb/Article/Pdf/86183 | | | |
| 329 | Diagnostic Cytopathology | Dey P | 2005 | 0 | http://www.ncbi.nlm.nih.gov/pubmed/15584052 | | | |
| 711 | Current Pharmaceutical Design | Saleh F | 2005 | 0 | http://www.ncbi.nlm.nih.gov.ezproxy.aub.edu.lb/pubmed/16248801 | | | |
| 59 | Autonomic and Autacoid Pharmacology | Benter IF | 2005 | 0 | http://www.ncbi.nlm.nih.gov/pubmed/16176445 | | | |
| 62 | Pharmacological Research | Benter IF | 2005 | 0 | http://www.ncbi.nlm.nih.gov/pubmed/16027001 | | | |
| 63 | Pharmacological Research | Sharma JN | 2005 | 0 | http://www.ncbi.nlm.nih.gov/pubmed/?term=A+bradykinin+antagonist+abolishes+beneficial+effect+of+captopril+on+duration+of+++++++survival+after+acute+coronary+artery+ligation+in+hypertensive+rats. | | | |
| 64 | Life Sciences | Bitar MS | 2005 | 0 | http://www.ncbi.nlm.nih.gov/pubmed/?term=Oxidative+stress--mediated+alterations+in+glucose+dynamics+in+a+genetic+animal+++++++model+of+type+II+diabetes | | | |
| 65 | European Journal of Pharmacology | Bitar MS | 2005 | 0 | http://www.ncbi.nlm.nih.gov/pubmed/?term=Nitric+oxide+dynamics+and+endothelial+dysfunction+in+type+II+model+of+genetic+++++++diabetes | | | |
| 137 | Molecular and Cellular Biochemistry | Al-Maghrebi M | 2005 | 0 | http://www.ncbi.nlm.nih.gov/pubmed/?term=Regulation+of+elongation+factor-1+expression+by+vitamin+E+in+diabetic+rat+kidneys | | | |
| 153 | Free Radical Research | Benov L | 2005 | 0 | http://www.ncbi.nlm.nih.gov.ezproxy.aub.edu.lb/pubmed/?term=A+manganese+porphyrin+suppresses+oxidative+stress+and+extends+the+life+span+of+streptozotocin-diabetic+rats | | | |
| 155 | Pharmacological Research | Benter IF | 2005 | 0 | http://www.ncbi.nlm.nih.gov/pubmed/15886012 | | | |
| 157 | Journal of Vascular Research | Benter IF | 2005 | 0 | http://www.ncbi.nlm.nih.gov/pubmed/15915001 | | | |
| 215 | Pharmacological Research | Yousif MH | 2005 | 0 | http://www.ncbi.nlm.nih.gov/pubmed/?term=Histamine-induced+vasodilation+in+the+perfused+kidney+of+STZ-diabetic+rats%3A+role+of+EDNO+and+EDHF | | | |
| 222 | Autonomic and Autacoid Pharmacology | Yousif MH | 2005 | 0 | http://www.ncbi.nlm.nih.gov/pubmed/?term=The+role+of+tyrosine+kinase-mediated+pathways+in+diabetes-induced+alterations+in+responsiveness+of+rat+carotid+artery | | | |
| 302 | Journal of International academy of Periodontology | Al Khalaf M | 2005 | 0 | http://www.ncbi.nlm.nih.gov/pubmed/19565011 | | | |
| 303 | Anticancer Research | Al Maghrebi M | 2005 | 0 | http://www.ncbi.nlm.nih.gov/pmc/articles/PMC3682968/ | | | |
| 307 | Journal of Histochemistry and Cytochemistry | Al Mulla F | 2005 | 0 | http://www.ncbi.nlm.nih.gov/pubmed/15872055 | | | |
| 315 | Medical Principles and Practice | Amirrad M | 2005 | 0 | http://www.ncbi.nlm.nih.gov/pubmed/15785095 | | | |
| 322 | Diagnostic Cytopathology | Das DK | 2005 | 0 | http://www.ncbi.nlm.nih.gov/pubmed/15830362 | | | |
| 516 | Molecular and Cellular Biochemistry | Benter IF | 2005 | 0 | http://www.ncbi.nlm.nih.gov/pubmed/15724451 | | | |
| 530 | Acta Cytologica | Das DK | 2005 | 0 | http://www.ncbi.nlm.nih.gov/pubmed/16124163 | | | |
| 710 | Current Pharmaceutical Design | Saleh F | 2005 | 0 | http://www.ncbi.nlm.nih.gov.ezproxy.aub.edu.lb/pubmed/?term=Direct+evidence+on+the+immune-mediated+spontaneous+regression+of+human+cancer%3A+an+incentive+for+pharmaceutical+companies+to+develop+a+novel+anti-cancer+vaccine | | | |
| 875 | European Journal of Nuclear Medicine and Molecular Imaging | Al-Saeedi F | 2005 | 0 | http://www.ncbi.nlm.nih.gov/pubmed/15660258 | | | |
| 877 | British Journal of Pharmacology | Benter IF | 2005 | 0 | http://www.ncbi.nlm.nih.gov/pubmed/15852031 | | | |
| 61 | Medical Principles and Practice | Al-Zuabi H | 2005 | 0 | http://www.ncbi.nlm.nih.gov/pubmed/16103693 | | | |
| 191 | Medical Principles and Practice | Moussa MA | 2005 | 0 | http://www.ncbi.nlm.nih.gov.ezproxy.aub.edu.lb/pubmed/15785099 | | | |
| 192 | Social Science and Medicine | Moussa MA | 2005 | 0 | http://www.ncbi.nlm.nih.gov.ezproxy.aub.edu.lb/pubmed/?term=Social+and+psychological+characteristics+of+Kuwaiti+children+and+adolescents+with+type+1+diabetes | | | |
| 195 | Acta Diabetologica | Moussa MA | 2005 | 0 | http://www.ncbi.nlm.nih.gov/pubmed/?term=Factors+associated+with+type+1+diabetes+in+Kuwaiti+children | | | |
| 497 | Archives of Environmental and Occupational Health | Arifhodzic NA | 2005 | 0 | http://www.ncbi.nlm.nih.gov/pubmed/17290844 | | | |
| 545 | Nutr J | Dehghan M | 2005 | 0 | http://www.ncbi.nlm.nih.gov/pubmed/15921524 | | | |
| 621 | International Orthopaedics | Katchy KC | 2005 | 0 | http://www.ncbi.nlm.nih.gov.ezproxy.aub.edu.lb/pubmed/?term=Malignant+bone+tumors+in+Kuwait%3A+a+10-year+clinicopathological+study. | | | |
| 484 | BMC Pulm Med | Alotaibi S | 2005 | 0 | http://www.ncbi.nlm.nih.gov/pubmed/15826314 | | | |
| 124 | Diabetes Care | Al Arouj M | 2005 | 0 | http://www.ncbi.nlm.nih.gov/pubmed/16123509 | | | |
| 58 | Saudi Medical Journal | Al Suwaidi J | 2005 | 0 | http://www.ncbi.nlm.nih.gov/pubmed/16228059 | | | |
| 277 | Medical Principles and Practice | Thotathil ZS | 2005 | 0 | http://www.ncbi.nlm.nih.gov/pubmed/?term=This+Cohort+study+was+undertaken+to+analyze+the+profile+of+patients+presenting+with+renal+cell+carcinoma | | | |
| 367 | Medical Principles and Practice | Al Aqeel A | 2005 | 0 | http://www.ncbi.nlm.nih.gov/pubmed/15608480 | | | |
| 57 | Inflammopharmacology | Sharma JN | 2005 | 0 | http://www.ncbi.nlm.nih.gov/pubmed/?term=The+kallikrein-kinin+system%3A+from+mediator+of+inflammation+to+modulator+of+++++++cardioprotection. | | | |
| 264 | Expert opinion on Therapeutic Targets | Novotny L | 2005 | 0 | http://www.ncbi.nlm.nih.gov/pubmed/15934920 | | | |
| 326 | Analytical and Quantitative Cytology and Histology | Das DK | 2005 | 0 | http://www.ncbi.nlm.nih.gov/pubmed/15913201 | | | |
| 344 | Saudi Medical Journal | Abdullah MA | 2005 | 0 | http://www.ncbi.nlm.nih.gov/pubmed/15983673 | | | |
| 317 | Acta Histochemica | Anim JT | 2005 | 0 | www.ncbi.nlm.nih.gov/pubmed/15950051 | | | |
| 330 | Diagnostic Cytopathology | Dey P | 2005 | 0 | http://www.ncbi.nlm.nih.gov/pubmed/15754372 | | | |
| 434 | Anticancer Research | Al-Maghrebi M | 2005 | 0 | http://www.ncbi.nlm.nih.gov/pubmed/16080495 | | | |
| 106 | J Med Liban | Bulbul M A | 2005 |  | Not Found | |  |  |
| 172 | Ethnicity & disease | El Saghir N S | 2005 |  | Not Found | |  |  |
| 180 | Ethnicity & disease | El Saghir N S | 2005 |  | Not Found | |  |  |
| 255 | La Tunisie medicale | Ghosn M | 2005 |  | Not Found | |  |  |
| 401 | International journal of gynaecology and obstetrics | Nassar A H | 2005 |  | Abstract Not Found | |  |  |
| 1038 | Clin Oncol (R Coll Radiol) | Otrock ZK | 2005 |  | http://www.ncbi.nlm.nih.gov/pubmed/16372500 | | | |
| 136 | The New Zealand medical journal | Choueiri M B | 2005 | 0 | http://www.ncbi.nlm.nih.gov/pubmed/?term=Inflammatory+breast+cancer+in+a+male+2005 | | | |
| 177 | Breast | El Saghir N S | 2005 | 0 | http://www.ncbi.nlm.nih.gov/pubmed/?term=Erysipelas+of+the+upper+extremity+following+locoregional+therapy+for+breast+cancer | | | |
| 178 | BMC cancer | El Saghir N S | 2005 | 0 | http://www.ncbi.nlm.nih.gov/pubmed/16332258 | | | |
| 287 | Skeletal radiology | Hourani M | 2005 | 0 | http://www.ncbi.nlm.nih.gov/pubmed/?term=MR+appearance+of+clear+cell+sarcoma+of+tendons+and+aponeuroses+(malignant+melanoma+of+soft+parts)%3A+radiologic-pathologic+correlation | | | |
| 330 | Journal of obstetrics and gynaecology | Khalil A M | 2005 | 0 | http://www.ncbi.nlm.nih.gov/pubmed/?term=Primary+squamous+cell+carcinoma+of+the+pelvic+retroperitoneum+presenting+as+an+adnexal+mass%3A+a+case+report | | | |
| 350 | European journal of gynaecological oncology | Makarem J A | 2005 | 0 | http://www.ncbi.nlm.nih.gov/pubmed/?term=makarem+2005+Primary+pure+squamous+cell+carcinoma+of+the+breast%3A+a+case+report+and+review+of+the+literature | | | |
| 363 | International journal of dermatology | Mourad Y A | 2005 | 0 | http://www.ncbi.nlm.nih.gov/pubmed/?term=Scrotal+ulceration+induced+by+all-trans+retinoic+acid+in+a+patient+with+acute+promyelocytic+leukemia | | | |
| 425 | Haematologica | Otrock Z K | 2005 | 0 | http://www.ncbi.nlm.nih.gov/pubmed/?term=Rituximab-induced+acute+thrombocytopenia%3A+a+report+of+two+cases | | | |
| 426 | Southern medical journal | Otrock Z K | 2005 | 0 | http://www.ncbi.nlm.nih.gov/pubmed/?term=Four+primary+tumors+of+lung%2C+bladder%2C+prostate%2C+and+breast+in+a+male+patient | | | |
| 453 | International surgery | Saghieh S | 2005 | 0 | http://www.ncbi.nlm.nih.gov/pubmed/?term=Sacral+osteoblastoma+presenting+as+a+L5-S1+disc+herniation | | | |
| 631 | Skeletal Radiology | Hourani M | 2005 | 0 | http://www.ncbi.nlm.nih.gov/pubmed/?term=MR+appearance+of+clear+cell+sarcoma+of+tendons+and+aponeuroses+(malignant+melanoma+of+soft+parts)%3A+radiologic-pathologic+correlation. | | | |
| 646 | Transplantation | Khalifeh M | 2005 | 0 | http://www.ncbi.nlm.nih.gov/pubmed/?term=Successful+living-related+liver+transplantation+for+familial+hypercholesterolemia+in+the+Middle+East. | | | |
| 659 | Eye (London, England) | Mansour AM | 2005 | 0 | http://www.ncbi.nlm.nih.gov.ezproxy.aub.edu.lb/pubmed/15094720 | | | |
| 937 | BMC Cancer | El Saghir NS | 2005 | 0 | http://www.ncbi.nlm.nih.gov/pubmed/16080790 | | | |
| 47 | The Journal of clinical endocrinology and metabolism | Al-Jenaidi F A | 2005 | 0 | http://www.ncbi.nlm.nih.gov/pubmed/?term=Contribution+of+selective+HLA-DRB1%2FDQB1+alleles+and+haplotypes+to+the+genetic+susceptibility+of+type+1+diabetes+among+Lebanese+and+Bahraini+Arabs | | | |
| 240 | International journal of radiation oncology, biology, physics | Geara F B | 2005 | 0 | http://www.ncbi.nlm.nih.gov/pubmed/?term=Nasopharyngeal+cancer+in+the+Middle+East%3A+experience+of+the+American+University+of+Beirut+Medical+Center | | | |
| 293 | World review of nutrition and dietetics | Hwalla N | 2005 | 0 | http://www.ncbi.nlm.nih.gov/pubmed/?term=Adolescent+obesity+and+physical+activity+hwalla+2005 | | | |
| 308 | Child: care, health and development | Jabre P | 2005 | 0 | http://www.ncbi.nlm.nih.gov/pubmed/?term=Overweight+children+in+Beirut%3A+prevalence+estimates+and+characteristics+jabre+2005 | | | |
| 323 | J Med Liban | Kassab R | 2005 | 0 | http://www.ncbi.nlm.nih.gov/pubmed/?term=%5BManagement+of+acute+transmural+myocardial+infarction%3A+a+study+on+200+patients+admitted+to+a+tertiary+care+medical+center%5D | | | |
| 458 | Eastern Mediterranean health journal | Salameh P | 2005 | 0 | http://www.ncbi.nlm.nih.gov/pubmed/?term=Spirometric+changes+following+the+use+of+pesticides+2005 | | | |
| 505 | Journal of thrombosis and thrombolysis | Sleiman Zade Asfahani W H | 2005 | 0 | http://www.ncbi.nlm.nih.gov/pubmed/?term=Venous+thromboembolism+in+cancer+patients+referred+to+the+American+University+of+Beirut-Medical+Center+secondary+to+deep+vein+thrombosis%3B+occurrence+and+risk+factors | | | |
| 521 | Journal of human hypertension | Tohme R A | 2005 | 0 | http://www.ncbi.nlm.nih.gov/pubmed/?term=The+prevalence+of+hypertension+and+its+association+with+other+cardiovascular+disease+risk+factors+in+a+representative+sample+of+the+Lebanese+population | | | |
| 585 | Mol Cell Biochem | Chahine R | 2005 | 0 | http://www.ncbi.nlm.nih.gov/pubmed/16311929 | | | |
| 629 | Diabetes Care | Hirbli KI | 2005 | 0 | http://www.ncbi.nlm.nih.gov/pubmed/15855610 | | | |
| 728 | J Thromb Thrombolysis | Almawi WY | 2005 | 0 | http://www.ncbi.nlm.nih.gov/pubmed/16261289 | | | |
| 967 | Atherosclerosis | Dakik HA | 2005 | 0 | http://www.ncbi.nlm.nih.gov/pubmed/15907855 | | | |
| 229 | The Journal of clinical endocrinology and metabolism | Fuleihan Gel H | 2005 | 0 | http://www.ncbi.nlm.nih.gov/pubmed/?term=Pamidronate+in+the+prevention+of+chemotherapy-induced+bone+loss+in+premenopausal+women+with+breast+cancer%3A+a+randomized+controlled+trial | | | |
| 324 | Journal of endocrinological investigation | Kassem H S | 2005 | 0 | http://www.ncbi.nlm.nih.gov/pubmed/?term=Insulin+therapy+during+Ramadan+fast+for+Type+1+diabetes+patients+kassem+2005 | | | |
| 487 | Am J of clinical oncology | Shamseddine A | 2005 | 0 | http://www.ncbi.nlm.nih.gov/pubmed/?term=A+clinical+phase+II+study+of+cisplatinum+and+vinorelbine+(PVn)+in+advanced+breast+carcinoma+(ABC) | | | |
| 493 | Food and chemical toxicology | Shihadeh A | 2005 | 0 | http://www.ncbi.nlm.nih.gov/pubmed/?term=Polycyclic+aromatic+hydrocarbons%2C+carbon+monoxide%2C+%22tar%22%2C+and+nicotine+in+the+mainstream+smoke+aerosol+of+the+narghile+water+pipe | | | |
| 954 | Am J Cardiol | Azar RR | 2005 | 0 | http://www.ncbi.nlm.nih.gov/pubmed/15642557 | | | |
| 58 | Journal of interprofessional care | Arevian M | 2005 | 0 | http://www.ncbi.nlm.nih.gov/pubmed/?term=The+significance+of+a+collaborative+practice+model+in+delivering+care+to+chronically+ill+patients%3A+a+case+study+of+managing+diabetes+mellitus+in+a+primary+health+care+center | | | |
| 335 | J Med Liban | Khauli R B | 2005 | 0 | http://www.ncbi.nlm.nih.gov/pubmed/?term=Prostate+cancer%3A+diagnostic+and+therapeutic+strategies+with+emphasis+on+the+role+of+PSA | | | |
| 233 | Cancer chemotherapy and pharmacology | Gali-Muhtasib H U | 2005 | 0 | http://www.ncbi.nlm.nih.gov/pubmed/?term=Quinoxaline+1%2C4-dioxides+induce+G2%2FM+cell+cycle+arrest+and+apoptosis+in+human+colon+cancer+cells | | | |
| 548 | Food and chemical toxicology | Shihadeh A | 2005 | 0 | http://www.ncbi.nlm.nih.gov/pubmed/15778004 | | | |
| 556 | J Hypertens | Al Jaroudi WA | 2005 | 0 | http://www.ncbi.nlm.nih.gov/pubmed/15662227 | | | |
| 640 | Endothelium | Karam CN | 2005 | 0 | http://www.ncbi.nlm.nih.gov/pubmed/?term=Effect+of+systemic+insulin+and+angiotensin+II+receptor+subtype-1+antagonist+on+endothelin-1+receptor+subtype(s)+regulation+and+binding+in+diabetic+rat+heart. | | | |
| 703 | Behav Res Methods | Shihadeh A | 2005 | 0 | http://www.ncbi.nlm.nih.gov/pubmed/16097360 | | | |
| 706 | Tobacco control | Soweid RA | 2005 | 0 | http://www.ncbi.nlm.nih.gov/pubmed/16319352 | | | |
| 729 | Radiat Res | Darwiche N | 2005 | 0 | http://www.ncbi.nlm.nih.gov/pubmed/15733037 | | | |
| 394 | Oncogene | Nasr R | 2005 | 0 | http://www.ncbi.nlm.nih.gov/pubmed/?term=Efficacy+and+mechanism+of+action+of+the+proteasome+inhibitor+PS-341+in+T-cell+lymphomas+and+HTLV-I+associated+adult+T-cell+leukemia%2Flymphoma | | | |
| 491 | Clinical pharmacokinetics | Shamseddine A I | 2005 | 0 | http://www.ncbi.nlm.nih.gov/pubmed/?term=Comparative+pharmacokinetics+and+metabolic+pathway+of+gemcitabine+during+intravenous+and+intra-arterial+delivery+in+unresectable+pancreatic+cancer+patients | | | |
| 45 | J Med Liban | Al-Geizawi S M | 2005 | 0 | http://www.ncbi.nlm.nih.gov/pubmed/16604992 | | | |
| 266 | J Med Liban | Haddad M C | 2005 | 0 | http://www.ncbi.nlm.nih.gov/pubmed/16604991 | | | |
| 736 | Sante Publique | Medlej-Hashim M | 2005 | 1 | http://www.ncbi.nlm.nih.gov/pubmed/16485442 | | | |
| 44 | J Postgrad Med | Alymlahi E | 2005 | 0 | http://www.ncbi.nlm.nih.gov/pubmed/16006708 | | | |
| 254 | Joint Bone Spine | Faik A | 2005 | 0 | http://www.ncbi.nlm.nih.gov/pubmed/15797501 | | | |
| 264 | Presse médicale | Ghfir I | 2005 | 0 | http://www.ncbi.nlm.nih.gov/pubmed/16208262 | | | |
| 270 | Presse médicale | Haddad F | 2005 | 1 | http://www.ncbi.nlm.nih.gov/pubmed/15687974 | | | |
| 372 | Chirurgie de la Main | Madhar M | 2005 | 1 | http://www.ncbi.nlm.nih.gov/pubmed/16121630 | | | |
| 951 | Revue médicale de Liège | Rouas L | 2005 | 1 | http://www.ncbi.nlm.nih.gov/pubmed/16457389 | | | |
| 952 | Annales d'Endocrinologie | Hassani R | 2005 | 1 | http://www.ncbi.nlm.nih.gov/pubmed/16392188 | | | |
| 953 | Neurochirurgie | El Malki M | 2005 | 1 | http://www.ncbi.nlm.nih.gov/pubmed/16389904 | | | |
| 956 | Presse médicale | Rouas L | 2005 | 1 | http://www.ncbi.nlm.nih.gov/pubmed/16301963 | | | |
| 957 | B-ENT | Darouassi Y | 2005 | 1 | http://www.ncbi.nlm.nih.gov/pubmed/16255499 | | | |
| 959 | Journal de radiologie | Adnani A | 2005 | 1 | http://www.ncbi.nlm.nih.gov/pubmed/16224347 | | | |
| 960 | Cancer radiothérapie | Oudidi A | 2005 | 1 | http://www.ncbi.nlm.nih.gov/pubmed/16176884 | | | |
| 961 | Annales françaises d'Oto-rhino-laryngologie et de Pathologie Cervico-faciale | Mazouzi A | 2005 | 1 | http://www.ncbi.nlm.nih.gov/pubmed/16142093 | | | |
| 963 | Revue de laryngologie - otologie - rhinologie | Sefiani S | 2005 | 1 | http://www.ncbi.nlm.nih.gov/pubmed/16080650 | | | |
| 964 | Progrès en urologie | El Mejjad A | 2005 | 1 | http://www.ncbi.nlm.nih.gov/pubmed/15999614 | | | |
| 965 | Revue de stomatologie et de chirurgie maxillo-faciale | Abada RL | 2005 | 1 | http://www.ncbi.nlm.nih.gov/pubmed/15976707 | | | |
| 966 | Journal de radiologie | Fikri M | 2005 | 1 | http://www.ncbi.nlm.nih.gov/pubmed/15959436 | | | |
| 967 | Cancer radiothérapie | BenJelloun H | 2005 | 1 | http://www.ncbi.nlm.nih.gov/pubmed/15953749 | | | |
| 969 | Journal français d'ophtalmologie | Abdelouahed K | 2005 | 1 | http://www.ncbi.nlm.nih.gov/pubmed/15851954 | | | |
| 970 | Progrès en urologie | El Mejjad A | 2005 | 1 | http://www.ncbi.nlm.nih.gov/pubmed/15822401 | | | |
| 971 | Journal de radiologie | Jroundi L | 2005 | 1 | http://www.ncbi.nlm.nih.gov/pubmed/15798629 | | | |
| 972 | Annales de Dermatologie et de Vénéréologie | Benamar L | 2005 | 1 | http://www.ncbi.nlm.nih.gov/pubmed/15798578 | | | |
| 3 | Diabetes and Metabolism | Ababou MR | 2005 | 1 | http://www.ncbi.nlm.nih.gov/pubmed/15803117 | | | |
| 15 | Clinical Rheumatology | Achemlal L | 2005 | 0 | http://www.ncbi.nlm.nih.gov/pubmed/15747054 | | | |
| 56 | Annales de Biologie Clinique | Azzouzi N | 2005 | 1 | http://www.ncbi.nlm.nih.gov/pubmed/15771975 | | | |
| 166 | Nutrition Metabolism and Cardiovasc Diseases | Cherki M | 2005 | 0 | http://www.ncbi.nlm.nih.gov/pubmed/16216721 | | | |
| 196 | European Journal of Clinical Nutrition | El Aayachi M | 2005 | 0 | http://www.ncbi.nlm.nih.gov/pubmed/16118656 | | | |
| 338 | Saudi Journal of Kidney Disease and Transplantation | Khanfri N | 2005 | 0 | http://www.ncbi.nlm.nih.gov/pubmed/18209464 | | | |
| 369 | Clin Exp Hypertens | Lyoussi B | 2005 | 0 | http://www.ncbi.nlm.nih.gov/pubmed/15921074 | | | |
| 945 | Eastern Mediterranean Health Journal | Kjiri S | 2005 | 1 | http://www.ncbi.nlm.nih.gov/pubmed/16602462 | | | |
| 955 | Revue de laryngologie - otologie - rhinologie | Ezzoubi M | 2005 | 1 | http://www.ncbi.nlm.nih.gov/pubmed/16366379 | | | |
| 958 | Annales de Cardiologie et d'Angéiologie | Bendahmane S | 2005 | 1 | http://www.ncbi.nlm.nih.gov/pubmed/16237916 | | | |
| 962 | Progrès en urologie | Mbarki M | 2005 | 1 | http://www.ncbi.nlm.nih.gov/pubmed/16097146 | | | |
| 976 | Bulletin du Cancer | Madani A | 2005 | 1 | http://www.ncbi.nlm.nih.gov/pubmed/15749649 | | | |
| 60 | Monaldi Arch Chest Dis | Bartal M | 2005 | 0 | http://www.ncbi.nlm.nih.gov/pubmed/16454221 | | | |
| 148 | Transactions of the Royal Society of Tropical Medicine and Hygiene | Boutayeb A | 2005 | 0 | http://www.ncbi.nlm.nih.gov/pubmed/16274715 | | | |
| 291 | Pathology | Hbid O | 2005 | 0 | http://www.ncbi.nlm.nih.gov/pubmed/16194827 | | | |
| 297 | Pediatr Blood Cancer | Hessissen L | 2005 | 0 | http://www.ncbi.nlm.nih.gov/pubmed/15547930 | | | |
| 376 | Phytotherapy Research | Maghrani M | 2005 | 0 | http://www.ncbi.nlm.nih.gov/pubmed/15852497 | | | |
| 459 | Cancer Genet Cytogenet | Rodriguez S | 2005 | 0 | http://www.ncbi.nlm.nih.gov/pubmed/15721635 | | | |
| 706 | Journal of Ethnopharmacology | Eddouks M | 2005 | 0 | http://www.ncbi.nlm.nih.gov/pubmed/15848019 | | | |
| 707 | Journal of Ethnopharmacology | Eddouks M | 2005 | 0 | http://www.ncbi.nlm.nih.gov/pubmed/15707780 | | | |
| 968 | Neoplasma | Aboudkhil S | 2005 | 1 | http://www.ncbi.nlm.nih.gov/pubmed/15875090 | | | |
| 176 | Annals of Nutrition and Metabolism | Derouiche A | 2005 | 0 | http://www.ncbi.nlm.nih.gov/pubmed/16020940 | | | |
| 188 | Journal of Ethnopharmacology | Eddouks M | 2005 | 0 | http://www.ncbi.nlm.nih.gov/pubmed/15814271 | | | |
| 189 | Diabetes Research and Clinical Practice | Eddouks M | 2005 | 0 | http://www.ncbi.nlm.nih.gov/pubmed/15713350 | | | |
| 193 | Journal of Ethnopharmacology | Eddouks M | 2005 | 0 | http://www.ncbi.nlm.nih.gov/pubmed/16099613 | | | |
| 55 | Saudi Med J | Sweileh WM | 2005 | 0 | http://www.ncbi.nlm.nih.gov/pubmed/16311686 | | | |
| 57 | Saudi Med J | Sweileh WM | 2005 | 0 | http://www.ncbi.nlm.nih.gov/pubmed/15900357 | | | |
| 58 | Saudi Med J | Sweileh WM | 2005 | 0 | http://www.ncbi.nlm.nih.gov/pubmed/15756358 | | | |
| 56 | Asian Pacific journal of cancer prevention | Abu-Rabia A | 2005 | 0 | http://www.ncbi.nlm.nih.gov/pubmed/16236008 | | | |
| 32 | Saudi medical journal | Alla A H | 2005 | 0 | http://www.ncbi.nlm.nih.gov/pubmed/?term=A+large+giant+cell+tumor+of+the+sacrum.+Advantage+of+an+abdomino-sacral+approach | | | |
| 53 | Indian pediatrics | Elamin A | 2005 | 0 | http://www.ncbi.nlm.nih.gov/pubmed/15695853 | | | |
| 64 | Diabetes research and clinical practice | Elrayah H | 2005 | 0 | http://www.ncbi.nlm.nih.gov/pubmed/?term=Economic+burden+on+families+of+childhood+type+1+diabetes+in+urban+Sudan | | | |
| 73 | Journal of family and community medicine | Hussein S E | 2005 | 0 | http://www.ncbi.nlm.nih.gov/pubmed/?term=The+pattern+of+commoner+health+problems+among+basic+school+children%2C+gezira+state%2C+Sudan | | | |
| 74 | Journal of family and community medicine | Hussein S E | 2005 | 0 | http://www.ncbi.nlm.nih.gov/pubmed/?term=Prevalence+and+risk+factors+of+asthma+among+wad+medani+basic+school+children%2C+gezira+state%2C+Sudan | | | |
| 4 | Diabetic medicine | Abdelgadir M | 2005 | 0 | https://www.ncbi.nlm.nih.gov.ezproxy.aub.edu.lb/pubmed/?term=Glycaemic+and+insulin+responses+of+six+traditional+Sudanese+carbohydrate-rich+meals+in+subjects+with+Type+2+diabetes+mellitus | | | |
| 82 | Saudi medical journal | Khairy G A | 2005 | 0 | http://www.ncbi.nlm.nih.gov/pubmed/15900371 | | | |
| 25 | Saudi medical journal | Ahmed M A | 2005 | 0 | http://www.ncbi.nlm.nih.gov/pubmed/?term=Evaluation+of+palliative+management+of+advanced+breast+cancer+in+Khartoum%2C+Sudan | | | |
| 146 | Acta Pharmacological Sinica | Kamal M | 2006 | 0 | http://www.ncbi.nlm.nih.gov/pubmed/?term=Effect+of+nicotinamide+on+newly+diagnosed+type+1+diabetic+children. | | | |
| 149 | Pharmacoepidemiology Drug Safety | Al Khaja KA | 2006 | 0 | http://www.ncbi.nlm.nih.gov/pubmed/16342299 | | | |
| 135 | Eastern Mediterranean health journal | Awadallah MS | 2006 | 0 | http://www.ncbi.nlm.nih.gov/pubmed/17333812 | | | |
| 137 | Journal of Renin-Angiotensin-Aldosterone System | Kassab S | 2006 | 0 | http://www.ncbi.nlm.nih.gov/pubmed/?term=he+angiotensin+type+1+receptor+antagonist+valsartan+attenuates+pathological+ventricular+hypertrophy+induced+by+hyperhomocysteinemia+in+rats. | | | |
| 145 | Sultan Qaboos University Medical Journal | Kassab S | 2006 | 0 | http://www.ncbi.nlm.nih.gov/pubmed/?term=Cardiovascular+Responses+to+Tonic+Pain+in+REM+Sleep-Deprived+Rats%3A+Role+of+Melatonin+and+Beta+Endorphin. | | | |
| 143 | Clinical and Vaccine Immunology | Almawi WY | 2006 | 0 | http://www.ncbi.nlm.nih.gov/pubmed/16988007 | | | |
| 142 | Annals of Saudi Medicine | Al-Hamdan N | 2006 | 0 | http://www.ncbi.nlm.nih.gov/pubmed/17143018 | | | |
| 147 | Saudi Medical Journal | Alnasir FA | 2006 | 0 | http://www.ncbi.nlm.nih.gov/pubmed/16680261 | | | |
| 137 | Annals of Saudi Medicine | Afifi M | 2006 |  | http://www.ncbi.nlm.nih.gov/pubmed/16861865 | | | |
| 138 | Annals of Saudi Medicine | Lafta RK | 2006 |  | http://www.ncbi.nlm.nih.gov/pubmed/?term=Reply+to+Re%3A+Childhood+obesity+in+Iraq%3A+a+gender+perspective. | | | |
| 139 | Journal of Investigative Dermatology | Emmert S | 2006 |  | http://www.ncbi.nlm.nih.gov/pubmed/?term=Relationship+between+waist+circumference+and+blood+pressure+among+the+population+in+Baghdad%2C+Iraq. | | | |
| 209 | Journal of Palliative Medicine | Menkin ES | 2006 |  | http://www.ncbi.nlm.nih.gov/pubmed/?term=Iraq+of+the+palliative+care+movement. | | | |
| 133 | Saudi Journal of Kidney Disease and Transplantation | Altaee IK | 2006 | 0 | http://www.ncbi.nlm.nih.gov/pubmed/?term=Incidence+and+types+of+malignancies+in+renal+transplant+recipients+in+Iraq. | | | |
| 140 | MMWR Morbidity and Mortality Weekly Report | Centers for Disease Control and Prevention (CDC) | 2006 | 0 | http://www.ncbi.nlm.nih.gov/pubmed/?term=Tobacco+use+among+students+aged+13-15+years--Kurdistan+Region%2C+Iraq%2C+2005. | | | |
| 143 | Saudi Medical Journal | Subhi MD | 2006 | 0 | http://www.ncbi.nlm.nih.gov/pubmed/?term=Blood+pressure+profiles+and+hypertension+in+Iraqi+primary+school+children. | | | |
| 263 | European Journal of Clinical Nutrition | Al-Tamer YY | 2006 | 0 | http://www.ncbi.nlm.nih.gov/pubmed/16775580 | | | |
| 150 | Life Science Journal | Al-Azzawie HF | 2006 | 0 | http://www.ncbi.nlm.nih.gov/pubmed/?term=Hypoglycemic+and+antioxidant+effect+of+oleuropein+in+alloxan-diabetic+rabbits. | | | |
| 152 | Archives of Oral Biology | Al-Azzawi LM | 2006 | 0 | http://www.ncbi.nlm.nih.gov/pubmed/16055079 | | | |
| 135 | Journal of Pineal Research | Kadhim HM | 2006 | 0 | http://www.ncbi.nlm.nih.gov/pubmed/?term=ffects+of+melatonin+and+zinc+on+lipid+profile+and+renal+function+in+type+2+diabetic+patients+poorly+controlled+with+metformin. | | | |
| 134 | Saudi Medical Journal | Zangana AM | 2006 | 0 | http://www.ncbi.nlm.nih.gov/pubmed/?term=Satellite+implantation+of+the+skin+with+malignant+melanoma. | | | |
| 127 | J Med Liban | Kadhim HS | 2006 | 0 | http://www.ncbi.nlm.nih.gov/pubmed/?term=Possible+role+of+nuclear+factor+kappaB+detected+by+in+situ+hybridization+in+the+pathogenesis+of+transitional+cell+carcinoma+of+the+bladder. | | | |
| 131 | Eastern Mediterranean Health Journal | Fakri S | 2006 | 0 | http://www.ncbi.nlm.nih.gov/pubmed/?term=Antiperspirant+use+as+a+risk+factor+for+breast+cancer+in+Iraq. | | | |
| 132 | Saudi Medical Journal | Hussain SA | 2006 | 0 | http://www.ncbi.nlm.nih.gov/pubmed/17013468 | | | |
| 145 | Saudi Medical Journal | Al-Maroof RA | 2006 | 0 | http://www.ncbi.nlm.nih.gov/pubmed/?term=Serum+zinc+levels+in+diabetic+patients+and+effect+of+zinc+supplementation+on+glycemic+control+of+type+2+diabetics. | | | |
| 141 | Journal of Craniofacial Surgery | Kummoona R | 2006 | 0 | http://www.ncbi.nlm.nih.gov/pubmed/?term=Apoptotic+changes+of+Middle+East+jaw+lymphoma. | | | |
| 103 | Saudi Medical Journal | Al-Dabbagh TQ | 2006 |  | http://www.ncbi.nlm.nih.gov/pubmed/16758073 | | | |
| 671 | Saudi Medical Journal | Nahar IK | 2006 |  | http://www.ncbi.nlm.nih.gov/pubmed/?term=The+use+of+etoricoxib+in+patients+with+bronchial+asthma+associated+with+aspirin+sensitivity. | | | |
| 399 | Endocrine Pathology | Al-Brahim N | 2006 | 0 | http://www.ncbi.nlm.nih.gov/pubmed/17917001 | | | |
| 404 | Obesity Surgery | Al-Fahad T | 2006 | 0 | http://www.ncbi.nlm.nih.gov/pubmed/16687041 | | | |
| 474 | World journal of Surgical Oncology | Aldahham A | 2006 | 0 | http://www.ncbi.nlm.nih.gov/pubmed/17026774 | | | |
| 477 | East African Medical Journal | Ali Y | 2006 | 0 | http://www.ncbi.nlm.nih.gov/pubmed/17455454 | | | |
| 553 | Military Medicine | Donovan DJ | 2006 | 0 | http://www.ncbi.nlm.nih.gov/pubmed/16602518 | | | |
| 582 | British Journal of Haematology | Gupta A | 2006 | 0 | http://www.ncbi.nlm.nih.gov.ezproxy.aub.edu.lb/pubmed/16412013 | | | |
| 905 | European Journal of Epidemiology | Zubaid M | 2006 | 0 | http://www.ncbi.nlm.nih.gov/pubmed/16547833 | | | |
| 49 | Medical Principles and Practice | Al-Mutairi N | 2006 | 0 | http://www.ncbi.nlm.nih.gov/pubmed/?term=Cutaneous+manifestations+of+diabetes+mellitus.+Study+from+Farwaniya+hospital%2C+++++++Kuwait | | | |
| 106 | Pediatric Diabetes | Abdul-Rasoul M | 2006 | 0 | http://www.ncbi.nlm.nih.gov/pubmed/?term='The+honeymoon+phase'+in+children+with+type+1+diabetes+mellitus%3A+frequency%2C+duration%2C+and+influential+factors | | | |
| 376 | Respirology | Al Mutairi SS | 2006 | 0 | http://www.ncbi.nlm.nih.gov/pubmed/16771915 | | | |
| 610 | Acta Cytologica | Joqai S | 2006 | 0 | http://www.ncbi.nlm.nih.gov.ezproxy.aub.edu.lb/pubmed/?term=Fine+needle+aspiration+cytology+of+Hodgkin's+lymphoma%3A+A+cytohistologic+correlation+study+from+a+cancer+center+in+Kuwait. | | | |
| 612 | Interactive cardiovascular and thoracic surgery | K Ayed A | 2006 | 0 | http://www.ncbi.nlm.nih.gov.ezproxy.aub.edu.lb/pubmed/?term=Prognostic+significance+of+cyclin+D1+expression+in+resected+stage+I%2C+II+non-small+cell+lung+cancer+in+Arabs. | | | |
| 619 | Medical Principles and Practice | Kapila K | 2006 | 0 | http://www.ncbi.nlm.nih.gov.ezproxy.aub.edu.lb/pubmed/?term=Changing+spectrum+of+squamous+cell+abnormalities+observed+on+papanicolaou+smears+in+Mubarak+Al-Kabeer+Hospital%2C+Kuwait%2C+over+a+13-year+period. | | | |
| 624 | International Journal of Urology | Kehinde EO | 2006 | 0 | http://www.ncbi.nlm.nih.gov.ezproxy.aub.edu.lb/pubmed/?term=Do+differences+in+age+specific+androgenic+steroid+hormone+levels+account+for+differing+prostate+cancer+rates+between+Arabs+and+Caucasians%3F | | | |
| 625 | International Urology and Nephrology | Kehinde EO | 2006 | 0 | http://www.ncbi.nlm.nih.gov.ezproxy.aub.edu.lb/pubmed/?term=Prostate+cancer+risk%3A+the+significance+of+differences+in+age+related+changes+in+serum+conjugated+and+unconjugated+steroid+hormone+concentrations+between+Arab+and+Caucasian+men. | | | |
| 651 | southern medical journal | Marouf R | 2006 | 0 | http://www.ncbi.nlm.nih.gov.ezproxy.aub.edu.lb//pubmed/16929874 | | | |
| 690 | The Journal of Asthma | Owayed A | 2006 | 0 | http://www.ncbi.nlm.nih.gov/pubmed/?term=Proficiency+of+pediatricians+in+the+use+of+inhaled+medication+delivery+systems+for+the+management+of+asthma | | | |
| 750 | Medical Principles and Practice | Jadaon MM | 2006 | 0 | http://www.ncbi.nlm.nih.gov/pubmed/16484835 | | | |
| 897 | American Journal of Reproductive Immunology | Mahmoud FF | 2006 | 0 | http://www.ncbi.nlm.nih.gov/pubmed/16911714 | | | |
| 904 | Acta Cardiologica | Zubaid M | 2006 | 0 | http://www.ncbi.nlm.nih.gov/pubmed/16970053 | | | |
| 146 | Journal of Cardiothoracic and Vascular Anesthesia | Al-Shawaf E | 2006 | 0 | http://www.ncbi.nlm.nih.gov/pubmed/16750735 | | | |
| 53 | Journal of Human Hypertension | Doi SA | 2006 | 0 | http://www.ncbi.nlm.nih.gov/pubmed/?term=Optimal+use+and+interpretation+of+the+aldosterone+renin+ratio+to+detect+++++++aldosterone+excess+in+hypertension | | | |
| 54 | Archives of Medical Research | Sharma JN | 2006 | 0 | http://www.ncbi.nlm.nih.gov/pubmed/?term=Role+of+tissue+kallikrein-kininogen-kinin+pathways+in+the+cardiovascular+system | | | |
| 485 | Internet Journal of Pediatrics and Neonatology | Alotaibi S | 2006 | 0 | http://www.ncbi.nlm.nih.gov/pubmed/21666762 | | | |
| 531 | Diagnostic Cytopathology | Das DK | 2006 | 0 | http://www.ncbi.nlm.nih.gov/pubmed/16604559 | | | |
| 548 | Cytopathology | Dey P | 2006 | 0 | http://www.ncbi.nlm.nih.gov/pubmed/16961657 | | | |
| 717 | The Scientific World Journal | Sharma JN | 2006 | 0 | http://www.ncbi.nlm.nih.gov.ezproxy.aub.edu.lb/pubmed/17041716 | | | |
| 884 | J Hum Hypertens | Doi SA | 2006 | 0 | http://www.ncbi.nlm.nih.gov/pubmed/16617310 | | | |
| 51 | Methods and Findings in experimental and clinical pharmacology | Sharma JN | 2006 | 0 | http://www.ncbi.nlm.nih.gov/pubmed/?term=Effect+of+captopril+in+the+presence+of+kinin+B2+receptor+antagonist+on+duration+++++++of+survival+after+prolonged+coronary+artery+ligation+in+hypertensive+rats. | | | |
| 55 | Journal of Nutrition | Al-Qattan KK | 2006 | 0 | http://www.ncbi.nlm.nih.gov/pubmed/?term=Nitric+oxide+mediates+the+blood-pressure+lowering+effect+of+garlic+in+the+rat+++++++two-kidney%2C+one-clip+model+of+hypertension. | | | |
| 56 | Heart and Circulatory Physiology: American Journal of Physiology | Benter IF | 2006 | 0 | http://www.ncbi.nlm.nih.gov/pubmed/16403946 | | | |
| 121 | British Journal of Nutrition | Al-Amin ZM | 2006 | 0 | http://www.ncbi.nlm.nih.gov/pubmed/?term=Anti-diabetic+and+hypolipidaemic+properties+of+ginger+(Zingiber+officinale)+in+streptozotocin-induced+diabetic+rats | | | |
| 202 | Medicina | Renno WM | 2006 | 0 | http://www.ncbi.nlm.nih.gov/pubmed/?term=Talin+immunogold+density+increases+in+sciatic+nerve+of+diabetic+rats+after+nerve+growth+factor+treatment | | | |
| 216 | Cell Biochemistry and Function | Yousif MH | 2006 | 0 | http://www.ncbi.nlm.nih.gov/pubmed/?term=Signal+transduction+through+Ras-GTPase+and+Ca2%2B%2F+calmodulin-dependent+protein+kinase+II+contributes+to+development+of+diabetes-induced+renal+vascular+dysfunction | | | |
| 217 | Cell Biochemistry and Function | Yousif MH | 2006 | 0 | http://www.ncbi.nlm.nih.gov/pubmed/?term=Role+of+protein+kinases+in+mediating+diabetes-induced+augmented+vasoconstriction+to+endothelin-1+in+the+renal+arteries+of+STZ-diabetic+rats | | | |
| 224 | Cell Biochemistry and Function | Yousif MH | 2006 | 0 | http://www.ncbi.nlm.nih.gov/pubmed/?term=Phosphoinositide+3-kinase+mediated+signalling+contributes+to+development+of+diabetes-induced+abnormal+vascular+reactivity+of+rat+carotid+artery | | | |
| 402 | Free Radical Biology and Medicine | Al-Enezi KS | 2006 | 0 | http://www.ncbi.nlm.nih.gov/pubmed/16545681 | | | |
| 442 | Journal of Clinical Oncology | Al-Mulla F | 2006 | 0 | http://www.ncbi.nlm.nih.gov/pubmed/17179102 | | | |
| 479 | Free Radical Biology and Medicine | Alkhalaf M | 2006 | 0 | http://www.ncbi.nlm.nih.gov/pubmed/16814113 | | | |
| 496 | International Urology and Nephrology | Anim JT | 2006 | 0 | http://www.ncbi.nlm.nih.gov/pubmed/16502049 | | | |
| 547 | Cytojournal | Dey P | 2006 | 0 | http://www.ncbi.nlm.nih.gov/pubmed/17069647 | | | |
| 609 | Acta Cytologica | Joqai S | 2006 | 0 | http://www.ncbi.nlm.nih.gov.ezproxy.aub.edu.lb/pubmed/?term=Role+of+fine+needle+aspiration+cytology+in+nodular+sclerosis+variant+of+Hodgkin's+lymphoma. | | | |
| 636 | Histochemistry and Cell Biology | Krajci D | 2006 | 0 | http://www.ncbi.nlm.nih.gov.ezproxy.aub.edu.lb/pubmed/?term=Intranuclear+microtubules+are+hallmarks+of+an+unusual+form+of+cell+death+in+cisplatin-treated+C6+glioma+cells. | | | |
| 682 | Experimental Oncology | Novotny L | 2006 | 0 | http://www.ncbi.nlm.nih.gov.ezproxy.aub.edu.lb/pubmed/?term=Antileukemic+activity+of+sulfonamide+conjugates+of+arabinosylcytosine. | | | |
| 718 | Inflammopharmacology | Sharma JN | 2006 | 0 | http://www.ncbi.nlm.nih.gov.ezproxy.aub.edu.lb/pubmed/?term=The+role+of+leukotrienes+in+the+pathophysiology+of+inflammatory+disorders%3A+is+there+a+case+for+revisiting+leukotrienes+as+therapeutic+targets%3F | | | |
| 730 | Anatomia, Histologia, Embryologia | Temmim L | 2006 | 0 | http://www.ncbi.nlm.nih.gov.ezproxy.aub.edu.lb/pubmed/16542178 | | | |
| 735 | The Journal of Nutrition | Thomson M | 2006 | 0 | http://www.ncbi.nlm.nih.gov.ezproxy.aub.edu.lb/pubmed/?term=Including+garlic+in+the+diet+may+help+lower+blood+glucose%2C+cholesterol%2C+and+triglycerides | | | |
| 419 | The Journal of the Royal Society for the Promotion of Health | Al-Isa AN | 2006 | 0 | http://www.ncbi.nlm.nih.gov/pubmed/16478016 | | | |
| 500 | J Natl Med Assoc | Awadalla AW | 2006 | 0 | http://www.ncbi.nlm.nih.gov/pubmed/16749648 | | | |
| 501 | J Natl Med Assoc | Awadalla AW | 2006 | 0 | http://www.ncbi.nlm.nih.gov/pubmed/16749649 | | | |
| 509 | International Journal of Oral and Maxillofacial Surgery | Behbehani F | 2006 | 0 | http://www.ncbi.nlm.nih.gov/pubmed/16503397 | | | |
| 510 | International Journal of Tuberculosis and Lung Disease | Behbehani N | 2006 | 0 | http://www.ncbi.nlm.nih.gov/pubmed/16602397 | | | |
| 446 | European Journal of Clinical Microbiology and Infectious Diseases | Al-Obaid I | 2006 | 0 | http://www.ncbi.nlm.nih.gov/pubmed/17033790 | | | |
| 52 | Saudi Medical Journal | Zubaid M | 2006 | 0 | http://www.ncbi.nlm.nih.gov/pubmed/?term=Troponin+estimation+identifies+myocardial+infa7ion+patients+with+different+++++++characteristics | | | |
| 145 | Journal of International academy of Periodontology | Al-Shammari KF | 2006 | 0 | http://www.ncbi.nlm.nih.gov/pubmed/?term=Association+of+periodontal+disease+severity+with+diabetes+duration+and+diabetic+complications+in+patients+with+type+1+diabetes+mellitus | | | |
| 410 | Annals of Saudi Medicine | Al-Hamdan N | 2006 | 0 | http://www.ncbi.nlm.nih.gov/pubmed/17143018 | | | |
| 427 | Obesity Reviews | Al-Kandari YY | 2006 | 0 | http://www.ncbi.nlm.nih.gov/pubmed/16629871 | | | |
| 440 | Modern Pathology | Al-Mulla F | 2006 | 0 | http://www.ncbi.nlm.nih.gov/pubmed/16528379 | | | |
| 544 | Molecular and Cellular Biochemistry | Dashti HM | 2006 | 0 | http://www.ncbi.nlm.nih.gov/pubmed/16652223 | | | |
| 261 | Cancer Letters | Novotny L | 2006 | 0 | http://www.ncbi.nlm.nih.gov/pubmed/15885888 | | | |
| 441 | Journal of Clinical Pathology | Al-Mulla F | 2006 | 0 | http://www.ncbi.nlm.nih.gov/pubmed/16731603 | | | |
| 443 | Free Radical Research | Al-Mutairi DA | 2006 | 0 | http://www.ncbi.nlm.nih.gov/pubmed/16551574 | | | |
| 528 | Neoplasma | Cojocel C | 2006 | 0 | http://www.ncbi.nlm.nih.gov/pubmed/16830059 | | | |
| 185 | Diabetic Medicine | Mojiminiyi OA | 2006 | 0 | http://www.ncbi.nlm.nih.gov.ezproxy.aub.edu.lb/pubmed/?term=Prevalence+and+associations+of+low+plasma+erythropoietin+in+patients+with+Type+2+diabetes+mellitus | | | |
| 102 | The New England journal of medicine | Boukhalil P | 2006 |  | Not Found | |  |  |
| 519 | Gut | Tohme C | 2006 |  | Abstract Not Found | |  |  |
| 208 | Eur J Pediatr Surg | Faraj W | 2006 | 0 | http://www.ncbi.nlm.nih.gov/pubmed/?term=Solid+pseudopapillary+neoplasm+of+the+pancreas+in+a+12-year-old+female%3A+case+report+and+review+of+the+literature | | | |
| 212 | Lung cancer | Fares M D | 2006 | 0 | http://www.ncbi.nlm.nih.gov/pubmed/16959369 | | | |
| 407 | Journal of hepato-biliary-pancreatic surgery | Noun R | 2006 | 0 | http://www.ncbi.nlm.nih.gov/pubmed/?term=Extracystic+biliary+carcinoma+associated+with+anomalous+pancreaticobiliary+junction+and+cysts | | | |
| 411 | Obesity surgery | Noun R | 2006 | 0 | http://www.ncbi.nlm.nih.gov/pubmed/?term=Laparoscopic+latero-lateral+jejuno-jejunostomy+as+a+rescue+procedure+after+complicated+mini-gastric+bypass+noun+2006 | | | |
| 424 | Am J of clinical oncology | Otrock Z K | 2006 | 0 | http://www.ncbi.nlm.nih.gov/pubmed/?term=Should+we+screen+patients+for+inherited+thrombophilia+before+starting+thalidomide%3F | | | |
| 427 | Am J of hematology | Otrock Z K | 2006 | 0 | http://www.ncbi.nlm.nih.gov/pubmed/?term=Non-Hodgkin+disease+in+beta-thalassemia+major+otrock | | | |
| 478 | Neuro endocrinology letters | Seif F E | 2006 | 0 | http://www.ncbi.nlm.nih.gov/pubmed/?term=seif++++2006+Hypercalcemia+in+glioblastoma+multiforme | | | |
| 552 | International journal of cardiology | Abdallah MH | 2006 | 0 | http://www.ncbi.nlm.nih.gov/pubmed/16321706 | | | |
| 566 | Obstet Gynecol | Atallah D | 2006 | 0 | http://www.ncbi.nlm.nih.gov/pubmed/17018494 | | | |
| 586 | Acta Neurochir (Wien) | Chamoun RB | 2006 | 0 | http://www.ncbi.nlm.nih.gov/pubmed/16374565 | | | |
| 606 | European Journal of Pediatric Surgery | Faraj W | 2006 | 0 | http://www.ncbi.nlm.nih.gov/pubmed/?term=Solid+pseudopapillary+neoplasm+of+the+pancreas+in+a+12-year-old+female%3A+case+report+and+review+of+the+literature. | | | |
| 649 | Nephrology, dialysis, transplantation | Khoriaty R | 2006 | 0 | http://www.ncbi.nlm.nih.gov.ezproxy.aub.edu.lb/pubmed/16968731 | | | |
| 936 | Anti-cancer drugs | El-Saghir NS | 2006 | 0 | http://www.ncbi.nlm.nih.gov/pubmed/16940812 | | | |
| 949 | Can J Cardiol | Abdallah MH | 2006 | 0 | http://www.ncbi.nlm.nih.gov/pubmed/16802002 | | | |
| 984 | Colorectal Dis | Hatoum HA | 2006 | 0 | http://www.ncbi.nlm.nih.gov/pubmed/16784482 | | | |
| 992 | J Thromb Thrombolysis | Isma'eel H | 2006 | 0 | http://www.ncbi.nlm.nih.gov/pubmed/16622615 | | | |
| 1034 | International journal of gynecological cancer | Otrock ZK | 2006 | 0 | http://www.ncbi.nlm.nih.gov/pubmed/17009994 | | | |
| 1035 | Leuk Lymphoma | Otrock ZK | 2006 | 0 | http://www.ncbi.nlm.nih.gov/pubmed/16840224 | | | |
| 1036 | Am J Hematol | Otrock ZK | 2006 | 0 | http://www.ncbi.nlm.nih.gov/pubmed/16493602 | | | |
| 1037 | Am J Hematol | Otrock ZK | 2006 | 0 | http://www.ncbi.nlm.nih.gov/pubmed/16432854 | | | |
| 12 | Thrombosis research | Abchee A | 2006 | 0 | http://www.ncbi.nlm.nih.gov/pubmed/15985286 | | | |
| 62 | Journal of transcultural nursing | Arevian M | 2006 | 0 | http://www.ncbi.nlm.nih.gov/pubmed/?term=Raising+awareness+and+providing+free+screening+improves+cervical+cancer+screening+among+economically+disadvantaged+Lebanese%2FArmenian+women | | | |
| 82 | Hypertension | Bahous S A | 2006 | 0 | http://www.ncbi.nlm.nih.gov/pubmed/?term=Aortic+stiffness%2C+living+donors%2C+and+renal+transplantation+2006 | | | |
| 111 | European journal of public health | Chakar H | 2006 | 0 | http://www.ncbi.nlm.nih.gov/pubmed/16698887 | | | |
| 179 | BMC Cancer | El Saghir N S | 2006 | 0 | http://www.ncbi.nlm.nih.gov/pubmed/16857060 | | | |
| 182 | Photodermatology, photoimmunology & photomedicine | El Sayed F | 2006 | 0 | http://www.ncbi.nlm.nih.gov/pubmed/16436177 | | | |
| 336 | Revue des maladies respiratoires | Khayat G | 2006 | 0 | http://www.ncbi.nlm.nih.gov/pubmed/?term=Organization+and+results+of+an+information+campaign+and+early+diagnostic+in+Lebanon | | | |
| 415 | Journal of transcultural nursing | Noureddine S | 2006 | 0 | http://www.ncbi.nlm.nih.gov/pubmed/?term=noureddine+Delay+in+seeking+health+care+for+acute+coronary+syndromes+in+a+Lebanese+sample+2006 | | | |
| 459 | Journal of epidemiology and community health | Salameh P | 2006 | 0 | http://www.ncbi.nlm.nih.gov/pubmed/16476757 | | | |
| 461 | European journal of epidemiology | Salameh P R | 2006 | 0 | http://www.ncbi.nlm.nih.gov/pubmed/?term=Chronic+bronchitis+and+pesticide+exposure%3A+a+case-control+study+in+Lebanon | | | |
| 525 | J Med Liban | Waked M | 2006 | 0 | http://www.ncbi.nlm.nih.gov/pubmed/?term=Asthma%2C+allergic+rhinitis+and+eczema+in+13-14-year-old+schoolchildren+across+Lebanon | | | |
| 530 | World journal of gastroenterology | Yaghi C | 2006 | 0 | http://www.ncbi.nlm.nih.gov/pubmed/?term=Hepatocellular+carcinoma+in+Lebanon%3A+Etiology+and+prognostic+factors+associated+with+short-term+survival | | | |
| 568 | Clinical endocrinology | Azar RR | 2006 | 0 | http://www.ncbi.nlm.nih.gov/pubmed/17121520 | | | |
| 658 | Eye (London, England) | Mansour AM | 2006 | 0 | http://www.ncbi.nlm.nih.gov.ezproxy.aub.edu.lb/pubmed/16021195 | | | |
| 720 | J Fr Ophtalmol | Waked N | 2006 | 1 | http://www.ncbi.nlm.nih.gov/pubmed/16557173 | | | |
| 724 | Retina | Zein WM | 2006 | 0 | http://www.ncbi.nlm.nih.gov/pubmed/16467667 | | | |
| 1048 | European journal of public health | Tamim H | 2006 | 0 | http://www.ncbi.nlm.nih.gov/pubmed/16675481 | | | |
| 1050 | Int J Eat Disord | Tamim H | 2006 | 0 | http://www.ncbi.nlm.nih.gov/pubmed/16231340 | | | |
| 253 | Anticancer research | Ghosn M | 2006 | 0 | http://www.ncbi.nlm.nih.gov/pubmed/?term=Phase+II+trial+of+capecitabine+and+vinorelbine+as+first-line+chemotherapy+for+metastatic+breast+cancer+patients+ghosn+2006 | | | |
| 492 | Oncology | Shamseddine A I | 2006 | 0 | http://www.ncbi.nlm.nih.gov/pubmed/?term=a++clinical+phase+II+study+of+a+non-anthracycline+sequential+combination+of+cisplatin-vinorelbine+followed+by+docetaxel+as+first-line+treatment+in+metastatic+breast+cancer | | | |
| 953 | Am Heart J | Azar RR | 2006 | 0 | http://www.ncbi.nlm.nih.gov/pubmed/16442924 | | | |
| 10 | Current hematology reports | Abboud M R | 2006 | 0 | http://www.ncbi.nlm.nih.gov/pubmed/?term=Prevention+and+management+of+strokes+in+patients+with+sickle+cell+disease+Abboud+2006 | | | |
| 14 | International journal of cardiology | Abdallah M H | 2006 | 0 | http://www.ncbi.nlm.nih.gov/pubmed/?term=The+management+of+acute+myocardial+infarction+in+developing+countries+Abdallah+2006 | | | |
| 232 | The international journal of biochemistry & cell biology | Gali-Muhtasib H | 2006 | 0 | http://www.ncbi.nlm.nih.gov/pubmed/?term=Thymoquinone%3A+a+promising+anti-cancer+drug+from+natural+sources | | | |
| 360 | Am J of roentgenology | Mehanna M J | 2006 | 0 | http://www.ncbi.nlm.nih.gov/pubmed/?term=Complications+of+adjustable+gastric+banding%2C+a+radiological+pictorial+review | | | |
| 612 | Current Opinion in Pediatrics | Ghanem I | 2006 | 0 | http://www.ncbi.nlm.nih.gov/pubmed/?term=The+management+of+osteoid+osteoma%3A+updates+and+controversies. | | | |
| 279 | Leukemia research | Harakeh S | 2006 | 0 | http://www.ncbi.nlm.nih.gov/pubmed/?term=Inhibition+of+proliferation+and+induction+of+apoptosis+by+2-benzoyl-3-phenyl-6%2C7-dichloroquinoxaline+1%2C4-dioxide+in+adult+T-cell+leukemia+cells | | | |
| 555 | Lancet Oncol | Ahmad K | 2006 | 0 | http://www.ncbi.nlm.nih.gov/pubmed/16977728 | | | |
| 591 | J Toxicol Environ Health A | Daher CF | 2006 | 0 | http://www.ncbi.nlm.nih.gov/pubmed/16728375 | | | |
| 601 | Immunopharmacology and Immunotoxicology | El-Haibi C | 2006 | 0 | http://www.ncbi.nlm.nih.gov/pubmed/?term=Effect+of+atorvastatin+on+antibody%2C+interleukin-4+and+gamma-interferon+production+in+mice+immunized+with+egg+albumin. | | | |
| 734 | Molecular biology reports | Mahfouz RA | 2006 | 0 | http://www.ncbi.nlm.nih.gov/pubmed/?term=Apolipoprotein+E+gene+polymorphism+and+allele+frequencies+in+the+Lebanese+population. | | | |
| 990 | International journal of cardiology | Isma'eel H | 2006 | 0 | http://www.ncbi.nlm.nih.gov/pubmed/16887209 | | | |
| 396 | Public Health Nutrition | Nasreddine L | 2006 | 0 | http://www.ncbi.nlm.nih.gov/pubmed/?term=Food+consumption+patterns+in+an+adult+urban+population+in+Beirut%2C+Lebanon | | | |
| 576 | Acta Neurol Scand | Beydoun A | 2006 | 0 | http://www.ncbi.nlm.nih.gov/pubmed/16674606 | | | |
| 561 | Clin Vaccine Immunol | Almawi WY | 2006 | 0 | http://www.ncbi.nlm.nih.gov/pubmed/16988007 | | | |
| 198 | Anti-cancer drugs | El-Saghir N S | 2006 | 0 | http://www.ncbi.nlm.nih.gov/pubmed/?term=Combined+ovarian+ablation+and+aromatase+inhibition+as+first-line+therapy+for+hormone+receptor-positive+metastatic+breast+cancer+in+premenopausal+women%3A+report+of+three+cases | | | |
| 200 | Journal of cutaneous pathology | El-Tal A E | 2006 | 0 | http://www.ncbi.nlm.nih.gov/pubmed/16640543 | | | |
| 41 | International journal of gynecological cancer | Akoum R | 2006 | 0 | http://www.ncbi.nlm.nih.gov/pubmed/?term=Gynecological+tumors+revealing+hereditary+nonpolyposis+colorectal+cancer%3A+analysis+of+a+large+Lebanese+pedigree | | | |
| 265 | J Med Liban | Haddad F G | 2006 | 0 | http://www.ncbi.nlm.nih.gov/pubmed/?term=Obesity+and+related+diseases+in+a+Lebanese+medical+center%5D | | | |
| 934 | Journal de radiologie | El Abdi B | 2006 |  | http://www.ncbi.nlm.nih.gov/pubmed/16888594 | | | |
| 943 | Journal de radiologie | Sqalli Houssaini N | 2006 |  | http://www.ncbi.nlm.nih.gov/pubmed/16733418 | | | |
| 14 | Clinical Rheumatology | Achemlal L | 2006 | 0 | http://www.ncbi.nlm.nih.gov/pubmed/16247586 | | | |
| 24 | Indian journal of cancer | Ainahi A | 2006 | 0 | http://www.ncbi.nlm.nih.gov/pubmed/17065770 | | | |
| 64 | Cancer Radiothérapie | Belaabidia B | 2006 | 1 | http://www.ncbi.nlm.nih.gov/pubmed/16330234 | | | |
| 78 | Bulletin de la Société belge d'ophtalmologie | Benatiya Andaloussi I | 2006 | 0 | http://www.ncbi.nlm.nih.gov/pubmed/16903514 | | | |
| 80 | Journal of Craniofacial Surgery | Benazzou S | 2006 | 0 | http://www.ncbi.nlm.nih.gov/pubmed/17003639 | | | |
| 81 | Journal of Craniofacial Surgery | Benazzou S | 2006 | 0 | http://www.ncbi.nlm.nih.gov/pubmed/17119430 | | | |
| 436 | Annales d'Endocrinologie | Oudidi A | 2006 | 1 | http://www.ncbi.nlm.nih.gov/pubmed/17072244 | | | |
| 502 | International Journal of Urology | Tazzi H | 2006 | 0 | http://www.ncbi.nlm.nih.gov/pubmed/16643640 | | | |
| 751 | Archives de Pédiatrie | Hessissen L | 2006 | 1 | http://www.ncbi.nlm.nih.gov/pubmed/16531021 | | | |
| 928 | Neurochirurgie | Laghmari M | 2006 | 1 | http://www.ncbi.nlm.nih.gov/pubmed/17203903 | | | |
| 929 | Journal de gynécologie, obstétrique et biologie de la reproduction | Mharrech A | 2006 | 1 | http://www.ncbi.nlm.nih.gov/pubmed/17151543 | | | |
| 930 | Revue de stomatologie et de chirurgie maxillo-faciale | Oujilal A | 2006 | 1 | http://www.ncbi.nlm.nih.gov/pubmed/17128196 | | | |
| 931 | Revue neurologique | Rafai MA | 2006 | 1 | http://www.ncbi.nlm.nih.gov/pubmed/17086152 | | | |
| 932 | Annales de Cardiologie et d'Angéiologie | Belghiti H | 2006 | 1 | http://www.ncbi.nlm.nih.gov/pubmed/16922172 | | | |
| 933 | Bulletin de la Société belge d'ophtalmologie | El Kettani A | 2006 | 1 | http://www.ncbi.nlm.nih.gov/pubmed/16903510 | | | |
| 935 | Chirurgie de la main | Akjouj S | 2006 | 1 | http://www.ncbi.nlm.nih.gov/pubmed/16841775 | | | |
| 936 | Revue neurologique | Fikri M | 2006 | 1 | http://www.ncbi.nlm.nih.gov/pubmed/16840987 | | | |
| 938 | Presse médicale | MaÃ¢ouni S | 2006 | 1 | http://www.ncbi.nlm.nih.gov/pubmed/16840896 | | | |
| 939 | Annales de Dermatologie et de Vénéréologie | Terrab Z | 2006 | 1 | http://www.ncbi.nlm.nih.gov/pubmed/16760834 | | | |
| 940 | La Revue de médecine interne | El aichaoui S | 2006 | 1 | http://www.ncbi.nlm.nih.gov/pubmed/16750282 | | | |
| 942 | Annales de Dermatologie et de Vénéréologie | Rouas L | 2006 | 1 | http://www.ncbi.nlm.nih.gov/pubmed/16733452 | | | |
| 944 | Annales de Chirurgie Plastique Esthétique | El Hamdouchi K | 2006 | 1 | http://www.ncbi.nlm.nih.gov/pubmed/16690191 | | | |
| 946 | Annales d'Endocrinologie | Znati K | 2006 | 1 | http://www.ncbi.nlm.nih.gov/pubmed/16596061 | | | |
| 947 | Annales d'Endocrinologie | Benazzouz B | 2006 | 1 | http://www.ncbi.nlm.nih.gov/pubmed/16596053 | | | |
| 948 | Journal de radiologie | Fikri M | 2006 | 1 | http://www.ncbi.nlm.nih.gov/pubmed/16550116 | | | |
| 949 | Progrès en urologie | Mezzour MH | 2006 | 1 | http://www.ncbi.nlm.nih.gov/pubmed/16526548 | | | |
| 950 | Journal de radiologie | Adnani A | 2006 | 1 | http://www.ncbi.nlm.nih.gov/pubmed/16484937 | | | |
| 954 | Indian Journal of Otolaryngology and Head and Neck Surgery | Mansouri H | 2006 | 1 | http://www.ncbi.nlm.nih.gov/pubmed/23120255 | | | |
| 25 | Indian journal of cancer | Ainahi A | 2006 | 0 | http://www.ncbi.nlm.nih.gov/pubmed/16790944 | | | |
| 29 | The International Journal of Tuberculosis and Lung Disease | Aït-Khaled N | 2006 | 0 | http://www.ncbi.nlm.nih.gov/pubmed/16466046 | | | |
| 30 | The International Journal of Tuberculosis and Lung Disease | Aït-Khaled N | 2006 | 0 | http://www.ncbi.nlm.nih.gov/pubmed/16898377 | | | |
| 134 | The International Journal of Tuberculosis and Lung Disease | Bouayad Z | 2006 | 0 | http://www.ncbi.nlm.nih.gov/pubmed/16602399 | | | |
| 222 | Clinica chimica acta; international journal of clinical chemistry | El Messal M | 2006 | 0 | http://www.ncbi.nlm.nih.gov/pubmed/16280123 | | | |
| 227 | The International Journal of Tuberculosis and Lung Disease | El Rhazi K | 2006 | 0 | http://www.ncbi.nlm.nih.gov/pubmed/17131788 | | | |
| 371 | Pediatr Blood Cancer | Madani A | 2006 | 0 | http://www.ncbi.nlm.nih.gov/pubmed/16035094 | | | |
| 428 | Rev Laryngol Otol Rhinol (Bord) | Nouri H | 2006 | 1 | http://www.ncbi.nlm.nih.gov/pubmed/17315794 | | | |
| 455 | Body Image | Rguibi M | 2006 | 0 | http://www.ncbi.nlm.nih.gov/pubmed/18089243 | | | |
| 460 | La Presse Médicale | Sabry M | 2006 | 1 | http://www.ncbi.nlm.nih.gov/pubmed/16493348 | | | |
| 937 | Annales d'Endocrinologie | Kabbaj N | 2006 | 1 | http://www.ncbi.nlm.nih.gov/pubmed/16840914 | | | |
| 74 | Diabetes Research and Clinical Practice | Benaji B | 2006 | 1 | http://www.ncbi.nlm.nih.gov/pubmed/16647781 | | | |
| 220 | Acta Cardiologica | El Messal M | 2006 | 0 | http://www.ncbi.nlm.nih.gov/pubmed/17117756 | | | |
| 941 | Annales françaises d'Oto-rhino-laryngologie et de Pathologie Cervico-faciale | M'Rabti H | 2006 | 1 | http://www.ncbi.nlm.nih.gov/pubmed/16733467 | | | |
| 51 | Phytotherapy Research | Amrani S | 2006 | 0 | http://www.ncbi.nlm.nih.gov/pubmed/17006976 | | | |
| 160 | Clinica Chimica Acta | Chater R | 2006 | 0 | http://www.ncbi.nlm.nih.gov/pubmed/16806138 | | | |
| 238 | Journal of ethnopharmacology | EL-Hilaly J | 2006 | 0 | http://www.ncbi.nlm.nih.gov/pubmed/16417981 | | | |
| 362 | Journal of Ethnopharmacology | Lemhadri A | 2006 | 0 | http://www.ncbi.nlm.nih.gov/pubmed/16567073 | | | |
| 528 | Journal of Ethnopharmacology | Zeggwagh NA | 2006 | 0 | http://www.ncbi.nlm.nih.gov/pubmed/16787724 | | | |
| 451 | Eastern Mediterranean Health Journal | Rguibi M | 2006 | 0 | http://www.ncbi.nlm.nih.gov/pubmed/17333802 | | | |
| 454 | Public health nutrition | Rguibi M | 2006 | 0 | http://www.ncbi.nlm.nih.gov/pubmed/16925877 | | | |
| 462 | Evidence Based Complementary and Alternative Medicine | Samane S | 2006 | 1 | http://www.ncbi.nlm.nih.gov/pubmed/16951716 | | | |
| 127 | Clinical Nuclear Medicine | Biyi A | 2006 | 0 | http://www.ncbi.nlm.nih.gov/pubmed/16785816 | | | |
| 359 | Acta Cardiologica | Laraqui A | 2006 | 0 | http://www.ncbi.nlm.nih.gov/pubmed/16485733 | | | |
| 181 | Cancer Investigation | Drissi A | 2006 | 0 | http://www.ncbi.nlm.nih.gov/pubmed/16982463 | | | |
| 184 | Virology | Duprez R | 2006 | 0 | http://www.ncbi.nlm.nih.gov/pubmed/16793109 | | | |
| 54 | The Journal of asthma | El-Sharif N | 2006 | 0 | http://www.ncbi.nlm.nih.gov/pubmed/16754529 | | | |
| 92 | BMC public health | Eljedi A | 2006 | 0 | http://www.ncbi.nlm.nih.gov.ezproxy.aub.edu.lb/pubmed/17074088 | | | |
| 96 | International journal of epidemiology | Kark JD | 2006 | 0 | http://www.ncbi.nlm.nih.gov.ezproxy.aub.edu.lb/pubmed/16455758 | | | |
| 53 | J Med Chem | Najajreh Y | 2006 | 0 | http://www.ncbi.nlm.nih.gov/pubmed/16854072 | | | |
| 5 | Diabetes research and clinical practice | Abdelgadir M | 2006 | 0 | http://www.ncbi.nlm.nih.gov/pubmed/?term=The+influence+of+glucose+self-monitoring+on+glycaemic+control+in+patients+with+diabetes+mellitus+in+Sudan | | | |
| 34 | Journal of the National Medical Association | Awadalla A W | 2006 | 0 | http://www.ncbi.nlm.nih.gov/pubmed/16749648 | | | |
| 54 | Journal of diabetes and its complications | Elamin A | 2006 | 0 | http://www.ncbi.nlm.nih.gov.ezproxy.aub.edu.lb/pubmed/16798477 | | | |
| 163 | Journal of the National Medical Association | Awadalla AW | 2006 | 0 | http://www.ncbi.nlm.nih.gov/pubmed/16749649 | | | |
| 26 | Journal of family and community medicine | Ahmed M E | 2006 | 0 | http://www.ncbi.nlm.nih.gov/pubmed/?term=The+role+of+medical+students+in+patient+education+to+promote+home+management+of+diabetes+mellitus+in+wad+medani+town%2C+Sudan+2003 | | | |
| 70 | Annals of oncology | Hamad H M | 2006 | 0 | http://www.ncbi.nlm.nih.gov/pubmed/?term=Cancer+initiatives+in+Sudan | | | |
| 47 | Clinical cancer research | Dysvik B | 2006 | 0 | http://www.ncbi.nlm.nih.gov/pubmed/?term=Gene+expression+profiles+of+head+and+neck+carcinomas+from+Sudanese+and+Norwegian+patients+reveal+common+biological+pathways+regardless+of+race+and+lifestyle | | | |
| 131 | Annals of Saudi Medicine | Alhilli FA | 2007 |  | http://www.ncbi.nlm.nih.gov/pubmed/?term=Lung+cancer+in+Bahrain+(1952-2004). | | | |
| 141 | Journal of Thrombosis Thrombolysis | Gupta PK | 2007 | 0 | http://www.ncbi.nlm.nih.gov/pubmed/?term=Bilateral+medial+cerebellar+infarction+in+a+patient+positive+for+lupus+anticoagulant. | | | |
| 129 | Neuroepidemiology | Kamran S | 2007 | 0 | http://www.ncbi.nlm.nih.gov.ezproxy.aub.edu.lb/pubmed/?term=The+level+of+awareness+of+stroke+risk+factors+and+symptoms+in+the+Gulf+++++++Cooperation+Council+countries%3A+Gulf+Cooperation+Council+stroke+awareness+study | | | |
| 132 | Eastern Mediterranean health journal | Fadhil I | 2007 | 0 | http://www.ncbi.nlm.nih.gov/pubmed/?term=Tobacco+control+in+Bahrain%3A+an+overview. | | | |
| 144 | Diabetes Research and Clinical Practice | Golbahar J | 2007 | 0 | http://www.ncbi.nlm.nih.gov/pubmed/16963146 | | | |
| 201 | Annals of Saudi Medicine | Alsayyad J | 2007 | 0 | http://www.ncbi.nlm.nih.gov/pubmed/17684428 | | | |
| 138 | Current Neurovascular Research | Al-Bahrani A | 2007 | 0 | http://www.ncbi.nlm.nih.gov/pubmed/?term=TNF-alpha+and+IL-8+in+acute+stroke+and+the+modulation+of+these+cytokines+by+antiplatelet+agents. | | | |
| 139 | Annals of Saudi Medicine | Al-Mahroos F | 2007 | 0 | http://www.ncbi.nlm.nih.gov/pubmed/?term=Diabetic+neuropathy%2C+foot+ulceration%2C+peripheral+vascular+disease+and+potential+risk+factors+among+patients+with+diabetes+in+Bahrain%3A+a+nationwide+primary+care+diabetes+clinic-based+study. | | | |
| 134 | The Gulf Journal of Oncology | Al-Zahrani AS | 2007 | 0 | http://www.ncbi.nlm.nih.gov/pubmed/20084720 | | | |
| 112 | Saudi Medical Journal | Mansour AA | 2007 |  | http://www.ncbi.nlm.nih.gov/pubmed/?term=Metformin+discontinuation+rate+among+patients+with+type-2+diabetes+mellitus+in+Basrah%2C+Iraq. | | | |
| 117 | Endocrine Practice | Al-Himyari FA | 2007 |  | http://www.ncbi.nlm.nih.gov/pubmed/?term=Stress+hyperglycemia+in+nondiabetic+Iraqi+patients+presenting+with+acute+stroke. | | | |
| 126 | Saudi Medical Journal | Sharquie KE | 2007 | 0 | http://www.ncbi.nlm.nih.gov/pubmed/?term=Invasive+squamous+cell+carcinoma+of+the+eyes+in+patients+with+epidermodysplasia+verruciformis. | | | |
| 205 | Ultrastructural Pathology | Kummoona R | 2007 | 0 | http://www.ncbi.nlm.nih.gov/pubmed/?term=Ultrastructural+studies+of+jaw+lymphomas+and+apoptosis. | | | |
| 111 | Rural and Remote Health | Mansour AA | 2007 | 0 | http://www.ncbi.nlm.nih.gov/pubmed/?term=Cut-off+values+for+waist+circumference+in+rural+Iraqi+adults+for+the+diagnosis+of+metabolic+syndrome. | | | |
| 115 | Conflict and Health | Siziya S | 2007 | 0 | http://www.ncbi.nlm.nih.gov/pubmed/?term=Correlates+of+current+cigarette+smoking+among+in-school+adolescents+in+the+Kurdistan+region+of+Iraq. | | | |
| 121 | Asian Pacific Journal of Cancer Prevention | Habib OS | 2007 | 0 | http://www.ncbi.nlm.nih.gov/pubmed/?term=Cancer+registration+in+Basrah+2005%3A+preliminary+results. | | | |
| 123 | Annals of Nutrition Metabolism | Mansour AA | 2007 | 0 | http://www.ncbi.nlm.nih.gov/pubmed/?term=Predictors+of+incident+diabetes+mellitus+in+Basrah%2C+Iraq. | | | |
| 128 | Archives of Medical Research | Mansour AA | 2007 | 0 | http://www.ncbi.nlm.nih.gov/pubmed/?term=Cut-off+values+for+anthropometric+variables+that+confer+increased+risk+of+type+2+diabetes+mellitus+and+hypertension+in+Iraq. | | | |
| 130 | Annals of Thoracic Medicine | Al Obaidi AH | 2007 | 0 | http://www.ncbi.nlm.nih.gov/pubmed/?term=Expired+breath+condensate+hydrogen+peroxide+concentration+and+pH+for+screening+cough+variant+asthma+among+chronic+cough. | | | |
| 204 | Annals of Burns and Fire Disasters | Kadir AR | 2007 | 0 | http://www.ncbi.nlm.nih.gov/pubmed/21991095 | | | |
| 206 | Sultan Qaboos University Medical Journal | Ali HY | 2007 | 0 | http://www.ncbi.nlm.nih.gov/pubmed/?term=Antiphosphatidyl+serine+autoantibodies+and+premature+coronary+events. | | | |
| 208 | Neurosciences | Al-Nimer MS | 2007 | 0 | http://www.ncbi.nlm.nih.gov/pubmed/?term=Assessment+of+nitrosative+oxidative+stress+in+patients+with+middle+cerebral+artery+occlusion. | | | |
| 124 | Annals of Thoracic Medicine | Al Obaidi AH | 2007 | 0 | http://www.ncbi.nlm.nih.gov/pubmed/?term=Role+of+airway+lactoperoxidase+in+scavenging+of+hydrogen+peroxide+damage+in+asthma. | | | |
| 125 | Lancet | Mansour AA | 2007 | 0 | http://www.ncbi.nlm.nih.gov/pubmed/17544764 | | | |
| 207 | Journal of Pineal Research | Hussain SA | 2007 | 0 | http://www.ncbi.nlm.nih.gov/pubmed/17349025 | | | |
| 116 | Journal of Craniofacial Surgery | Kummoona R | 2007 | 0 | http://www.ncbi.nlm.nih.gov/pubmed/?term=Periorbital+and+orbital+malignancies%3A+methods+of+management+and+reconstruction+in+Iraq. | | | |
| 113 | Saudi Medical Journal | Mula-Abed WA | 2007 | 0 | http://www.ncbi.nlm.nih.gov/pubmed/?term=Prevalence+of+dyslipidemia+in+the+Iraqi+adult+population. | | | |
| 114 | Saudi Medical Journal | Al-Obaidy AH | 2007 | 0 | http://www.ncbi.nlm.nih.gov/pubmed/18060217 | | | |
| 122 | Eastern Mediterranean Health Journal | Al-Tawil NG | 2007 | 0 | http://www.ncbi.nlm.nih.gov/pubmed/?term=Prevalence+of+and+factors+associated+with+overweight+and+obesity+among+a+group+of+Iraqi+women. | | | |
| 129 | Saudi Medical Journal | Sharquie KE | 2007 | 0 | http://www.ncbi.nlm.nih.gov/pubmed/?term=Acral+lentiginous+melanoma+versus+lentigo+maligna+melanoma+among+Iraqi+patients. | | | |
| 118 | Journal of Medicinal Food | Hussain SA | 2007 | 0 | http://www.ncbi.nlm.nih.gov/pubmed/?term=Silymarin+as+an+adjunct+to+glibenclamide+therapy+improves+long-term+and+postprandial+glycemic+control+and+body+mass+index+in+type+2+diabetes. | | | |
| 401 | Medical Principles and Practice | Al-Enezi A | 2007 | 0 | http://www.ncbi.nlm.nih.gov/pubmed/17303956 | | | |
| 460 | Angiology | Al-Sayegh A | 2007 | 0 | http://www.ncbi.nlm.nih.gov/pubmed/17351169 | | | |
| 576 | The Gulf Journal of Oncology | Fayaz S | 2007 | 0 | http://www.ncbi.nlm.nih.gov.ezproxy.aub.edu.lb/pubmed/?term=Case+report+of+long+term+survivor+of+metastatic+cloacogenic+carcinoma+of+the+anal+canal+with+chemotherapy. | | | |
| 589 | Medical Principles and Practice | Hayat S | 2007 | 0 | http://www.ncbi.nlm.nih.gov.ezproxy.aub.edu.lb/pubmed/?term=Acute+myocardial+infarction+following+sildenafil+intake+in+a+nitrate-free+patient+without+previous+history+of+coronary+artery+disease. | | | |
| 701 | Acta Haematologica | Ramamoorthy SK | 2007 | 0 | http://www.ncbi.nlm.nih.gov/pubmed/?term=Safety+of+imatinib+in+chronic+myeloid+leukemia+in+blastic+crisis+presenting+as+cholestatic+jaundice | | | |
| 714 | Medical Principles and Practice | Selvan JP | 2007 | 0 | http://www.ncbi.nlm.nih.gov.ezproxy.aub.edu.lb/pubmed/17159370 | | | |
| 903 | Medical Principles and Practice | Zubaid M | 2007 | 0 | http://www.ncbi.nlm.nih.gov/pubmed/17917438 | | | |
| 38 | Neuroepidemiology | Kamran S | 2007 | 0 | http://www.ncbi.nlm.nih.gov.ezproxy.aub.edu.lb/pubmed/?term=The+level+of+awareness+of+stroke+risk+factors+and+symptoms+in+the+Gulf+++++++Cooperation+Council+countries%3A+Gulf+Cooperation+Council+stroke+awareness+study | | | |
| 44 | Saudi Medical Journal | Al-Adsani AM | 2007 | 0 | http://www.ncbi.nlm.nih.gov/pubmed/17457481 | | | |
| 47 | The British Journal of general Practice | Serour M | 2007 | 0 | http://www.ncbi.nlm.nih.gov/pubmed/17394732 | | | |
| 50 | BJU International | Al-Humnayan A | 2007 | 0 | http://www.ncbi.nlm.nih.gov/pubmed/?term=The+prevalence+and+predictors+of+erectile+dysfunction+in+men+with+newly+diagnosed+++++++with+type+2+diabetes+mellitus | | | |
| 131 | International Journal of Cardiology | AlKhalaf M | 2007 | 0 | http://www.ncbi.nlm.nih.gov/pubmed/19565011 | | | |
| 182 | Scandinavian Journal of Clinical and Laboratory Investigation | Mojiminiyi OA | 2007 | 0 | http://www.ncbi.nlm.nih.gov.ezproxy.aub.edu.lb/pubmed/17366001 | | | |
| 184 | International Journal of Obesity | Mojiminiyi OA | 2007 | 0 | http://www.ncbi.nlm.nih.gov.ezproxy.aub.edu.lb/pubmed/16755284 | | | |
| 353 | The Gulf Journal of Oncology | Abuzallouf S | 2007 | 0 | http://www.ncbi.nlm.nih.gov/pubmed/20084709 | | | |
| 354 | Medical Principles and Practice | Abuzallouf S | 2007 | 0 | http://www.ncbi.nlm.nih.gov/pubmed/17159359 | | | |
| 356 | Eastern Mediterranean Health Journal | Afifi M | 2007 | 0 | http://www.ncbi.nlm.nih.gov/pubmed/17684870 | | | |
| 362 | Metabolism | Akanji AO | 2007 | 0 | http://www.ncbi.nlm.nih.gov/pubmed/17379005 | | | |
| 363 | Scandinavian Journal of Clinical and Laboratory Investigation | Akanji AO | 2007 | 0 | http://www.ncbi.nlm.nih.gov/pubmed/17763192 | | | |
| 380 | The Gulf Journal of Oncology | Al Saleh K | 2007 | 0 | http://www.ncbi.nlm.nih.gov/pubmed/20084711 | | | |
| 450 | Medical Principles and Practice | Al-Qaoud N | 2007 | 0 | http://www.ncbi.nlm.nih.gov/pubmed/17541295 | | | |
| 475 | Medical Principles and Practice | Alfeeli MA | 2007 | 0 | http://www.ncbi.nlm.nih.gov/pubmed/17159362 | | | |
| 480 | International Journal of Cardiology | Alkhalaf M | 2007 | 0 | http://www.ncbi.nlm.nih.gov/pubmed/16797751 | | | |
| 606 | Eastern Mediterranean health journal = La revue de santé de la Méditerranée orientale = al-Majallah | Jackson RT | 2007 | 0 | http://www.ncbi.nlm.nih.gov.ezproxy.aub.edu.lb/pubmed/?term=Comparison+of+BMI-for-age+in+adolescent+girls+in+3+countries+of+the+Eastern+Mediterranean+Region. | | | |
| 634 | Medical Principles and Practice | Khoursheed M | 2007 | 0 | http://www.ncbi.nlm.nih.gov.ezproxy.aub.edu.lb/pubmed/?term=Slippage+after+adjustable+gastric+banding+according+to+the+pars+flaccida+and+the+perigastric+approach. | | | |
| 650 | Acta Haematologica | Marouf R | 2007 | 0 | http://www.ncbi.nlm.nih.gov.ezproxy.aub.edu.lb//pubmed/17135722 | | | |
| 746 | The Gulf Journal of Oncology | Vasishta S | 2007 | 0 | http://www.ncbi.nlm.nih.gov.ezproxy.aub.edu.lb/pubmed/20084713 | | | |
| 166 | Molecular and Cellular Biochemistry | Dashti HM | 2007 | 0 | http://www.ncbi.nlm.nih.gov.ezproxy.aub.edu.lb/pubmed/17447017 | | | |
| 747 | Annals of Saudi Medicine | Sheikh M | 2007 | 0 | http://www.ncbi.nlm.nih.gov.ezproxy.aub.edu.lb/pubmed/?term=Relative+contribution+of+digital+rectal+examination+and+transrectal+ultrasonography+in+interpreting+serum+prostate-specific+antigen+values+for+screening+prostate+cancer+in+Arab+men | | | |
| 352 | The Gulf Journal of Oncology | Abuzallouf S | 2007 | 0 | http://www.ncbi.nlm.nih.gov/pubmed/20084721 | | | |
| 439 | Methods in Molecular Biology | Al-Mulla F | 2007 | 0 | http://www.ncbi.nlm.nih.gov/pubmed/18220226 | | | |
| 445 | Clinical and Experimental Medicine | Al-Mutairi SS | 2007 | 0 | http://www.ncbi.nlm.nih.gov/pubmed/17609877 | | | |
| 466 | Current Opinion in Ophthalmology | Alabduljalil T | 2007 | 0 | http://www.ncbi.nlm.nih.gov/pubmed/18162997 | | | |
| 678 | Nicotine and Tobacco Research | Neergaard J | 2007 | 0 | http://www.ncbi.nlm.nih.gov.ezproxy.aub.edu.lb/pubmed/17943617 | | | |
| 716 | Inflammopharmacology | Sharma JN | 2007 | 0 | http://www.ncbi.nlm.nih.gov.ezproxy.aub.edu.lb/pubmed/?term=Role+of+nitric+oxide+in+inflammatory+diseases+Sharma+JN | | | |
| 45 | Methods and Findings in experimental and clinical pharmacology | Sharma JN | 2007 | 0 | http://www.ncbi.nlm.nih.gov/pubmed/?term=Changes+in+plasma+prekallikrein+activity%2C+blood+pressure%2C+and+left+ventricular+++++++thickness+in+hypertensive+and+normotensive+diabetic+rats. | | | |
| 48 | Journal of Vascular Research | Al-Sarraf H | 2007 | 0 | http://www.ncbi.nlm.nih.gov/pubmed/17191032 | | | |
| 156 | American Journal of Heart and Circulatory Physiology | Benter IF | 2007 | 0 | http://www.ncbi.nlm.nih.gov/pubmed/17213482 | | | |
| 165 | Diabetes Research and Clinical Practice | Chehadeh W | 2007 | 0 | http://www.ncbi.nlm.nih.gov.ezproxy.aub.edu.lb/pubmed/?term=Predictors+of+glucose+intolerance+in+HCV-infected+patients+with+no+family+history+of+diabetes | | | |
| 219 | Vascular Pharmacology | Yousif MH | 2007 | 0 | http://www.ncbi.nlm.nih.gov/pubmed/?term=Role+of+cytochrome+P450+metabolites+of+arachidonic+acid+in+regulation+of+corporal+smooth+muscle+tone+in+diabetic+and+older+rats | | | |
| 227 | Pharmacological Research | Yousif MH | 2007 | 0 | http://www.ncbi.nlm.nih.gov/pubmed/?term=Different+responses+to+angiotensin-(1-7)+in+young%2C+aged+and+diabetic+rabbit+corpus+cavernosum | | | |
| 350 | International Urology and Nephrology | Abul FT | 2007 | 0 | http://www.ncbi.nlm.nih.gov/pubmed/17308874 | | | |
| 464 | Acta Cardiologica | Al-Zaid NS | 2007 | 0 | http://www.ncbi.nlm.nih.gov/pubmed/17824299 | | | |
| 481 | Pharmacology | Alkhalaf M | 2007 | 0 | http://www.ncbi.nlm.nih.gov/pubmed/17534123 | | | |
| 482 | European Journal of Cancer Prevention | Alkhalaf M | 2007 | 0 | http://www.ncbi.nlm.nih.gov/pubmed/17554206 | | | |
| 550 | Clinical Medicine and Research | Doi SA | 2007 | 0 | http://www.ncbi.nlm.nih.gov/pubmed/17607042 | | | |
| 583 | Diagnostic Cytopathology | Haji BE | 2007 | 0 | http://www.ncbi.nlm.nih.gov.ezproxy.aub.edu.lb/pubmed/?term=Fine-needle+aspiration+cytologic+features+of+four+special+types+of+breast+cancers%3A+mucinous%2C+medullary%2C+apocrine%2C+and+papillary. | | | |
| 592 | Medical Principles and Practice | Hegazy AM | 2007 | 0 | http://www.ncbi.nlm.nih.gov.ezproxy.aub.edu.lb/pubmed/?term=Early+changes+in+ventricular+repolarization+after+thrombolytic+therapy+in+patients+with+acute+myocardial+infarction+as+indicators+for+prediction+of+epicardial+coronary+artery+reperfusion. | | | |
| 593 | Medical Principles and Practice | Hegazy AM | 2007 | 0 | http://www.ncbi.nlm.nih.gov.ezproxy.aub.edu.lb/pubmed/?term=Predictive+accuracy+of+tissue+Doppler+imaging+for+assessment+of+noninfarct+myocardial+region+in+patients+with+acute+myocardial+infarction. | | | |
| 633 | The International Journal of Biological Markers | Kharrat N | 2007 | 0 | http://www.ncbi.nlm.nih.gov.ezproxy.aub.edu.lb/pubmed/?term=(AC)+dinucleotide+repeat+polymorphism+in+intron+1+of+human+EGFR+shows+ethnic+specificities+and+high+evidence+for+association+with+breast+cancer. | | | |
| 696 | American Journal of Reproductive Immunology | Raghupathy R | 2007 | 0 | http://onlinelibrary.wiley.com/doi/10.1111/j.1600-0897.2007.00488.x/pdf | | | |
| 709 | Journal of Carcinogenesis | Saleh F | 2007 | 0 | http://www.ncbi.nlm.nih.gov.ezproxy.aub.edu.lb/pubmed/?term=Pathobiological+features+of+breast+tumours+in+the+State+of+Kuwait%3A+a+comprehensive+analysis | | | |
| 873 | Current Therapeutic Research Clinical and Experimental | Al-Saeedi F | 2007 | 0 | http://www.ncbi.nlm.nih.gov/pubmed/24683213 | | | |
| 874 | Anticancer Research | Al-Saeedi F | 2007 | 0 | http://www.ncbi.nlm.nih.gov/pubmed/17465218 | | | |
| 502 | BMC Cancer | Awadalla AW | 2007 | 0 | http://www.ncbi.nlm.nih.gov/pubmed/17578579 | | | |
| 521 | BMC Med Genet | Bohlega S | 2007 | 0 | http://www.ncbi.nlm.nih.gov/pubmed/17996090 | | | |
| 532 | Cytopathology | Das DK | 2007 | 0 | http://www.ncbi.nlm.nih.gov/pubmed/17488258 | | | |
| 533 | Diagnostic Cytopathology | Das DK | 2007 | 0 | http://www.ncbi.nlm.nih.gov/pubmed/17497663 | | | |
| 42 | Journal of Cardiovascular Surgery | Asfar S | 2007 | 0 | http://www.ncbi.nlm.nih.gov/pubmed/17989630 | | | |
| 46 | Medical Principles and Practice | Hegazy AM | 2007 | 0 | http://www.ncbi.nlm.nih.gov/pubmed/?term=Cardiac+memory+versus+likelihood+of+ischemic+heart+disease+in+hypertensive+++++++patients+with+ventricular+repolarization+abnormalities+after+repetitive+uniform+++++++ventricular+extrasystoles | | | |
| 426 | Nursing & Health sciences | Al-Kandari F | 2007 | 0 | http://www.ncbi.nlm.nih.gov/pubmed/17470185 | | | |
| 453 | Analytical Chemistry Insights | Al-Saeedi F | 2007 | 0 | http://www.ncbi.nlm.nih.gov/pubmed/19662181 | | | |
| 463 | The Gulf Journal of Oncology | Al-Zahrani AS | 2007 | 0 | http://www.ncbi.nlm.nih.gov/pubmed/20084720 | | | |
| 126 | Circulation | Chehab G | 2007 |  | Not Found | |  |  |
| 169 | Southern medical journal | El Hajj II | 2007 |  | Not Found | |  |  |
| 362 | Diabetes Care | Merheb M | 2007 |  | Abstract Not Found | |  |  |
| 409 | Gut | Noun R | 2007 |  | Abstract Not Found | |  |  |
| 428 | Digestive diseases and sciences | Otrock Z K | 2007 |  | Abstract Not Found | |  |  |
| 56 | International surgery | Arabi A | 2007 | 0 | http://www.ncbi.nlm.nih.gov/pubmed/?term=Papillary+carcinoma+arising+in+a+thyroglossal+duct+cyst%3B+two+case+reports+and+review+of+the+literature | | | |
| 70 | Clinical lymphoma & myeloma | Azar C | 2007 | 0 | http://www.ncbi.nlm.nih.gov/pubmed/17621410 | | | |
| 312 | The oncologist | Jamali F R | 2007 | 0 | http://www.ncbi.nlm.nih.gov/pubmed/?term=Disease+progression+following+imatinib+failure+in+gastrointestinal+stromal+tumors%3A+role+of+surgical+therapy | | | |
| 313 | Leuk Lymphoma | Jamali F R | 2007 | 0 | http://www.ncbi.nlm.nih.gov/pubmed/?term=An+overview+of+the+pathogenesis+and+natural+history+of+post-transplant+T-cell+lymphoma+(corrected+and+republished+article+originally+printed+in+Leukemia+%26+Lymphoma%2C+June+2007%3B+48(6)%3A+1237+-+1241) | | | |
| 627 | Am J of Hematology | Hatoum HA | 2007 | 0 | http://www.ncbi.nlm.nih.gov/pubmed/?term=Acute+myeloid+leukemia+with+T-cell+receptor+gamma+gene+rearrangement+occurring+in+a+patient+with+chronic+lymphocytic+leukemia%3A+a+case+report. | | | |
| 1033 | Digestive diseases and sciences | Otrock ZK | 2007 | 0 | http://www.ncbi.nlm.nih.gov/pubmed/17436106 | | | |
| 40 | Journal of cancer research and therapeutics | Akoum R | 2007 | 0 | http://www.ncbi.nlm.nih.gov/pubmed/18079576 | | | |
| 78 | Inhalation toxicology | Bacha Z A | 2007 | 0 | http://www.ncbi.nlm.nih.gov/pubmed/?term=Saliva+cotinine+and+exhaled+carbon+monoxide+levels+in+natural+environment+waterpipe+smokers | | | |
| 104 | Canadian Urological Association journal | Bulbul M A | 2007 | 0 | http://www.ncbi.nlm.nih.gov/pubmed/18542801 | | | |
| 112 | J Med Liban | Chakar H | 2007 | 0 | http://www.ncbi.nlm.nih.gov/pubmed/17685119 | | | |
| 117 | Echocardiography | Chammas E | 2007 | 0 | http://www.ncbi.nlm.nih.gov/pubmed/17767527 | | | |
| 155 | The open clinical cancer journal | Dbouk H A | 2007 | 0 | http://www.ncbi.nlm.nih.gov/pubmed/?term=Significance+of+CEA+and+VEGF+as+Diagnostic+Markers+of+Colorectal+Cancer+in+Lebanese+Patients | | | |
| 201 | Gastrointestinal Endoscopy | El-Zahabi L M | 2007 | 0 | http://www.ncbi.nlm.nih.gov/pubmed/?term=The+value+of+EUS+in+predicting+the+response+of+gastric+mucosa-associated+lymphoid+tissue+lymphoma+to+Helicobacter+pylori+eradication | | | |
| 241 | International journal of radiation oncology, biology, physics | Geara F B | 2007 | 0 | http://www.ncbi.nlm.nih.gov/pubmed/?term=PURPOSE%3A+To+analyze+tumor+control+and+survival+for+breast+cancer+patients+with+10+or+more+positive+lymph+nodes+without+systemic+disease%2C+treated+by+adjuvant+radiation+alone+or+combined-modality+therapy.+METHODS | | | |
| 290 | J Med Liban | Hoyek-Gebeily J | 2007 | 0 | http://www.ncbi.nlm.nih.gov/pubmed/?term=Prognostic+significance+of+EGFR%2C+p53+and+E-cadherin+in+mucoepidermoid+cancer+of+the+salivary+glands%3A+a+retrospective+case+series | | | |
| 309 | Eastern Mediterranean health journal | Jackson R T | 2007 | 0 | http://www.ncbi.nlm.nih.gov/pubmed/?term=Comparison+of+BMI-for-age+in+adolescent+girls+in+3+countries+of+the+Eastern+Mediterranean+Region | | | |
| 406 | Obesity surgery | Noun R | 2007 | 0 | http://www.ncbi.nlm.nih.gov/pubmed/?term=Mini-gastric+bypass+by+mini-laparotomy%3A+a+cost-effective+alternative+in+the+laparoscopic+era | | | |
| 448 | Molecular biology reports | Sabbagh A S | 2007 | 0 | http://www.ncbi.nlm.nih.gov/pubmed/?term=ApoB-100+R3500Q+mutation+in+the+Lebanese+population%3A+prevalence+and+historical+review+of+the+literature | | | |
| 499 | Soc Sci Med | Sibai A M | 2007 | 0 | http://www.ncbi.nlm.nih.gov/pubmed/?term=Marital+status%2C+intergenerational+co-residence+and+cardiovascular+and+all-cause+mortality+among+middle-aged+and+older+men+and+women+during+wartime+in+Beirut%3A+gains+and+liabilities | | | |
| 512 | Am J of health behavior | Tamim H | 2007 | 0 | http://www.ncbi.nlm.nih.gov/pubmed/?term=Cigarette+and+nargileh+smoking+practices+among+school+students+in+Beirut%2C+Lebanon | | | |
| 603 | Science of the Total Environment | El-Zein A | 2007 | 0 | http://www.ncbi.nlm.nih.gov/pubmed/?term=Did+a+ban+on+diesel-fuel+reduce+emergency+respiratory+admissions+for+children%3F | | | |
| 86 | Anaesthesia | Baraka A S | 2007 | 0 | http://www.ncbi.nlm.nih.gov/pubmed/?term=Supplementation+of+pre-oxygenation+in+morbidly+obese+patients+using+nasopharyngeal+oxygen+insufflation | | | |
| 250 | Am J of clinical oncology | Ghosn M | 2007 | 0 | http://www.ncbi.nlm.nih.gov/pubmed/?term=FOLFOX-6+combination+as+the+first-line+treatment+of+locally+advanced+and%2For+metastatic+pancreatic+cancer+Ghosn+2007 | | | |
| 101 | World journal of gastroenterology | Boujaoude J | 2007 | 0 | http://www.ncbi.nlm.nih.gov/pubmed/?term=Role+of+endoscopic+ultrasound+in+diagnosis+and+therapy+of+pancreatic+adenocarcinoma+Boujaoude+2007 | | | |
| 320 | Current opinion in psychiatry | Karam E | 2007 | 0 | http://www.ncbi.nlm.nih.gov/pubmed/?term=Alcohol+use+among+college+students%3A+an+international+perspective+karam+e+2007 | | | |
| 597 | Thromb Haemost | El Accaoui RN | 2007 | 0 | http://www.ncbi.nlm.nih.gov/pubmed/17549307 | | | |
| 643 | Kidney International | Karnib HH | 2007 | 0 | http://www.ncbi.nlm.nih.gov/pubmed/?term=Genes+for+diabetic+nephropathy%3A+sweet+prospects+on+the+horizon. | | | |
| 935 | Int J Surg | El Saghir NS | 2007 | 0 | http://www.ncbi.nlm.nih.gov/pubmed/17660128 | | | |
| 977 | Expert Opin Drug Discov | Darwiche N | 2007 | 0 | http://www.ncbi.nlm.nih.gov/pubmed/23484647 | | | |
| 87 | Food and nutrition bulletin | Batal M | 2007 | 0 | http://www.ncbi.nlm.nih.gov/pubmed/?term=Traditional+Lebanese+recipes+based+on+wild+plants%3A+an+answer+to+diet+simplification%3F | | | |
| 151 | Leukemia | Darwiche N | 2007 | 0 | http://www.ncbi.nlm.nih.gov/pubmed/?term=Reactive+oxygen+species+mediate+N-(4-hydroxyphenyl)retinamide-induced+cell+death+in+malignant+T+cells+and+are+inhibited+by+the+HTLV-I+oncoprotein+Tax | | | |
| 154 | Haematologica | Dbaibo G S | 2007 | 0 | http://www.ncbi.nlm.nih.gov/pubmed/?term=Arsenic+trioxide+induces+accumulation+of+cytotoxic+levels+of+ceramide+in+acute+promyelocytic+leukemia+and+adult+T-cell+leukemia%2Flymphoma+cells+through+de+novo+ceramide+synthesis+and+inhibition+of+glucosylceramide | | | |
| 192 | Oncology reports | El-Najjar N | 2007 | 0 | http://www.ncbi.nlm.nih.gov/pubmed/?term=Onopordum+cynarocephalum+induces+apoptosis+and+protects+against+1%2C2+dimethylhydrazine-induced+colon+cancer | | | |
| 278 | Anticancer research | Harakeh S | 2007 | 0 | http://www.ncbi.nlm.nih.gov/pubmed/17352246 | | | |
| 299 | Radiation oncology | Itani W | 2007 | 0 | http://www.ncbi.nlm.nih.gov/pubmed/?term=Radiosensitization+by+2-benzoyl-3-phenyl-6%2C7-dichloroquinoxaline+1%2C4-dioxide+under+oxia+and+hypoxia+in+human+colon+cancer+cells | | | |
| 558 | Molecular Carcinogenesis | Al-Ayyoubi S | 2007 | 0 | http://www.ncbi.nlm.nih.gov/pubmed/17192871 | | | |
| 654 | Journal of the renin-angiotensin-aldosterone system : JRAAS | Maharsy WM | 2007 | 0 | http://www.ncbi.nlm.nih.gov.ezproxy.aub.edu.lb/pubmed/17703431 | | | |
| 681 | Canadian Journal of Physiology and Pharmacology | Nuwayri-Salti N | 2007 | 0 | http://www.ncbi.nlm.nih.gov.ezproxy.aub.edu.lb/pubmed/?term=Effect+of+type-1+diabetes+mellitus+on+the+regulation+of+insulin+and+endothelin-1+receptors+in+rat+hearts. | | | |
| 739 | Molecular biology reports | Sabbagh AS | 2007 | 0 | http://www.ncbi.nlm.nih.gov/pubmed/17103020 | | | |
| 740 | Transfus Med | Sabbagh AS | 2007 | 0 | http://www.ncbi.nlm.nih.gov/pubmed/18067652 | | | |
| 964 | International journal of cardiology | Dakik HA | 2007 | 0 | http://www.ncbi.nlm.nih.gov/pubmed/17397950 | | | |
| 991 | International journal of cardiology | Isma'eel H | 2007 | 0 | http://www.ncbi.nlm.nih.gov/pubmed/16757043 | | | |
| 993 | International journal of cardiology | Isma'eel H | 2007 | 0 | http://www.ncbi.nlm.nih.gov/pubmed/16352353 | | | |
| 1007 | Molecular biology reports | Mahfouz R | 2007 | 0 | http://www.ncbi.nlm.nih.gov/pubmed/17149654 | | | |
| 285 | Soc Sci Med | Hospers A P | 2007 | 0 | http://www.ncbi.nlm.nih.gov/pubmed/?term=Health+care+delivery+systems+for+older+adults%3A+how+do+the+Netherlands+and+Lebanon+compare%3F | | | |
| 23 | Leukemia | Abou-Merhi R | 2007 | 0 | http://www.ncbi.nlm.nih.gov/pubmed/17568816 | | | |
| 262 | FASEB journal | Habib A | 2007 | 0 | http://www.ncbi.nlm.nih.gov/pubmed/?term=Modulation+of+COX-2+expression+by+statins+in+human+monocytic+cells | | | |
| 594 | Oncogene | Darwiche N | 2007 | 0 | http://www.ncbi.nlm.nih.gov/pubmed/17525749 | | | |
| 222 | J Med Liban | Farhat M H | 2007 | 0 | http://www.ncbi.nlm.nih.gov/pubmed/17966739 | | | |
| 408 | J Chir (Paris) | Noun R | 2007 | 0 | http://www.ncbi.nlm.nih.gov/pubmed/?term=Laparoscopic+mini-gastric+bypass%3A+an+effective+option+for+the+treatment+of+morbid+obesity+2007 | | | |
| 410 | Obesity surgery | Noun R | 2007 | 0 | http://www.ncbi.nlm.nih.gov/pubmed/?term=Mini-gastric+bypass+for+revision+of+failed+primary+restrictive+procedures%3A+a+valuable+option+noun+2007 | | | |
| 526 | J Med Liban | Waked M | 2007 | 0 | http://www.ncbi.nlm.nih.gov/pubmed/?term=Symptoms%2C+severity+and+asthma+control+in+5-14+y-old+Lebanon+school+children | | | |
| 534 | Middle East journal of anesthesiology | Yazigi A | 2007 | 0 | http://www.ncbi.nlm.nih.gov/pubmed/?term=The+accuracy+of+non-invasive+nasal+capnography+in+morbidly+obese+patients+after+bariatric+surgery | | | |
| 572 | Ethnicity & disease | Badr KF | 2007 | 0 | http://www.ncbi.nlm.nih.gov/pubmed/17682358 | | | |
| 115 | European journal of anaesthesiology | Chalhoub V | 2007 | 0 | http://www.ncbi.nlm.nih.gov/pubmed/?term=Effect+of+vital+capacity+manoeuvres+on+arterial+oxygenation+in+morbidly+obese+patients+undergoing+open+bariatric+surgery | | | |
| 215 | Medical oncology | Farhat F S | 2007 | 0 | http://www.ncbi.nlm.nih.gov/pubmed/?term=A+general+review+of+the+role+of+irinotecan+(CPT11)+in+the+treatment+of+gastric+cancer | | | |
| 162 | European journal of oncology nursing | Doumit M A | 2007 | 0 | http://www.ncbi.nlm.nih.gov/pubmed/?term=The+lived+experience+of+Lebanese+oncology+patients+receiving+palliative+care | | | |
| 918 | Journal de radiologie | Taleb F | 2007 |  | http://www.ncbi.nlm.nih.gov/pubmed/17457274 | | | |
| 27 | Journal of genetics | Ait Chihab K | 2007 | 0 | http://www.ncbi.nlm.nih.gov/pubmed/17968143 | | | |
| 69 | Annales d'Endocrinologie | Belhadi L | 2007 | 1 | http://www.ncbi.nlm.nih.gov/pubmed/17692810 | | | |
| 153 | Journal de Radiologie | Bricha M | 2007 | 0 | http://www.ncbi.nlm.nih.gov/pubmed/18235353 | | | |
| 232 | Southern Medical Journal | Elabsi M | 2007 | 0 | http://www.ncbi.nlm.nih.gov/pubmed/17943052 | | | |
| 233 | Presse médicale | Elabsi M | 2007 | 1 | http://www.ncbi.nlm.nih.gov/pubmed/17553658 | | | |
| 283 | Med Trop (Mars) | Harket A | 2007 | 1 | http://www.ncbi.nlm.nih.gov/pubmed/17784682 | | | |
| 289 | Journal français d'ophtalmologie | Hassikou H | 2007 | 1 | http://www.ncbi.nlm.nih.gov/pubmed/17318105 | | | |
| 332 | Journal des maladies vasculaires | Kamaoui I | 2007 | 1 | http://www.ncbi.nlm.nih.gov/pubmed/17276641 | | | |
| 414 | J Fr Ophtalmol | Naama O | 2007 | 1 | http://www.ncbi.nlm.nih.gov/pubmed/17318111 | | | |
| 427 | Rev Stomatol Chir Maxillofac | Nouri H | 2007 | 1 | http://www.ncbi.nlm.nih.gov/pubmed/17624384 | | | |
| 433 | Med Oral Patol Oral Cir Bucal | Otmani N | 2007 | 0 | http://www.ncbi.nlm.nih.gov/pubmed/17468714 | | | |
| 442 | Internal medicine | Rabhi M | 2007 | 0 | http://www.ncbi.nlm.nih.gov/pubmed/17409605 | | | |
| 493 | Renal Failure | Tarrass F | 2007 | 0 | http://www.ncbi.nlm.nih.gov/pubmed/18067055 | | | |
| 702 | Revue de Stomatologie et de Chirurgie Maxillo-faciale | Chbani L | 2007 | 1 | http://www.ncbi.nlm.nih.gov/pubmed/17537470 | | | |
| 908 | Neurochirurgie | Haddad H | 2007 | 1 | http://www.ncbi.nlm.nih.gov/pubmed/18061213 | | | |
| 909 | Revue neurologique | Rabhi M | 2007 | 1 | http://www.ncbi.nlm.nih.gov/pubmed/18033053 | | | |
| 910 | Annales de Dermatologie et de Vénéréologie | El Ghelbazouri N | 2007 | 1 | http://www.ncbi.nlm.nih.gov/pubmed/17925690 | | | |
| 911 | Revue de stomatologie et de chirurgie maxillo-faciale | Elboukhari A | 2007 | 1 | http://www.ncbi.nlm.nih.gov/pubmed/17919669 | | | |
| 912 | Annales de Biologie Clinique | Ballouch L | 2007 | 1 | http://www.ncbi.nlm.nih.gov/pubmed/17913677 | | | |
| 913 | Revue de stomatologie et de chirurgie maxillo-faciale | El Khatib K | 2007 | 1 | http://www.ncbi.nlm.nih.gov/pubmed/17881024 | | | |
| 914 | Neurochirurgie | Aniba K | 2007 | 1 | http://www.ncbi.nlm.nih.gov/pubmed/17707865 | | | |
| 915 | Annales de Cardiologie et d'Angéiologie | Benzarouel D | 2007 | 1 | http://www.ncbi.nlm.nih.gov/pubmed/17573029 | | | |
| 916 | Revue neurologique | Messouak O | 2007 | 1 | http://www.ncbi.nlm.nih.gov/pubmed/17571029 | | | |
| 917 | Annales d'Endocrinologie | Safi S | 2007 | 1 | http://www.ncbi.nlm.nih.gov/pubmed/17531185 | | | |
| 919 | Revue neurologique | Rahmani M | 2007 | 1 | http://www.ncbi.nlm.nih.gov/pubmed/17452948 | | | |
| 920 | Revue de stomatologie et de chirurgie maxillo-faciale | Benbouzid MA | 2007 | 1 | http://www.ncbi.nlm.nih.gov/pubmed/17399753 | | | |
| 921 | Annales de Chirurgie Plastique Esthétique | Belmahi A | 2007 | 1 | http://www.ncbi.nlm.nih.gov/pubmed/17382442 | | | |
| 922 | Revue neurologique | Messouak O | 2007 | 1 | http://www.ncbi.nlm.nih.gov/pubmed/17351544 | | | |
| 923 | Revue de stomatologie et de chirurgie maxillo-faciale | Bencheikh R | 2007 | 1 | http://www.ncbi.nlm.nih.gov/pubmed/17350059 | | | |
| 924 | Revue de stomatologie et de chirurgie maxillo-faciale | Bencheikh R | 2007 | 1 | http://www.ncbi.nlm.nih.gov/pubmed/17350056 | | | |
| 926 | Revue de stomatologie et de chirurgie maxillo-faciale | Znati K | 2007 | 1 | http://www.ncbi.nlm.nih.gov/pubmed/17275047 | | | |
| 927 | Revue des maladies respiratoires | Herrak L | 2007 | 1 | http://www.ncbi.nlm.nih.gov/pubmed/17268369 | | | |
| 31 | Allergy | Ait-Khaled N | 2007 | 0 | http://www.ncbi.nlm.nih.gov/pubmed/17298341 | | | |
| 110 | Eastern Mediterranean Health Journal | Bennis S | 2007 | 1 | http://www.ncbi.nlm.nih.gov/pubmed/18290409 | | | |
| 305 | International journal of environmental health research | Houssaini AS | 2007 | 0 | http://www.ncbi.nlm.nih.gov/pubmed/17613089 | | | |
| 456 | J Hypertens | Rguibi M | 2007 | 0 | http://www.ncbi.nlm.nih.gov/pubmed/17563556 | | | |
| 457 | Obesity Reviews | Rguibi M | 2007 | 0 | http://www.ncbi.nlm.nih.gov/pubmed/17212791 | | | |
| 489 | Journal of Ethnopharmacology | Tahraoui A | 2007 | 0 | http://www.ncbi.nlm.nih.gov/pubmed/17052873 | | | |
| 492 | Saudi Journal of Kidney Disease and Transplantation | Tarrass F | 2007 | 0 | http://www.ncbi.nlm.nih.gov/pubmed/17679745 | | | |
| 684 | Journal of Biomedicine and Biotechnology | Bennouar N | 2007 | 0 | http://www.ncbi.nlm.nih.gov/pubmed/17497026 | | | |
| 750 | Hepatology Research | Ezzikouri S | 2007 | 0 | http://www.ncbi.nlm.nih.gov/pubmed/17573955 | | | |
| 28 | Brazilian Journal of Medical and Biological Research | Ait M’Barek L | 2007 | 0 | http://www.ncbi.nlm.nih.gov/pubmed/17934650 | | | |
| 105 | Cancer Detection and Prevention | Bennani H | 2007 | 0 | http://www.ncbi.nlm.nih.gov/pubmed/17174037 | | | |
| 239 | Pak J Pharm Sci | EL-Hilaly J | 2007 | 0 | http://www.ncbi.nlm.nih.gov/pubmed/17604246 | | | |
| 256 | international journal of cancer | Feng BJ | 2007 | 0 | http://www.ncbi.nlm.nih.gov/pubmed/17582611 | | | |
| 438 | Dig Liver Dis | Pineau P | 2007 | 0 | http://www.ncbi.nlm.nih.gov/pubmed/17531558 | | | |
| 112 | Revue de Pneumologie Clinique | Benouhoud N | 2007 | 1 | http://www.ncbi.nlm.nih.gov/pubmed/17457283 | | | |
| 170 | Journal of Pediatric Hematology/Oncology | Dakka N | 2007 | 0 | http://www.ncbi.nlm.nih.gov/pubmed/17762501 | | | |
| 138 | Journal of Craniofacial Surgery | Boulaadas M | 2007 | 0 | http://www.ncbi.nlm.nih.gov/pubmed/17912082 | | | |
| 361 | Med Trop (Mars) | Lemerle J | 2007 | 1 | http://www.ncbi.nlm.nih.gov/pubmed/18225736 | | | |
| 192 | Journal of Herbal Pharmocotherapy | Eddouks M | 2007 | 0 | http://www.ncbi.nlm.nih.gov/pubmed/18285308 | | | |
| 49 | Int J Tuberc Lung Dis | Al Zabadi H | 2007 | 0 | http://www.ncbi.nlm.nih.gov/pubmed/17705961 | | | |
| 50 | Pharm World Sci | Sweileh WM | 2007 | 0 | http://www.ncbi.nlm.nih.gov/pubmed/17333496 | | | |
| 52 | J Ambul Care Manage | Ghosh HA | 2007 | 0 | http://www.ncbi.nlm.nih.gov/pubmed/17170640 | | | |
| 85 | Public health nutrition | Al Sabbah H | 2007 | 0 | http://www.ncbi.nlm.nih.gov.ezproxy.aub.edu.lb/pubmed/17381946 | | | |
| 95 | The Israel Medical Association journal: IMAJ | Jabara R | 2007 | 0 | http://www.ncbi.nlm.nih.gov.ezproxy.aub.edu.lb/pubmed/17491229 | | | |
| 157 | Food Nutr Bull | Abdeen Z | 2007 | 0 | http://www.ncbi.nlm.nih.gov/pubmed/17974360 | | | |
| 91 | International journal of surgery (London, England) | El Saghir NS | 2007 | 0 | http://www.ncbi.nlm.nih.gov.ezproxy.aub.edu.lb/pubmed/17660128 | | | |
| 51 | Chemosphere | Deeb O | 2007 | 0 | http://www.ncbi.nlm.nih.gov/pubmed/17307223 | | | |
| 134 | BMC cancer | Kadouri L | 2007 | 0 | http://www.ncbi.nlm.nih.gov.ezproxy.aub.edu.lb/pubmed/17233897 | | | |
| 22 | Journal of cancer research and therapeutics | Ahmed H G | 2007 | 0 | https://www.ncbi.nlm.nih.gov.ezproxy.aub.edu.lb/pubmed/?term=Impact+of+Toombak+dipping+in+the+etiology+of+oral+cancer%3A+gender-exclusive+hazard+in+the+Sudan | | | |
| 51 | Tropical doctor | El Zein A M | 2007 | 0 | http://www.ncbi.nlm.nih.gov.ezproxy.aub.edu.lb/pubmed/?term=Stroke+in+CT-scan+Department+of+Khartoum+Hospital%2C+Sudan | | | |
| 71 | Saudi medical journal | Haroun S A | 2007 | 0 | http://www.ncbi.nlm.nih.gov/pubmed/?term=Aggressive+rhabdomyosarcoma+of+the+vulva+in+a+young+Sudanese+woman | | | |
| 35 | BMC cancer | Awadalla A W | 2007 | 0 | http://www.ncbi.nlm.nih.gov/pubmed/?term=Factors+associated+with+quality+of+life+of+outpatients+with+breast+cancer+and+gynecologic+cancers+and+their+family+caregivers%3A+a+controlled+study | | | |
| 104 | Journal of family and community medicine | Osman el F M | 2007 | 0 | http://www.ncbi.nlm.nih.gov/pubmed/?term=Clinico-epidemiological+features+of+hypertensive+subjects+in+kassala+town%2C+eastern+Sudan | | | |
| 105 | East African medical journal | Osman E M | 2007 | 0 | http://www.ncbi.nlm.nih.gov/pubmed/?term=Patients+knowledge+of+hypertension+and+its+control+in+Eastern+Sudan | | | |
| 48 | Science of the total environment | Ebrahim A M | 2007 | 0 | http://www.ncbi.nlm.nih.gov/pubmed/?term=Study+of+selected+trace+elements+in+cancerous+and+non-cancerous+human+breast+tissues+from+Sudanese+subjects+using+instrumental+neutron+activation+analysis | | | |
| 29 | Allergy | Ait-Khaled N | 2007 | 0 | https://www.ncbi.nlm.nih.gov.ezproxy.aub.edu.lb/pubmed/?term=Prevalence+of+symptoms+of+asthma%2C+rhinitis+and+eczema+in+13-+to+14-year-old+children+in+Africa%3A+the+International+Study+of+Asthma+and+Allergies+in+Childhood+Phase+III | | | |
| 33 | Human biology | Al-Yahyaee S | 2007 | 0 | http://www.ncbi.nlm.nih.gov/pubmed/?term=N-acetyltransferase+polymorphism+among+northern+Sudanese | | | |
| 36 | Breast Cancer Research and Treatment | Awadelkarim K D | 2007 | 0 | http://www.ncbi.nlm.nih.gov/pubmed/?term=BRCA1+and+BRCA2+status+in+a+Central+Sudanese+series+of+breast+cancer+patients%3A+interactions+with+genetic%2C+ethnic+and+reproductive+factors | | | |
| 123 | Obesity Reviews | Louri N | 2008 | 0 | http://www.ncbi.nlm.nih.gov/pubmed/?term=toma+obstruction+after+laparoscopic+adjustable+gastric+banding+for+morbid+obesity%3A+report+of+two+cases+and+treatment+options. | | | |
| 118 | Journal of endocrinological investigation | Almawi W | 2008 | 0 | http://www.ncbi.nlm.nih.gov/pubmed/?term=Association+of+comorbid+depression%2C+anxiety%2C+and+stress+disorders+with+Type+2+diabetes+in+Bahrain%2C+a+country+with+a+very+high+prevalence+of+Type+2+diabetes. | | | |
| 121 | Journal of Postgraduate Medicine | Damanhori A | 2008 | 0 | http://www.ncbi.nlm.nih.gov/pubmed/18953141 | | | |
| 126 | The journal of school health | Moh'd Al-Mulla A | 2008 | 0 | http://www.ncbi.nlm.nih.gov.ezproxy.aub.edu.lb//pubmed/18489467 | | | |
| 130 | Clinical and Vaccine Immunology | Stayoussef M | 2008 | 0 | http://www.ncbi.nlm.nih.gov.ezproxy.aub.edu.lb/pubmed/?term=Modulation+of+type+1+diabetes+susceptibility+by+tumor+necrosis+factor+alpha+-308+G%2FA+and+lymphotoxin+alpha+%2B249+A%2FG+haplotypes+and+lack+of+linkage+disequilibrium+with+predisposing+DQB1-DRB1+haplotypes+in+Bahraini+patients. | | | |
| 124 | International Journal of Health Sciences | Saadat S | 2008 | 0 | http://www.ncbi.nlm.nih.gov/pubmed/21475500 | | | |
| 194 | Pakistan Journal of Biological Sciences | Musaiger AO | 2008 | 0 | http://www.ncbi.nlm.nih.gov/pubmed/18819593 | | | |
| 198 | Archivos latinoamericanos de nutrición | Musaiger AO | 2008 | 0 | http://www.ncbi.nlm.nih.gov/pubmed/18589580 | | | |
| 117 | Anthropologischer Anzeiger | Bader Z | 2008 | 0 | http://www.ncbi.nlm.nih.gov/pubmed/19216180 | | | |
| 125 | Hematology/Oncology and Stem Cell Therapy | Alsayyad J | 2008 | 0 | http://www.ncbi.nlm.nih.gov/pubmed/20063549 | | | |
| 127 | Saudi Medical Journal | Zubaid M | 2008 | 0 | http://www.ncbi.nlm.nih.gov.ezproxy.aub.edu.lb/pubmed/18246236 | | | |
| 102 | New England Journal of Medicine | Frangoul H | 2008 |  | http://www.ncbi.nlm.nih.gov/pubmed/?term=Shortage+of+chemotherapeutic+agents+in+Iraq+and+outcome+of+childhood+acute+lymphocytic+leukemia%2C+1990-2002. | | | |
| 107 | Iranian Journal of Immunology | Mohammed SN | 2008 |  | http://www.ncbi.nlm.nih.gov/pubmed/?term=Anticardiolipin+and+antiphospholipid+antibodies+in+Iraqi+patients+with+angina+pectoris. | | | |
| 90 | BMC Public Health | Abdulamir AS | 2008 | 0 | http://www.ncbi.nlm.nih.gov/pubmed/19055849 | | | |
| 91 | Journal of Carcinogenesis | Al-Dujaily EA | 2008 | 0 | http://www.ncbi.nlm.nih.gov/pubmed/19008567 | | | |
| 92 | Journal of Asthma | Ahmad Al Obaidi AH | 2008 | 0 | http://www.ncbi.nlm.nih.gov/pubmed/18951256 | | | |
| 97 | Oman Medical Journal | Hanna BE | 2008 | 0 | http://www.ncbi.nlm.nih.gov/pmc/articles/PMC3273920/#!po=15.6250 | | | |
| 98 | The Permanente Journal | Mansour AA | 2008 | 0 | http://www.ncbi.nlm.nih.gov/pubmed/?term=Are+foot+abnormalities+more+common+in+adults+with+diabetes%3F+A+cross-sectional+study+in+basrah%2C+iraq. | | | |
| 99 | Ultrastructural Pathology | Kummoona R | 2008 | 0 | http://www.ncbi.nlm.nih.gov/pubmed/?term=Proliferative+activity+in+oral+carcinomas+studied+with+Ag-NOR+and+electron+microscopy. | | | |
| 100 | Saudi Medical Journal | Al-Harris ES | 2008 | 0 | http://www.ncbi.nlm.nih.gov/pubmed/18690299 | | | |
| 101 | Diabetes Research and Clinical Practice | Almustafa M | 2008 | 0 | http://www.ncbi.nlm.nih.gov/pubmed/18687501 | | | |
| 103 | Conflict and Health | Mansour AA | 2008 | 0 | http://www.ncbi.nlm.nih.gov/pubmed/?term=Patients'+opinion+on+the+barriers+to+diabetes+control+in+areas+of+conflicts%3A+The+Iraqi+example. | | | |
| 104 | Journal of Immunotoxicology | Ali HY | 2008 | 0 | http://www.ncbi.nlm.nih.gov/pubmed/?term=Anti-beta(2)-glycoprotein+I+autoantibody+expression+as+a+potential+biomarker+for+strokes+in+patients+with+anti-phospholipid+syndrome. | | | |
| 106 | Oral surgery, oral medicine, oral pathology, oral radiology, and endodontics | Uthman AT | 2008 | 0 | http://www.ncbi.nlm.nih.gov/pubmed/?term=Prevalence+in+digital+panoramic+radiographs+of+carotid+area+calcification+among+Iraqi+individuals+with+stroke-related+disease. | | | |
| 109 | Journal of Clinical Hypertension | Al-Tamer YY | 2008 | 0 | http://www.ncbi.nlm.nih.gov/pubmed/18256577 | | | |
| 119 | Diabetes Research and Clinical Practice | Mansour AA | 2008 | 0 | http://www.ncbi.nlm.nih.gov/pubmed/?term=Diabetes+screening+in+Basrah%2C+Iraq%3A+a+population-based+cross-sectional+study. | | | |
| 120 | Clinical Oral Investigations | Al-Rawi NH | 2008 | 0 | http://www.ncbi.nlm.nih.gov/pubmed/?term=Squamous+cell+carcinoma+of+the+oral+cavity%3A+a+case+series+analysis+of+clinical+presentation+and+histological+grading+of+1%2C425+cases+from+Iraq. | | | |
| 105 | Journal of Asthma | Al Obaidi AH | 2008 | 0 | http://www.ncbi.nlm.nih.gov/pubmed/18569238 | | | |
| 110 | Journal of oral pathology and medicine | Kummoona R | 2008 | 0 | http://www.ncbi.nlm.nih.gov/pubmed/18248353 | | | |
| 108 | Diabetes Obesity and Metabolism | Khutsoane D | 2008 | 0 | http://www.ncbi.nlm.nih.gov/pubmed/?term=Biphasic+insulin+aspart+30+treatment+improves+glycaemic+control+in+patients+with+type+2+diabetes+in+a+clinical+practice+setting%3A+experience+from+the+PRESENT+study. | | | |
| 203 | Journal of Cardiovascular Pharmacology | Adam JK | 2008 | 0 | http://www.ncbi.nlm.nih.gov/pubmed/?term=A+double-blind+placebo-controlled+investigation+of+the+psychomotor+profile+of+clopidogrel+in+healthy+volunteers. | | | |
| 150 | Saudi Medical Journal | Ayad NM | 2008 |  | http://www.ncbi.nlm.nih.gov.ezproxy.aub.edu.lb/pubmed/?term=Biphasic+insulin+aspart+30+treatment+improves+glycemic+control+among+patients+with+type+2+diabetes+in+Saudi+Arabia+and+the+Gulf+region | | | |
| 389 | Saudi Medical Journal | Al-Adsani A | 2008 |  | http://www.ncbi.nlm.nih.gov/pubmed/18998023 | | | |
| 372 | The Gulf Journal of Oncology | Al Hendal A | 2008 | 0 | http://www.ncbi.nlm.nih.gov/pubmed/20084779 | | | |
| 428 | Clinical Medicine Oncology | Al-Khashnam H | 2008 | 0 | http://www.ncbi.nlm.nih.gov/pubmed/21892304 | | | |
| 660 | Medical Principles and Practice | Mittal R | 2008 | 0 | http://www.ncbi.nlm.nih.gov.ezproxy.aub.edu.lb//pubmed/18059108 | | | |
| 729 | World Journal of Surgical Oncology | Sultan M | 2008 | 0 | http://www.ncbi.nlm.nih.gov.ezproxy.aub.edu.lb/pubmed/18664291 | | | |
| 754 | Cancer Genet Cytogenet | Zamecnikova A | 2008 | 0 | http://www.ncbi.nlm.nih.gov/pubmed/18503829 | | | |
| 755 | Leukemia Research | Zamecnikova A | 2008 | 0 | http://www.ncbi.nlm.nih.gov/pubmed/18294688 | | | |
| 871 | Medical Principles and Practice | Al-Brahim N | 2008 | 0 | http://www.ncbi.nlm.nih.gov/pubmed/18523405 | | | |
| 880 | Acta Cytologica | Das DK | 2008 | 0 | http://www.ncbi.nlm.nih.gov/pubmed/18540301 | | | |
| 902 | Saudi Medical Journal | Zubaid M | 2008 | 0 | http://www.ncbi.nlm.nih.gov/pubmed/18246236 | | | |
| 36 | Urology | Al-Hunayan A | 2008 | 0 | http://www.ncbi.nlm.nih.gov.ezproxy.aub.edu.lb/pubmed/?term=Hyperhomocysteinemia+is+a+risk+factor+for+erectile+dysfunction+in+men+with+++++++adult-onset+diabetes+mellitus. | | | |
| 39 | Medical Principles and Practice | Matowe WC | 2008 | 0 | http://www.ncbi.nlm.nih.gov.ezproxy.aub.edu.lb/pubmed/?term=Self-monitoring+of+blood+pressure+and+the+role+of+community+pharmacists+in+++++++Kuwait | | | |
| 40 | Angiology | Qadan LR | 2008 | 0 | http://www.ncbi.nlm.nih.gov/pubmed/?term=Prevalence+of+metabolic+syndrome+in+patients+with+clinically+advanced+peripheral++++++++vascular+disease | | | |
| 113 | Annals of Nutrition and Metabolism | Al Mutairi SS | 2008 | 0 | http://www.karger.com.ezproxy.aub.edu.lb/Article/Pdf/151487 | | | |
| 148 | Medical Principles and Practice | Awad A | 2008 | 0 | http://www.ncbi.nlm.nih.gov.ezproxy.aub.edu.lb/pubmed/?term=Self-medication+practices+among+diabetic+patients+in+Kuwait | | | |
| 187 | Nutrition metabolism and Cardiovascular Diseases | Mojiminiyi OA | 2008 | 0 | http://www.ncbi.nlm.nih.gov.ezproxy.aub.edu.lb/pubmed/?term=Body+iron+stores+in+relation+to+the+metabolic+syndrome%2C+glycemic+control+and+complications+in+female+patients+with+type+2+diabetes | | | |
| 340 | Endocrine Journal | Abbas JM | 2008 | 0 | http://www.ncbi.nlm.nih.gov/pubmed/18385530 | | | |
| 351 | The Gulf Journal of Oncology | Abuzallouf S | 2008 | 0 | http://www.ncbi.nlm.nih.gov/pubmed/20084796 | | | |
| 383 | The Gulf Journal of Oncology | Al Wakiel H | 2008 | 0 | http://www.ncbi.nlm.nih.gov/pubmed/20084775 | | | |
| 392 | Medical Principles and Practice | Al-Awadhi AM | 2008 | 0 | http://www.ncbi.nlm.nih.gov/pubmed/18287800 | | | |
| 396 | Medical Principles and Practice | Al-Bader WR | 2008 | 0 | http://www.ncbi.nlm.nih.gov/pubmed/18059096 | | | |
| 425 | Nursing & Health sciences | Al-Kandari F | 2008 | 0 | http://www.ncbi.nlm.nih.gov/pubmed/18257831 | | | |
| 438 | Journal of Clinical Pathology | Al-Mulla F | 2008 | 0 | http://www.ncbi.nlm.nih.gov/pubmed/18375747 | | | |
| 507 | Nuclear Medicine Communications | Ballani NS | 2008 | 0 | http://www.ncbi.nlm.nih.gov/pubmed/18458599 | | | |
| 551 | American Journal of Clinical Pathology | Doi SA | 2008 | 0 | http://www.ncbi.nlm.nih.gov/pubmed/18426742 | | | |
| 581 | Emergency Medicine Journal | Gonnah R | 2008 | 0 | http://www.ncbi.nlm.nih.gov.ezproxy.aub.edu.lb/pubmed/?term=Can+a+change+in+policy+reduce+emergency+hospital+admissions%3F+Effect+of+admission+avoidance+team%2C+guideline+implementation+and+maximising+the+observation+unit. | | | |
| 617 | Acta Cytologica | Kapila K | 2008 | 0 | http://www.ncbi.nlm.nih.gov.ezproxy.aub.edu.lb/pubmed/?term=Fine+needle+aspiration+cytology+of+breast+masses+in+children+and+adolescents%3A+experience+with+1404+aspirates. | | | |
| 618 | Cytopathology | Kapila K | 2008 | 0 | http://www.ncbi.nlm.nih.gov.ezproxy.aub.edu.lb/pubmed/?term=Could+nuclear+matrix+protein+22+(NMP22)+play+a+role+with+urine+cytology+in+screening+for+bladder+cancer%3F--experience+at+Kuwait+University. | | | |
| 661 | The Journal of School Health | Moh'd Al-Mulla A | 2008 | 0 | http://www.ncbi.nlm.nih.gov.ezproxy.aub.edu.lb//pubmed/18489467 | | | |
| 664 | Medical Principles and Practice | Mojiminiyi OA | 2008 | 0 | http://www.ncbi.nlm.nih.gov/pubmed/?term=Determinants+and+associations+of+homocysteine+and+prothrombotic+risk+factors+in+Kuwaiti+patients+with+cerebrovascular+accident. | | | |
| 689 | Medical Principles and Practice | Owayed A | 2008 | 0 | http://www.karger.com/Article/Pdf/129607 | | | |
| 694 | Annals of Saudi medicine | Qasem JA | 2008 | 0 | http://www.ncbi.nlm.nih.gov/pubmed/?term=Meteorological+factors%2C+aeroallergens+and+asthma-related+visits+in+Kuwait%3A+a+12-month+retrospective+study | | | |
| 707 | Journal of Environmental Pathology, Toxicology and Oncology | Saleh F | 2008 | 0 | http://www.ncbi.nlm.nih.gov.ezproxy.aub.edu.lb/pubmed/?term=The+first+pilot+study+on+characteristics+and+practice+patterns+of+Kuwaiti+breast+cancer+patients | | | |
| 708 | Neoplasma | Saleh F | 2008 | 0 | http://www.ncbi.nlm.nih.gov.ezproxy.aub.edu.lb/pubmed/?term=Invasive+cribriform+breast+carcinomas+in+patients+with+grade+1+and+stage+IIA+(T2+N0+M0)+breast+cancer+strongly+express+the+v3+and+v6%2C+but+not+the+v4+isoforms+of+the+metastatic+marker+CD44 | | | |
| 473 | Journal of the Egyptian National Cancer Institute | Alawadi S | 2008 | 0 | http://www.ncbi.nlm.nih.gov/pubmed/20424658 | | | |
| 691 | Journal of Carcinogenesis | Palmieri B | 2008 | 0 | http://www.ncbi.nlm.nih.gov/pmc/articles/PMC2669724/?report=classic | | | |
| 744 | The Gulf Journal of Oncology | Usmani S | 2008 | 0 | http://www.ncbi.nlm.nih.gov.ezproxy.aub.edu.lb/pubmed/?term=Functional+breast+imaging+with+Tc+99m+Mibi+for+detection+of+primary+breast+lesion+and+axillary+lymph+node+metastases | | | |
| 901 | Journal of Cardiovascular Medicine (Hagerstown) | Zubaid M | 2008 | 0 | http://www.ncbi.nlm.nih.gov/pubmed/18545068 | | | |
| 37 | The Scientific World Journal | Sharma JN | 2008 | 0 | http://www.ncbi.nlm.nih.gov/pubmed/18454246 | | | |
| 398 | The Gulf Journal of Oncology | Al-Bahar S | 2008 | 0 | http://www.ncbi.nlm.nih.gov/pubmed/20084792 | | | |
| 565 | Eastern Medditerranian Health Journal | El-Sabban F | 2008 | 0 | http://www.ncbi.nlm.nih.gov/pubmed/18557469 | | | |
| 570 | The Gulf Journal of Oncology | Elqazzar AH | 2008 | 0 | http://www.ncbi.nlm.nih.gov.ezproxy.aub.edu.lb/pubmed/20084793 | | | |
| 623 | The Canadian Journal of Urology | Kehinde EO | 2008 | 0 | http://www.ncbi.nlm.nih.gov.ezproxy.aub.edu.lb/pubmed/18405443 | | | |
| 681 | Neoplasma | Novotny L | 2008 | 0 | http://www.ncbi.nlm.nih.gov/pubmed/18237244 | | | |
| 700 | The Gulf Journal of Oncology | Ramamoorthy K | 2008 | 0 | http://www.ncbi.nlm.nih.gov/pubmed/?term=Primary+treatment+of+acute+myeloid+leukemia+(non+M3)+in+elderly%3A+a+review | | | |
| 704 | International Journal of Health Sciences | Saadat S | 2008 | 0 | http://www.ncbi.nlm.nih.gov/pmc/articles/PMC3068727/pdf/ijhs-2-2-0167.pdf | | | |
| 862 | The Gulf Journal of Oncology | Al-Bahar S | 2008 | 0 | http://www.ncbi.nlm.nih.gov/pubmed/20084792 | | | |
| 35 | Vascular Pharmacology | Yousif MH | 2008 | 0 | http://www.ncbi.nlm.nih.gov.ezproxy.aub.edu.lb/pubmed/?term=Inhibition+of+Ras-GTPase+signaling+by+FPTIII+ameliorates+development+of+++++++cardiovascular+dysfunction+in+diabetic-hypertensive+rats. | | | |
| 41 | Cell Biochemistry and Function | Yousif MH | 2008 | 0 | http://www.ncbi.nlm.nih.gov/pubmed/?term=Role+of+Ca2%2B%2Fcalmodulin-dependent+protein+kinase+II+in+development+of+vascular+++++++dysfunction+in+diabetic+rats+with+hypertension | | | |
| 43 | American Journal of Nephrology | Benter IF | 2008 | 0 | http://www.ncbi.nlm.nih.gov/pubmed/17890855 | | | |
| 142 | BMC Medical Physics | Al-Saeedi FJ | 2008 | 0 | http://www.ncbi.nlm.nih.gov/pubmed/18559077 | | | |
| 176 | Experimental Diabetes Research | Malatiali S | 2008 | 0 | http://www.ncbi.nlm.nih.gov/pubmed/?term=Phlorizin+prevents+glomerular+hyperfiltration+but+not+hypertrophy+in+diabetic+rats | | | |
| 201 | British Journal of Nutrition | Renno WM | 2008 | 0 | http://www.ncbi.nlm.nih.gov/pubmed/?term=Effect+of+green+tea+on+kidney+tubules+of+diabetic+rats | | | |
| 204 | Pediatric Diabetes | Robertson K | 2008 | 0 | http://www.ncbi.nlm.nih.gov/pubmed/?term=Exercise+in+children+and+adolescents+with+diabetes+dasman | | | |
| 218 | Cell Biochemistry and Function | Yousif MH | 2008 | 0 | http://www.ncbi.nlm.nih.gov/pubmed/?term=Phosphoinositide+3-kinase+contributes+to+diabetes-induced+abnormal+vascular+reactivity+in+rat+perfused+mesenteric+bed | | | |
| 483 | Archives of Medical Research | Alkhalaf M | 2008 | 0 | http://www.ncbi.nlm.nih.gov/pubmed/18164959 | | | |
| 688 | Cell Biochemistry and Function | Owayed A | 2008 | 0 | http://onlinelibrary.wiley.com/doi/10.1002/cbf.1484/pdf | | | |
| 492 | Hematology | AlShemmari SH | 2008 | 0 | http://www.ncbi.nlm.nih.gov/pubmed/18702874 | | | |
| 534 | Diagnostic Cytopathology | Das DK | 2008 | 0 | http://www.ncbi.nlm.nih.gov/pubmed/18831027 | | | |
| 174 | Diabetes and Metabolism | Khutsoane D | 2008 | 0 | http://www.ncbi.nlm.nih.gov/pubmed/?term=iphasic+insulin+aspart+30+treatment+improves+glycaemic+control+in+patients+with+type+2+diabetes+in+a+clinical+practice+setting%3A+experience+from+the+PRESENT+study | | | |
| 193 | Medical Principles and Practice | Moussa MA | 2008 | 0 | http://www.ncbi.nlm.nih.gov.ezproxy.aub.edu.lb/pubmed/18523392 | | | |
| 418 | Eastern Mediterranean Health Journal | Al-Isa AN | 2008 | 0 | http://www.ncbi.nlm.nih.gov/pubmed/18561725 | | | |
| 692 | Indian Journal of Medical Sciences | Panicker NR | 2008 | 0 | http://www.indianjmedsci.org/article.asp?issn=0019-5359;year=2008;volume=62;issue=1;spage=1;epage=7;aulast=Panicker | | | |
| 385 | Medical Principles and Practice | Al-Adsani A | 2008 | 0 | http://www.ncbi.nlm.nih.gov/pubmed/18059095 | | | |
| 495 | Clinical lymphoma and myeloma | Ameen RM | 2008 | 0 | http://www.ncbi.nlm.nih.gov/pubmed/18501104 | | | |
| 147 | Annals of Saudi Medicine | Al-Shoumer KA | 2008 | 0 | http://www.ncbi.nlm.nih.gov/pubmed/?term=Serum+leptin+and+its+relationship+with+metabolic+variables+in+Arabs+with+type+2+diabetes+mellitus | | | |
| 498 | The Gulf Journal of Oncology | Arora R | 2008 | 0 | http://www.ncbi.nlm.nih.gov/pubmed/20084773 | | | |
| 338 | World Journal of Gastroenterology | Abaza MS | 2008 | 0 | http://www.ncbi.nlm.nih.gov/pubmed/18777593 | | | |
| 339 | Tumour Biology | Abaza MS | 2008 | 0 | http://www.ncbi.nlm.nih.gov/pubmed/18802399 | | | |
| 94 | J Med Liban | Berbari A E | 2008 |  | Not Found | |  |  |
| 119 | Revue des maladies respiratoires | Chaouachi K | 2008 |  | Not Found | |  |  |
| 134 | The Journal of urology | Chouairy C J | 2008 |  | Not Found | |  |  |
| 138 | J Med Liban | Daher M | 2008 |  | Not Found | |  |  |
| 450 | ONS connect | Saca-Hazboun H | 2008 |  | Abstract Not Found | |  |  |
| 463 | Dermatology | Saleh F H | 2008 |  | Abstract Not Found | |  |  |
| 481 | Hematology/oncology and stem cell therapy | Seoud M | 2008 |  | Abstract Not Found | |  |  |
| 211 | Respirology | Fares M | 2008 | 0 | http://www.ncbi.nlm.nih.gov/pubmed/?term=Metastases+in+malignant+pleural+mesothelioma%3A+a+new+radiological+appearance | | | |
| 214 | European journal of gynaecological oncology | Farhat F | 2008 | 0 | http://www.ncbi.nlm.nih.gov/pubmed/?term=A+case+of+synchronous+relapse+of+breast+cancer+and+uterine+mullerian+adenosarcoma+post+tamoxifen+in+a+premenopausal+woman | | | |
| 319 | Journal of clinical neuroscience | Karam C | 2008 | 0 | http://www.ncbi.nlm.nih.gov/pubmed/?term=Cerebral+dural+sinus+thrombosis+following+cisplatin+chemotherapy+karam+c+2008 | | | |
| 329 | Transfusion | Kfoury-Baz E M | 2008 | 0 | http://www.ncbi.nlm.nih.gov/pubmed/?term=kfoury+2008+Plasmapheresis+in+asparaginase-induced+hypertriglyceridemia | | | |
| 340 | International journal of dermatology | Kurban M | 2008 | 0 | http://www.ncbi.nlm.nih.gov/pubmed/?term=Acquired+perforating+dermatosis+heralding+metastatic+renal+cell+carcinoma+to+the+liver | | | |
| 366 | Dermatology online journal | Moussallem C D | 2008 | 0 | http://www.ncbi.nlm.nih.gov/pubmed/?term=Malignant+porocarcinoma+of+the+nail+fold%3A+a+tricky+diagnosis | | | |
| 370 | Cases journal | Musallam K M | 2008 | 0 | http://www.ncbi.nlm.nih.gov/pubmed/?term=Solitary+mediastinal+lymph+node+metastasis+in+rectosigmoid+carcinoma%3A+a+case+report | | | |
| 423 | Journal of medical case reports | Otrock Z K | 2008 | 0 | http://www.ncbi.nlm.nih.gov/pubmed/?term=Non-Hodgkin's+lymphoma+in+a+woman+with+adult-onset+Still's+disease%3A+a+case+report | | | |
| 430 | Journal of pediatric hematology/oncology | Rammal H | 2008 | 0 | http://www.ncbi.nlm.nih.gov/pubmed/?term=Multifocal+Ewing+sarcoma+of+the+foot+rammal | | | |
| 435 | BMC neurology | Riachy M | 2008 | 0 | http://www.ncbi.nlm.nih.gov/pubmed/?term=Prediction+of+the+survival+and+functional+ability+of+severe+stroke+patients+after+ICU+therapeutic+intervention | | | |
| 514 | Pathology, research and practice | Tawil A | 2008 | 0 | http://www.ncbi.nlm.nih.gov/pubmed/?term=Periosteal+osteoblastoma+of+the+calvaria+mimicking+a+meningioma | | | |
| 517 | Cases journal | Tohfe M | 2008 | 0 | http://www.ncbi.nlm.nih.gov/pubmed/?term=tohfe+Metastatic+prostate+adenocarcinoma+presenting+with+pulmonary+symptoms%3A+a+case+report+and+review+of+the+literature | | | |
| 607 | European journal of gynaecological oncology | Farhat F | 2008 | 0 | http://www.ncbi.nlm.nih.gov/pubmed/?term=A+case+of+synchronous+relapse+of+breast+cancer+and+uterine+m%D8%A3%C2%BCllerian+adenosarcoma+post+tamoxifen+in+a+premenopausal+woman. | | | |
| 632 | Neuropediatrics | Hourani R | 2008 | 0 | http://www.ncbi.nlm.nih.gov/pubmed/18504683 | | | |
| 694 | Dermatology | Saleh Z | 2008 | 0 | http://www.ncbi.nlm.nih.gov/pubmed/18714160 | | | |
| 983 | Southern medical journal | Hatoum HA | 2008 | 0 | http://www.ncbi.nlm.nih.gov/pubmed/19209120 | | | |
| 1006 | Pathology | Mahfouz RA | 2008 | 0 | http://www.ncbi.nlm.nih.gov/pubmed/18038331 | | | |
| 1030 | Annals of hematology | Otrock ZK | 2008 | 0 | http://www.ncbi.nlm.nih.gov/pubmed/18587577 | | | |
| 1032 | Intern Emerg Med | Otrock ZK | 2008 | 0 | http://www.ncbi.nlm.nih.gov/pubmed/18270789 | | | |
| 16 | J Med Liban | Abdel-Massih T | 2008 | 0 | http://www.ncbi.nlm.nih.gov/pubmed/?term=Epidemiology+of+cardiac+tumors+in+adults+in+Lebanon | | | |
| 85 | Journal of clinical gastroenterology | Barada K | 2008 | 0 | http://www.ncbi.nlm.nih.gov/pubmed/?term=Upper+gastrointestinal+bleeding+in+patients+with+acute+coronary+syndromes%3A+clinical+predictors+and+prophylactic+role+of+proton+pump+inhibitors | | | |
| 98 | Clinical lymphoma & myeloma | Birjawi G A | 2008 | 0 | http://www.ncbi.nlm.nih.gov/pubmed/?term=Abdominal+manifestations+of+multiple+myeloma%3A+a+retrospective+radiologic+overview | | | |
| 118 | Angiology | Chammas E | 2008 | 0 | http://www.ncbi.nlm.nih.gov/pubmed/?term=Myocardial+perfusion+in+patients+with+a+totally+occluded+left+anterior+descending+coronary+artery+reinjected+by+a+normal+right+coronary+artery%3A+the+role+of+collateral+circulation | | | |
| 159 | Annals of Saudi medicine | Dib J G | 2008 | 0 | http://www.ncbi.nlm.nih.gov/pubmed/18299654 | | | |
| 195 | Nicotine Tob Res | El-Roueiheb Z | 2008 | 0 | http://www.ncbi.nlm.nih.gov/pubmed/?term=Cigarette+and+waterpipe+smoking+among+Lebanese+adolescents%2C+a+cross-sectional+study%2C+2003-2004 | | | |
| 223 | Journal of oncology | Farhat M H | 2008 | 0 | http://www.ncbi.nlm.nih.gov/pubmed/?term=Small+bowel+tumors%3A+clinical+presentation%2C+prognosis%2C+and+outcome+in+33+patients+in+a+tertiary+care+center | | | |
| 224 | World journal of gastroenterology | Farhat M H | 2008 | 0 | http://www.ncbi.nlm.nih.gov/pubmed/?term=Prognostic+factors+in+patients+with+advanced+cholangiocarcinoma%3A+role+of+surgery%2C+chemotherapy+and+body+mass+index | | | |
| 239 | European journal of endocrinology | Gannage-Yared M H | 2008 | 0 | http://www.ncbi.nlm.nih.gov/pubmed/?term=Osteoprotegerin+in+relation+to+body+weight%2C+lipid+parameters+insulin+sensitivity%2C+adipocytokines%2C+and+C-reactive+protein+in+obese+and+non-obese+young+individuals%3A+results+from+both+cross-sectional+and+inter | | | |
| 264 | Journal of cardiothoracic and vascular anesthesia | Haddad F | 2008 | 0 | http://www.ncbi.nlm.nih.gov/pubmed/?term=Can+femoral+artery+pressure+monitoring+be+used+routinely+in+cardiac+surgery%3F | | | |
| 271 | Pediatric blood & cancer | Haidar R | 2008 | 0 | http://www.ncbi.nlm.nih.gov/pubmed/?term=Limb+salvage+surgery+for+children+and+adolescents+with+malignant+bone+tumors+in+a+developing+country | | | |
| 303 | Scottish medical journal | Jabbour R | 2008 | 0 | http://www.ncbi.nlm.nih.gov/pubmed/?term=Early+neurologic+complications+following+coronary+bypass+surgery+Jabbour+R+2008 | | | |
| 372 | International journal of public health | Musharrafieh U | 2008 | 0 | http://www.ncbi.nlm.nih.gov/pubmed/?term=Determinants+of+university+students+physical+exercise%3A+a+study+from+Lebanon | | | |
| 416 | Am J of critical care | Noureddine S | 2008 | 0 | http://www.ncbi.nlm.nih.gov/pubmed/?term=Response+to+signs+and+symptoms+of+acute+coronary+syndrome%3A+differences+between+Lebanese+men+and+women+noureddine | | | |
| 419 | Biological trace element research | Obeid O | 2008 | 0 | http://www.ncbi.nlm.nih.gov/pubmed/?term=Plasma+copper%2C+zinc%2C+and+selenium+levels+and+correlates+with+metabolic+syndrome+components+of+lebanese+adults | | | |
| 434 | Revue des maladies respiratoires | Riachy M | 2008 | 0 | http://www.ncbi.nlm.nih.gov/pubmed/?term=Are+narghile+smokers+different+from+cigarette+smokers%3F+2007+riachy | | | |
| 443 | Eastern Mediterranean health journal | Saade G | 2008 | 0 | http://www.ncbi.nlm.nih.gov/pubmed/?term=Patterns+of+tobacco+use%3A+results+from+the+2005+Global+Youth+Tobacco+Survey+in+Lebanon | | | |
| 445 | Preventive medicine | Saade G | 2008 | 0 | http://www.ncbi.nlm.nih.gov/pubmed/?term=Linking+Global+Youth+Tobacco+Survey+(GYTS)+data+to+the+WHO+Framework+Convention+on+Tobacco+Control+(FCTC)%3A+the+case+for+Lebanon | | | |
| 449 | Genetic testing | Sabbagh A S | 2008 | 0 | http://www.ncbi.nlm.nih.gov/pubmed/?term=High+prevalence+of+MTHFR+gene+A1298C+polymorphism+in+Lebanon | | | |
| 457 | Nicotine Tob Res | Salameh P | 2008 | 0 | http://www.ncbi.nlm.nih.gov/pubmed/?term=Waterpipe+smoking%3A+construction+and+validation+of+the+Lebanon+Waterpipe+Dependence+Scale+(LWDS-11) | | | |
| 527 | Public Health | Waked M | 2008 | 0 | http://www.ncbi.nlm.nih.gov/pubmed/18313092 | | | |
| 531 | Nutrition journal | Yahia N | 2008 | 0 | http://www.ncbi.nlm.nih.gov/pubmed/18973661 | | | |
| 533 | Journal of cardiothoracic and vascular anesthesia | Yazigi A | 2008 | 0 | http://www.ncbi.nlm.nih.gov/pubmed/?term=Comparison+of+central+venous+to+mixed+venous+oxygen+saturation+in+patients+with+low+cardiac+index+and+filling+pressures+after+coronary+artery+surgery | | | |
| 538 | Journal of epidemiology and community health | Zabaneh J E | 2008 | 0 | http://www.ncbi.nlm.nih.gov/pubmed/?term=Living+and+health+conditions+of+Palestinian+refugees+in+an+unofficial+camp+in+the+Lebanon%3A+a+cross-sectional+survey | | | |
| 655 | Scandinavian journal of clinical and laboratory investigation | Mahfouz RA | 2008 | 0 | http://www.ncbi.nlm.nih.gov.ezproxy.aub.edu.lb/pubmed/19378435 | | | |
| 719 | J Asthma Allergy | Waked M | 2008 | 0 | http://www.ncbi.nlm.nih.gov/pubmed/21437138 | | | |
| 733 | Molecular biology reports | Mahfouz RA | 2008 | 0 | http://www.ncbi.nlm.nih.gov/pubmed/?term=Factor+XIII+gene+V34L+mutation+in+the+Lebanese+population%3A+another+unique+feature+in+this+community%3F | | | |
| 988 | Am J Cardiol | Isma'eel H | 2008 | 0 | http://www.ncbi.nlm.nih.gov/pubmed/18638603 | | | |
| 1029 | JOP | Noun R | 2008 | 0 | http://www.ncbi.nlm.nih.gov/pubmed/18648138 | | | |
| 1045 | BJOG | Tamim H | 2008 | 0 | http://www.ncbi.nlm.nih.gov/pubmed/18053105 | | | |
| 83 | Nuclear medicine communications | Ballani N S | 2008 | 0 | http://www.ncbi.nlm.nih.gov/pubmed/?term=Role+of+serial+quantitative+gallium-67+tumor+uptake+in+assessing+response+rates+for+chemotherapy+in+lymphoma+patients | | | |
| 254 | Cancer chemotherapy and pharmacology | Ghosn M | 2008 | 0 | http://www.ncbi.nlm.nih.gov/pubmed/?term=Sequential+vinorelbine-capecitabine+followed+by+docetaxel+in+advanced+breast+cancer%3A+long-term+results+of+a+pilot+phase+II+trial | | | |
| 284 | Annals of nutrition & metabolism | Helou N | 2008 | 0 | http://www.ncbi.nlm.nih.gov/pubmed/?term=Variation+of+postprandial+PYY+3-36+response+following+ingestion+of+differing+macronutrient+meals+in+obese+females | | | |
| 327 | Investigational new drugs | Kattan J G | 2008 | 0 | http://www.ncbi.nlm.nih.gov/pubmed/?term=Weekly+docetaxel%2C+zoledronic+acid+and+estramustine+in+hormone-refractory+prostate+cancer+(HRPC) | | | |
| 89 | Bone Marrow Transplant | Bazarbachi A | 2008 | 0 | http://www.ncbi.nlm.nih.gov/pubmed/?term=Hematopoietic+stem+cell+transplantation+in+Lebanon%3A+first+comprehensive+report | | | |
| 175 | Cancer | El Saghir N S | 2008 | 0 | http://www.ncbi.nlm.nih.gov/pubmed/?term=Locally+advanced+breast+cancer%3A+treatment+guideline+implementation+with+particular+attention+to+low-+and+middle-income+countries | | | |
| 339 | Clinical Therapeutics | Kobeissy A | 2008 | 0 | http://www.ncbi.nlm.nih.gov/pubmed/?term=Suggested+insulin+regimens+for+patients+with+type+1+diabetes+mellitus+who+wish+to+fast+during+the+month+of+Ramadan | | | |
| 378 | J Med Liban | Naccache N | 2008 | 0 | http://www.ncbi.nlm.nih.gov/pubmed/?term=Pain+management+and+health+care+policy+naccache | | | |
| 388 | Tobacco control | Nakkash R | 2008 | 0 | http://www.ncbi.nlm.nih.gov/pubmed/?term=Smuggling+as+the+%22key+to+a+combined+market%22%3A+British+American+Tobacco+in+Lebanon+nakkash | | | |
| 439 | Paediatric drugs | Saab R | 2008 | 0 | http://www.ncbi.nlm.nih.gov/pubmed/?term=Epidemiology+and+management+options+for+colorectal+cancer+in+children+saab+2008 | | | |
| 544 | Diabetes Research and Clinical Practice | Ziyadeh F N | 2008 | 0 | http://www.ncbi.nlm.nih.gov/pubmed/?term=Different+roles+for+TGF-beta+and+VEGF+in+the+pathogenesis+of+the+cardinal+features+of+diabetic+nephropathy | | | |
| 545 | Current diabetes reviews | Ziyadeh F N | 2008 | 0 | http://www.ncbi.nlm.nih.gov/pubmed/18220694 | | | |
| 546 | Current opinion in obstetrics & gynecology | Zreik T G | 2008 | 0 | http://www.ncbi.nlm.nih.gov/pubmed/?term=Fertility+drugs+and+risk+of+ovarian+cancer%3A+dispelling+the+myth | | | |
| 978 | J Cell Mol Med | Gali-Muhtasib H | 2008 | 0 | http://www.ncbi.nlm.nih.gov/pubmed/18366456 | | | |
| 142 | J Med Liban | Daher M | 2008 | 0 | http://www.ncbi.nlm.nih.gov/pubmed/?term=Implementation+of+palliative+care+in+Lebanon%3A+past%2C+present%2C+and+future | | | |
| 191 | Oncology reports | El-Najjar N | 2008 | 0 | http://www.ncbi.nlm.nih.gov/pubmed/?term=Anti-colon+cancer+effects+of+Salograviolide+A+isolated+from+Centaurea+ainetensis | | | |
| 275 | Chemico-biological interactions | Hammud H H | 2008 | 0 | http://www.ncbi.nlm.nih.gov/pubmed/?term=Copper-adenine+complex%2C+a+compound%2C+with+multi-biochemical+targets+and+potential+anti-cancer+effect | | | |
| 277 | Medical oncology | Harakeh S | 2008 | 0 | http://www.ncbi.nlm.nih.gov/pubmed/?term=Epigallocatechin-3-gallate+induces+apoptosis+and+cell+cycle+arrest+in+HTLV-1-positive+and+-negative+leukemia+cells | | | |
| 283 | Radiotherapy and oncology | Haykal J | 2008 | 0 | http://www.ncbi.nlm.nih.gov/pubmed/?term=Radiosensitization+of+EMT6+mammary+carcinoma+cells+by+2-benzoyl-3-phenyl-6%2C7-dichloroquinoxaline+1%2C4-dioxide | | | |
| 300 | Cancer biology & therapy | Itani W S | 2008 | 0 | http://www.ncbi.nlm.nih.gov/pubmed/?term=Anti+colon+cancer+components+from+Lebanese+sage+(Salvia+libanotica)+essential+oil%3A+Mechanistic+basis | | | |
| 348 | Annals of biomedical engineering | Maasrani M | 2008 | 0 | http://www.ncbi.nlm.nih.gov/pubmed/?term=Analog+electrical+model+of+the+coronary+circulation+in+case+of+multiple+revascularizations | | | |
| 429 | Prostaglandins & other lipid mediators | Panjarian S | 2008 | 0 | http://www.ncbi.nlm.nih.gov/pubmed/?term=De+novo+N-palmitoylsphingosine+synthesis+is+the+major+biochemical+mechanism+of+ceramide+accumulation+following+p53+up-regulation | | | |
| 522 | J Med Liban | Tueni E | 2008 | 0 | http://www.ncbi.nlm.nih.gov/pubmed/?term=2008+tueni+Perspectives+in+palliative+care%3F | | | |
| 539 | Human molecular genetics | Zalloua P A | 2008 | 0 | http://www.ncbi.nlm.nih.gov/pubmed/?term=WFS1+mutations+are+frequent+monogenic+causes+of+juvenile-onset+diabetes+mellitus+in+Lebanon | | | |
| 609 | Cancer Research | Gali-Muhtasib H | 2008 | 0 | http://www.ncbi.nlm.nih.gov/pubmed/?term=Thymoquinone+triggers+inactivation+of+the+stress+response+pathway+sensor+CHEK1+and+contributes+to+apoptosis+in+colorectal+cancer+cells. | | | |
| 620 | Leukemia | Haddad L | 2008 | 0 | http://www.ncbi.nlm.nih.gov/pubmed/?term=KSHV-transformed+primary+effusion+lymphoma+cells+induce+a+VEGF-dependent+angiogenesis+and+establish+functional+gap+junctions+with+endothelial+cells. | | | |
| 653 | Journal of ethnopharmacology | Loizzo MR | 2008 | 0 | http://www.ncbi.nlm.nih.gov.ezproxy.aub.edu.lb/pubmed/18601990 | | | |
| 741 | Molecular biology reports | Shammaa DM | 2008 | 0 | http://www.ncbi.nlm.nih.gov/pubmed/17578681 | | | |
| 742 | Molecular biology reports | Shammaa DM | 2008 | 0 | http://www.ncbi.nlm.nih.gov/pubmed/17497226 | | | |
| 945 | J Palliat Med | Huijer HA | 2008 | 0 | http://www.ncbi.nlm.nih.gov/pubmed/19021476 | | | |
| 963 | J Am Coll Cardiol | Dakik HA | 2008 | 0 | http://www.ncbi.nlm.nih.gov/pubmed/18582642 | | | |
| 114 | Cancer genomics & proteomics | Chalabi N | 2008 | 0 | http://www.ncbi.nlm.nih.gov/pubmed/?term=Comparative+clinical+and+transcriptomal+profiles+of+breast+cancer+between+French+and+South+Mediterranean+patients+show+minor+but+significative+biological+differences | | | |
| 157 | PLoS medicine | Degenhardt L | 2008 | 0 | http://www.ncbi.nlm.nih.gov/pubmed/?term=Toward+a+global+view+of+alcohol%2C+tobacco%2C+cannabis%2C+and+cocaine+use%3A+findings+from+the+WHO+World+Mental+Health+Surveys | | | |
| 506 | Clinical nutrition | Sorensen J | 2008 | 0 | http://www.ncbi.nlm.nih.gov/pubmed/?term=EuroOOPS%3A+an+international%2C+multicentre+study+to+implement+nutritional+risk+screening+and+evaluate+clinical+outcome | | | |
| 715 | Int J Oral Maxillofac Implants | Tawil G | 2008 | 0 | http://www.ncbi.nlm.nih.gov/pubmed/18807573 | | | |
| 338 | Diabetes, obesity & metabolism | Khutsoane D | 2008 | 0 | http://www.ncbi.nlm.nih.gov/pubmed/?term=Biphasic+insulin+aspart+30+treatment+improves+glycaemic+control+in+patients+with+type+2+diabetes+in+a+clinical+practice+setting%3A+experience+from+the+PRESENT+study | | | |
| 497 | International journal of cardiology | Sibai A M | 2008 | 0 | http://www.ncbi.nlm.nih.gov/pubmed/?term=Coronary+angiography+in+Lebanon%3A+Use+and+overuse | | | |
| 498 | Health policy and planning | Sibai A M | 2008 | 0 | http://www.ncbi.nlm.nih.gov/pubmed/?term=The+appropriateness+of+use+of+coronary+angiography+in+Lebanon%3A+implications+for+health+policy | | | |
| 261 | Leukemia | Habib A | 2008 | 0 | http://www.ncbi.nlm.nih.gov/pubmed/?term=Arsenic+trioxide+inhibits+ATRA-induced+prostaglandin+E2+and+cyclooxygenase-1+in+NB4+cells%2C+a+model+of+acute+promyelocytic+leukemia | | | |
| 95 | Obesity surgery | Biagini J | 2008 | 0 | http://www.ncbi.nlm.nih.gov/pubmed/18365290 | | | |
| 168 | J Med Liban | El Bcheraoui C | 2008 | 0 | http://www.ncbi.nlm.nih.gov/pubmed/?term=Obesity+in+the+Lebanese+elderly%3A+prevalence%2C+relative+risks+and+anthropometrical+measurements | | | |
| 433 | Revue des maladies respiratoires | Riachy M | 2008 | 0 | http://www.ncbi.nlm.nih.gov/pubmed/?term=Impact+of+low+socioeconomic+status+on+the+demography+and+co-morbidities+of+asthma | | | |
| 511 | Annals of Saudi medicine | Taleb N | 2008 | 0 | http://www.ncbi.nlm.nih.gov/pubmed/?term=taleb+2008+Prevalence+and+determinants+of+albuminuria+in+a+cohort+of+diabetic+patients+in+Lebanon | | | |
| 24 | J Med Liban | Abou-Mourad Y | 2008 | 0 | http://www.ncbi.nlm.nih.gov/pubmed/?term=Docetaxel+and+irinotecan+as+first-line+chemotherapy+in+patients+with+advanced+non-small-cell+lung+cancer%3A+a+pilot+study | | | |
| 27 | J Med Liban | Abu-Saad Huijer H | 2008 | 0 | http://www.ncbi.nlm.nih.gov/pubmed/19534076 | | | |
| 132 | Current diabetes reports | Chen S | 2008 | 0 | http://www.ncbi.nlm.nih.gov/pubmed/18990304 | | | |
| 383 | J Med Liban | Naja Z | 2008 | 0 | http://www.ncbi.nlm.nih.gov/pubmed/?term=Management+of+cancer+pain%3A+different+intervention+techniques+naja | | | |
| 161 | Contemporary nurse | Doumit M A | 2008 | 0 | http://www.ncbi.nlm.nih.gov/pubmed/?term=The+purpose+of+this+study+was+to+describe+and+interpret+the+phenomenon+of+communication+as+lived+by+Lebanese+cancer+patients.+Phenomenology+based+on+the+Utrecht+School+was+chosen+as+an+interpretive+descriptive+meth | | | |
| 165 | Cancer nursing | Doumit M A | 2008 | 0 | http://www.ncbi.nlm.nih.gov/pubmed/?term=The+lived+experience+of+Lebanese+family+caregivers+of+cancer+patients | | | |
| 8 | Journal de radiologie | Abassi R | 2008 | 1 | http://www.ncbi.nlm.nih.gov/pubmed/18477960 | | | |
| 753 | Minerva Stomatologica | Otmani N | 2008 |  | http://www.ncbi.nlm.nih.gov/pubmed/18427382 | | | |
| 895 | Annales de Pathologie | Jabri L | 2008 |  | http://www.ncbi.nlm.nih.gov/pubmed/18984285 | | | |
| 900 | Annales de Dermatologie et de Vénéréologie | Jamali M | 2008 |  | http://www.ncbi.nlm.nih.gov/pubmed/18598813 | | | |
| 903 | Annales de Dermatologie et de Vénéréologie | Tadlaoui I | 2008 |  | http://www.ncbi.nlm.nih.gov/pubmed/18374867 | | | |
| 17 | Annales d'endocrinologie | Aderdour L | 2008 | 0 | http://www.ncbi.nlm.nih.gov/pubmed/18930451 | | | |
| 89 | J Fr Ophtalmol | Benhaddou R | 2008 | 1 | http://www.ncbi.nlm.nih.gov/pubmed/18563045 | | | |
| 95 | Cases Journal | Benjelloun E B | 2008 | 0 | http://www.ncbi.nlm.nih.gov/pubmed/19077286 | | | |
| 147 | Acta Orthopaedica Belgica | Boussouga M | 2008 | 0 | http://www.ncbi.nlm.nih.gov/pubmed/18411611 | | | |
| 236 | Journal of cancer research and Therapeutics | Elharroudi T | 2008 | 0 | http://www.ncbi.nlm.nih.gov/pubmed/19052394 | | | |
| 316 | Cases journal | Ismaili N | 2008 | 0 | http://www.ncbi.nlm.nih.gov/pubmed/19040740 | | | |
| 354 | American Journal of Neuroradiology | Lakhdar F | 2008 | 1 | http://www.ncbi.nlm.nih.gov/pubmed/17572493 | | | |
| 366 | Joint Bone Spine | Lmejjati M | 2008 | 0 | http://www.ncbi.nlm.nih.gov/pubmed/18487067 | | | |
| 370 | Clin Exp Rheumatol | Maamar M | 2008 | 0 | http://www.ncbi.nlm.nih.gov/pubmed/18799103 | | | |
| 384 | Acta Otolaryngol | Mansouri H | 2008 | 0 | http://www.ncbi.nlm.nih.gov/pubmed/18274920 | | | |
| 430 | Joint Bone Spine | Okacha N | 2008 | 0 | http://www.ncbi.nlm.nih.gov/pubmed/17905632 | | | |
| 509 | Singapore Medical Journal | Tazzi I | 2008 | 0 | http://www.ncbi.nlm.nih.gov/pubmed/18756337 | | | |
| 521 | Archives of Cardiovascular Diseases | Tribak M | 2008 | 0 | http://www.ncbi.nlm.nih.gov/pubmed/18477949 | | | |
| 739 | Singapore Medical Journal | Tazi I | 2008 | 0 | http://www.ncbi.nlm.nih.gov/pubmed/18756337 | | | |
| 897 | Revue de stomatologie et de chirurgie maxillo-faciale | Moumine M | 2008 | 1 | http://www.ncbi.nlm.nih.gov/pubmed/18951596 | | | |
| 898 | Revue neurologique | Bourezgui M | 2008 | 1 | http://www.ncbi.nlm.nih.gov/pubmed/18808776 | | | |
| 899 | La Revue du praticien | El Harroudi T | 2008 | 1 | http://www.ncbi.nlm.nih.gov/pubmed/18689111 | | | |
| 901 | Revue neurologique | Moudden M | 2008 | 1 | http://www.ncbi.nlm.nih.gov/pubmed/18565363 | | | |
| 902 | Bulletin du Cancer | Benazzouz B | 2008 | 1 | http://www.ncbi.nlm.nih.gov/pubmed/18495576 | | | |
| 904 | Cancer radiothérapie | Khouchani M | 2008 | 1 | http://www.ncbi.nlm.nih.gov/pubmed/18343704 | | | |
| 906 | La Revue de médecine interne | Ennibi K | 2008 | 1 | http://www.ncbi.nlm.nih.gov/pubmed/18221824 | | | |
| 907 | Presse médicale | Rafai MA | 2008 | 1 | http://www.ncbi.nlm.nih.gov/pubmed/18201863 | | | |
| 45 | Revue des maladies respiratoires | Amara B | 2008 | 1 | http://www.ncbi.nlm.nih.gov/pubmed/18535524 | | | |
| 150 | Odontostomatologie Tropicale | Bouziane A | 2008 | 1 | http://www.ncbi.nlm.nih.gov/pubmed/19266844 | | | |
| 173 | J Clin Virol | Dardari R | 2008 | 0 | http://www.ncbi.nlm.nih.gov/pubmed/18024156 | | | |
| 228 | The International Journal of Tuberculosis and Lung Disease | El Rhazi K | 2008 | 0 | http://www.ncbi.nlm.nih.gov/pubmed/18926045 | | | |
| 243 | Psycho-oncology | Errihani H | 2008 | 0 | http://www.ncbi.nlm.nih.gov/pubmed/17458922 | | | |
| 249 | Mutation research | Ezzikouri S | 2008 | 0 | http://www.ncbi.nlm.nih.gov/pubmed/18023606 | | | |
| 250 | Archives of Medical Research | Ezzikouri S | 2008 | 0 | http://www.ncbi.nlm.nih.gov/pubmed/18164971 | | | |
| 399 | Clin Exp Hypertens | M'Guil M | 2008 | 0 | http://www.ncbi.nlm.nih.gov/pubmed/18633757 | | | |
| 401 | American Journal of Human Biology | Mohammed E A | 2008 | 0 | http://www.ncbi.nlm.nih.gov/pubmed/18293371 | | | |
| 434 | International Journal of Oral and Maxillofacial Surgery | Otmani N | 2008 | 0 | http://www.ncbi.nlm.nih.gov/pubmed/17822883 | | | |
| 450 | Eur Urol | Ravery V | 2008 | 0 | http://www.ncbi.nlm.nih.gov/pubmed/17467885 | | | |
| 481 | Thérapie | Soussi-Tanani D | 2008 | 1 | http://www.ncbi.nlm.nih.gov/pubmed/18561887 | | | |
| 504 | Pediatr Blood Cancer | Tazzi I | 2008 | 0 | http://www.ncbi.nlm.nih.gov/pubmed/18636463 | | | |
| 740 | Pediatr Blood Cancer | Tazi I | 2008 | 0 | http://www.ncbi.nlm.nih.gov/pubmed/18636463 | | | |
| 752 | Minerva Stomatologica | Otmani N | 2008 | 0 | http://www.ncbi.nlm.nih.gov/pubmed/19078892 | | | |
| 886 | Tunis Med | Ennibi K | 2008 | 1 | http://www.ncbi.nlm.nih.gov/pubmed/19469300 | | | |
| 892 | Eastern Mediterranean Health Journal | El AY | 2008 | 1 | http://www.ncbi.nlm.nih.gov/pubmed/19161081 | | | |
| 905 | Saudi Journal of Kidney Disease and Transplantation | Sqalli TH | 2008 | 0 | http://www.ncbi.nlm.nih.gov/pubmed/18310880 | | | |
| 85 | Bone Marrow Transplant | Benchekroun S | 2008 | 0 | http://www.ncbi.nlm.nih.gov/pubmed/18724279 | | | |
| 208 | Blood cells, molecules & diseases | El hauoari M | 2008 | 0 | http://www.ncbi.nlm.nih.gov/pubmed/18387322 | | | |
| 20 | Journal of ethnopharmacology | Afkir S | 2008 | 0 | http://www.ncbi.nlm.nih.gov/pubmed/18191352 | | | |
| 62 | Sociol Health Illn | Batnitzky A | 2008 | 0 | http://www.ncbi.nlm.nih.gov/pubmed/18373507 | | | |
| 261 | Bulletin of the world health organization | Gavlak D | 2008 | 0 | http://www.ncbi.nlm.nih.gov/pubmed/18297160 | | | |
| 526 | Methods and Findings in experimental and clinical pharmacology | Zeggwagh NA | 2008 | 0 | http://www.ncbi.nlm.nih.gov/pubmed/18806897 | | | |
| 738 | Journal of Clinical Oncology | Tazi I | 2008 | 0 | http://www.ncbi.nlm.nih.gov/pubmed/18936464 | | | |
| 280 | Pediatr Blood Cancer | Harif M | 2008 | 0 | http://www.ncbi.nlm.nih.gov/pubmed/18213709 | | | |
| 349 | Obesity Reviews | Lahmam A | 2008 | 0 | http://www.ncbi.nlm.nih.gov/pubmed/17931349 | | | |
| 380 | Acta bio-medica | Majaliwa E S | 2008 | 0 | http://www.ncbi.nlm.nih.gov/pubmed/19260389 | | | |
| 458 | The Lancet Oncology | Ribeiro RC | 2008 | 0 | http://www.ncbi.nlm.nih.gov/pubmed/18672210 | | | |
| 139 | Journal of Craniofacial Surgery | Boulaadas M | 2008 | 0 | http://www.ncbi.nlm.nih.gov/pubmed/18650758 | | | |
| 126 | J Thorac Oncol | Berthiller J | 2008 | 0 | http://www.ncbi.nlm.nih.gov/pubmed/19057263 | | | |
| 382 | Revue des maladies respiratoires | Malih M | 2008 | 1 | http://www.ncbi.nlm.nih.gov/pubmed/18946404 | | | |
| 195 | Diabetes Research and Clinical Practice | ElAchhab Y | 2008 | 0 | http://www.ncbi.nlm.nih.gov/pubmed/18279993 | | | |
| 190 | Phytotherapy Research | Eddouks M | 2008 | 0 | http://www.ncbi.nlm.nih.gov/pubmed/18064603 | | | |
| 145 | ONS connect | Saca-Hazboun H | 2008 | 0 | http://www.ncbi.nlm.nih.gov.ezproxy.aub.edu.lb/pubmed/18807706 | | | |
| 146 | ONS connect | Saca-Hazboun H | 2008 | 0 | http://www.ncbi.nlm.nih.gov.ezproxy.aub.edu.lb/pubmed/18767567 | | | |
| 147 | ONS connect | Saca-Hazboun H | 2008 | 0 | http://www.ncbi.nlm.nih.gov.ezproxy.aub.edu.lb/pubmed/18330335 | | | |
| 45 | Int J Clin Pharm TH | Sweileh WM | 2008 | 0 | http://www.ncbi.nlm.nih.gov/pubmed/19049697 | | | |
| 46 | J Stroke Cerebrovasc Dis | Sweileh WM | 2008 | 0 | http://www.ncbi.nlm.nih.gov/pubmed/18984436 | | | |
| 152 | Medical principles and practice | Sweileh WM | 2008 | 0 | http://www.ncbi.nlm.nih.gov.ezproxy.aub.edu.lb/pubmed/18287792 | | | |
| 153 | Journal of clinical laboratory analysis | Tarazi IS | 2008 | 0 | http://www.ncbi.nlm.nih.gov.ezproxy.aub.edu.lb/pubmed/18348310 | | | |
| 140 | Clinical Medicine: Oncology | Nehari M | 2008 | 0 | http://www.ncbi.nlm.nih.gov.ezproxy.aub.edu.lb/pmc/articles/PMC3161677/ | | | |
| 48 | J Epidemiol Community Health | Abu-Mourad T | 2008 | 0 | http://www.ncbi.nlm.nih.gov/pubmed/18621955 | | | |
| 80 | Preventing chronic disease | Abu-Rmeileh NM | 2008 | 0 | http://www.ncbi.nlm.nih.gov.ezproxy.aub.edu.lb/pubmed/18793500 | | | |
| 10 | Tropical doctor | Abuidris D O | 2008 | 0 | https://www.ncbi.nlm.nih.gov.ezproxy.aub.edu.lb/pubmed/?term=Childhood+cancer+in+Sudan%3A+1999-2007 | | | |
| 11 | Saudi medical journal | Abuidris D O | 2008 | 0 | https://www.ncbi.nlm.nih.gov.ezproxy.aub.edu.lb/pubmed/?term=Histopathological+patterns+of+nasopharyngeal+carcinoma+in+Sudan | | | |
| 12 | Pediatric blood & cancer | Abuidris D O | 2008 | 0 | https://www.ncbi.nlm.nih.gov.ezproxy.aub.edu.lb/pubmed/18384057 | | | |
| 45 | Saudi medical journal | Bohlega S A | 2008 | 0 | http://www.ncbi.nlm.nih.gov/pubmed/18626519 | | | |
| 52 | Saudi medical journal | Elagib A H | 2008 | 0 | http://www.ncbi.nlm.nih.gov/pubmed/?term=Possible+predisposing+factors+for+thrombotic+cerebrovascular+accidents+in+Sudanese+patients | | | |
| 60 | Nuclear medicine communications | Elmadani A E | 2008 | 0 | http://www.ncbi.nlm.nih.gov/pubmed/?term=The+contribution+of+bone+SPECT+to+the+diagnosis+of+bone+metastases+in+an+African+population | | | |
| 63 | Eastern Mediterranean health journal | Elnasri H A | 2008 | 0 | https://www.ncbi.nlm.nih.gov.ezproxy.aub.edu.lb/pubmed/?term=Patterns+of+lipid+changes+among+type+2+diabetes+patients+in+Sudan | | | |
| 89 | Journal of wound care | Mahmoud S M | 2008 | 0 | https://www.ncbi.nlm.nih.gov.ezproxy.aub.edu.lb/pubmed/18705232 | | | |
| 95 | Education for health | Moukhyer M E | 2008 | 0 | http://www.ncbi.nlm.nih.gov/pubmed/?term=Health-related+behaviors+of+Sudanese+adolescents | | | |
| 113 | Annals of the New York Academy of Sciences | Siddig A | 2008 | 0 | http://www.ncbi.nlm.nih.gov/pubmed/18837889 | | | |
| 114 | Annals of the New York Academy of Sciences | Siddig A | 2008 | 0 | http://www.ncbi.nlm.nih.gov/pubmed/18837888 | | | |
| 44 | The lancet oncology | Boffetta P | 2008 | 0 | http://www.ncbi.nlm.nih.gov/pubmed/18598931 | | | |
| 50 | Hematology/oncology and stem cell therapy | El Hassan A | 2008 | 0 | https://www.ncbi.nlm.nih.gov.ezproxy.aub.edu.lb/pubmed/?term=Malignant+gastric+tumors+in+Sudan%3A+a+report+from+a+single+pathology+center | | | |
| 38 | Histopathology | Awadelkarim K D | 2008 | 0 | http://www.ncbi.nlm.nih.gov/pubmed/?term=Pathological%2C+clinical+and+prognostic+characteristics+of+breast+cancer+in+Central+Sudan+versus+Northern+Italy%3A+implications+for+breast+cancer+in+Africa | | | |
| 107 | Oncology reports | Roman E | 2008 | 0 | http://www.ncbi.nlm.nih.gov/pubmed/?term=Chromosomal+aberrations+in+head+and+neck+squamous+cell+carcinomas+in+Norwegian+and+Sudanese+populations+by+array+comparative+genomic+hybridization | | | |
| 90 | Acta bio-medica | Majaliwa E S | 2008 | 0 | http://www.ncbi.nlm.nih.gov/pubmed/?term=Type+1+diabetes+mellitus+in+the+African+population%3A+epidemiology+and+management+challenges | | | |
| 119 | British Medical Journal | Turone F | 2008 | 0 | http://www.ncbi.nlm.nih.gov/pubmed/?term=Outcomes+from+Khartoum+heart+hospital+match+the+best+Western+centres | | | |
| 184 | Pediatric Dermatology | Louri N | 2009 | 0 | http://www.ncbi.nlm.nih.gov/pubmed/20199469 | | | |
| 104 | Eastern Mediterranean health journal | Fadhil I | 2009 | 0 | http://www.ncbi.nlm.nih.gov/pubmed/20187549 | | | |
| 112 | Journal of Stroke and Cerebrovascular Diseases | Almawi WY | 2009 | 0 | http://www.ncbi.nlm.nih.gov/pubmed/19717029 | | | |
| 119 | Public Health Nutrition | Al-Ghawi A | 2009 | 0 | http://www.ncbi.nlm.nih.gov/pubmed/?term=Study+of+the+knowledge%2C+attitudes+and+practices+of+physicians+towards+obesity+management+in+primary+health+care+in+Bahrain. | | | |
| 120 | Clinical and Vaccine Immunology | Stayoussef M | 2009 | 0 | http://www.ncbi.nlm.nih.gov/pubmed/19005023 | | | |
| 199 | Journal of Immigrant and Minority Health | Musaiger AO | 2009 | 0 | http://www.ncbi.nlm.nih.gov/pubmed/18607727 | | | |
| 109 | Eastern Mediterranean Health Journal | Ravichandran K | 2009 | 0 | http://www.ncbi.nlm.nih.gov/pubmed/?term=Association+of+reproductive+factors+with+the+incidence+of+breast+cancer+in+Gulf+Cooperation+Council+countries | | | |
| 116 | Journal of Neurological Sciences | Benamer HT | 2009 | 0 | http://www.ncbi.nlm.nih.gov/pubmed/19428027 | | | |
| 114 | Journal of Oleo Science | Freije A | 2009 | 0 | http://www.ncbi.nlm.nih.gov/pubmed/?term=Fatty+acid+profile+of+the+erythrocyte+membranes+of+healthy+Bahraini+citizens+in+comparison+with+coronary+heart+disease+patients. | | | |
| 106 | Journal of endocrinological investigation | Al-Salman RA | 2009 | 1 | http://www.ncbi.nlm.nih.gov/pubmed/?term=Prevalence+and+risk+factors+of+albuminuria+in+Type+2+diabetes+in+Bahrain. | | | |
| 107 | International Journal of Pediatric Obesity | Al-Raees GY | 2009 | 0 | http://www.ncbi.nlm.nih.gov/pubmed/?term=Prevalence+of+overweight+and+obesity+among+children+aged+2-5+years+in+Bahrain%3A+a+comparison+between+two+reference+standards. | | | |
| 108 | American Journal of Cardiology | El-Menyar A | 2009 | 0 | http://www.ncbi.nlm.nih.gov/pubmed/?term=Comparison+of+men+and+women+with+acute+coronary+syndrome+in+six+Middle+Eastern+++++++countries | | | |
| 113 | Annals of Saudi Medicine | Gharib NM | 2009 | 0 | http://www.ncbi.nlm.nih.gov/pubmed/19584585 | | | |
| 115 | Diagnostic Cytopathology | Al-Sindi K | 2009 | 0 | http://www.ncbi.nlm.nih.gov/pubmed/?term=Efficacy+of+fine-needle+aspiration+biopsy+in+diagnosis+of+breast+cancer%3A+a+retrospective+study+of+303+cases+in+Bahrain. | | | |
| 122 | Angiology | El-Menyar A | 2009 | 0 | http://www.ncbi.nlm.nih.gov/pubmed/?term=Ankle-brachial+index+and+extent+of+atherosclerosis+in+patients+from+the+Middle+East+(the+AGATHA-ME+study)%3A+a+cross-sectional+multicenter+study | | | |
| 110 | Eastern Mediterranean Health Journal | Al-Hamdan N | 2009 | 0 | http://www.ncbi.nlm.nih.gov/pubmed/19731776 | | | |
| 111 | Acta Cardiologica | Zubaid M | 2009 | 0 | http://www.ncbi.nlm.nih.gov/pubmed/19725435 | | | |
| 69 | Eastern Mediterranean Health Journal | Mossawe JF | 2009 | 0 | http://www.ncbi.nlm.nih.gov/pubmed/20214136 | | | |
| 71 | International Journal of Diabetes in Developing Countries | Al-Nimer MS | 2009 | 0 | http://www.ncbi.nlm.nih.gov/pubmed/20062559 | | | |
| 74 | Eating and Weight Disorders | Mansour AA | 2009 | 0 | http://www.ncbi.nlm.nih.gov/pubmed/19934637 | | | |
| 75 | Neurology India | Al-Allawi NA | 2009 | 0 | http://www.ncbi.nlm.nih.gov/pubmed/19934565 | | | |
| 76 | Saudi Medical Journal | Hasan NA | 2009 | 0 | http://www.ncbi.nlm.nih.gov/pubmed/19838431 | | | |
| 78 | Allergology International | Alsamarai AM | 2009 | 0 | http://www.ncbi.nlm.nih.gov/pubmed/19700932 | | | |
| 79 | World Allergy Organization Journal | Al Obaidi AH | 2009 | 0 | http://www.ncbi.nlm.nih.gov/pubmed/23283064 | | | |
| 80 | Journal Laboratory Physicians | Al-Hakeim HK | 2009 | 0 | http://www.ncbi.nlm.nih.gov/pubmed/21938249 | | | |
| 85 | Saudi Medical Journal | Al-Shamiri SA | 2009 | 0 | http://www.ncbi.nlm.nih.gov/pubmed/19198717 | | | |
| 86 | Journal of Craniofacial Surgery | Kummoona R | 2009 | 0 | http://www.ncbi.nlm.nih.gov/pubmed/19165012 | | | |
| 88 | Neurosciences | Hussein II | 2009 | 0 | http://www.ncbi.nlm.nih.gov/pubmed/21048570 | | | |
| 202 | Saudi Journal of Kidney Disease and Transplantation | Al-Bazzaz PH | 2009 | 0 | http://www.ncbi.nlm.nih.gov/pubmed/19587505 | | | |
| 268 | Saudi Medical Journal | Hussain SA | 2009 | 0 | http://www.ncbi.nlm.nih.gov/pubmed/19139781 | | | |
| 82 | Asian Pacific Journal of Cancer Prevention | Salim EI | 2009 | 0 | http://www.ncbi.nlm.nih.gov/pubmed/19469617 | | | |
| 83 | Journal of the Neuroligical Sciences | Benamer HT | 2009 | 0 | http://www.ncbi.nlm.nih.gov/pubmed/19428027 | | | |
| 77 | Saudi Journal of Kidney Disease and Transplantation | Hashim Al-Saedi AJ | 2009 | 0 | http://www.ncbi.nlm.nih.gov/pubmed/19736492 | | | |
| 87 | Applied immunohistochemistry & molecular morphology | Al-Abbasi DS | 2009 | 0 | http://www.ncbi.nlm.nih.gov/pubmed/19151604 | | | |
| 89 | Oncology reports | Mumtaz M | 2009 | 0 | http://www.ncbi.nlm.nih.gov/pubmed/19082456 | | | |
| 73 | BMC Women's Health | Majid RA | 2009 | 0 | http://www.ncbi.nlm.nih.gov/pubmed/20003359 | | | |
| 81 | Journal of Stroke and Cerebrovascular Disease | Al-Rawi MA | 2009 | 0 | http://www.ncbi.nlm.nih.gov/pubmed/19560679 | | | |
| 84 | MMWR Morbidity and Mortality Weekly Report | Centers for Disease Control and Prevention (CDC) | 2009 | 0 | http://www.ncbi.nlm.nih.gov/pubmed/19343010 | | | |
| 126 | Diabetes and Metabolism | Al-Hajeri T | 2009 | 0 | http://www.ncbi.nlm.nih.gov/pubmed/?term=Profound+weight+loss+in+a+type+2+diabetic+patient+with+diabetic+neuropathic+cachexia%3A+a+case+report | | | |
| 203 | Medical Principles and Practice | Rifat Mannan AA | 2009 | 0 | http://www.ncbi.nlm.nih.gov/pubmed/?term=An+unusual+case+of+extensive+xanthogranulomatous+orchitis+in+a+diabetic+patient | | | |
| 341 | The Gulf Journal of Oncology | Abdel Motaal MM | 2009 | 0 | http://www.ncbi.nlm.nih.gov/pubmed/20084786 | | | |
| 370 | The Gulf Journal of Oncology | Al Bahar S | 2009 | 0 | http://www.ncbi.nlm.nih.gov/pubmed/20084788 | | | |
| 455 | The Breast Journal | Al-Said Ali A | 2009 | 0 | http://www.ncbi.nlm.nih.gov/pubmed/19120376 | | | |
| 580 | Orthopedics | Gondusky JS | 2009 | 0 | http://www.ncbi.nlm.nih.gov.ezproxy.aub.edu.lb/pubmed/?term=Salmonella+osteomyelitis+in+new-onset+diabetes+mellitus. | | | |
| 616 | Diagnostic Cytopathology | Kapila K | 2009 | 0 | http://www.ncbi.nlm.nih.gov.ezproxy.aub.edu.lb/pubmed/?term=Mucoepidermoid+thymic+carcinoma%3A+a+challenging+mediastinal+aspirate. | | | |
| 649 | Familial Cancer | Marafie MJ | 2009 | 0 | http://www.ncbi.nlm.nih.gov.ezproxy.aub.edu.lb/pubmed/19669601 | | | |
| 727 | Annals of Saudi Medicine | Singh NG | 2009 | 0 | http://www.ncbi.nlm.nih.gov.ezproxy.aub.edu.lb/pubmed/19847088 | | | |
| 756 | Pediatr Blood Cancer | Zamecnikova A | 2009 | 0 | http://www.ncbi.nlm.nih.gov/pubmed/19142993 | | | |
| 17 | Indian Journal of Nephrology | Al-Hilali N | 2009 | 0 | http://www.ncbi.nlm.nih.gov/pubmed/20535251 | | | |
| 27 | Medical Principles and Practice | Al-Bustan SA | 2009 | 0 | http://www.ncbi.nlm.nih.gov/pubmed/?term=Apolipoprotein+E%2C+CI+and+B+gene+polymorphisms+in+a+sample+of+patients+with+++++++coronary+heart+disease+in+the+Kuwaiti+population. | | | |
| 32 | Medical Principles and Practice | Longenecker JC | 2009 | 0 | http://www.ncbi.nlm.nih.gov.ezproxy.aub.edu.lb/pubmed/?term=Association+of+low+heart+rate+variability+with+atherosclerotic+cardiovascular+++++++disease+in+hemodialysis+patients | | | |
| 163 | European Journal of Gastroenterology and Hepatology | Chehadeh W | 2009 | 0 | http://www.ncbi.nlm.nih.gov.ezproxy.aub.edu.lb/pubmed/18717762 | | | |
| 170 | Saudi Journal of Kidney Diseases and Transplantation | Ghani AA | 2009 | 0 | http://www.ncbi.nlm.nih.gov/pubmed/19237811 | | | |
| 186 | Medical Principles and Practice | Mojiminiyi OA | 2009 | 0 | http://www.ncbi.nlm.nih.gov.ezproxy.aub.edu.lb/pubmed/?term=Which+obesity+index+best+explains+the+link+between+adipokines%2C+coronary+heart+disease+risk+and+metabolic+abnormalities+in+type+2+diabetes+mellitus%3F | | | |
| 188 | Medical Principles and Practice | Mousa A | 2009 | 0 | http://www.ncbi.nlm.nih.gov.ezproxy.aub.edu.lb/pubmed/?term=Induction+of+interleukin-18+in+atherosclerotic+patients%3A+a+role+for+Chlamydia+pneumoniae | | | |
| 379 | Medical Principles and Practice | Al Sairafi M | 2009 | 0 | http://www.ncbi.nlm.nih.gov/pubmed/19060489 | | | |
| 388 | Diabetes and Metabolism | Al-Adsani A | 2009 | 0 | http://www.ncbi.nlm.nih.gov/pubmed/19250850 | | | |
| 459 | General Thoracic and Cardiovascular Surgery | Al-Sarraf N | 2009 | 0 | http://www.ncbi.nlm.nih.gov/pubmed/19214449 | | | |
| 472 | BMC Cancer | Alawadi S | 2009 | 0 | http://www.ncbi.nlm.nih.gov/pubmed/19586536 | | | |
| 561 | Journal of Womens Health | El-Hammasi K | 2009 | 0 | http://www.ncbi.nlm.nih.gov/pubmed/19951218 | | | |
| 628 | The international journal of tuberculosis and lung disease | Khadadah M | 2009 | 0 | http://www.ncbi.nlm.nih.gov.ezproxy.aub.edu.lb/pubmed/19723383 | | | |
| 652 | Japanese Journal of Clinical Oncology | Marugame T | 2009 | 0 | http://www.ncbi.nlm.nih.gov.ezproxy.aub.edu.lb/pubmed/19155285 | | | |
| 867 | European Journal of Clinical Nutrition | Al-Qaoud N | 2009 | 0 | http://www.ncbi.nlm.nih.gov/pubmed/19337291 | | | |
| 721 | Medical Principles and Practice | Sheikh M | 2009 | 0 | http://www.ncbi.nlm.nih.gov.ezproxy.aub.edu.lb/pubmed/19494542 | | | |
| 29 | Journal of Neurological Sciences | Benamer HT | 2009 | 0 | http://www.ncbi.nlm.nih.gov/pubmed/19428027 | | | |
| 178 | Journal of Gastroenterology and Hepatology | Mohamed MK | 2009 | 0 | http://www.ncbi.nlm.nih.gov/pubmed/19196393 | | | |
| 378 | Cathaterization and Cardiovascular interventions | Al Rashdan I | 2009 | 0 | http://www.ncbi.nlm.nih.gov/pubmed/19496132 | | | |
| 538 | Diagnostic Cytopathology | Das DK | 2009 | 0 | http://www.ncbi.nlm.nih.gov/pubmed/19373908 | | | |
| 569 | The Gulf Journal of Oncology | Elqazzar AH | 2009 | 0 | http://www.ncbi.nlm.nih.gov.ezproxy.aub.edu.lb/pubmed/?term=Metastatic+bone+disease%3A+evaluation+by+functional+imaging+in+correlation+with+morphologic+modalities. | | | |
| 702 | Eastern Mediterranean Health Journal | Ravichandran K | 2009 | 0 | http://www.ncbi.nlm.nih.gov/pubmed/?term=Association+of+reproductive+factors+with+the+incidence+of+breast+cancer+in+Gulf+Cooperation+Council+countries | | | |
| 745 | The Gulf Journal of Oncology | Varghese A | 2009 | 0 | http://www.ncbi.nlm.nih.gov.ezproxy.aub.edu.lb/pubmed/20194087 | | | |
| 889 | Neoplasma | Novotny L | 2009 | 0 | http://www.ncbi.nlm.nih.gov/pubmed/19309219 | | | |
| 23 | Redox Report | Khan I | 2009 | 0 | http://www.ncbi.nlm.nih.gov/pubmed/?term=Effect+of+potent+redox-modulating+manganese+porphyrin%2C+MnTM-2-PyP%2C+on+the+++++++Na(%2B)%2FH(%2B)+exchangers+NHE-1+and+NHE-3+in+the+diabetic+rat | | | |
| 28 | Current Hypertension Reports | Sharma JN | 2009 | 0 | http://www.ncbi.nlm.nih.gov/pubmed/19442326 | | | |
| 30 | Vascular Pharmacology | Benter IF | 2009 | 0 | http://www.ncbi.nlm.nih.gov/pubmed/19410658 | | | |
| 33 | Journal of Toxicological Sciences | Al-Bloushi S | 2009 | 0 | http://www.ncbi.nlm.nih.gov.ezproxy.aub.edu.lb/pubmed/?term=Green+tea+modulates+reserpine+toxicity+in+animal+model | | | |
| 133 | Journal of Nutrition | Al-Khalifa A | 2009 | 0 | http://www.ncbi.nlm.nih.gov/pubmed/19818281 | | | |
| 136 | Pharmacological research | Al-Maghrebi M | 2009 | 0 | http://www.ncbi.nlm.nih.gov/pubmed/?term=Endogenous+angiotensin-(1-7)+reduces+cardiac+ischemia-induced+dysfunction+in+diabetic+hypertensive+rats | | | |
| 154 | Vascular Pharmacology | Benter IF | 2009 | 0 | http://www.ncbi.nlm.nih.gov/pubmed/19577003 | | | |
| 223 | Autonomic and Autacoid Pharmacology | Yousif MH | 2009 | 0 | http://www.ncbi.nlm.nih.gov/pubmed/?term=Role+of+20-hydroxyeicosatetraenoic+acid+in+altering+vascular+reactivity+in+diabetes | | | |
| 225 | Autonomic and Autacoid Pharmacology | Yousif MH | 2009 | 0 | http://www.ncbi.nlm.nih.gov/pubmed/?term=Cytochrome+P450+metabolites+of+arachidonic+acid+play+a+role+in+the+enhanced+cardiac+dysfunction+in+diabetic+rats+following+ischaemic+reperfusion+injury | | | |
| 390 | International Journal of Food Sciences and Nutrition | Al-Amiri HA | 2009 | 0 | http://www.ncbi.nlm.nih.gov/pubmed/19919514 | | | |
| 429 | Annals of Saudi Medicine | Al-Khawari H | 2009 | 0 | http://www.ncbi.nlm.nih.gov/pubmed/19584584 | | | |
| 535 | Acta Cytologica | Das DK | 2009 | 0 | http://www.ncbi.nlm.nih.gov/pubmed/19798876 | | | |
| 536 | Diagnostic Cytopathology | Das DK | 2009 | 0 | http://www.ncbi.nlm.nih.gov/pubmed/19373880 | | | |
| 552 | International Journal of Technological Assessment in Health Care | Doi SA | 2009 | 0 | http://www.ncbi.nlm.nih.gov/pubmed/19126258 | | | |
| 567 | Saudi Medical Journal | El-Sabban FM | 2009 | 0 | http://www.ncbi.nlm.nih.gov/pubmed/19618019 | | | |
| 641 | International Journal of Oncology | Luqmani YA | 2009 | 0 | http://www.ncbi.nlm.nih.gov.ezproxy.aub.edu.lb/pubmed/?term=Modification+of+gene+expression+induced+by+siRNA+targeting+of+estrogen+receptor+alpha+in+MCF7+human+breast+cancer+cells. | | | |
| 886 | International Immunopharmacology | Ezeamuzie CI | 2009 | 0 | http://www.ncbi.nlm.nih.gov/pubmed/19505590 | | | |
| 413 | The Gulf Journal of Oncology | Al-Hendal A | 2009 | 0 | http://www.ncbi.nlm.nih.gov/pubmed/20084789 | | | |
| 537 | Acta Cytologica | Das DK | 2009 | 0 | http://www.ncbi.nlm.nih.gov/pubmed/19798886 | | | |
| 900 | Acta Cardiologica | Zubaid M | 2009 | 0 | http://www.ncbi.nlm.nih.gov/pubmed/19725435 | | | |
| 24 | Annals of Saudi Medicine | Ghani AA | 2009 | 0 | http://www.ncbi.nlm.nih.gov/pubmed/?term=Renal+biopsy+in+patients+with+type+2+diabetes+mellitus%3A+indications+and+nature+of+++++++the+lesions | | | |
| 25 | American Journal of Cardiology | El-Menyar A | 2009 | 0 | http://www.ncbi.nlm.nih.gov/pubmed/?term=Comparison+of+men+and+women+with+acute+coronary+syndrome+in+six+Middle+Eastern+++++++countries | | | |
| 168 | Angiology | El-Menyar A | 2009 | 0 | http://www.ncbi.nlm.nih.gov/pubmed/?term=Ankle-brachial+index+and+extent+of+atherosclerosis+in+patients+from+the+Middle+East+(the+AGATHA-ME+study)%3A+a+cross-sectional+multicenter+study | | | |
| 448 | European Journal of Clinical Nutrition | Al-Qaoud N | 2009 | 0 | http://www.ncbi.nlm.nih.gov/pubmed/19337291 | | | |
| 449 | Medical Principles and Practice | Al-Qaoud N | 2009 | 0 | http://www.ncbi.nlm.nih.gov/pubmed/19204429 | | | |
| 488 | Cardiovasc Ther | Alsayegh F | 2009 | 0 | http://www.ncbi.nlm.nih.gov/pubmed/19426243 | | | |
| 546 | Saudi Medical Journal | Dehghan M | 2009 | 0 | http://www.ncbi.nlm.nih.gov/pubmed/19139794 | | | |
| 558 | Asia-Pacific Journal of Public Health | El-Bayoumy I | 2009 | 0 | http://www.ncbi.nlm.nih.gov/pubmed/19190003 | | | |
| 638 | Medical Principles and Practice | Lamloum SM | 2009 | 0 | http://www.ncbi.nlm.nih.gov.ezproxy.aub.edu.lb/pubmed/?term=Relationship+between+postoperative+infectious+complications+and+glycemic+control+for+diabetic+patients+in+an+orthopedic+hospital+in+Kuwait. | | | |
| 403 | Cases Journal | Al-Fadhli J | 2009 | 0 | http://www.ncbi.nlm.nih.gov/pubmed/19918456 | | | |
| 26 | Metabolic Syndrome and Related Disorders | Akanji AO | 2009 | 0 | http://www.ncbi.nlm.nih.gov/pubmed/19642911 | | | |
| 409 | Eastern Mediterranean Health Journal | Al-Hamdan N | 2009 | 0 | http://www.ncbi.nlm.nih.gov/pubmed/19731776 | | | |
| 431 | Medical Principles and Practice | Al-Khawari H | 2009 | 0 | http://www.ncbi.nlm.nih.gov/pubmed/19204434 | | | |
| 34 | American Journal of Cardiovascular Drugs | Al Khalaf MM | 2009 | 0 | http://www.ncbi.nlm.nih.gov/pubmed/19178130 | | | |
| 430 | Hematology/Oncology and Stem Cell Therapy | Al-Khawari H | 2009 | 0 | http://www.ncbi.nlm.nih.gov/pubmed/20139054 | | | |
| 437 | Journal of Clinical Pathology | Al-Mulla F | 2009 | 0 | http://www.ncbi.nlm.nih.gov/pubmed/19329713 | | | |
| 65 | J Med Liban | Atallah D | 2009 |  | Not Found | |  |  |
| 96 | J Med Liban | Birjawi G | 2009 |  | Not Found | |  |  |
| 412 | Progress in cardiovascular nursing | Noureddine S | 2009 |  | Abstract Not Found | |  |  |
| 616 | J Med Liban | Ghossain A | 2009 |  | http://www.ncbi.nlm.nih.gov/pubmed/?term=Special+and+rare+images+in+breast+cancer. | | | |
| 2 | Archives of dermatology | Abbas O | 2009 | 0 | Not Found | |  |  |
| 7 | Radiation oncology | Abboud M | 2009 | 0 | http://www.ncbi.nlm.nih.gov/pubmed/19580667 | | | |
| 9 | Case reports in oncology | Abboud M | 2009 | 0 | http://www.ncbi.nlm.nih.gov/pubmed/?term=Maxillary+Sinus+Squamous+Cell+Carcinoma+Presenting+with+Fatal+Tumor+Lysis+Syndrome%3A+A+Case+Report+and+Review+of+the+Literature | | | |
| 54 | Lupus | Aoun E G | 2009 | 0 | http://www.ncbi.nlm.nih.gov/pubmed/?term=hood+stroke+in+a+child+with+familial+Mediterranean+fever+carrying+several+prothrombotic+risk+factors | | | |
| 99 | Transfusion | Bitar N | 2009 | 0 | http://www.ncbi.nlm.nih.gov/pubmed/?term=Adult+T-cell+leukemia%2Flymphoma+in+the+Middle+East%3A+first+report+of+two+cases+from+Lebanon | | | |
| 171 | Clinical and applied thrombosis/hemostasis | El Rassi F A | 2009 | 0 | http://www.ncbi.nlm.nih.gov/pubmed/?term=Myocardial+infarction+in+a+28-year-old+thalassemia+intermedia+patient | | | |
| 216 | World journal of surgical oncology | Farhat F S | 2009 | 0 | http://www.ncbi.nlm.nih.gov/pubmed/?term=Tongue+carcinoma+in+an+adult+Down's+syndrome+patient%3A+a+case+report | | | |
| 221 | Breast | Farhat M H | 2009 | 0 | http://www.ncbi.nlm.nih.gov/pubmed/?term=Modified+resistance+to+chemotherapy+and+trastuzumab+by+bevacizumab+in+locally+recurrent+breast+cancer | | | |
| 263 | The Annals of thoracic surgery | Haddad F | 2009 | 0 | http://www.ncbi.nlm.nih.gov/pubmed/19853134 | | | |
| 373 | Neuropediatrics | Muwakkit S | 2009 | 0 | http://www.ncbi.nlm.nih.gov/pubmed/?term=Wernicke's+encephalopathy+during+total+parenteral+nutrition+in+a+child+with+acute+lymphoblastic+leukemia+and+acute+pancreatitis | | | |
| 560 | J Neurosurg | Ali Y | 2009 | 0 | http://www.ncbi.nlm.nih.gov/pubmed/19361258 | | | |
| 567 | Scand J Plast Reconstr Surg Hand Surg | Atiyeh BS | 2009 | 0 | http://www.ncbi.nlm.nih.gov/pubmed/19688646 | | | |
| 615 | The American Journal of Dermatopathology | Ghosn S | 2009 | 0 | http://www.ncbi.nlm.nih.gov/pubmed/?term=Concomitant+occurrence+of+kimura+disease+and+mycosis+fungoides+in+a+Lebanese+woman%3A+significance+and+response+to+rituximab. | | | |
| 961 | Clinical cardiology | Dakik HA | 2009 | 0 | http://www.ncbi.nlm.nih.gov/pubmed/19816970 | | | |
| 30 | Palliative & supportive care | Abu-Saad Huijer H | 2009 | 0 | http://www.ncbi.nlm.nih.gov/pubmed/?term=Perspectives+on+palliative+care+in+Lebanon%3A+knowledge%2C+attitudes%2C+and+practices+of+medical+and+nursing+specialties | | | |
| 110 | Journal of clinical densitometry | Chaiban J | 2009 | 0 | http://www.ncbi.nlm.nih.gov/pubmed/?term=Modeling+pathways+for+low+bone+mass+in+children+with+malignancies | | | |
| 120 | Hematology/oncology and stem cell therapy | Charafeddine K M | 2009 | 0 | http://www.ncbi.nlm.nih.gov/pubmed/?term=Long-term+outcome+of+adult+acute+lymphoblastic+leukemia+in+Lebanon%3A+a+single+institution+experience+from+the+American+University+of+Beirut | | | |
| 124 | Metabolism | Chedid R | 2009 | 0 | http://www.ncbi.nlm.nih.gov/pubmed/?term=Impact+of+different+metabolic+syndrome+classifications+on+the+metabolic+syndrome+prevalence+in+a+young+Middle+Eastern+population | | | |
| 133 | Saudi journal of kidney diseases and transplantation | Chmaisse H N | 2009 | 0 | http://www.ncbi.nlm.nih.gov/pubmed/?term=A+study+on+the+association+between+angiotensin-I+converting+enzyme+I%2FD+dimorphism+and+type-2+diabetes+mellitus | | | |
| 145 | Journal of nuclear cardiology | Dakik H A | 2009 | 0 | http://www.ncbi.nlm.nih.gov/pubmed/?term=International+variations+in+the+ischemic+burden+post-acute+myocardial+infarction%3A+prognostic+implications | | | |
| 167 | Journal of cardiothoracic surgery | Eid A H | 2009 | 0 | http://www.ncbi.nlm.nih.gov/pubmed/?term=Primary+congenital+anomalies+of+the+coronary+arteries+and+relation+to+atherosclerosis%3A+an+angiographic+study+in+Lebanon | | | |
| 238 | European journal of endocrinology | Gannage-Yared M H | 2009 | 0 | http://www.ncbi.nlm.nih.gov/pubmed/?term=Vitamin+D+in+relation+to+metabolic+risk+factors%2C+insulin+sensitivity+and+adiponectin+in+a+young+Middle-Eastern+population | | | |
| 244 | Addiction | Ghandour L A | 2009 | 0 | http://www.ncbi.nlm.nih.gov/pubmed/?term=Lifetime+alcohol+use%2C+abuse+and+dependence+among+university+students+in+Lebanon%3A+exploring+the+role+of+religiosity+in+different+religious+faiths | | | |
| 273 | European archives of oto-rhino-laryngology | Hamdan A L | 2009 | 0 | http://www.ncbi.nlm.nih.gov/pubmed/?term=Vocal+changes+following+radiotherapy+to+the+head+and+neck+for+non-laryngeal+tumors | | | |
| 282 | Annals of surgical oncology | Hatoum Hassan A | 2009 | 0 | http://www.ncbi.nlm.nih.gov/pubmed/?term=hatoum+Hassan+2009+Ratio+between+positive+lymph+nodes+and+total+excised+axillary+lymph+nodes+as+an+independent+prognostic+factor+for+overall+survival+in+patients+with+nonmetastatic+lymph+node-positive+breast+cancer | | | |
| 291 | European journal of echocardiography | Hussein A | 2009 | 0 | http://www.ncbi.nlm.nih.gov/pubmed/?term=hussein+A+2009+Value+of+aortic+arch+analysis+during+routine+transthoracic+echocardiography+in+adults | | | |
| 294 | The international journal of cardiovascular imaging | Isma'eel H | 2009 | 0 | http://www.ncbi.nlm.nih.gov/pubmed/?term=Optimal+phase+for+coronary+interpretations+and+correlation+of+ejection+fraction+using+late-diastole+and+end-diastole+imaging+in+cardiac+computed+tomography+angiography%3A+implications+for+prospective+triggering | | | |
| 297 | Journal of nuclear cardiology | Itani S | 2009 | 0 | http://www.ncbi.nlm.nih.gov/pubmed/?term=Variations+in+the+referral+patterns+to+stress+nuclear+imaging+and+stress+echocardiography+scans | | | |
| 392 | J Med Liban | Nasr E | 2009 | 0 | http://www.ncbi.nlm.nih.gov/pubmed/?term=Outcome+and+prognostic+factors+in+the+conservative+treatment+of+breast+cancer+nasr+2009 | | | |
| 413 | The Journal of cardiovascular nursing | Noureddine S | 2009 | 0 | http://www.ncbi.nlm.nih.gov/pubmed/?term=Patterns+of+responses+to+cardiac+events+over+time+noureddine+2009 | | | |
| 446 | J Med Liban | Saade G | 2009 | 0 | http://www.ncbi.nlm.nih.gov/pubmed/?term=Tobacco+use+and+cessation+counseling+among+health+professional+students%3A+Lebanon+Global+Health+Professions+Student+Survey | | | |
| 473 | Canadian journal of ophthalmology | Salti H I | 2009 | 0 | http://www.ncbi.nlm.nih.gov/pubmed/?term=Prevalence+and+determinants+of+retinopathy+in+a+cohort+of+Lebanese+type+II+diabetic+patients | | | |
| 508 | Food additives & contaminants | Soubra L | 2009 | 0 | http://www.ncbi.nlm.nih.gov/pubmed/?term=Occurrence+of+total+aflatoxins%2C+ochratoxin+A+and+deoxynivalenol+in+foodstuffs+available+on+the+Lebanese+market+and+their+impact+on+dietary+exposure+of+children+and+teenagers+in+Beirut | | | |
| 535 | Journal of public health | Yeretzian J S | 2009 | 0 | http://www.ncbi.nlm.nih.gov/pubmed/?term='It+won't+happen+to+me'%3A+the+knowledge-attitude+nexus+in+adolescent+smoking+2009 | | | |
| 650 | Clinical imaging | Khoury NJ | 2009 | 0 | http://www.ncbi.nlm.nih.gov.ezproxy.aub.edu.lb/pubmed/19559346 | | | |
| 707 | Clin Vaccine Immunol | Stayoussef M | 2009 | 0 | http://www.ncbi.nlm.nih.gov/pubmed/19005023 | | | |
| 432 | Journal of interventional cardiology | Rebeiz A G | 2009 | 0 | http://www.ncbi.nlm.nih.gov/pubmed/?term=Comparison+of+the+systemic+levels+of+inflammatory+markers+after+percutaneous+coronary+intervention+with+bare+metal+versus+sirolimus-eluting+stents | | | |
| 472 | Ophthalmic surgery, lasers & imaging | Salti H I | 2009 | 0 | http://www.ncbi.nlm.nih.gov/pubmed/?term=Enhancing+nonmydriatic+color+photographs+of+the+retina+with+monochromatic+views+and+a+stereo+pair+to+detect+diabetic+retinopathy | | | |
| 504 | International journal of cardiology | Sleilaty G | 2009 | 0 | http://www.ncbi.nlm.nih.gov/pubmed/?term=Postoperative+oral+amiodarone+versus+oral+bisoprolol+as+prophylaxis+against+atrial+fibrillation+after+coronary+artery+bypass+graft+surgery%3A+a+prospective+randomized+tria | | | |
| 3 | Journal of cutaneous pathology | Abbas O | 2009 | 0 | http://www.ncbi.nlm.nih.gov/pubmed/?term=Cutaneous+sebaceous+neoplasms+as+markers+of+Muir-Torre+syndrome%3A+a+diagnostic+algorithm | | | |
| 32 | J Med Liban | Adib S M | 2009 | 0 | http://www.ncbi.nlm.nih.gov/pubmed/19623881 | | | |
| 33 | Eastern Mediterranean health journal | Adib S M | 2009 | 0 | http://www.ncbi.nlm.nih.gov/pubmed/?term=Research+in+action%3A+mammography+utilization+following+breast+cancer+awareness+campaigns+in+Lebanon+2002-05 | | | |
| 113 | Archives of pathology & laboratory medicine | Chakhachiro Z I | 2009 | 0 | http://www.ncbi.nlm.nih.gov/pubmed/19961258 | | | |
| 150 | Pediatric hematology and oncology | Dardas M | 2009 | 0 | http://www.ncbi.nlm.nih.gov/pubmed/?term=Thyroid+cancer+in+Lebanese+children+and+adolescents%3A+a+15-year+experience+at+a+single+institution | | | |
| 258 | J Med Liban | Ghossain A | 2009 | 0 | http://www.ncbi.nlm.nih.gov/pubmed/19623880 | | | |
| 288 | J Med Liban | Hourani M H | 2009 | 0 | http://www.ncbi.nlm.nih.gov/pubmed/?term=Hourani%2CM.H.%3B+Nassar%2CL.%3B+Haydar%2CM.%3B+Hourany-Rizk%2C+R.+G.+FoldersLebanon%3B++TitleImaging+in+oncology+Pub+Year2009 | | | |
| 289 | J Med Liban | Hourani R | 2009 | 0 | http://www.ncbi.nlm.nih.gov/pubmed/?term=Imaging+of+common+neurological+disorders+hourani+M+2009+hourani+M+h | | | |
| 352 | Immunopharmacology and immunotoxicology | Makkouk A | 2009 | 0 | http://www.ncbi.nlm.nih.gov/pubmed/?term=The+potential+use+of+Toll-like+receptor+(TLR)+agonists+and+antagonists+as+prophylactic+and%2For+therapeutic+agents+makkouk++2009 | | | |
| 358 | Nutrition and health | Mattar M | 2009 | 0 | http://www.ncbi.nlm.nih.gov/pubmed/?term=Fish+oil+and+the+management+of+hypertriglyceridemia+2008+mattar+M | | | |
| 379 | J Med Liban | Naccache N | 2009 | 0 | http://www.ncbi.nlm.nih.gov/pubmed/19623887 | | | |
| 389 | Tobacco control | Nakkash R | 2009 | 0 | http://www.ncbi.nlm.nih.gov/pubmed/?term=The+tobacco+industry's+thwarting+of+marketing+restrictions+and+health+warnings+in+Lebanon | | | |
| 402 | J Med Liban | Nasser S M | 2009 | 0 | http://www.ncbi.nlm.nih.gov/pubmed/?term=nasser+2009+Flat+epithelial+atypia+of+the+breast | | | |
| 403 | J Med Liban | Nasser S M | 2009 | 0 | http://www.ncbi.nlm.nih.gov/pubmed/?term=Gene+expression+profiling+in+breast+cancer+nasser+2009+Gene+expression+profiling+has+been+increasingly+used+to+determine+new+cancer+markers.+This+technology+holds+major+promises+for | | | |
| 421 | Critical reviews in oncology/hematology | Otrock Z K | 2009 | 0 | http://www.ncbi.nlm.nih.gov/pubmed/?term=Hypoxia-inducible+factor+in+cancer+angiogenesis%3A+structure%2C+regulation+and+clinical+perspectives | | | |
| 468 | Asian Pacific journal of cancer prevention | Salim E I | 2009 | 0 | http://www.ncbi.nlm.nih.gov/pubmed/?term=Cancer+epidemiology+and+control+in+the+arab+world+-+past%2C+present+and+future++salim+2009 | | | |
| 673 | Clinical cancer research : an official journal of the American Association for Cancer Research | Nasr R | 2009 | 0 | http://www.ncbi.nlm.nih.gov.ezproxy.aub.edu.lb/pubmed/19808868 | | | |
| 674 | Am J of perinatology | Nassar AH | 2009 | 0 | http://www.ncbi.nlm.nih.gov.ezproxy.aub.edu.lb/pubmed/19021096 | | | |
| 1025 | Thrombosis research | Musallam KM | 2009 | 0 | http://www.ncbi.nlm.nih.gov/pubmed/18992924 | | | |
| 19 | Human mutation | Abifadel M | 2009 | 0 | http://www.ncbi.nlm.nih.gov/pubmed/?term=The+molecular+basis+of+familial+hypercholesterolemia+in+Lebanon%3A+spectrum+of+LDLR+mutations+and+role+of+PCSK9+as+a+modifier+gene | | | |
| 333 | Tobacco control | Khalil J | 2009 | 0 | http://www.ncbi.nlm.nih.gov/pubmed/?term=The+tobacco+health+nexus%3F+Health+messages+in+narghile+advertisements+2009 | | | |
| 351 | Health promotion practice | Makhoul J | 2009 | 0 | http://www.ncbi.nlm.nih.gov/pubmed/?term=Understanding+youth%3A+using+qualitative+methods+to+verify+quantitative+community+indicators | | | |
| 436 | Clinical lymphoma & myeloma | Rizk S | 2009 | 0 | http://www.ncbi.nlm.nih.gov/pubmed/?term=The+antiproliferative+effect+of+kefir+cell-free+fraction+on+HuT-102+malignant+T+lymphocytes | | | |
| 551 | Am J Physiol Heart Circ Physiol | Abboud K | 2009 | 0 | http://www.ncbi.nlm.nih.gov/pubmed/19542492 | | | |
| 583 | J Ethnopharmacol | Cardile V | 2009 | 0 | http://www.ncbi.nlm.nih.gov/pubmed/19715748 | | | |
| 628 | Radiation Oncology | Haykal J | 2009 | 0 | http://www.ncbi.nlm.nih.gov/pubmed/?term=The+radiosensitizer+2-benzoyl-3-phenyl-6%2C7-dichloroquinoxaline+1%2C4-dioxide+induces+DNA+damage+in+EMT-6+mammary+carcinoma+cells. | | | |
| 688 | Cancer Research | Saab R | 2009 | 0 | http://www.ncbi.nlm.nih.gov.ezproxy.aub.edu.lb/pubmed/?term=p18Ink4c+and+p53+Act+as+tumor+suppressors+in+cyclin+D1-driven+primitive+neuroectodermal+tumor. | | | |
| 738 | Molecular biology reports | Sabbagh AS | 2009 | 0 | http://www.ncbi.nlm.nih.gov/pubmed/?term=Prevalence+of+the+prothrombin+G20210A+polymorphism+in+the+Lebanese+population%3A+use+of+a+reverse+hybridization+strip+assay+approach. | | | |
| 962 | JAMA | Dakik HA | 2009 | 0 | http://www.ncbi.nlm.nih.gov/pubmed/19567432 | | | |
| 989 | International journal of cardiology | Isma'eel H | 2009 | 0 | http://www.ncbi.nlm.nih.gov/pubmed/18164497 | | | |
| 1024 | J Natl Cancer Inst | Musallam KM | 2009 | 0 | http://www.ncbi.nlm.nih.gov/pubmed/19858426 | | | |
| 474 | Diabetic medicine | Salti I | 2009 | 0 | http://www.ncbi.nlm.nih.gov/pubmed/?term=Efficacy+and+safety+of+insulin+glargine+and+glimepiride+in+subjects+with+Type+2+diabetes+before%2C+during+and+after+the+period+of+fasting+in+Ramadan | | | |
| 645 | The International Journal of Tuberculosis and Lung Disease | Khadadah M | 2009 | 0 | http://www.ncbi.nlm.nih.gov/pubmed/19723383 | | | |
| 21 | International surgery | Abou-Jaoude M | 2009 | 0 | http://www.ncbi.nlm.nih.gov/pubmed/20187510 | | | |
| 42 | Hereditary cancer in clinical practice | Akoum R | 2009 | 0 | http://www.ncbi.nlm.nih.gov/pubmed/?term=Early-onset+breast+cancer+in+a+Lebanese+family+with+Lynch+syndrome+due+to+MSH2+gene+mutation | | | |
| 49 | Journal of physical activity & health | Al-Tannir M | 2009 | 0 | http://www.ncbi.nlm.nih.gov/pubmed/19564659 | | | |
| 68 | Blood coagulation & fibrinolysis | Awar Z | 2009 | 0 | http://www.ncbi.nlm.nih.gov/pubmed/19587584 | | | |
| 371 | The Journal of asthma | Musharrafieh U | 2009 | 0 | http://www.ncbi.nlm.nih.gov/pubmed/19484674 | | | |
| 528 | Eastern Mediterranean health journal | Waked M | 2009 | 0 | http://www.ncbi.nlm.nih.gov/pubmed/?term=Water-pipe+(narguile)+smokers+in+Lebanon%3A+a+pilot+study | | | |
| 69 | European journal of anaesthesiology | Ayoub C M | 2009 | 0 | http://www.ncbi.nlm.nih.gov/pubmed/?term=Prophylactic+amiodarone+versus+lidocaine+for+prevention+of+reperfusion+ventricular+fibrillation+after+release+of+aortic+cross-clamp | | | |
| 16 | The Thoracic and cardiovascular Surgeon | Achir A | 2009 | 0 | http://www.ncbi.nlm.nih.gov/pubmed/19241318 | | | |
| 22 | Cases journal | Ahallal Y | 2009 | 0 | http://www.ncbi.nlm.nih.gov/pubmed/20062669 | | | |
| 35 | Internal medicine | Akhaddar A | 2009 | 0 | http://www.ncbi.nlm.nih.gov/pubmed/19293554 | | | |
| 101 | Eastern Mediterranean Health Journal | Benmansour N | 2009 | 1 | http://www.ncbi.nlm.nih.gov/pubmed/20214149 | | | |
| 146 | Cancer Radiothérapie | Bourhaleb Z | 2009 | 1 | http://www.ncbi.nlm.nih.gov/pubmed/19695931 | | | |
| 151 | Cases Journal | Brahmi SA | 2009 | 0 | http://www.ncbi.nlm.nih.gov/pubmed/19829939 | | | |
| 224 | Functional Neurology | El Otmani H | 2009 | 0 | http://www.ncbi.nlm.nih.gov/pubmed/20018139 | | | |
| 235 | International Journal of Dermatology | Elghissassi I | 2009 | 0 | http://www.ncbi.nlm.nih.gov/pubmed/19416377 | | | |
| 237 | Cases Journal | Elhassani LK | 2009 | 0 | http://www.ncbi.nlm.nih.gov/pubmed/19829837 | | | |
| 282 | Presse médicale | Harifi G | 2009 | 1 | http://www.ncbi.nlm.nih.gov/pubmed/19386466 | | | |
| 286 | Journal of clinical rheumatology | Harzy T | 2009 | 0 | http://www.ncbi.nlm.nih.gov/pubmed/19131765 | | | |
| 287 | Journal of clinical rheumatology | Harzy T | 2009 | 0 | http://www.ncbi.nlm.nih.gov/pubmed/19349797 | | | |
| 314 | Journal of cancer research and therapeutics | Ismaili N | 2009 | 0 | http://www.ncbi.nlm.nih.gov/pubmed/19542673 | | | |
| 478 | J Gastrointest Cancer | Soufi M | 2009 | 0 | http://www.ncbi.nlm.nih.gov/pubmed/19728181 | | | |
| 488 | Rheumatology International | Tahiri L | 2009 | 0 | http://www.ncbi.nlm.nih.gov/pubmed/19337735 | | | |
| 495 | Cases Journal | Tazzi EM | 2009 | 0 | http://www.ncbi.nlm.nih.gov/pubmed/20062662 | | | |
| 518 | Annals of Burns and Fire Disasters | Tourabi K | 2009 | 1 | http://www.ncbi.nlm.nih.gov/pubmed/21991185 | | | |
| 736 | Annals of Saudi Medicine | Tazi I | 2009 | 0 | http://www.ncbi.nlm.nih.gov/pubmed/19700904 | | | |
| 737 | Cancer Radiothérapie | Tazi I | 2009 | 0 | http://www.ncbi.nlm.nih.gov/pubmed/19692278 | | | |
| 744 | Cancer Radiothérapie | Sbitti Y | 2009 | 1 | http://www.ncbi.nlm.nih.gov/pubmed/19097926 | | | |
| 881 | Progrès en urologie | Ammani A | 2009 | 1 | http://www.ncbi.nlm.nih.gov/pubmed/19699458 | | | |
| 884 | Annales d'Endocrinologie | BaÃ¯zri H | 2009 | 1 | http://www.ncbi.nlm.nih.gov/pubmed/19591972 | | | |
| 885 | Journal de chirurgie | Rifki Jai S | 2009 | 1 | http://www.ncbi.nlm.nih.gov/pubmed/19524241 | | | |
| 888 | Revue neurologique | Bougteba A | 2009 | 1 | http://www.ncbi.nlm.nih.gov/pubmed/19446857 | | | |
| 889 | Revue de pneumologie clinique | Zidane A | 2009 | 1 | http://www.ncbi.nlm.nih.gov/pubmed/19375048 | | | |
| 890 | Annales françaises d'Oto-rhino-laryngologie et de Pathologie Cervico-faciale | Dib N | 2009 | 1 | http://www.ncbi.nlm.nih.gov/pubmed/19296927 | | | |
| 891 | Annales de Dermatologie et de Vénéréologie | Abilkassem R | 2009 | 1 | http://www.ncbi.nlm.nih.gov/pubmed/19171242 | | | |
| 893 | Revue neurologique | El Moutawakil B | 2009 | 1 | http://www.ncbi.nlm.nih.gov/pubmed/19144365 | | | |
| 13 | Eastern Mediterranean Health Journal | Abir-Khalil S | 2009 | 0 | http://www.ncbi.nlm.nih.gov/pubmed/19554987 | | | |
| 96 | Annales de Cardiologie et d'Angéiologie | Benjelloun H | 2009 | 1 | http://www.ncbi.nlm.nih.gov/pubmed/18678361 | | | |
| 144 | Respiratory Medicine | Bourdin A | 2009 | 0 | http://www.ncbi.nlm.nih.gov/pubmed/20122628 | | | |
| 169 | Pediatric Hematology and Oncology | Dakka N | 2009 | 0 | http://www.ncbi.nlm.nih.gov/pubmed/19437324 | | | |
| 206 | Respiratory medicine | El Ftouh M | 2009 | 0 | http://www.ncbi.nlm.nih.gov/pubmed/20122626 | | | |
| 210 | Respiratory medicine | El Hasnaoui A | 2009 | 0 | http://www.ncbi.nlm.nih.gov/pubmed/20122627 | | | |
| 231 | Eastern Mediterranean Health Journal | El Taquri A | 2009 | 0 | http://www.ncbi.nlm.nih.gov/pubmed/19731771 | | | |
| 247 | Cancer Detection and Prevention | Ezzikouri S | 2009 | 0 | http://www.ncbi.nlm.nih.gov/pubmed/19233569 | | | |
| 248 | Infection, genetics and evolution : journal of molecular epidemiology and evolutionary genetics in i | Ezzikouri S | 2009 | 0 | http://www.ncbi.nlm.nih.gov/pubmed/19465161 | | | |
| 257 | British journal of cancer | Feng BJ | 2009 | 0 | http://www.ncbi.nlm.nih.gov/pubmed/19724280 | | | |
| 267 | Nutricion Hospitalaria | Guerrero Morilla R | 2009 | 1 | http://www.ncbi.nlm.nih.gov/pubmed/20049379 | | | |
| 281 | Archives de pédiatrie | Harif M | 2009 | 1 | http://www.ncbi.nlm.nih.gov/pubmed/19541126 | | | |
| 299 | BMC Public Health | Hmamouchi I | 2009 | 0 | http://www.ncbi.nlm.nih.gov/pubmed/19828021 | | | |
| 303 | Ethnicity & health | Hoopman R | 2009 | 0 | http://www.ncbi.nlm.nih.gov/pubmed/19012066 | | | |
| 315 | Radiation oncology | Ismaili N | 2009 | 0 | http://www.ncbi.nlm.nih.gov/pubmed/19351405 | | | |
| 413 | J Chir (Paris) | Mssrouri R | 2009 | 1 | http://www.ncbi.nlm.nih.gov/pubmed/19446693 | | | |
| 420 | Respiratory Medicine | Nafti S | 2009 | 0 | http://www.ncbi.nlm.nih.gov/pubmed/20122625 | | | |
| 677 | European Archives of Oto-Rhino-Laryngology | Afqir S | 2009 | 0 | http://www.ncbi.nlm.nih.gov/pubmed/19159940 | | | |
| 883 | Presse médicale | Benomar S | 2009 | 1 | http://www.ncbi.nlm.nih.gov/pubmed/19643562 | | | |
| 894 | Cancer radiothérapie | Bouhafa T | 2009 | 1 | http://www.ncbi.nlm.nih.gov/pubmed/19119040 | | | |
| 194 | Diabetes and Metabolism | ElAchhab Y | 2009 | 1 | http://www.ncbi.nlm.nih.gov/pubmed/19046915 | | | |
| 21 | Journal of cancer research and therapeutics | Afqir S | 2009 | 0 | http://www.ncbi.nlm.nih.gov/pubmed/19293481 | | | |
| 77 | Journal of Neurological Sciences | Benamer H T | 2009 | 1 | http://www.ncbi.nlm.nih.gov/pubmed/19428027 | | | |
| 887 | Bulletin du Cancer | Ismaili N | 2009 | 1 | http://www.ncbi.nlm.nih.gov/pubmed/19457759 | | | |
| 11 | Journal of cancer research and therapeutics | Abdelhakim A | 2009 | 0 | http://www.ncbi.nlm.nih.gov/pubmed/19841562 | | | |
| 73 | Biochem Biophys Res Commun | Belmokhtar M | 2009 | 0 | http://www.ncbi.nlm.nih.gov/pubmed/19715668 | | | |
| 76 | Nat Prod Res | Benamar M | 2009 | 0 | http://www.ncbi.nlm.nih.gov/pubmed/19401921 | | | |
| 209 | Blood cells, molecules & diseases | El hauoari M | 2009 | 0 | http://www.ncbi.nlm.nih.gov/pubmed/18829351 | | | |
| 229 | Rev Epidemiol Sante Publique | El Rhazi K | 2009 | 1 | http://www.ncbi.nlm.nih.gov/pubmed/19409741 | | | |
| 351 | The Scientific World Journal | Lahmiti S | 2009 | 0 | http://www.ncbi.nlm.nih.gov/pubmed/19347229 | | | |
| 527 | Clinical and experimental Hypertension | Zeggwagh NA | 2009 | 0 | http://www.ncbi.nlm.nih.gov/pubmed/19811353 | | | |
| 882 | Thérapie | Abdeljebbar LH | 2009 | 1 | http://www.ncbi.nlm.nih.gov/pubmed/19664406 | | | |
| 425 | Int J Public Health | Nejjari C | 2009 | 0 | http://www.ncbi.nlm.nih.gov/pubmed/19851709 | | | |
| 99 | Respiratory Medicine | Benkheder A | 2009 | 0 | http://www.ncbi.nlm.nih.gov/pubmed/20122624 | | | |
| 55 | Oncol Res | Attaleb M | 2009 | 0 | http://www.ncbi.nlm.nih.gov/pubmed/20112504 | | | |
| 336 | Journal of Medical Virology | Khair MM | 2009 | 0 | http://www.ncbi.nlm.nih.gov/pubmed/19235879 | | | |
| 132 | Néphrologie & thérapeutique | Bouattar T | 2009 | 1 | http://www.ncbi.nlm.nih.gov/pubmed/19269914 | | | |
| 512 | Eastern Mediterranean Health Journal | Tazi MA | 2009 | 0 | http://www.ncbi.nlm.nih.gov/pubmed/20187534 | | | |
| 285 | European journal of cancer prevention | Hartman E | 2009 | 0 | http://www.ncbi.nlm.nih.gov/pubmed/19581808 | | | |
| 157 | Fundamental & Clinical Pharmacology | Chaouki W | 2009 | 0 | http://www.ncbi.nlm.nih.gov/pubmed/19656204 | | | |
| 158 | Die Pharmazie | Chaouki W | 2009 | 0 | http://www.ncbi.nlm.nih.gov/pubmed/19746845 | | | |
| 390 | Cellular and molecular biology | Meftah E K M | 2009 | 0 | http://www.ncbi.nlm.nih.gov/pubmed/20003812 | | | |
| 143 | ONS connect | Saca-Hazboun H | 2009 | 0 | http://www.ncbi.nlm.nih.gov.ezproxy.aub.edu.lb/pubmed/19777874 | | | |
| 144 | ONS connect | Saca-Hazboun H | 2009 | 0 | http://www.ncbi.nlm.nih.gov.ezproxy.aub.edu.lb/pubmed/19715060 | | | |
| 36 | PPAR Res | Ereqat S | 2009 | 0 | http://www.ncbi.nlm.nih.gov/pubmed/19859551 | | | |
| 37 | Int J Clin Pharm TH | Sweileh WM | 2009 | 0 | http://www.ncbi.nlm.nih.gov/pubmed/19640349 | | | |
| 38 | BMC Public Health | Abudayya AH | 2009 | 0 | http://www.ncbi.nlm.nih.gov/pubmed/19527503 | | | |
| 41 | International journal of clinical and experimental medicine | Sweileh WM | 2009 | 0 | http://www.ncbi.nlm.nih.gov/pubmed/19436831 | | | |
| 42 | J Stroke Cerebrovasc Dis | Sweileh WM | 2009 | 0 | http://www.ncbi.nlm.nih.gov/pubmed/19251184 | | | |
| 43 | Pulm Pharmacol Ther | Al Zabadi H | 2009 | 0 | http://www.ncbi.nlm.nih.gov/pubmed/19138752 | | | |
| 44 | The Libyan journal of medicine | Sawalha A | 2009 | 0 | http://www.ncbi.nlm.nih.gov/pubmed/21483501 | | | |
| 118 | Annals of Saudi medicine | Swaileh WM | 2009 | 0 | http://www.ncbi.nlm.nih.gov.ezproxy.aub.edu.lb/pubmed/19318756 | | | |
| 142 | Indian journal of pediatrics | Radi S | 2009 | 0 | http://www.ncbi.nlm.nih.gov.ezproxy.aub.edu.lb/pubmed/19330304 | | | |
| 149 | The Canadian journal of clinical pharmacology = Journal canadien de pharmalcologie clinique | Sameer AE | 2009 | 0 | http://www.ncbi.nlm.nih.gov.ezproxy.aub.edu.lb/pubmed/19193970 | | | |
| 151 | Current drug safety | Sweileh WM | 2009 | 0 | http://www.ncbi.nlm.nih.gov.ezproxy.aub.edu.lb/pubmed/19442103 | | | |
| 109 | Seminars in nuclear medicine | Palestro CJ | 2009 | 0 | http://www.ncbi.nlm.nih.gov.ezproxy.aub.edu.lb/pubmed/19038600 | | | |
| 100 | Health promotion practice | Makhoul J | 2009 | 0 | http://www.ncbi.nlm.nih.gov.ezproxy.aub.edu.lb/pubmed/17971480 | | | |
| 115 | Annals of cardiac anaesthesia | Skaik YA | 2009 | 0 | http://www.ncbi.nlm.nih.gov.ezproxy.aub.edu.lb/pubmed/19602755 | | | |
| 128 | Journal of pain and symptom management | Bingley A | 2009 | 0 | http://www.ncbi.nlm.nih.gov.ezproxy.aub.edu.lb/pubmed/18823750 | | | |
| 39 | Prev Med | Khader A | 2009 | 0 | http://www.ncbi.nlm.nih.gov/pubmed/19520108 | | | |
| 47 | J Hum Nutr Diet | Al Sabbah H | 2009 | 0 | http://www.ncbi.nlm.nih.gov/pubmed/18759957 | | | |
| 103 | BMC public health | Mikki N | 2009 | 0 | http://www.ncbi.nlm.nih.gov.ezproxy.aub.edu.lb/pubmed/20030822 | | | |
| 72 | Lancet | Husseini A | 2009 | 0 | http://icph.birzeit.edu/uploads/File/lancet99/3-%20cardiovascular%203-%20diseases%20diabetes%20cancer%20in%20oPt%20Abed%20etc%202009.pdf | | | |
| 33 | Eastern Mediterranean health journal = La revue de sante de la Medoterranee orientale = al-Majallah | Abu Sham'a RA | 2009 | 0 | http://www.ncbi.nlm.nih.gov/pubmed/20218139 | | | |
| 34 | Journal of health care finance | Jabr SF | 2009 | 0 | http://www.ncbi.nlm.nih.gov/pubmed/19891208 | | | |
| 27 | Saudi medical journal | Ahmed M E | 2009 | 0 | http://www.ncbi.nlm.nih.gov/pubmed/19882060 | | | |
| 1 | Psycho-oncology | Abasher S M | 2009 | 0 | http://www.ncbi.nlm.nih.gov/pubmed/?term=Sexual+health+issues+in+Sudanese+women+before+and+during+hormonal+treatment+for+breast+cancer | | | |
| 2 | Eastern Mediterranean health journal | Abdel G A | 2009 | 0 | http://www.ncbi.nlm.nih.gov/pubmed/?term=Plasma+homocysteine+levels+in+cardiovascular+disease%2C+malaria+and+protein-energy+malnutrition+in+Sudan | | | |
| 7 | The Tohoku journal of experimental medicine | Abdelgadir M | 2009 | 0 | Health related quality of life and sense of coherence in Sudanese diabetic subjects with lower limb amputation | | | |
| 16 | Diagnostic cytopathology | Ahmed H G | 2009 | 0 | https://www.ncbi.nlm.nih.gov.ezproxy.aub.edu.lb/pubmed/?term=Utility+of+fine-needle+aspiration+as+a+diagnostic+technique+in+breast+lumps | | | |
| 18 | Rare Tumors | Ahmed H G | 2009 | 0 | http://www.ncbi.nlm.nih.gov/pubmed/?term=Assessment+of+cytological+atypia%2C+AgNOR+and+nuclear+area+in+epithelial+cells+of+normal+oral+mucosa+exposed+to+toombak+and+smoking | | | |
| 20 | CytoJournal | Ahmed H G | 2009 | 0 | http://www.ncbi.nlm.nih.gov/pubmed/19495410 | | | |
| 72 | The Gulf journal of oncology | Hassan F M | 2009 | 0 | http://www.ncbi.nlm.nih.gov/pubmed/20194088 | | | |
| 117 | Saudi Journal of Kidney Disease and Transplantation | Suleiman B | 2009 | 0 | http://www.ncbi.nlm.nih.gov/pubmed/?term=Lipid+profile+in+post+renal+transplant+patients+treated+with+cyclosporine+in+Sudan | | | |
| 43 | Journal of the neurological sciences | Benamer H T | 2009 | 0 | http://www.ncbi.nlm.nih.gov/pubmed/19428027 | | | |
| 66 | European journal of pharmaceutics and biopharmaceutics | Elsayed A | 2009 | 0 | http://www.ncbi.nlm.nih.gov/pubmed/?term=Formulation+and+characterization+of+an+oily-based+system+for+oral+delivery+of+insulin | | | |
| 85 | Lakartidningen | Lindblom D | 2009 | 1 | https://www.ncbi.nlm.nih.gov.ezproxy.aub.edu.lb/pubmed/?term=%5BHeart+surgery+in+Sudan.+Experiences+from+a+highly+specialized+cardiac+surgical+centre+managed+by+an+Italian+humanitarian+organization%5D | | | |
| 40 | Diabetes Research and Clinical Practice | Whitford DL | 2010 | 0 | http://www.sciencedirect.com/science/article/pii/S0168822710004080# | | | |
| 41 | Saudi Medical Journal | Al-Bannay R | 2010 | 0 | http://www.ncbi.nlm.nih.gov/pubmed/?term=Hypertensive+crisis.+Clinical+presentation%2C+comorbidities%2C+and+target+organ+involvement. | | | |
| 42 | BMC Complementary and Alternative Medicine | Khalaf AJ | 2010 | 0 | http://www.ncbi.nlm.nih.gov/pmc/articles/PMC2912778/pdf/1472-6882-10-35.pdf | | | |
| 102 | Clinical chemistry and laboratory medicine | Nemr R | 2010 | 0 | http://www.ncbi.nlm.nih.gov/pubmed/?term=Differential+contribution+of+MTHFR+C677T+variant+to+the+risk+of+diabetic+nephropathy+in+Lebanese+and+Bahraini+Arabs+20120 | | | |
| 103 | Tobacco control | Nakkash R | 2010 | 0 | http://www.ncbi.nlm.nih.gov/pubmed/20501497 | | | |
| 37 | Eastern Mediterranean Health Journal | Mirmiran P | 2010 | 0 | http://www.ncbi.nlm.nih.gov.ezproxy.aub.edu.lb/pubmed/21218730 | | | |
| 43 | International Journal of Surgery | Najjar H | 2010 | 0 | http://www.ncbi.nlm.nih.gov.ezproxy.aub.edu.lb/pubmed/20601253 | | | |
| 45 | Journal of the Neurological Sciences | Tran J | 2010 | 0 | http://www.ncbi.nlm.nih.gov.ezproxy.aub.edu.lb/pubmed/20541222 | | | |
| 46 | Diabetic Medicine | Mabry RM | 2010 | 0 | http://www.ncbi.nlm.nih.gov/pubmed/?term=Gender+differences+in+prevalence+of+the+metabolic+syndrome+in+Gulf+Cooperation+Council+Countries%3A+a+systematic+9 | | | |
| 105 | Cochrane Database Syst Rev | Amin F | 2010 | 0 | http://www.ncbi.nlm.nih.gov/pubmed/20166092 | | | |
| 201 | BMJ Case Reports | Amin OS | 2010 | 0 | http://www.ncbi.nlm.nih.gov/pubmed/22766571 | | | |
| 45 | Journal of the Pakistan Medical Association | Al-Abdulla NO | 2010 | 0 | http://www.ncbi.nlm.nih.gov/pubmed/21381556 | | | |
| 47 | Eastern Mediterranean Health Journal | Alwan NA | 2010 | 0 | http://www.ncbi.nlm.nih.gov/pubmed/21218740 | | | |
| 53 | Saudi Medical Journal | Awad SM | 2010 | 0 | http://www.ncbi.nlm.nih.gov/pubmed/20844816 | | | |
| 56 | Eastern Mediterranean Health Journal | Al-Asadi N | 2010 | 0 | http://www.ncbi.nlm.nih.gov/pubmed/20799530 | | | |
| 57 | International Journal of Environmental Research and Public Health | Busby C | 2010 | 0 | http://www.ncbi.nlm.nih.gov/pubmed/20717542 | | | |
| 61 | Neurosciences | Khidhir AJ | 2010 | 0 | http://www.ncbi.nlm.nih.gov/pubmed/20677587 | | | |
| 62 | Neurosciences | Al-Nimer MS | 2010 | 0 | http://www.ncbi.nlm.nih.gov/pubmed/20672493 | | | |
| 67 | Medical Principles and Practice | Mansour AA | 2010 | 0 | http://www.ncbi.nlm.nih.gov/pubmed/20357499 | | | |
| 70 | American Journal of Public Health | Hagopian A | 2010 | 0 | http://www.ncbi.nlm.nih.gov/pubmed/20167894 | | | |
| 72 | Libyan Journal of Medicine | Hashim AF | 2010 | 0 | http://www.ncbi.nlm.nih.gov/pubmed/21483581 | | | |
| 200 | Neurosciences | Hasan ZN | 2010 | 0 | http://www.ncbi.nlm.nih.gov/pubmed/20672495 | | | |
| 51 | Saudi Pharmaceutical Journal | Al-Hamdani FY | 2010 | 0 | http://www.ncbi.nlm.nih.gov/pubmed/23960734 | | | |
| 55 | Allergy and Asthma Proceedings | Alzakar RH | 2010 | 0 | http://www.ncbi.nlm.nih.gov/pubmed/20819323 | | | |
| 63 | Asian Pacific Journal of Cancer Prevention | Salim EI | 2010 | 0 | http://www.ncbi.nlm.nih.gov/pubmed/20553067 | | | |
| 64 | International Journal of Surgery | Najjar H | 2010 | 0 | http://www.ncbi.nlm.nih.gov/pubmed/20601253 | | | |
| 66 | BMC International Health and Human Rights | Tawfik-Shukor A | 2010 | 0 | http://www.ncbi.nlm.nih.gov/pubmed/20529346 | | | |
| 50 | Asian Pacific Journal of Cancer Prevention | Habib OS | 2010 | 0 | http://www.ncbi.nlm.nih.gov/pubmed/21133640 | | | |
| 58 | American Journal of Public Health | Greiser E | 2010 | 0 | http://www.ncbi.nlm.nih.gov/pubmed/20705960 | | | |
| 59 | Journal of oral pathology and medicine | Jawad SN | 2010 | 0 | http://www.ncbi.nlm.nih.gov/pubmed/20701666 | | | |
| 60 | Indian Journal of Pathology and Microbiology | Ahmed MM | 2010 | 0 | http://www.ncbi.nlm.nih.gov/pubmed/20699499 | | | |
| 65 | Head and Neck Oncology | Sarkis SA | 2010 | 0 | http://www.ncbi.nlm.nih.gov/pubmed/20579333 | | | |
| 68 | Archiv der Pharmazie | Al-Masoudi NA | 2010 | 0 | http://www.ncbi.nlm.nih.gov/pubmed/20222061 | | | |
| 54 | Neurosciences | Al-Shimmery EK | 2010 | 0 | http://www.ncbi.nlm.nih.gov/pubmed/20831024 | | | |
| 199 | Skinmed | Aldhalimi MA | 2010 | 0 | http://www.ncbi.nlm.nih.gov/pubmed/21137604 | | | |
| 22 | Annals of Saudi Medicine | Al-Adsani A | 2010 | 0 | http://www.ncbi.nlm.nih.gov/pubmed/20103962 | | | |
| 138 | Annals of Saudi Medicine | Al-Mahdi M | 2010 | 0 | http://www.ncbi.nlm.nih.gov/pubmed/?term=Successful+transfer+from+insulin+to+oral+sulfonylurea+in+a+3-year-old+girl+with+a+mutation+in+the+KCNJ11+gene | | | |
| 177 | International Urology and Nephrology | Mannan AA | 2010 | 0 | http://www.ncbi.nlm.nih.gov/pubmed/?term=An+unusual+case+of+extensive+epididymotesticular+malakoplakia+in+a+diabetic+patient | | | |
| 369 | Middle East African Journal of Ophthalmology | Al Baghli A | 2010 | 0 | http://www.ncbi.nlm.nih.gov/pubmed/21180442 | | | |
| 458 | General Thoracic and Cardiovascular Surgery | Al-Sarraf N | 2010 | 0 | http://www.ncbi.nlm.nih.gov/pubmed/20349302 | | | |
| 571 | The Gulf Journal of Oncology | Eshra A | 2010 | 0 | http://www.ncbi.nlm.nih.gov.ezproxy.aub.edu.lb/pubmed/20601338 | | | |
| 591 | Medical Principles and Practice | Hegazi MO | 2010 | 0 | http://www.ncbi.nlm.nih.gov.ezproxy.aub.edu.lb/pubmed/19996624 | | | |
| 615 | Acta Cytologica | Kapila K | 2010 | 0 | http://www.ncbi.nlm.nih.gov.ezproxy.aub.edu.lb/pubmed/?term=Expression+of+estrogen+receptor+alpha+and+estrogen+receptor+beta+in+fine+needle+aspirates+of+breast+carcinoma. | | | |
| 648 | Pathology Oncology Research | Mannan AA | 2010 | 0 | http://www.ncbi.nlm.nih.gov/pubmed/19757197 | | | |
| 658 | Indian Journal of Medical and Paediatric Oncology | Mittal R | 2010 | 0 | http://www.ncbi.nlm.nih.gov.ezproxy.aub.edu.lb//pubmed/20931020 | | | |
| 753 | Leukemia Research | Zamecnikova A | 2010 | 0 | http://www.ncbi.nlm.nih.gov/pubmed/20211490 | | | |
| 20 | Medical Principles and Practice | Saad H | 2010 | 0 | http://www.ncbi.nlm.nih.gov/pubmed/?term=Influence+of+diabetes+on+the+outcome+of+acute+coronary+syndrome+in+Kuwait | | | |
| 21 | Medical Principles and Practice | Shah, MA | 2010 | 0 | http://www.ncbi.nlm.nih.gov/pubmed/?term=Prevalence+and+correlates+of+major+chronic+illnesses+among+older+Kuwaiti+++++++nationals+in+two+governorates | | | |
| 104 | Pediatric Diabetes | Abdul-Rasoul M | 2010 | 0 | http://www.ncbi.nlm.nih.gov/pubmed/?term=Ketoacidosis+at+presentation+of+type+1+diabetes+in+children+in+Kuwait%3A+frequency+and+clinical+characteristics | | | |
| 114 | Angiology | Al Rashdan I | 2010 | 0 | http://www.ncbi.nlm.nih.gov.ezproxy.aub.edu.lb/pubmed/?term=Prevalence+of+overweight%2C+obesity%2C+and+metabolic+syndrome+among+adult+Kuwaitis%3A+results+from+community-based+national+survey | | | |
| 127 | British Journal of Nutrition | Al-Isa A | 2010 | 0 | http://www.ncbi.nlm.nih.gov/pubmed/19664298 | | | |
| 135 | Pediatric Diabetes | AlKhawari M | 2010 | 0 | http://www.ncbi.nlm.nih.gov/pubmed/?term=Adolescents+on+basal-bolus+insulin+can+fast+during+Ramadan | | | |
| 183 | Clinical Chemistry and Laboratory medicine | Mojiminiyi OA | 2010 | 0 | http://www.ncbi.nlm.nih.gov.ezproxy.aub.edu.lb/pubmed/?term=Effect+of+homeostasis+model+assessment+computational+method+on+the+definition+and+associations+of+insulin+resistance | | | |
| 334 | British Dental Journal | N/A | 2010 | 0 | http://www.ncbi.nlm.nih.gov/pubmed/20577237 | | | |
| 336 | Medical Principles and Practice | Abal AT | 2010 | 0 | http://www.ncbi.nlm.nih.gov/pubmed/20516706 | | | |
| 397 | Medical Principles and Practice | Al-Bahar S | 2010 | 0 | http://www.ncbi.nlm.nih.gov/pubmed/20357498 | | | |
| 416 | Atherosclerosis | Al-Isa AN | 2010 | 0 | http://www.ncbi.nlm.nih.gov/pubmed/19766219 | | | |
| 417 | International Journal of Pediatrics | Al-Isa AN | 2010 | 0 | http://www.ncbi.nlm.nih.gov/pubmed/20886010 | | | |
| 451 | Journal of Developmental and Behavioral Pediatrics | Al-Qaoud N | 2010 | 0 | http://www.ncbi.nlm.nih.gov/pubmed/20611037 | | | |
| 469 | Obesity Surgery | Alasfar F | 2010 | 0 | http://www.ncbi.nlm.nih.gov/pubmed/18839083 | | | |
| 470 | Annals of Saudi Medicine | Alawadhi SA | 2010 | 0 | http://www.ncbi.nlm.nih.gov/pubmed/20697165 | | | |
| 539 | Indian Journal of Pathology and Microbiology | Das DK | 2010 | 0 | http://www.ncbi.nlm.nih.gov/pubmed/21045393 | | | |
| 556 | Asian Pacific Journal of Cancer Prevention | Elbasmi AA | 2010 | 0 | http://www.ncbi.nlm.nih.gov/pubmed/21039045 | | | |
| 605 | Medical Principles and Practice | Jackson RT | 2010 | 0 | http://www.ncbi.nlm.nih.gov.ezproxy.aub.edu.lb/pubmed/20516702 | | | |
| 608 | Diagnostic molecular pathology : the American journal of surgical pathology, part B | Jadaon MM | 2010 | 0 | http://www.ncbi.nlm.nih.gov.ezproxy.aub.edu.lb/pubmed/?term=High+prevalence+of+activated+protein+C+resistance+and+factor+V+Leiden+mutation+in+an+Arab+population+and+patients+with+venous+thrombosis+in+Kuwait. | | | |
| 614 | Acta Cytologica | Kapila K | 2010 | 0 | http://www.ncbi.nlm.nih.gov.ezproxy.aub.edu.lb/pubmed/?term=Fine+needle+aspiration+cytology+of+the+thyroid+in+children+and+adolescents%3A+experience+with+792+aspirates. | | | |
| 643 | Annals of the Academy of Medicine, Singapore | Mahmoud F | 2010 | 0 | http://www.ncbi.nlm.nih.gov.ezproxy.aub.edu.lb/pubmed/?term=T+lymphocyte+activation+profiles+in+peripheral+blood+of+long-+versus+short-term+residents+of+Kuwait%3A+comparison+with+asthmatics. | | | |
| 654 | Acta Oncologica | Memon A | 2010 | 0 | http://www.ncbi.nlm.nih.gov.ezproxy.aub.edu.lb/pubmed/20397774 | | | |
| 659 | Saudi Medical Journal | Mittal R | 2010 | 0 | http://www.ncbi.nlm.nih.gov.ezproxy.aub.edu.lb//pubmed/20062903 | | | |
| 662 | Eastern Mediterranean health journal = La revue de santé de la Méditerranée orientale = al-Majallah | Mohammad HR | 2010 | 0 | http://www.ncbi.nlm.nih.gov.ezproxy.aub.edu.lb//pubmed/21218733 | | | |
| 663 | Journal of Clinical Hypertension | Mojiminiyi OA | 2010 | 0 | http://www.ncbi.nlm.nih.gov/pubmed/20433554 | | | |
| 669 | Acta Haematologica | Mustafa NY | 2010 | 0 | http://www.ncbi.nlm.nih.gov/pubmed/?term=Hypercoagulable+state+and+methylenetetrahydrofolate+reductase+(MTHFR)+C677T+mutation+in+patients+with+beta-thalassemia+major+in+Kuwait. | | | |
| 675 | Pediatric Dermatology | Nanda A | 2010 | 0 | http://www.ncbi.nlm.nih.gov.ezproxy.aub.edu.lb/pubmed/?term=Mycosis+fungoides+in+Arab+children+and+adolescents%3A+a+report+of+36+patients+from+Kuwait. | | | |
| 685 | Journal of obstetrics and gynaecology | Omu AE | 2010 | 0 | http://informahealthcare.com/doi/pdf/10.3109/01443610903443913 | | | |
| 863 | Medical Principles and Practice | Al-Bahar S | 2010 | 0 | http://www.ncbi.nlm.nih.gov/pubmed/20357498 | | | |
| 377 | Angiology | Al Rashdan I | 2010 | 0 | http://www.ncbi.nlm.nih.gov/pubmed/19398419 | | | |
| 742 | The British Journal of Radiology | Usmani S | 2010 | 0 | http://www.ncbi.nlm.nih.gov.ezproxy.aub.edu.lb/pubmed/?term=Scintimammography+in+conjunction+with+ultrasonography+for+local+breast+cancer+recurrence+in+post-mastectomy+breast | | | |
| 743 | Hellenic Journal of Nuclear Medicine | Usmani S | 2010 | 0 | http://www.ncbi.nlm.nih.gov.ezproxy.aub.edu.lb/pubmed/?term=Evaluation+of+the+gamma+probe+guided+sentinel+lymph+node+biopsy+and+the+blue+dye+technique+in+the+management+of+breast+cancer | | | |
| 175 | Diabetic Medicine | Mabry RM | 2010 | 0 | http://www.ncbi.nlm.nih.gov/pubmed/?term=Gender+differences+in+prevalence+of+the+metabolic+syndrome+in+Gulf+Cooperation+Council+Countries%3A+a+systematic+9 | | | |
| 523 | American Journal of Human Biology | Calistro Alvarado L | 2010 | 0 | http://www.ncbi.nlm.nih.gov/pubmed/20087895 | | | |
| 656 | Eastern Mediterranean health journal = La revue de santé de la Méditerranée orientale = al-Majallah | Mirmiran P | 2010 | 0 | http://www.ncbi.nlm.nih.gov.ezproxy.aub.edu.lb/pubmed/21218730 | | | |
| 673 | International Journal of Surgery | Najjar H | 2010 | 0 | http://www.ncbi.nlm.nih.gov.ezproxy.aub.edu.lb/pubmed/20601253 | | | |
| 680 | Neoplasma | Novotny L | 2010 | 0 | http://www.ncbi.nlm.nih.gov/pubmed/20568891 | | | |
| 738 | Journal of the Neurological Sciences | Tran J | 2010 | 0 | http://www.ncbi.nlm.nih.gov.ezproxy.aub.edu.lb/pubmed/20541222 | | | |
| 758 | Expert Review of Hematology | Zamecnikova A | 2010 | 0 | http://www.ncbi.nlm.nih.gov/pubmed/21082933 | | | |
| 14 | Pharmacology | Yousif MH | 2010 | 0 | http://www.ncbi.nlm.nih.gov/pubmed/?term=ole+of+20-hydroxyeicosatetraenoic+and+epoxyeicosatrienoic+acids+in+the+++++++regulation+of+vascular+function+in+a+model+of+hypertension+and+endothelial+++++++dysfunction. | | | |
| 18 | European Journal of Pharmacology | Dahaunsi GS | 2010 | 0 | http://www.ncbi.nlm.nih.gov/pubmed/?term=Angiotensin-(1-7)+prevents+diabetes-induced+attenuation+in+PPAR-gamma+and+++++++catalase+activities | | | |
| 19 | Kidney and Blood Pressure Research | Dahaunsi GS | 2010 | 0 | http://www.ncbi.nlm.nih.gov/pubmed/?term=FPTIII+mitigates+peroxisome-mediated+oxidative+stress+in+kidneys+of+spontaneously+++++++hypertensive+diabetic+rats | | | |
| 31 | Evidence Based Complementary and Alternative Medicine | Drobiova H | 2010 | 0 | http://www.ncbi.nlm.nih.gov/pubmed/?term=Garlic+increases+antioxidant+levels+in+diabetic+and+hypertensive+rats+determined++++++++by+a+modified+peroxidase+method | | | |
| 161 | Life Sciences | Bitar MS | 2010 | 0 | http://www.ncbi.nlm.nih.gov.ezproxy.aub.edu.lb/pubmed/?term=Inflammation+and+apoptosis+in+aortic+tissues+of+aged+type+II+diabetes%3A+amelioration+with+alpha-lipoic+acid+through+phosphatidylinositol+3-kinase%2FAkt-+dependent+mechanism | | | |
| 395 | Anticancer Research | Al-Bader M | 2010 | 0 | http://www.ncbi.nlm.nih.gov/pubmed/21036734 | | | |
| 562 | European Journal of Pharmacology | El-Hashim A | 2010 | 0 | http://www.ncbi.nlm.nih.gov/pubmed/20006602 | | | |
| 572 | Journal of Pharmacology and Experimental Theraputics | Ezeamuzie CI | 2010 | 0 | http://jpet.aspetjournals.org.ezproxy.aub.edu.lb/content/334/1/302.long | | | |
| 637 | Journal of Theoretical biology | Kumar mallik M | 2010 | 0 | http://www.ncbi.nlm.nih.gov.ezproxy.aub.edu.lb/pubmed/?term=A+hypothesis+and+theoretical+model+speculating+the+possible+role+of+therapy+mediated+neoplastic+cell+loss+in+promoting+the+process+of+glioblastoma+relapse. | | | |
| 666 | BMC Infectious Diseases | Mokaddas E | 2010 | 0 | http://www.ncbi.nlm.nih.gov/pubmed/?term=Levels+of+(1%C3%A2%E2%80%A0%E2%80%993)-%D8%AE%C2%B2-D-glucan%2C+Candida+mannan+and+Candida+DNA+in+serum+samples+of+pediatric+cancer+patients+colonized+with+Candida+species. | | | |
| 679 | Anticancer Research | Novotny L | 2010 | 0 | http://www.ncbi.nlm.nih.gov/pubmed/21187467 | | | |
| 695 | The Gulf Journal of Oncology | Ragheb AM | 2010 | 0 | http://www.ncbi.nlm.nih.gov/pubmed/?term=Focus+on+breast+and+prostate+cancer+prevention%2C+early+detection+and+new+trends+in+management+highlights+of+the+1st+National+Conference+on+Cancer+Control+in | | | |
| 487 | Int J Dermatol | Alsaleh QA | 2010 | 0 | http://www.ncbi.nlm.nih.gov/pubmed/21155090 | | | |
| 489 | American Journal of Hospice and Palliative Care | Alshemmari S | 2010 | 0 | http://www.ncbi.nlm.nih.gov/pubmed/20228359 | | | |
| 494 | Ann Hematol | Ameen R | 2010 | 0 | http://www.ncbi.nlm.nih.gov/pubmed/19711076 | | | |
| 503 | Medical Principles and Practice | Babusik P | 2010 | 0 | http://www.ncbi.nlm.nih.gov/pubmed/20516703 | | | |
| 693 | Annals of thoracic medicine | Panicker R | 2010 | 0 | http://www.thoracicmedicine.org/article.asp?issn=1817-1737;year=2010;volume=5;issue=1;spage=37;epage=42;aulast=Panicker | | | |
| 123 | Diabetes Care | Al Arouj M | 2010 | 0 | http://www.ncbi.nlm.nih.gov/pubmed/?term=Recommendations+for+management+of+diabetes+during+Ramadan%3A+update+2010 | | | |
| 15 | Global Health Promotion | Ramadan J | 2010 | 0 | http://www.ncbi.nlm.nih.gov/pubmed/?term=Developing+a+national+physical+activity+plan%3A+the+Kuwait+example | | | |
| 499 | The Gulf Journal of Oncology | Arora R | 2010 | 0 | http://www.ncbi.nlm.nih.gov/pubmed/20601337 | | | |
| 112 | Eastern Medditerranian Health Journal | Al Khalaf MM | 2010 | 0 | http://www.ncbi.nlm.nih.gov/pubmed/20799528 | | | |
| 408 | Annals of Thoracic Medicine | Al-Ghimlas F | 2010 | 0 | http://www.ncbi.nlm.nih.gov/pubmed/20981184 | | | |
| 522 | The Gulf Journal of Oncology | Bourisly MJ | 2010 | 0 | http://www.ncbi.nlm.nih.gov/pubmed/20601340 | | | |
| 555 | The Gulf Journal of Oncology | Elbasmi A | 2010 | 0 | http://www.ncbi.nlm.nih.gov/pubmed/20601333 | | | |
| 347 | The Gulf Journal of Oncology | Abu Zallouf S | 2010 | 0 | http://www.ncbi.nlm.nih.gov/pubmed/20601343 | | | |
| 407 | Respirology | Al-Ghimlas F | 2010 | 0 | http://www.ncbi.nlm.nih.gov/pubmed/20497386 | | | |
| 72 | JACC. Cardiovascular interventions | Azar R R | 2010 |  | Not Found | |  |  |
| 144 | European journal of pediatric surgery | Daher P | 2010 |  | Not Found | |  |  |
| 414 | Evidence-based nursing | Noureddine S | 2010 |  | Abstract Not Found | |  |  |
| 420 | Acta oncologica | Otrock Z K | 2010 |  | Abstract Not Found | |  |  |
| 952 | J Med Liban | Daher M | 2010 |  | http://www.ncbi.nlm.nih.gov/pubmed/20549890 | | | |
| 1 | Clinical and experimental dermatology | Abbas O | 2010 | 0 | Not Found | |  |  |
| 4 | Journal of cutaneous pathology | Abbas O | 2010 | 0 | http://www.ncbi.nlm.nih.gov/pubmed/?term=Perforating+cutaneous+metastasis+from+an+ovarian+adenocarcinoma | | | |
| 5 | The American surgeon | Abboud B | 2010 | 0 | http://www.ncbi.nlm.nih.gov/pubmed/?term=Are+papillary+microcarcinomas+of+the+thyroid+gland+revealed+by+cervical+adenopathy+more+aggressive%3F | | | |
| 8 | The breast journal | Abboud M | 2010 | 0 | http://www.ncbi.nlm.nih.gov/pubmed/?term=Complete+response+of+brain+metastases+from+breast+cancer+overexpressing+Her-2%2Fneu+to+radiation+and+concurrent+Lapatinib+and+Capecitabine | | | |
| 13 | Clinical cardiology | Abdallah M | 2010 | 0 | http://www.ncbi.nlm.nih.gov/pubmed/?term=Acute+coronary+syndromes%3A+clinical+characteristics%2C+management%2C+and+outcomes+at+the+American+University+of+Beirut+Medical+Center%2C+2002-2005 | | | |
| 37 | Radiation oncology | Aftimos P G | 2010 | 0 | http://www.ncbi.nlm.nih.gov/pubmed/?term=Adjuvant+chemo-radiation+for+gastric+adenocarcinoma%3A+an+institutional+experience | | | |
| 121 | Southern medical journal | Charafeddine K M | 2010 | 0 | http://www.ncbi.nlm.nih.gov/pubmed/20622728 | | | |
| 135 | J Med Liban | Chouairy C J | 2010 | 0 | http://www.ncbi.nlm.nih.gov/pubmed/20549899 | | | |
| 148 | Clinical cardiology | Dakik H A | 2010 | 0 | http://www.ncbi.nlm.nih.gov/pubmed/20641110 | | | |
| 185 | Pediatric neurology | El-Bitar M K | 2010 | 0 | http://www.ncbi.nlm.nih.gov/pubmed/?term=Severe+cerebral+vaso-occlusive+disease+in+macrophage+activation+syndrome | | | |
| 209 | World journal of surgical oncology | Faraj W | 2010 | 0 | http://www.ncbi.nlm.nih.gov/pubmed/?term=Primary+undifferentiated+embryonal+sarcoma+of+the+liver+mistaken+for+hydatid+disease | | | |
| 257 | Leukemia research | Ghosn M G | 2010 | 0 | http://www.ncbi.nlm.nih.gov/pubmed/19674788 | | | |
| 353 | Clinical and experimental dermatology | Malek J | 2010 | 0 | http://www.ncbi.nlm.nih.gov/pubmed/?term=Multiple+nodules+and+plaques+over+the+abdomen+malek+2010 | | | |
| 357 | International journal of hematology | Masroujeh R | 2010 | 0 | http://www.ncbi.nlm.nih.gov/pubmed/?term=Myasthenia+gravis+developing+in+a+patient+with+CNS+lymphoma | | | |
| 359 | Thorax | Mazboudi O | 2010 | 0 | http://www.ncbi.nlm.nih.gov/pubmed/?term=Endobronchial+metastasis+of+osteosarcoma+presenting+as+position-dependent+and+variable+airway+obstruction+of+the+trachea+mazboudi | | | |
| 467 | European journal of gastroenterology & hepatology | Saliba T | 2010 | 0 | http://www.ncbi.nlm.nih.gov/pubmed/?term=Hepatocellular+carcinoma+in+two+patients+with+cardiac+cirrhosis+saliba+2010 | | | |
| 564 | J Thromb Thrombolysis | Aoun EG | 2010 | 0 | http://www.ncbi.nlm.nih.gov/pubmed/20174855 | | | |
| 587 | Cancer genetics and cytogenetics | Charafeddine KM | 2010 | 0 | http://www.ncbi.nlm.nih.gov/pubmed/20513531 | | | |
| 1004 | Europace | Kossaify A | 2010 | 0 | http://www.ncbi.nlm.nih.gov/pubmed/20639208 | | | |
| 34 | Womens Health Issues | Afifi R A | 2010 | 0 | http://www.ncbi.nlm.nih.gov/pubmed/?term=Social+capital%2C+women's+autonomy+and+smoking+among+married+women+in+low-income+urban+neighborhoods+of+Beirut%2C+Lebanon | | | |
| 35 | European journal of public health | Afifi R A | 2010 | 0 | http://www.ncbi.nlm.nih.gov/pubmed/?term=Neighbourhood+influences+on+narghile+smoking+among+youth+in+Beirut | | | |
| 79 | J Med Liban | Bahous J | 2010 | 1 | http://www.ncbi.nlm.nih.gov/pubmed/21409942 | | | |
| 225 | International journal of mental health nursing | Farhood L F | 2010 | 0 | http://www.ncbi.nlm.nih.gov/pubmed/?term=Detainment+and+health%3A+the+case+of+the+Lebanese+hostages+of+war | | | |
| 228 | Pediatrics international | Fazah A | 2010 | 0 | http://www.ncbi.nlm.nih.gov/pubmed/?term=Activity%2C+inactivity+and+quality+of+life+among+Lebanese+adolescents | | | |
| 295 | Clinical and experimental obstetrics & gynecology | Isma'eel H | 2010 | 0 | http://www.ncbi.nlm.nih.gov/pubmed/?term=Diabetes+supersedes+dobutamine+stress+echocardiography+in+predicting+cardiac+events+in+female+patients | | | |
| 298 | International journal of cardiology | Itani S | 2010 | 0 | http://www.ncbi.nlm.nih.gov/pubmed/?term=The+interaction+of+gender+and+clinical+risk+profile+in+patients+referred+for+exercise+nuclear+imaging+versus+exercise+echocardiography | | | |
| 317 | Public Health Nutrition | Kanaan M N | 2010 | 0 | http://www.ncbi.nlm.nih.gov/pubmed/?term=Gender+differences+in+determinants+of+weight-control+behaviours+among+adolescents+in+Beirut | | | |
| 342 | Cancer epidemiology | Lakkis N A | 2010 | 0 | http://www.ncbi.nlm.nih.gov/pubmed/?term=Breast+cancer+in+Lebanon%3A+incidence+and+comparison+to+regional+and+Western+countries | | | |
| 368 | Medical oncology | Musallam K M | 2010 | 0 | http://www.ncbi.nlm.nih.gov/pubmed/?term=Primary+colorectal+lymphoma+musallam | | | |
| 387 | Tobacco control | Nakkash R | 2010 | 0 | http://www.ncbi.nlm.nih.gov/pubmed/?term=Health+warning+labelling+practices+on+narghile+(shisha%2C+hookah)+waterpipe+tobacco+products+and+related+accessories | | | |
| 399 | Annals of nutrition & metabolism | Nasreddine L | 2010 | 0 | http://www.ncbi.nlm.nih.gov/pubmed/?term=Metabolic+syndrome+and+insulin+resistance+in+obese+prepubertal+children+in+Lebanon%3A+a+primary+health+concern | | | |
| 404 | Clinical chemistry and laboratory medicine | Nemr R | 2010 | 0 | http://www.ncbi.nlm.nih.gov/pubmed/?term=Differential+contribution+of+MTHFR+C677T+variant+to+the+risk+of+diabetic+nephropathy+in+Lebanese+and+Bahraini+Arabs+20120 | | | |
| 417 | International journal of nursing studies | Noureddine S | 2010 | 0 | http://www.ncbi.nlm.nih.gov/pubmed/?term=Response+to+a+cardiac+event+in+relation+to+cardiac+knowledge+and+risk+perception+in+a+Lebanese+sample%3A+a+cross+sectional+survey | | | |
| 451 | Pediatric blood & cancer | Saghieh S | 2010 | 0 | http://www.ncbi.nlm.nih.gov/pubmed/?term=Seven-year+experience+of+using+Repiphysis+expandable+prosthesis+in+children+with+bone+tumors | | | |
| 452 | The Iowa orthopaedic journal | Saghieh S | 2010 | 0 | http://www.ncbi.nlm.nih.gov/pubmed/?term=The+risk+of+local+recurrence+along+the+core-needle+biopsy+tract+in+patients+with+bone+sarcomas | | | |
| 486 | World journal of surgical oncology | Shamseddine A | 2010 | 0 | http://www.ncbi.nlm.nih.gov/pubmed/?term=Unusually+young+age+distribution+of+primary+hepatic+leiomyosarcoma%3A+case+series+and+review+of+the+adult+literature | | | |
| 510 | Acta diabetologica | Taleb N | 2010 | 0 | http://www.ncbi.nlm.nih.gov/pubmed/?term=Prevalence+of+asthmatic+symptoms+in+Lebanese+patients+with+type+1+diabetes+and+their+unaffected+siblings+compared+to+age-matched+controls | | | |
| 536 | Atherosclerosis | Youhanna S | 2010 | 0 | http://www.ncbi.nlm.nih.gov/pubmed/?term=Parental+consanguinity+and+family+history+of+coronary+artery+disease+strongly+predict+early+stenosis | | | |
| 543 | Genetic testing and molecular biomarkers | Zgheib N K | 2010 | 0 | http://www.ncbi.nlm.nih.gov/pubmed/?term=Cytochrome+P4502E1+(CYP2E1)+genetic+polymorphisms+in+a+Lebanese+population%3A+frequency+distribution+and+association+with+morbid+diseases | | | |
| 669 | Annals of Thoracic Medicine | Nahas R | 2010 | 1 | http://www.ncbi.nlm.nih.gov.ezproxy.aub.edu.lb/pubmed/22347345 | | | |
| 982 | Indian J Surg Oncol | Hatoum HA | 2010 | 0 | http://www.ncbi.nlm.nih.gov/pubmed/22695879 | | | |
| 73 | The American journal of cardiology | Azar R R | 2010 | 0 | http://www.ncbi.nlm.nih.gov/pubmed/?term=Effect+of+ezetimibe%2Fatorvastatin+combination+on+oxidized+low+density+lipoprotein+cholesterol+in+patients+with+coronary+artery+disease+or+coronary+artery+disease+equivalent+Azar+2010 | | | |
| 74 | Clinical cardiology | Azar R R | 2010 | 0 | http://www.ncbi.nlm.nih.gov/pubmed/?term=Effect+of+high+bolus+dose+tirofiban+on+the+inflammatory+response+following+percutaneous+coronary+intervention | | | |
| 170 | Annals of nutrition & metabolism | El Khoury D | 2010 | 0 | http://www.ncbi.nlm.nih.gov/pubmed/?term=Metabolic+and+appetite+hormone+responses+of+hyperinsulinemic+normoglycemic+males+to+meals+with+varied+macronutrient+compositions | | | |
| 217 | Medical oncology | Farhat F S | 2010 | 0 | http://www.ncbi.nlm.nih.gov/pubmed/?term=Role+of+low+dose+capecitabine+combined+to+irinotecan+in+advanced+and+metastatic+gastric+cancer | | | |
| 242 | Radiation oncology | Geara F B | 2010 | 0 | http://www.ncbi.nlm.nih.gov/pubmed/20863389 | | | |
| 92 | Journal of clinical oncology | Bazarbachi A | 2010 | 0 | http://www.ncbi.nlm.nih.gov/pubmed/?term=Meta-analysis+on+the+use+of+zidovudine+and+interferon-alfa+in+adult+T-cell+leukemia%2Flymphoma+showing+improved+survival+in+the+leukemic+subtypes | | | |
| 218 | Expert review of anticancer therapy | Farhat F S | 2010 | 0 | http://www.ncbi.nlm.nih.gov/pubmed/20397919 | | | |
| 243 | J Med Liban | Ghabril R | 2010 | 0 | http://www.ncbi.nlm.nih.gov/pubmed/?term=Renovascular+hypertension+in+children+Ghabril+2010 | | | |
| 246 | Drug discovery today | Ghantous A | 2010 | 0 | http://www.ncbi.nlm.nih.gov/pubmed/?term=What+made+sesquiterpene+lactones+reach+cancer+clinical+trials%3F | | | |
| 322 | Diabetes Research and Clinical Practice | Karnib H H | 2010 | 0 | http://www.ncbi.nlm.nih.gov/pubmed/?term=The+cardiorenal+syndrome+in+diabetes+mellitus+karnib+2010 | | | |
| 349 | J Med Liban | Majdalani M N | 2010 | 0 | http://www.ncbi.nlm.nih.gov/pubmed/?term=Management+of+hypertensive+emergencies+in+children+majdalani+2010 | | | |
| 385 | International journal of surgery | Najjar H | 2010 | 0 | http://www.ncbi.nlm.nih.gov/pubmed/?term=Age+at+diagnosis+of+breast+cancer+in+Arab+nations+najjar+2010 | | | |
| 437 | TheScientificWorldJournal | Saab R | 2010 | 0 | http://www.ncbi.nlm.nih.gov/pubmed/?term=Cellular+senescence%3A+many+roads%2C+one+final+destination | | | |
| 466 | Womens Health Issues | Saliba M | 2010 | 0 | http://www.ncbi.nlm.nih.gov/pubmed/?term=Expanding+concern+for+women's+health+in+developing+countries%3A+the+case+of+the+Eastern+Mediterranean+Region | | | |
| 469 | Asian Pacific journal of cancer prevention | Salim E I | 2010 | 0 | http://www.ncbi.nlm.nih.gov/pubmed/?term=Cancer+epidemiology+in+South-West+Asia+-+past%2C+present+and+future+salim+2010 | | | |
| 476 | J Med Liban | Sanjad S A | 2010 | 0 | http://www.ncbi.nlm.nih.gov/pubmed/?term=Etiology+of+hypertension+in+children+and+adolescents+sanjad+2010 | | | |
| 516 | Journal of oncology | Tfayli A | 2010 | 0 | http://www.ncbi.nlm.nih.gov/pubmed/?term=Breast+cancer+in+low-+and+middle-income+countries%3A+an+emerging+and+challenging+epidemic | | | |
| 547 | Breast Cancer Res Treat | Zreik T G | 2010 | 0 | http://www.ncbi.nlm.nih.gov/pubmed/?term=zreik+2010+Fertility+drugs+and+the+risk+of+breast+cancer%3A+a+meta-analysis+and+review | | | |
| 592 | Asian Pacific journal of cancer prevention | Daher M | 2010 | 0 | http://www.ncbi.nlm.nih.gov/pubmed/20590358 | | | |
| 11 | Coronary artery disease | Abchee A | 2010 | 0 | http://www.ncbi.nlm.nih.gov/pubmed/?term=The+I+allele+of+the+angiotensin+converting+enzyme+I%2FD+polymorphism+confers+protection+against+coronary+artery+disease | | | |
| 15 | Leukemia research | Abdel-Massih R M | 2010 | 0 | http://www.ncbi.nlm.nih.gov/pubmed/?term=The+apoptotic+and+anti-proliferative+activity+of+Origanum+majorana+extracts+on+human+leukemic+cell+line | | | |
| 88 | Journal of the National Comprehensive Cancer Network | Bazarbachi A | 2010 | 0 | http://www.ncbi.nlm.nih.gov/pubmed/?term=Modification+and+implementation+of+NCCN+guidelines+on+lymphomas+in+the+Middle+East+and+North+Africa+region | | | |
| 189 | Radiation oncology | El-Khatib M | 2010 | 0 | http://www.ncbi.nlm.nih.gov/pubmed/?term=Cell+death+by+the+quinoxaline+dioxide+DCQ+in+human+colon+cancer+cells+is+enhanced+under+hypoxia+and+is+independent+of+p53+and+p21 | | | |
| 190 | Apoptosis | El-Najjar N | 2010 | 0 | http://www.ncbi.nlm.nih.gov/pubmed/?term=Reactive+oxygen+species+mediate+thymoquinone-induced+apoptosis+and+activate+ERK+and+JNK+signaling | | | |
| 292 | Obesity reviews | Hwalla N | 2010 | 0 | http://www.ncbi.nlm.nih.gov/pubmed/?term=Matter+related+to+the+research+article+published+in+Obesity+Reviews+(2009%3B+9%3A+389-399)%2C+entitled+'Obesity+in+Mediterranean+region+(1997-2007)%3A+a+systematic+review' | | | |
| 318 | J Med Liban | Kanj N | 2010 | 1 | http://www.ncbi.nlm.nih.gov/pubmed/?term=%5BTobacco%3A+its+control+is+urgently+needed%5D+kanj+2010 | | | |
| 328 | Inhalation toxicology | Katurji M | 2010 | 0 | http://www.ncbi.nlm.nih.gov/pubmed/?term=Direct+measurement+of+toxicants+inhaled+by+water+pipe+users+in+the+natural+environment+using+a+real-time+in+situ+sampling+technique | | | |
| 332 | Tobacco control | Khalil J | 2010 | 0 | http://www.ncbi.nlm.nih.gov/pubmed/?term=Lebanon%3A+diet+hookah+khalil+2010 | | | |
| 391 | Asia-Pacific journal of public health | Nakkash R T | 2010 | 0 | http://www.ncbi.nlm.nih.gov/pubmed/?term=Building+research+evidence+for+policy+advocacy%3A+a+qualitative+evaluation+of+existing+smoke-free+policies+in+Lebanon | | | |
| 444 | Tobacco control | Saade G | 2010 | 0 | http://www.ncbi.nlm.nih.gov/pubmed/?term=indoor+secondhand+tobacco+smoke+emission+levels+in+six+Lebanese+cities | | | |
| 483 | Food and chemical toxicology | Sepetdjian E | 2010 | 0 | http://www.ncbi.nlm.nih.gov/pubmed/?term=sepetdjian+Carcinogenic+PAH+in+waterpipe+charcoal+products | | | |
| 593 | Atmos Environ (1994) | Daher N | 2010 | 0 | http://www.ncbi.nlm.nih.gov/pubmed/20161525 | | | |
| 595 | Canadian Journal of Physiology and Pharmacology | Der-Boghossian AH | 2010 | 0 | http://www.ncbi.nlm.nih.gov/pubmed/20651823 | | | |
| 625 | Canadian Journal of Physiology and Pharmacology | Hantouche CM | 2010 | 0 | http://www.ncbi.nlm.nih.gov/pubmed/20130739 | | | |
| 50 | Journal of community genetics | Alwan A | 2010 | 0 | http://www.ncbi.nlm.nih.gov/pubmed/?term=ALOX5AP+gene+variants+show+differential+association+with+coronary+artery+disease+in+different+populations | | | |
| 361 | Annals of nutrition & metabolism | Mehio Sibai A | 2010 | 0 | http://www.ncbi.nlm.nih.gov/pubmed/?term=Nutrition+transition+and+cardiovascular+disease+risk+factors+in+Middle+East+and+North+Africa+countries%3A+reviewing+the+evidence | | | |
| 59 | Journal of interprofessional care | Arevian M | 2010 | 0 | http://www.ncbi.nlm.nih.gov/pubmed/?term=Training+trainees%2C+young+activists%2C+to+conduct+awareness+campaigns+about+prevention+of+substance+abuse+among+Lebanese%2FArmenian+young+people | | | |
| 22 | International surgery | Abou-Jaoude M M | 2010 | 0 | http://www.ncbi.nlm.nih.gov/pubmed/21309411 | | | |
| 227 | J Med Liban | Fazah A | 2010 | 0 | http://www.ncbi.nlm.nih.gov/pubmed/20549896 | | | |
| 267 | J Med Liban | Hage C N | 2010 | 0 | http://www.ncbi.nlm.nih.gov/pubmed/?term=Health+habits+and+vaccination+status+of+Lebanese+residents%3A+are+future+doctors+applying+the+rules+of+prevention%3F%5D | | | |
| 321 | J Med Liban | Karam E G | 2010 | 0 | http://www.ncbi.nlm.nih.gov/pubmed/?term=A+rapid+situation+assessment+(RSA)+study+of+alcohol+and+drug+use+in+Lebanon | | | |
| 43 | Genetic testing and molecular biomarkers | Akra-Ismail M | 2010 | 0 | http://www.ncbi.nlm.nih.gov/pubmed/?term=Association+between+angiotensin-converting+enzyme+insertion%2Fdeletion+genetic+polymorphism+and+hypertension+in+a+sample+of+Lebanese+patients | | | |
| 163 | European journal of oncology nursing | Doumit M A | 2010 | 0 | http://www.ncbi.nlm.nih.gov/pubmed/19815459 | | | |
| 164 | Cancer nursing | Doumit M A | 2010 | 0 | http://www.ncbi.nlm.nih.gov/pubmed/20142735 | | | |
| 693 | Journal de Radiologie | Boufettal H | 2010 | 1 | http://www.ncbi.nlm.nih.gov/pubmed/21242917 | | | |
| 694 | Journal de Radiologie | Boufettal H | 2010 | 1 | http://www.ncbi.nlm.nih.gov/pubmed/21178881 | | | |
| 874 | Annales de Dermatologie et de Vénéréologie | Chiheb S | 2010 |  | http://www.ncbi.nlm.nih.gov/pubmed/20171446 | | | |
| 4 | World journal of Surgical Oncology | Abahssain H | 2010 | 0 | http://www.ncbi.nlm.nih.gov/pubmed/20704728 | | | |
| 154 | Orthopaedics & Traumatology, Surgery & Research | Chahdi H | 2010 | 0 | http://www.ncbi.nlm.nih.gov/pubmed/20580629 | | | |
| 202 | Chirurgie de la main | El bouanani A | 2010 | 1 | http://www.ncbi.nlm.nih.gov/pubmed/20724204 | | | |
| 218 | World journal of Surgical Oncology | El Mesbahi O | 2010 | 0 | http://www.ncbi.nlm.nih.gov/pubmed/20819211 | | | |
| 288 | Korean Journal of ophthalmology | Hassane S | 2010 | 0 | http://www.ncbi.nlm.nih.gov/pubmed/21165236 | | | |
| 292 | B-ENT | Herrag M | 2010 | 0 | http://www.ncbi.nlm.nih.gov/pubmed/21090166 | | | |
| 352 | Orthopaedics & Traumatology, Surgery & Research | Lahrach K | 2010 | 0 | http://www.ncbi.nlm.nih.gov/pubmed/21056026 | | | |
| 381 | The Pan African Medical Journal | Hassani K I M | 2010 | 0 | http://www.ncbi.nlm.nih.gov/pubmed/21119996 | | | |
| 405 | Can J Neurol Sci | Mostarchid B E | 2010 | 0 | http://www.ncbi.nlm.nih.gov/pubmed/20724262 | | | |
| 411 | Tex Heart Inst J | Moutaouekkil E M | 2010 | 0 | http://www.ncbi.nlm.nih.gov/pubmed/20401297 | | | |
| 424 | The Pan African Medical Journal | Najoua Z | 2010 | 1 | http://www.ncbi.nlm.nih.gov/pubmed/21918693 | | | |
| 441 | J Emerg Trauma Shock | Rabbani K | 2010 | 0 | http://www.ncbi.nlm.nih.gov/pubmed/20931002 | | | |
| 447 | Cancer Radiothérapie | Rais H | 2010 | 1 | http://www.ncbi.nlm.nih.gov/pubmed/20189431 | | | |
| 472 | The Pan African Medical Journal | Serraj M | 2010 | 1 | http://www.ncbi.nlm.nih.gov/pubmed/21436950 | | | |
| 479 | Indian Journal of Pathology and Microbiology | Soufiane M | 2010 | 0 | http://www.ncbi.nlm.nih.gov/pubmed/20699519 | | | |
| 501 | World journal of Surgical Oncology | Tazi E | 2010 | 0 | http://www.ncbi.nlm.nih.gov/pubmed/21073751 | | | |
| 713 | Journal of Cancer Research and Therapeutics | Errihani H | 2010 | 0 | http://www.ncbi.nlm.nih.gov/pubmed/21119292 | | | |
| 716 | Neurology India | Hamzi K | 2010 | 0 | http://www.ncbi.nlm.nih.gov/pubmed/20228473 | | | |
| 861 | Annales de Dermatologie et de Vénéréologie | Gallouj S | 2010 | 1 | http://www.ncbi.nlm.nih.gov/pubmed/21134592 | | | |
| 862 | The Pan African Medical Journal | Rachid B | 2010 | 1 | http://www.ncbi.nlm.nih.gov/pubmed/21120001 | | | |
| 863 | Revue des maladies respiratoires | Achir A | 2010 | 1 | http://www.ncbi.nlm.nih.gov/pubmed/21111285 | | | |
| 864 | Annales de Pathologie | Harchichi N | 2010 | 1 | http://www.ncbi.nlm.nih.gov/pubmed/21055530 | | | |
| 866 | Néphrologie & thérapeutique | Zbiti N | 2010 | 1 | http://www.ncbi.nlm.nih.gov/pubmed/20920898 | | | |
| 867 | Revue de stomatologie et de chirurgie maxillo-faciale | Kerrary S | 2010 | 1 | http://www.ncbi.nlm.nih.gov/pubmed/20817202 | | | |
| 868 | Presse médicale | Darouassi Y | 2010 | 1 | http://www.ncbi.nlm.nih.gov/pubmed/20620016 | | | |
| 869 | Archives de pédiatrie | Charhi H | 2010 | 1 | http://www.ncbi.nlm.nih.gov/pubmed/20570497 | | | |
| 871 | La Revue de médecine interne | Chekrine T | 2010 | 1 | http://www.ncbi.nlm.nih.gov/pubmed/20554090 | | | |
| 873 | Cancer radiothérapie | Tawfiq N | 2010 | 1 | http://www.ncbi.nlm.nih.gov/pubmed/20434933 | | | |
| 876 | Revue de laryngologie - otologie - rhinologie | Essaadi M | 2010 | 1 | http://www.ncbi.nlm.nih.gov/pubmed/21866745 | | | |
| 877 | Le Mali médical | Berrada N | 2010 | 1 | http://www.ncbi.nlm.nih.gov/pubmed/21470955 | | | |
| 878 | Journal des maladies vasculaires | Azghari A | 2010 | 1 | http://www.ncbi.nlm.nih.gov/pubmed/19962259 | | | |
| 880 | Chirurgie de la main | Biyi A | 2010 | 1 | http://www.ncbi.nlm.nih.gov/pubmed/19811940 | | | |
| 896 | Annales de Cardiologie et d'Angéiologie | Tamdy A | 2010 | 1 | http://www.ncbi.nlm.nih.gov/pubmed/18980753 | | | |
| 5 | BMC Research Notes | Abahssain H | 2010 | 0 | http://www.ncbi.nlm.nih.gov/pubmed/21059204 | | | |
| 42 | J Infect Dev Ctries | Alhamany Z | 2010 | 0 | http://www.ncbi.nlm.nih.gov/pubmed/21252451 | | | |
| 72 | Transfus Clin Biol | Bellaoui N | 2010 | 1 | http://www.ncbi.nlm.nih.gov/pubmed/20674439 | | | |
| 137 | Cancer Radiothérapie | Boufettal H | 2010 | 1 | http://www.ncbi.nlm.nih.gov/pubmed/20674443 | | | |
| 226 | Revue de pneumologie clinique | El Ouazzani H | 2010 | 1 | http://www.ncbi.nlm.nih.gov/pubmed/21167440 | | | |
| 234 | Eastern Mediterranean health journal | El-Awa F | 2010 | 0 | http://www.ncbi.nlm.nih.gov/pubmed/20795439 | | | |
| 246 | Free radical research | Ezzikouri S | 2010 | 0 | http://www.ncbi.nlm.nih.gov/pubmed/19929244 | | | |
| 272 | Saudi Journal of Kidney Disease and Transplantation | Haddiya I | 2010 | 0 | http://www.ncbi.nlm.nih.gov/pubmed/20427893 | | | |
| 295 | Pediatr Blood Cancer | Hessissen L | 2010 | 0 | http://www.ncbi.nlm.nih.gov/pubmed/19746454 | | | |
| 313 | BMC Research Notes | Ismaili N | 2010 | 0 | http://www.ncbi.nlm.nih.gov/pubmed/20920323 | | | |
| 329 | Annales de Biologie Clinique | Kabbaj M | 2010 | 0 | http://www.ncbi.nlm.nih.gov/pubmed/20478772 | | | |
| 334 | Child's nervous system | Karkouri M | 2010 | 0 | http://www.ncbi.nlm.nih.gov/pubmed/20179946 | | | |
| 353 | Neurochirurgie | Lakhdar F | 2010 | 0 | http://www.ncbi.nlm.nih.gov/pubmed/20615516 | | | |
| 397 | J Eur Acad Dermatol Venereol | Meziane M | 2010 | 0 | http://www.ncbi.nlm.nih.gov/pubmed/19207652 | | | |
| 435 | The Pan African Medical Journal | Ouadnouni Y | 2010 | 1 | http://www.ncbi.nlm.nih.gov/pubmed/21918708 | | | |
| 468 | Journal of hematology & oncology | Sbitti Y | 2010 | 0 | http://www.ncbi.nlm.nih.gov/pubmed/20569496 | | | |
| 484 | American Journal of Health Promotion | Tachfouti N | 2010 | 0 | http://www.ncbi.nlm.nih.gov/pubmed/20465147 | | | |
| 515 | Annals of Human Biology | They-They TP | 2010 | 0 | http://www.ncbi.nlm.nih.gov/pubmed/20687780 | | | |
| 245 | Bulletin du cancer | Errihani H | 2010 | 1 | http://www.ncbi.nlm.nih.gov/pubmed/20385518 | | | |
| 75 | Curr Diabetes Rev | Benalla W | 2010 | 0 | http://www.ncbi.nlm.nih.gov/pubmed/20522017 | | | |
| 149 | Cost Effectiveness and Resource Allocation | Boutayeb S | 2010 | 1 | http://www.ncbi.nlm.nih.gov/pubmed/20828417 | | | |
| 404 | J Pharm Pharmacol | Monfalouti H E | 2010 | 0 | http://www.ncbi.nlm.nih.gov/pubmed/21054392 | | | |
| 423 | International Journal of Surgery | Najjar H | 2010 | 0 | http://www.ncbi.nlm.nih.gov/pubmed/20601253 | | | |
| 494 | Indian Journal of Palliative Care | Tazzi E | 2010 | 0 | http://www.ncbi.nlm.nih.gov/pubmed/21218002 | | | |
| 676 | La Presse Médicale | Abahssain H | 2010 | 1 | http://www.ncbi.nlm.nih.gov/pubmed/21074352 | | | |
| 872 | Presse médicale | Allam W | 2010 | 1 | http://www.ncbi.nlm.nih.gov/pubmed/20537855 | | | |
| 875 | Bulletin du Cancer | Mellas N | 2010 | 1 | http://www.ncbi.nlm.nih.gov/pubmed/20051349 | | | |
| 207 | Cellular and molecular biology | El Hamdani W | 2010 | 0 | http://www.ncbi.nlm.nih.gov/pubmed/20937225 | | | |
| 215 | Medical oncology | El khair MM | 2010 | 0 | http://www.ncbi.nlm.nih.gov/pubmed/19771536 | | | |
| 259 | Pak J Pharm Sci | Gaamoussi F | 2010 | 0 | http://www.ncbi.nlm.nih.gov/pubmed/20363702 | | | |
| 306 | The Pan African Medical Journal | Hsaini Y | 2010 | 0 | http://www.ncbi.nlm.nih.gov/pubmed/21293744 | | | |
| 422 | Cellular and molecular biology | Naji F | 2010 | 0 | http://www.ncbi.nlm.nih.gov/pubmed/21215239 | | | |
| 269 | Cardiovascular Journal of Africa | Habbal R | 2010 | 0 | http://www.ncbi.nlm.nih.gov/pubmed/20838718 | | | |
| 426 | Journal of Pain and Symptom Management | Nejmi M | 2010 | 0 | http://www.ncbi.nlm.nih.gov/pubmed/20619213 | | | |
| 524 | European Journal of Cancer | Zanetti R | 2010 | 0 | http://www.ncbi.nlm.nih.gov/pubmed/20031391 | | | |
| 47 | Cancer Res | Ameziane-El-Hassani R | 2010 | 0 | http://www.ncbi.nlm.nih.gov/pubmed/20424115 | | | |
| 123 | Eastern Mediterranean Health Journal | Berraho M | 2010 | 0 | http://www.ncbi.nlm.nih.gov/pubmed/20799598 | | | |
| 131 | Rev Epidemiol Sante Publique | Bouaiti E | 2010 | 0 | http://www.ncbi.nlm.nih.gov/pubmed/20080370 | | | |
| 368 | Rev Neurol (Paris) | Lotfi S | 2010 | 1 | http://www.ncbi.nlm.nih.gov/pubmed/20299068 | | | |
| 461 | Asian Pacific journal of cancer prevention | Salim EI | 2010 | 0 | http://www.ncbi.nlm.nih.gov/pubmed/20553067 | | | |
| 475 | Epidemiologia & Prevenzione | Sobrato I | 2010 | 1 | http://www.ncbi.nlm.nih.gov/pubmed/20595732 | | | |
| 103 | International Journal of Biological Markers | Bennani B | 2010 | 1 | http://www.ncbi.nlm.nih.gov/pubmed/21161938 | | | |
| 129 | Human & Experimental Toxicology | Bnouham M | 2010 | 0 | http://www.ncbi.nlm.nih.gov/pubmed/20154101 | | | |
| 156 | Pharm Biol | Chaouki W | 2010 | 0 | http://www.ncbi.nlm.nih.gov/pubmed/20645812 | | | |
| 28 | Medical principles and practice | Zaharna MM | 2010 | 0 | http://www.ncbi.nlm.nih.gov/pubmed/20881413 | | | |
| 29 | Eastern Mediterranean health journal = La revue de sante de la Medoterranee orientale = al-Majallah | Mousa HS | 2010 | 0 | http://www.ncbi.nlm.nih.gov/pubmed/20799587 | | | |
| 32 | Saudi journal of kidney diseases and transplantation: an official publication of the Saudi Center fo | Sweileh WM | 2010 | 0 | http://www.ncbi.nlm.nih.gov/pubmed/20587868 | | | |
| 35 | Acta Diabetol | Ereqat S | 2010 | 0 | http://www.ncbi.nlm.nih.gov/pubmed/19885641 | | | |
| 78 | Archives of environmental & occupational health | Abu Sham'a | 2010 | 0 | http://www.ncbi.nlm.nih.gov.ezproxy.aub.edu.lb/pubmed/21186424 | | | |
| 127 | Cancer | Azaiza F | 2010 | 0 | http://www.ncbi.nlm.nih.gov/pubmed/20589933 | | | |
| 139 | Tobacco control | Nakkash R | 2010 | 0 | http://www.ncbi.nlm.nih.gov.ezproxy.aub.edu.lb/////pubmed/20501497 | | | |
| 141 | Asian Pacific journal of cancer prevention | Qasem H | 2010 | 0 | http://www.ncbi.nlm.nih.gov.ezproxy.aub.edu.lb//pubmed/20590363 | | | |
| 40 | Int J Eat Disord | Al Sabbah H | 2010 | 0 | http://www.ncbi.nlm.nih.gov/pubmed/19437462 | | | |
| 104 | Public health nutrition | Mikki N | 2010 | 0 | http://www.ncbi.nlm.nih.gov.ezproxy.aub.edu.lb/pubmed/20441660 | | | |
| 30 | Eastern Mediterranean health journal = La revue de sante de la Medoterranee orientale = al-Majallah | Ghazal Musmar S | 2010 | 0 | http://www.ncbi.nlm.nih.gov/pubmed/20795421 | | | |
| 100 | World journal of surgical oncology | H Salim OE | 2010 | 0 | http://www.ncbi.nlm.nih.gov/pubmed/?term=Colorectal+carcinoma+associated+with+schistosomiasis%3A+a+possible+causal+relationship | | | |
| 17 | Indian journal of cancer | Ahmed H G | 2010 | 0 | https://www.ncbi.nlm.nih.gov.ezproxy.aub.edu.lb/pubmed/?term=Frequency+of+breast+cancer+among+Sudanese+patients+with+breast+palpable+lumps | | | |
| 19 | Diagnostic cytopathology | Ahmed H G | 2010 | 0 | http://www.ncbi.nlm.nih.gov/pubmed/?term=Oral+epithelial+atypical+changes+in+apparently+healthy+oral+mucosa+exposed+to+smoking%2C+alcohol%2C+peppers+and+hot+meals%2C+using+the+AgNOR+and+Papanicolaou+staining+techniques | | | |
| 57 | Eastern Mediterranean health journal | El-Awa F | 2010 | 0 | https://www.ncbi.nlm.nih.gov.ezproxy.aub.edu.lb/pubmed/?term=Changes+in+tobacco+use+among+13-15-year-olds+between+1999+and+2007%3A+findings+from+the+Eastern+Mediterranean+Region | | | |
| 61 | Journal of foot and ankle surgery | ElMakki Ahmed M | 2010 | 0 | http://www.ncbi.nlm.nih.gov/pubmed/20123279 | | | |
| 65 | Experimental and clinical endocrinology & diabetes | Elrayah-Eliadarous H | 2010 | 0 | http://www.ncbi.nlm.nih.gov/pubmed/?term=Direct+costs+for+care+and+glycaemic+control+in+patients+with+type+2+diabetes+in+Sudan | | | |
| 79 | Journal of oral pathology and medicine | Jalouli J | 2010 | 0 | https://www.ncbi.nlm.nih.gov.ezproxy.aub.edu.lb/pubmed/?term=Presence+of+human+papilloma+virus%2C+herpes+simplex+virus+and+Epstein-Barr+virus+DNA+in+oral+biopsies+from+Sudanese+patients+with+regard+to+toombak+use | | | |
| 106 | Journal of cancer research and therapeutics | Osman T A | 2010 | 0 | http://www.ncbi.nlm.nih.gov/pubmed/?term=Pattern+of+malignant+tumors+registered+at+a+referral+oral+and+maxillofacial+hospital+in+Sudan+during+2006+and+2007 | | | |
| 109 | Infectious agents and cancer | Salih M M | 2010 | 0 | http://www.ncbi.nlm.nih.gov/pubmed/?term=Genotypes+of+human+papilloma+virus+in+Sudanese+women+with+cervical+pathology | | | |
| 111 | International journal of hypertension | Salman Z | 2010 | 0 | http://www.ncbi.nlm.nih.gov/pubmed/?term=High+Rate+of+Obesity-Associated+Hypertension+among+Primary+Schoolchildren+in+Sudan | | | |
| 157 | International Journal of Surgery | Abdelgadir MA | 2010 | 0 | http://www.ncbi.nlm.nih.gov/pubmed/20538080 | | | |
| 99 | International journal of surgery | Najjar H | 2010 | 0 | http://www.ncbi.nlm.nih.gov/pubmed/20601253 | | | |
| 59 | International journal of women's health | Elgaili E M | 2010 | 0 | http://www.ncbi.nlm.nih.gov/pubmed/?term=Breast+cancer+burden+in+central+Sudan+International+journal+of+women's+health | | | |
| 115 | Cancer genetics and cytogenetics | Siddiqui R | 2010 | 0 | http://www.ncbi.nlm.nih.gov/pubmed/?term=Distribution+of+common+genetic+subgroups+in+childhood+acute+lymphoblastic+leukemia+in+four+developing+countries | | | |
| 46 | Journal of oral pathology and medicine | Costea D E | 2010 | 0 | https://www.ncbi.nlm.nih.gov.ezproxy.aub.edu.lb/pubmed/?term=Adverse+effects+of+Sudanese+toombak+vs.+Swedish+snuff+on+human+oral+cells | | | |
| 29 | J Obstet Gynaecol | Naseer MA | 2011 | 0 | http://informahealthcare.com/doi/pdf/10.3109/01443615.2011.587054 | | | |
| 185 | The Gulf Journal of Oncology | Naseer MA | 2011 | 0 | http://www.ncbi.nlm.nih.gov/pubmed/21724533 | | | |
| 28 | Diabetes Research and Clinical Practice | Hussein WI | 2011 | 0 | http://www.sciencedirect.com/science/article/pii/S0168822711004013 | | | |
| 30 | Medical Principles and Practice | Al-Bannawi A | 2011 | 0 | http://www.karger.com/Article/Pdf/324553 | | | |
| 31 | The Gulf Journal of Oncology | Majumdar D | 2011 | 0 | http://www.ncbi.nlm.nih.gov/pubmed/?term=Respiratory+gated+simultaneous+integrated+boost-intensity+modulated+radiotherapy+(SIB-IMRT)+after+breast+conservative+surgery+for+carcinoma+of+the+breast%3A | | | |
| 38 | Molecular and Cellular Biochemistry | Al-Harbi EM | 2011 | 0 | http://link.springer.com/article/10.1007%2Fs11010-010-0688-y | | | |
| 21 | International Journal of Environmental Research and Public Health | Musaiger AO | 2011 | 0 | http://www.mdpi.com/1660-4601/8/9/3637 | | | |
| 24 | Journal of Obesity | Musaiger AO | 2011 | 0 | http://www.hindawi.com/journals/jobe/2011/407237/ | | | |
| 27 | JRSM Short Reports | Alhyas, L | 2011 | 0 | http://www.ncbi.nlm.nih.gov/pubmed/?term=Prevalences+of+overweight%2C+obesity%2C+hyperglycaemia%2C+hypertension+and+++++++dyslipidaemia+in+the+Gulf%3A+systematic+9 | | | |
| 32 | Journal of Neurological Sciences | Tran J | 2011 | 0 | http://www.sciencedirect.com/science/article/pii/S0022510X11002474 | | | |
| 44 | Obesity Reviews | NG SW | 2011 | 0 | http://www.ncbi.nlm.nih.gov/pubmed/?term=The+prevalence+and+trends+of+overweight%2C+obesity+and+nutrition-related+++++++non-communicable+diseases+in+the+Arabian+Gulf+States | | | |
| 22 | European Cytokine Network | Al-Habboubi HH | 2011 | 0 | http://www.jle.com/e-docs/00/04/6E/E4/vers_alt/VersionPDF.pdf | | | |
| 23 | Indian Journal of Physiology and Pharmacology | Razzak RL | 2011 | 0 | http://www.ncbi.nlm.nih.gov/pubmed/?term=Assessment+of+enhanced+endothelium-dependent+vasodilation+by+intermittent+fasting+in+Wistar+albino+rats. | | | |
| 26 | Ann Hepatology | Al-Muhannadi N | 2011 | 0 | http://www.ncbi.nlm.nih.gov/pubmed/21911893 | | | |
| 14 | Diabetes, metabolic syndrome and Obesity | Al-Hazzaa HM | 2011 | 0 | http://www.ncbi.nlm.nih.gov/pubmed/22253540 | | | |
| 20 | Disease Markers | Salem AH | 2011 | 0 | http://www.hindawi.com/journals/dm/2011/796520/abs/ | | | |
| 25 | Food and Nutrition Research | Musaiger AO | 2011 | 0 | http://www.ncbi.nlm.nih.gov/pmc/articles/PMC3171216/pdf/FNR-55-7122.pdf | | | |
| 34 | Nutrition Journal | Gharib N | 2011 | 0 | http://www.ncbi.nlm.nih.gov/pmc/articles/PMC3123629/pdf/1475-2891-10-62.pdf | | | |
| 35 | Asian Pacific Journal of Cancer Prevention | Salim EI | 2011 | 0 | http://www.apocpcontrol.org/paper_file/issue_abs/Volume12_No1/17-34%20b%2012.21%20Elsayed%20I.%20Salim.pdf | | | |
| 39 | Indian pediatrics | Jagannath V | 2011 | 0 | http://www.ncbi.nlm.nih.gov/pubmed/?term=%22Quality+assessment+of+systematic+reviews+of+health+care+interventions+using+AMSTAR%22 | | | |
| 193 | Diabetes, Metabolic Syndrome and Obesity | Musaiger AO | 2011 | 0 | http://www.ncbi.nlm.nih.gov/pubmed/21660292 | | | |
| 33 | Diabetes, Metabolic Syndrome and Obesity | Musaiger AO | 2011 | 0 | http://www.ncbi.nlm.nih.gov/pmc/articles/PMC3107691/pdf/dmso-4-089.pdf | | | |
| 39 | JAAPA | Tocci G | 2011 |  | http://www.ncbi.nlm.nih.gov/pubmed/21748956 | | | |
| 30 | Saudi Journal of Kidney Disease and Transplantation | Abid AF | 2011 | 0 | http://www.sjkdt.org/article.asp?issn=1319-2442;year=2011;volume=22;issue=6;spage=1205;epage=1207;aulast=Abid | | | |
| 260 | American Journal of Hematology | Abdulsalam AH | 2011 | 0 | http://www.ncbi.nlm.nih.gov/pubmed/21509791 | | | |
| 261 | American Journal of Hematology | Abdulsalam AH | 2011 | 0 | http://www.ncbi.nlm.nih.gov/pubmed/21465517 | | | |
| 31 | Oman Medical Journal | Mansour AA | 2011 | 0 | http://www.ncbi.nlm.nih.gov/pubmed/?term=Acute+Phase+Hyperglycemia+among+Patients+Hospitalized+with+Acute+Coronary+Syndrome%3A+Prevalence+and+Prognostic+Significance. | | | |
| 32 | International journal of hypertension | Hasan ZN | 2011 | 0 | http://dx.doi.org/10.4061/2011/701029 | | | |
| 33 | Sultan Qaboos University Medical Journal | Alrudainy LA | 2011 | 0 | http://www.ncbi.nlm.nih.gov/pmc/articles/pmid/21969893/ | | | |
| 40 | Indian Journal of Endocrinology and Metabolism | Al-Ani FS | 2011 | 0 | http://www.ijem.in/article.asp?issn=2230-8210;year=2011;volume=15;issue=2;spage=110;epage=114;aulast=Al%2DAni | | | |
| 41 | international journal of applied and basic medical research | Dezayee ZM | 2011 | 0 | http://www.ncbi.nlm.nih.gov/pmc/articles/PMC3657961/ | | | |
| 43 | Southern medical journal | Hasan ZN | 2011 | 0 | http://www.ncbi.nlm.nih.gov/pubmed/?term=%22Association+of+Chlamydia+pneumoniae+serology+and+ischemic+stroke%22+Hasan | | | |
| 193 | Eastern Mediterranean Health Journal | Abdel-Barry JA | 2011 | 0 | http://www.ncbi.nlm.nih.gov/pubmed/22259919 | | | |
| 197 | Arab Journal of Gastroenterology | Mansour AA | 2011 | 0 | http://www.ncbi.nlm.nih.gov/pubmed/21684484 | | | |
| 217 | Indian Journal of Pathology and Microbiology | Ahmed MM | 2011 | 0 | http://www.ncbi.nlm.nih.gov/pubmed/21393869 | | | |
| 265 | Saudi Journal of Kidney Disease and Transplantation | Awad SM | 2011 | 0 | http://www.ncbi.nlm.nih.gov/pubmed/22089804 | | | |
| 36 | Current Osteoporosis Reports | Fields J | 2011 | 0 | http://www.ncbi.nlm.nih.gov/pubmed/21901427 | | | |
| 27 | Indian Journal of Cancer | Yaqo RT | 2011 | 0 | http://www.indianjcancer.com/article.asp?issn=0019-509X;year=2011;volume=48;issue=4;spage=446;epage=451;aulast=Yaqo | | | |
| 34 | Journal of Pediatric Hematology/Oncology | Al-Hadad SA | 2011 | 0 | http://www.ncbi.nlm.nih.gov/pubmed/?term=Reality+of+pediatric+cancer+in+Iraq. | | | |
| 35 | Saudi Journal of Kidney Disease and Transplantation | Alsaedi AJ | 2011 | 0 | http://www.sjkdt.org/article.asp?issn=1319-2442;year=2011;volume=22;issue=5;spage=1044;epage=1045;aulast=Alsaedi | | | |
| 38 | Asian Pacific Journal of Cancer Prevention | Othman RT | 2011 | 0 | http://www.apocpcontrol.org/page/apjcp_issues_view.php?sid=Entrez:PubMed&id=pmid:21875278&key=2011.12.5.1261 | | | |
| 44 | BMC Research Notes | Al-Mumen MM | 2011 | 0 | http://www.ncbi.nlm.nih.gov/pubmed/21443808 | | | |
| 49 | Neurosciences | Al-Mendalawi MD | 2011 | 0 | http://www.ncbi.nlm.nih.gov/pubmed/21206456 | | | |
| 52 | Neurological Sciences | Mignarri A | 2011 | 0 | http://www.ncbi.nlm.nih.gov/pubmed/20857162 | | | |
| 195 | Oman Medical Journal | Qasim BJ | 2011 | 0 | http://www.ncbi.nlm.nih.gov/pubmed/22125723 | | | |
| 37 | Conflict and Health | Alaani S | 2011 | 0 | http://www.conflictandhealth.com/content/5/1/15 | | | |
| 46 | Pediatr Blood Cancer | Moleti ML | 2011 | 0 | http://www.ncbi.nlm.nih.gov/pubmed/21298740 | | | |
| 48 | Saudi Medical Journal | Omer AR | 2011 | 0 | http://www.ncbi.nlm.nih.gov/pubmed/21212918 | | | |
| 198 | BMJ Case Reports | Amin OS | 2011 | 0 | http://www.ncbi.nlm.nih.gov/pubmed/22715252 | | | |
| 196 | Food and Chemical Toxicology | Sulaiman GM | 2011 | 0 | http://www.ncbi.nlm.nih.gov/pubmed/21723909 | | | |
| 405 | Medical Principles and Practice | Al-Fraij AK | 2011 | 0 | http://www.ncbi.nlm.nih.gov/pubmed/21455005 | | | |
| 457 | The Gulf Journal of Oncology | Al-Saleh N | 2011 | 0 | http://www.ncbi.nlm.nih.gov/pubmed/21177213 | | | |
| 603 | Oral surgery, oral medicine, oral pathology, oral radiology, and endodontics | Ibrahim HH | 2011 | 0 | http://www.ncbi.nlm.nih.gov.ezproxy.aub.edu.lb/pubmed/?term=Malignant+gastrointestinal+stromal+tumor+of+the+tongue%3A+case+report+and+review+of+the+literature. | | | |
| 672 | Pediatric Dermatology | Najem N | 2011 | 0 | http://www.ncbi.nlm.nih.gov/pubmed/?term=Aleukemic+leukemia+cutis+in+a+child+preceding+T-cell+acute+lymphoblastic+leukemia. | | | |
| 724 | Indian Journal of Pathology and Microbiology | Singh NG | 2011 | 0 | http://www.ncbi.nlm.nih.gov.ezproxy.aub.edu.lb/pubmed/21393923 | | | |
| 725 | Indian Journal of Pathology and Microbiology | Singh NG | 2011 | 0 | http://www.ncbi.nlm.nih.gov.ezproxy.aub.edu.lb/pubmed/21623105 | | | |
| 726 | Medical Principles and Practice | Singh NG | 2011 | 0 | http://www.ncbi.nlm.nih.gov.ezproxy.aub.edu.lb/pubmed/?term=Mixed+adenocarcinoma-carcinoid+(collision+tumor)+of+the+appendix | | | |
| 741 | The Gulf Journal of Oncology | Usmani S | 2011 | 0 | http://www.ncbi.nlm.nih.gov.ezproxy.aub.edu.lb/pubmed/?term=Extensive+visceral+calcification+demonstrated+on+Tc-99m+MDP+bone+scan+in+patient+with+sphenoidal+sinus+carcinoma+and+hypercalcaemia+of+malignancy%3A+a+bad+prognostic+sign | | | |
| 872 | Breast J | Al-Khawari HA | 2011 | 0 | http://www.ncbi.nlm.nih.gov/pubmed/21929558 | | | |
| 892 | Hematology Reports | Záme?níkova A | 2011 | 0 | http://www.ncbi.nlm.nih.gov/pubmed/22184534 | | | |
| 893 | Medical Principles and Practice | Usmani S | 2011 | 0 | http://www.ncbi.nlm.nih.gov/pubmed/21576997 | | | |
| 906 | Circulation Cardiovascular Quality and Outcomes | Zubaid M | 2011 | 0 | http://www.ncbi.nlm.nih.gov/pubmed/21772004 | | | |
| 12 | Vascular Health and Risk Management | Olusi SO | 2011 | 0 | http://www.ncbi.nlm.nih.gov/pubmed/?term=Prevalence+of+LDL+atherogenic+phenotype+in+patients+with+systemic+lupus+++++++erythematosus | | | |
| 13 | Journal of Cross-cultural Gerontology | Al-Kandari AA | 2011 | 0 | http://www.ncbi.nlm.nih.gov/pubmed/?term=Relationship+of+strength+of+social+support+and+frequency+of+social+contact+with+++++++hypertension+and+general+health+status+among+older+adults+in+the+mobile+care+unit+++++++in+Kuwait. | | | |
| 130 | Journal of Periodontology | Al-Khabbaz AK | 2011 | 0 | http://www.ncbi.nlm.nih.gov/pubmed/?term=Knowledge+about+the+association+between+periodontal+diseases+and+diabetes+mellitus%3A+contrasting+dentists+and+physicians | | | |
| 149 | Medical Principles and Practice | Awad A | 2011 | 0 | http://www.ncbi.nlm.nih.gov.ezproxy.aub.edu.lb/pubmed/?term=Diabetic+patients'+knowledge+of+therapeutic+goals+in+Kuwait | | | |
| 164 | Journal of Infection and Public Health | Chehadeh W | 2011 | 0 | http://www.ncbi.nlm.nih.gov.ezproxy.aub.edu.lb/pubmed/?term=Hepatitis+C+virus+infection+in+a+population+with+high+incidence+of+type+2+diabetes%3A+impact+on+diabetes+complications | | | |
| 373 | Disease Markers | Al Khaldi RM | 2011 | 0 | http://www.ncbi.nlm.nih.gov/pubmed/21694446 | | | |
| 406 | Medical Principles and Practice | Al-Ghimlas F | 2011 | 0 | http://www.ncbi.nlm.nih.gov/pubmed/21577004 | | | |
| 415 | Journal of Obesity | Al-Isa AN | 2011 | 0 | http://www.ncbi.nlm.nih.gov/pubmed/21603221 | | | |
| 424 | Open Cardiovascular Medicine Journal | Al-Jarallah M | 2011 | 0 | http://www.ncbi.nlm.nih.gov/pubmed/21886684 | | | |
| 444 | Medical Principles and Practice | Al-Mutairi N | 2011 | 0 | http://www.ncbi.nlm.nih.gov/pubmed/21454995 | | | |
| 468 | Obesity Surgery | Alasfar F | 2011 | 0 | http://www.ncbi.nlm.nih.gov/pubmed/21633821 | | | |
| 486 | Internet Journal of Pediatrics and Neonatology | Alotaibi S | 2011 | 0 | http://www.ncbi.nlm.nih.gov/pubmed/21760757 | | | |
| 560 | Public Health Nutrition | El-Ghaziri M | 2011 | 0 | http://www.ncbi.nlm.nih.gov/pubmed/21756428 | | | |
| 566 | Ecology of Food and Nutrition | El-Sabban F | 2011 | 0 | http://www.ncbi.nlm.nih.gov/pubmed/21888596 | | | |
| 604 | Public Health Nutrition | Jackson RT | 2011 | 0 | http://www.ncbi.nlm.nih.gov.ezproxy.aub.edu.lb/pubmed/20920388 | | | |
| 620 | Journal of neuro-oncology | Katchy KC | 2011 | 0 | http://www.ncbi.nlm.nih.gov.ezproxy.aub.edu.lb/pubmed/?term=Intracranial+tumors+in+Kuwait%3A+a+15-year+survey. | | | |
| 622 | Scandinavian Journal of Urology and Nephrology | Kehinde EO | 2011 | 0 | http://www.ncbi.nlm.nih.gov.ezproxy.aub.edu.lb/pubmed/?term=Comparison+of+the+sensitivity+and+specificity+of+urine+cytology%2C+urinary+nuclear+matrix+protein-22+and+multitarget+fluorescence+in+situ+hybridization+assay+in+the+detection+of+bladder+cancer. | | | |
| 635 | Obesity surgery | Khoursheed M | 2011 | 0 | http://www.ncbi.nlm.nih.gov.ezproxy.aub.edu.lb/pubmed/20596789 | | | |
| 665 | Mycoses | Mokaddas E | 2011 | 0 | http://www.ncbi.nlm.nih.gov/pubmed/?term=Prevalence+of+Candida+dubliniensis+among+cancer+patients+in+Kuwait%3A+a+5-year+retrospective+study. | | | |
| 677 | Medical Principles and Practice | Narayanaswamy A | 2011 | 0 | http://www.ncbi.nlm.nih.gov.ezproxy.aub.edu.lb/pubmed/?term=Detection+rate+and+clinical+pattern+of+prostate+cancer+in+Kuwait%3A+a+single-center+experience. | | | |
| 705 | Clinical Chemistry | Safar FH | 2011 | 0 | http://www.ncbi.nlm.nih.gov.ezproxy.aub.edu.lb/pubmed/?term=Computational+methods+are+significant+determinants+of+the+associations+and+definitions+of+insulin+resistance+using+the+homeostasis+model+assessment+in+women+of+reproductive+age | | | |
| 706 | Biological Trace Element Research | Saleh F | 2011 | 0 | http://www.ncbi.nlm.nih.gov.ezproxy.aub.edu.lb/pubmed/?term=Abnormal+blood+levels+of+trace+elements+and+metals%2C+DNA+damage%2C+and+breast+cancer+in+the+state+of+Kuwait | | | |
| 713 | The Gulf Journal of Oncology | Samir SM | 2011 | 0 | http://www.ncbi.nlm.nih.gov.ezproxy.aub.edu.lb/pubmed/?term=Medullary+carcinoma+of+the+breast%3A+ten+year+clinical+experience+of+the+Kuwait+cancer+control+centre | | | |
| 732 | Medical Principles and Practice | Thalib L | 2011 | 0 | http://www.ncbi.nlm.nih.gov.ezproxy.aub.edu.lb/pubmed/?term=Regional+variability+in+hospital+mortality+in+patients+hospitalized+with+ST-segment+elevation+myocardial+infarction%3A+findings+from+the+Gulf+Registry+of+Acute+Coronary+Even | | | |
| 393 | Journal of Medical Virology | Al-Awadhi R | 2011 | 0 | http://www.ncbi.nlm.nih.gov/pubmed/21264866 | | | |
| 740 | Nuclear Medicine Communications | Usmani S | 2011 | 0 | http://www.ncbi.nlm.nih.gov.ezproxy.aub.edu.lb/pubmed/?term=Selective+approach+to+radionuclide-guided+sentinel+lymph+node+biopsy+in+high-risk+ductal+carcinoma+in+situ+of+the+breast | | | |
| 9 | JRSM Short Reports | Alhyas, L | 2011 | 0 | http://www.ncbi.nlm.nih.gov/pubmed/?term=Prevalences+of+overweight%2C+obesity%2C+hyperglycaemia%2C+hypertension+and+++++++dyslipidaemia+in+the+Gulf%3A+systematic+9 | | | |
| 10 | J Neurol Sci. | Tran J | 2011 | 0 | http://www.ncbi.nlm.nih.gov/pubmed/?term=The+population+attributable+fraction+of+stroke+associated+with+high+blood+++++++pressure+in+the+Middle+East+and+North+Africa. | | | |
| 16 | Obesity Reviews | NG SW | 2011 | 0 | http://www.ncbi.nlm.nih.gov/pubmed/?term=The+prevalence+and+trends+of+overweight%2C+obesity+and+nutrition-related+++++++non-communicable+diseases+in+the+Arabian+Gulf+States | | | |
| 173 | Expert Opinion on Theraputic Targets | Hasan A | 2011 | 0 | http://www.ncbi.nlm.nih.gov/pubmed/21870995 | | | |
| 198 | Metabolic Syndrome and Related Disorders | Ohaeri JU | 2011 | 0 | http://www.ncbi.nlm.nih.gov/pubmed/20964513 | | | |
| 436 | Methods in Molecular Biology | Al-Mulla F | 2011 | 0 | http://www.ncbi.nlm.nih.gov/pubmed/21370011 | | | |
| 578 | Journal of Nuclear Medicine Technology | Ghanem MA | 2011 | 0 | http://www.ncbi.nlm.nih.gov.ezproxy.aub.edu.lb/pubmed/21321247 | | | |
| 879 | Journal of Cytology | Das DK | 2011 | 0 | http://www.ncbi.nlm.nih.gov/pubmed/22090706 | | | |
| 132 | Experimental and Toxicologic Pathology | Al-Khalifa A | 2011 | 0 | http://www.ncbi.nlm.nih.gov/pubmed/?term=Low+carbohydrate+ketogenic+diet+prevents+the+induction+of+diabetes+using+streptozotocin+in+rats | | | |
| 140 | Molecular Biosystems | Al-Mulla F | 2011 | 0 | http://www.ncbi.nlm.nih.gov/pubmed/21850315 | | | |
| 141 | Medical Principles and Practice | Al-Saeedi F | 2011 | 0 | http://www.ncbi.nlm.nih.gov/pubmed/21252566 | | | |
| 160 | American Journal of Physiology Endocrinology and Metabolism | Bitar MS | 2011 | 0 | http://www.ncbi.nlm.nih.gov/pubmed/?term=A+defect+in+Nrf2+signaling+constitutes+a+mechanism+for+cellular+stress+hypersensitivity+in+a+genetic+rat+model+of+type+2+diabetes | | | |
| 169 | International Journal of Biological Sciences | El-Sayyad HI | 2011 | 0 | http://www.ncbi.nlm.nih.gov/pubmed/?term=Protective+effects+of+Morus+alba+leaves+extract+on+ocular+functions+of+pups+from+diabetic+and+hypercholesterolemic+mother+rats | | | |
| 375 | Disease Markers | Al Mutairi SS | 2011 | 0 | http://www.ncbi.nlm.nih.gov/pubmed/21846943 | | | |
| 381 | International Journal of Oncology | Al Saleh S | 2011 | 0 | http://www.ncbi.nlm.nih.gov/pubmed/21318221 | | | |
| 382 | PLoS One | Al Saleh S | 2011 | 0 | http://www.ncbi.nlm.nih.gov/pubmed/21713035 | | | |
| 517 | Journal of Cardiovascular Pharmacology | Benter IF | 2011 | 0 | http://www.ncbi.nlm.nih.gov/pubmed/21326110 | | | |
| 518 | J Neurotrauma | Benter IF | 2011 | 0 | http://www.ncbi.nlm.nih.gov/pubmed/21510818 | | | |
| 554 | Experimental and Molecular Pathology | El-Abdallah AA | 2011 | 0 | http://www.ncbi.nlm.nih.gov/pubmed/20950609 | | | |
| 563 | International Journal of Immunopathology and Pharmacology | El-Hashim AZ | 2011 | 0 | http://www.ncbi.nlm.nih.gov/pubmed/21496385 | | | |
| 577 | Anticancer Research | Ford CH | 2011 | 0 | http://www.ncbi.nlm.nih.gov.ezproxy.aub.edu.lb/pubmed/?term=Reassessment+of+estrogen+receptor+expression+in+human+breast+cancer+cell+lines. | | | |
| 579 | Prilozi | Gjorgov AN | 2011 | 0 | http://www.ncbi.nlm.nih.gov/pubmed/22286632 | | | |
| 613 | Journal of Cytology | Kapila K | 2011 | 0 | http://www.ncbi.nlm.nih.gov.ezproxy.aub.edu.lb/pubmed/?term=Her-2+neu+(Cerb-B2)+expression+in+fine+needle+aspiration+samples+of+breast+carcinoma%3A+A+pilot+study+comparing+FISH%2C+CISH+and+immunocytochemistry. | | | |
| 627 | European Journal of Applied Physiology | Khadadah M | 2011 | 0 | http://www.ncbi.nlm.nih.gov.ezproxy.aub.edu.lb/pubmed/?term=Effect+of+acute+cold+exposure+on+lung+perfusion+and+tracheal+smooth+muscle+contraction+in+rabbit. | | | |
| 632 | Medical Mycology : official publication of the International Society for Human and Animal Mycology | Khan Z | 2011 | 0 | http://www.ncbi.nlm.nih.gov.ezproxy.aub.edu.lb/pubmed/?term=Isolation+of+Cryptococcus+magnus+and+Cryptococcus+chernovii+from+nasal+cavities+of+pediatric+patients+with+acute+lymphoblastic+leukemia. | | | |
| 885 | International Immunopharmacology | Ezeamuzie CI | 2011 | 0 | http://www.ncbi.nlm.nih.gov/pubmed/21481814 | | | |
| 540 | Journal of Cytology | Das DK | 2011 | 0 | http://www.ncbi.nlm.nih.gov/pubmed/22090704 | | | |
| 541 | Diagnostic Cytopathology | Das DK | 2011 | 0 | http://www.ncbi.nlm.nih.gov/pubmed/21162094 | | | |
| 6 | Cerebrovascular Diseases | Albaker, O | 2011 | 0 | http://www.ncbi.nlm.nih.gov/pubmed/?term=Early+stroke+following+acute+myocardial+infa7ion%3A+incidence%2C+predictors+and+++++++outcome+in+six+Middle-Eastern+countries | | | |
| 8 | Open Cardiovascular Medicine Journal | Alanbaei, M | 2011 | 0 | http://www.ncbi.nlm.nih.gov/pubmed/21915225 | | | |
| 139 | Archives of Iranian Medicine | Al-Majed HT | 2011 | 0 | http://www.ncbi.nlm.nih.gov/pubmed/22039842 | | | |
| 209 | Clinical Medicine and Research | Thalib L | 2011 | 0 | http://www.ncbi.nlm.nih.gov/pubmed/?term=Impact+of+diabetic+status+on+the+hyperglycemia-induced+adverse+risk+of+short+term+outcomes+in+hospitalized+patients+with+acute+coronary+syndromes+in+the+Middle+East%3A+findings+from+the+Gulf+registry+of+Acute+Coronary+Events+(Gulf+RACE) | | | |
| 412 | Diabetes, Metabolic Syndrome and Obesity | Al-Hazzaa HM | 2011 | 0 | http://www.ncbi.nlm.nih.gov/pubmed/22253540 | | | |
| 456 | The Gulf Journal of Oncology | Al-Saleh K | 2011 | 0 | http://www.ncbi.nlm.nih.gov/pubmed/21177204 | | | |
| 526 | Heart Views | Christus T | 2011 | 0 | http://www.ncbi.nlm.nih.gov/pubmed/21731802 | | | |
| 749 | The Gulf Journal of Oncology | El-Kabany M | 2011 | 0 | http://www.ncbi.nlm.nih.gov/pubmed/21177214 | | | |
| 361 | Clinical Chemistry and Laboratory Medicine | Akanji AO | 2011 | 0 | http://www.ncbi.nlm.nih.gov/pubmed/21663566 | | | |
| 471 | Medical Oncology | Alawadi S | 2011 | 0 | http://www.ncbi.nlm.nih.gov/pubmed/20443084 | | | |
| 490 | The Gulf Journal of Oncology | Alshemmari S | 2011 | 0 | http://www.ncbi.nlm.nih.gov/pubmed/21177205 | | | |
| 514 | Anti-cancer Agents in Medicinal Chemistry | Benov L | 2011 | 0 | http://www.ncbi.nlm.nih.gov/pubmed/21355847 | | | |
| 520 | The Gulf Journal of Oncology | Bobin JY | 2011 | 0 | http://www.ncbi.nlm.nih.gov/pubmed/21177208 | | | |
| 11 | International Journal of Immunopathology and Pharmacology | Sharma, JN | 2011 | 0 | http://www.ncbi.nlm.nih.gov/pubmed/?term=The+effects+of+captopril+on+cardiac+regression%2C+blood+pressure+and+bradykinin+++++++components+in+diabetic+Wistar+Kyoto+rats | | | |
| 414 | Journal of Histochemistry and Cytochemistry | Al-Humood SA | 2011 | 0 | http://www.ncbi.nlm.nih.gov/pubmed/21832150 | | | |
| 454 | Journal of Nuclear Medicine Technology | Al-Saeedi F | 2011 | 0 | http://www.ncbi.nlm.nih.gov/pubmed/22080437 | | | |
| 75 | European heart journal | Azar R R | 2011 |  | Not Found | |  |  |
| 160 | Psychooncology | Doumit M A | 2011 |  | Not Found | |  |  |
| 367 | Annales de dermatologie et de venereologie | Moutran R | 2011 |  | Abstract Not Found | |  |  |
| 529 | Subst Use Misuse | Wells J E | 2011 |  | Not Found | |  |  |
| 193 | J Med Liban | Eloubeidi M A | 2011 | 0 | http://www.ncbi.nlm.nih.gov/pubmed/?term=Transduodenal+EUS-guided+FNA+of+the+right+adrenal+gland+to+diagnose+lung+cancer+where+percutaneous+approach+was+not+possible | | | |
| 194 | Journal of clinical lipidology | El-Rassi I | 2011 | 0 | http://www.ncbi.nlm.nih.gov/pubmed/?term=Fatal+cardiac+atherosclerosis+in+a+child+10+years+after+liver+transplantation%3A+a+case+report+and+a+review | | | |
| 204 | Molecular genetics and metabolism | Fahed A C | 2011 | 0 | http://www.ncbi.nlm.nih.gov/pubmed/21145767 | | | |
| 207 | Blood coagulation & fibrinolysis | Farah R A | 2011 | 0 | http://www.ncbi.nlm.nih.gov/pubmed/?term=Acquired+protein+C+deficiency+in+a+child+with+acute+myelogenous+leukemia%2C+splenic%2C+renal%2C+and+intestinal+infarction | | | |
| 210 | World journal of surgical oncology | Faraj W | 2011 | 0 | http://www.ncbi.nlm.nih.gov/pubmed/?term=Successful+one+stage+operation+for+a+synchronous%2C+duodenal+carcinoma%2C+colonic+carcinoma+and+renal+oncocytoma+in+an+adult+patient | | | |
| 325 | J Med Liban | Kattan J | 2011 | 0 | http://www.ncbi.nlm.nih.gov/pubmed/?term=Inflammatory+breast+cancer+with+refractory+diarrhea%3A+a+case+report+kattan+2011 | | | |
| 384 | Journal of anesthesia | Naja Z M | 2011 | 0 | http://www.ncbi.nlm.nih.gov/pubmed/?term=Multilevel+nerve+stimulator-guided+paravertebral+block+as+a+sole+anesthetic+technique+for+breast+cancer+surgery+in+morbidly+obese+patients | | | |
| 431 | Coronary artery disease | Rebeiz A G | 2011 | 0 | http://www.ncbi.nlm.nih.gov/pubmed/?term=Plasma+myeloperoxidase+concentration+predicts+the+presence+and+severity+of+coronary+disease+in+patients+with+chest+pain+and+negative+troponin-T | | | |
| 507 | Rare tumors | Soubra A | 2011 | 0 | http://www.ncbi.nlm.nih.gov/pubmed/?term=Peri-ampullary+mixed+acinar-endocrine+carcinoma | | | |
| 600 | Journal of Pediatric Hematology/Oncology | El-Bitar MK | 2011 | 0 | http://www.ncbi.nlm.nih.gov/pubmed/?term=Severe+hypoglycemic+seizures+in+a+child+receiving+6-mercaptopurine. | | | |
| 683 | Archives of Gynecology and Obstetrics | Oweini H | 2011 | 0 | http://www.ncbi.nlm.nih.gov.ezproxy.aub.edu.lb/pubmed/?term=Successful+pregnancy+involving+a+man+with+chronic+myeloid+leukemia+on+dasatinib. | | | |
| 686 | BMJ Case Reports | Riachy MA | 2011 | 0 | http://www.ncbi.nlm.nih.gov.ezproxy.aub.edu.lb/pubmed/?term=Streptococcus+pneumoniae+causing+septic+arthritis+with+shock+and+revealing+multiple+myeloma. | | | |
| 723 | Spine J | Yazbeck PG | 2011 | 0 | http://www.ncbi.nlm.nih.gov/pubmed/21835701 | | | |
| 1001 | Clin Med Insights Case Rep | Kossaify A | 2011 | 0 | http://www.ncbi.nlm.nih.gov/pubmed/22174592 | | | |
| 1002 | Case Rep Med | Kossaify A | 2011 | 0 | http://www.ncbi.nlm.nih.gov/pubmed/22162706 | | | |
| 1003 | Case Rep Med | Kossaify A | 2011 | 0 | http://www.ncbi.nlm.nih.gov/pubmed/22110511 | | | |
| 6 | The American surgeon | Abboud B | 2011 | 0 | http://www.ncbi.nlm.nih.gov/pubmed/22273220 | | | |
| 20 | Aging & mental health | AbiHabib L E | 2011 | 0 | http://www.ncbi.nlm.nih.gov/pubmed/?term=Developing+capacities+in+aging+studies+in+the+Middle+East%3A+Implementation+of+an+Arabic+version+of+the+CANE+IV+among+community-dwelling+older+adults+in+Lebanon | | | |
| 61 | Health care for women international | Arevian M | 2011 | 0 | http://www.ncbi.nlm.nih.gov/pubmed/?term=Beliefs+related+to+breast+cancer+and+breast+cancer+screening+among+Lebanese+Armenian+women | | | |
| 109 | J Med Liban | Chacar H R | 2011 | 0 | http://www.ncbi.nlm.nih.gov/pubmed/?term=Public+schools+adolescents'+obesity+and+growth+curves+in+Lebanon | | | |
| 125 | Journal of endocrinological investigation | Chedid R | 2011 | 0 | http://www.ncbi.nlm.nih.gov/pubmed/?term=Serum+uric+acid+in+relation+with+the+metabolic+syndrome+components+and+adiponectin+levels+in+Lebanese+University+students | | | |
| 149 | Genetic testing and molecular biomarkers | Darazy M | 2011 | 0 | http://www.ncbi.nlm.nih.gov/pubmed/?term=CYP1A1%2C+CYP2E1%2C+and+GSTM1+gene+polymorphisms+and+susceptibility+to+colorectal+and+gastric+cancer+among+Lebanese | | | |
| 166 | Saudi journal of kidney diseases and transplantation | Ei Wafai R J | 2011 | 0 | http://www.ncbi.nlm.nih.gov/pubmed/21422625 | | | |
| 183 | Journal of nuclear cardiology | El Sibai K | 2011 | 0 | http://www.ncbi.nlm.nih.gov/pubmed/?term=Evaluation+of+the+appropriateness+criteria+for+coronary+computed+tomography+angiography+in+an+academic+medical+center+in+a+developing+country%3A+comparison+of+the+2006+and+2010+criteria | | | |
| 237 | Clinical endocrinology | Gannage-Yared M H | 2011 | 0 | http://www.ncbi.nlm.nih.gov/pubmed/?term=Relation+between+androgens+and+cardiovascular+risk+factors+in+a+young+population | | | |
| 249 | Supportive care in cancer | Ghosn M | 2011 | 0 | http://www.ncbi.nlm.nih.gov/pubmed/?term=Experience+with+palliative+care+in+patients+with+advanced+cancer+at+a+tertiary+care+hospital+in+a+developing+country | | | |
| 251 | The oncologist | Ghosn M | 2011 | 0 | http://www.ncbi.nlm.nih.gov/pubmed/?term=Triple-negative+breast+cancer+in+Lebanon%3A+a+case+series | | | |
| 296 | International journal of obesity | Issa C | 2011 | 0 | http://www.ncbi.nlm.nih.gov/pubmed/?term=A+Mediterranean+diet+pattern+with+low+consumption+of+liquid+sweets+and+refined+cereals+is+negatively+associated+with+adiposity+in+adults+from+rural+Lebanon | | | |
| 375 | Pediatric neurology | Muwakkit S A | 2011 | 0 | http://www.ncbi.nlm.nih.gov/pubmed/?term=Inherited+thrombophilia+in+childhood+arterial+stroke%3A+data+from+Lebanon+2011 | | | |
| 380 | Integrative cancer therapies | Naja F | 2011 | 0 | http://www.ncbi.nlm.nih.gov/pubmed/?term=Complementary+and+alternative+medicine+use+among+pediatric+patients+with+leukemia%3A+the+case+of+Lebanon+naja | | | |
| 381 | European journal of nutrition | Naja F | 2011 | 0 | http://www.ncbi.nlm.nih.gov/pubmed/?term=Association+between+dietary+patterns+and+the+risk+of+metabolic+syndrome+among+Lebanese+adults+naja+2011 | | | |
| 382 | Public Health Nutrition | Naja F | 2011 | 0 | http://www.ncbi.nlm.nih.gov/pubmed/?term=Dietary+patterns+and+their+association+with+obesity+and+sociodemographic+factors+in+a+national+sample+of+Lebanese+adults | | | |
| 441 | Journal of the renin-angiotensin-aldosterone system | Saab Y B | 2011 | 0 | http://www.ncbi.nlm.nih.gov/pubmed/?term=The+association+of+hypertension+with+renin-angiotensin+system+gene+polymorphisms+in+the+Lebanese+population | | | |
| 442 | Pediatric blood & cancer | Saad R | 2011 | 0 | http://www.ncbi.nlm.nih.gov/pubmed/?term=Bereaved+parental+evaluation+of+the+quality+of+a+palliative+care+program+in+Lebanon | | | |
| 447 | PLoS One | Saade S | 2011 | 0 | http://www.ncbi.nlm.nih.gov/pubmed/?term=Large+scale+association+analysis+identifies+three+susceptibility+loci+for+coronary+artery+disease+saade | | | |
| 454 | Eastern Mediterranean health journal | Salameh P | 2011 | 0 | http://www.ncbi.nlm.nih.gov/pubmed/?term=Obesity-associated+distress+in+Lebanese+adolescents%3A+an+exploratory+look+at+a+large+cohort+of+students | | | |
| 456 | International journal of occupational medicine and environmental health | Salameh P | 2011 | 0 | http://www.ncbi.nlm.nih.gov/pubmed/?term=Validation+of+the+respiratory+toxics+exposure+score+(RTES)+for+chronic+obstructive+pulmonary+disease+screening | | | |
| 464 | Disease markers | Salem A H | 2011 | 0 | http://www.ncbi.nlm.nih.gov/pubmed/?term=Genetic+polymorphism+of+the+glutathione+S-transferase+M1+and+T1+genes+in+three+distinct+Arab+populations | | | |
| 524 | Clinical epidemiology | Waked M | 2011 | 0 | http://www.ncbi.nlm.nih.gov/pubmed/?term=Chronic+obstructive+pulmonary+disease+prevalence+in+Lebanon%3A+a+cross-sectional+descriptive+study | | | |
| 532 | Asia Pacific journal of clinical nutrition | Yahia N | 2011 | 0 | http://www.ncbi.nlm.nih.gov/pubmed/?term=Dieting+practices+and+body+image+perception+among+Lebanese+university+students | | | |
| 540 | Croatian medical journal | Zaraket R | 2011 | 0 | http://www.ncbi.nlm.nih.gov/pubmed/?term=2011+zakaret+Parental+perceptions+and+beliefs+about+childhood+asthma%3A+a+cross-sectional+study | | | |
| 549 | J Med Liban | Abboud B | 2011 | 0 | http://www.ncbi.nlm.nih.gov/pubmed/22746009 | | | |
| 598 | International Journal of General Medicine | El Baba K | 2011 | 0 | http://www.ncbi.nlm.nih.gov/pubmed/?term=Seasonal+variation+of+vitamin+D+and+HbA(1c)+levels+in+patients+with+type+1+diabetes+mellitus+in+the+Middle+East. | | | |
| 652 | The british journal of nutrition | Liu A | 2011 | 0 | http://www.ncbi.nlm.nih.gov.ezproxy.aub.edu.lb/pubmed/21736824 | | | |
| 656 | Genetic testing and molecular biomarkers | Mahfouz RA | 2011 | 0 | http://www.ncbi.nlm.nih.gov.ezproxy.aub.edu.lb/pubmed/21198321 | | | |
| 663 | North American journal of medical sciences | Merhi BA | 2011 | 0 | http://www.ncbi.nlm.nih.gov.ezproxy.aub.edu.lb/pubmed/22540059 | | | |
| 672 | Public Health Nutrition | Naja F | 2011 | 0 | http://www.ncbi.nlm.nih.gov.ezproxy.aub.edu.lb/pubmed/21557871 | | | |
| 71 | The American journal of cardiology | Azar M | 2011 | 0 | http://www.ncbi.nlm.nih.gov/pubmed/?term=Comparison+of+the+effects+of+combination+atorvastatin+(40+mg)+%2B+ezetimibe+(10+mg)+versus+atorvastatin+(40+mg)+alone+on+secretory+phospholipase+A2+activity+in+patients+with+stable+coronary+artery+disease+or+corona | | | |
| 248 | Medical oncology | Ghosn M | 2011 | 0 | http://www.ncbi.nlm.nih.gov/pubmed/21136213 | | | |
| 274 | J Med Liban | Hamdan R | 2011 | 0 | http://www.ncbi.nlm.nih.gov/pubmed/?term=Benefit+and+tolerability+of+the+coadministration+of+ezetimibe+and+atorvastatin+in+acute+coronary+syndrome+patients | | | |
| 343 | Preventive medicine | Lakkis N A | 2011 | 0 | http://www.ncbi.nlm.nih.gov/pubmed/?term=The+effect+of+two+types+of+sms-texts+on+the+uptake+of+screening+mammogram%3A+a+randomized+controlled+trial | | | |
| 471 | Telemedicine journal and e-health | Salti H | 2011 | 0 | http://www.ncbi.nlm.nih.gov/pubmed/?term=Nonmydriatic+retinal+image+review+at+time+of+endocrinology+visit+results+in+short-term+HbA1c+reduction+in+poorly+controlled+patients+with+diabetic+retinopathy | | | |
| 18 | International journal of clinical oncology | Abi Saad G S | 2011 | 0 | http://www.ncbi.nlm.nih.gov/pubmed/21258837 | | | |
| 63 | Current medical research and opinion | Arnaout M S | 2011 | 0 | http://www.ncbi.nlm.nih.gov/pubmed/?term=Hypertension+and+its+management+in+countries+in+Africa+and+the+Middle+East%2C+with+special+reference+to+the+place+of+beta-blockade | | | |
| 77 | J Med Liban | Azzi N | 2011 | 0 | http://www.ncbi.nlm.nih.gov/pubmed/?term=The+radial+approach+in+the+cardiac+catheterization+laboratory%3A+is+it+meant+to+become+the+gold+standard%3F | | | |
| 139 | Journal of pediatric hematology/oncology | Daher M | 2011 | 0 | http://www.ncbi.nlm.nih.gov/pubmed/?term=Opioids+for+cancer+pain+in+the+Middle+Eastern+countries%3A+a+physician+point+of+view | | | |
| 197 | The International journal of developmental biology | El-Saghir J A | 2011 | 0 | http://www.ncbi.nlm.nih.gov/pubmed/?term=Connexins%3A+a+junctional+crossroad+to+breast+cancer | | | |
| 203 | Nutrition & metabolism | Fahed A C | 2011 | 0 | http://www.ncbi.nlm.nih.gov/pubmed/21513517 | | | |
| 281 | International journal of clinical oncology | Hatoum H A | 2011 | 0 | http://www.ncbi.nlm.nih.gov/pubmed/?term=Metastasis+of+colorectal+carcinoma+to+the+testes%3A+clinical+presentation+and+possible+pathways | | | |
| 341 | Journal of molecular and cellular cardiology | Kurdi M | 2011 | 0 | http://www.ncbi.nlm.nih.gov/pubmed/?term=Three+4-letter+words+of+hypertension-related+cardiac+hypertrophy%3A+TRPC%2C+mTOR%2C+and+HDAC | | | |
| 369 | Hemoglobin | Musallam K M | 2011 | 0 | http://www.ncbi.nlm.nih.gov/pubmed/?term=Cerebral+infarction+in+children+with+sickle+cell+disease%3A+a+concise+overview+musallam | | | |
| 422 | Critical reviews in oncology/hematology | Otrock Z K | 2011 | 0 | http://www.ncbi.nlm.nih.gov/pubmed/?term=Is+VEGF+a+predictive+biomarker+to+anti-angiogenic+therapy%3F+otrock | | | |
| 438 | Seminars in cancer biology | Saab R | 2011 | 0 | http://www.ncbi.nlm.nih.gov/pubmed/?term=saab+Senescence+and+pre-malignancy%3A+how+do+tumors+progress%3F | | | |
| 440 | Current topics in developmental biology | Saab R | 2011 | 0 | http://www.ncbi.nlm.nih.gov/pubmed/?term=Myogenesis+and+rhabdomyosarcoma+the+Jekyll+and+Hyde+of+skeletal+muscle | | | |
| 482 | Vaccine | Seoud M | 2011 | 0 | http://www.ncbi.nlm.nih.gov/pubmed/?term=seoud+2011+Cervical+adenocarcinoma%3A+moving+towards+better+prevention | | | |
| 490 | Chemotherapy | Shamseddine A I | 2011 | 0 | http://www.ncbi.nlm.nih.gov/pubmed/?term=Platinum-based+compounds+for+the+treatment+of+metastatic+breast+cancer+shamseddine+2011 | | | |
| 520 | Molecules | Tohme R | 2011 | 0 | http://www.ncbi.nlm.nih.gov/pubmed/?term=A+journey+under+the+sea%3A+the+quest+for+marine+anti-cancer+alkaloids | | | |
| 618 | Cellular Immunology | Haddad JJ | 2011 | 0 | http://www.ncbi.nlm.nih.gov/pubmed/?term=NF-%D8%AE%D8%9BB+cellular+and+molecular+regulatory+mechanisms+and+pathways%3A+therapeutic+pattern+or+pseudoregulation%3F | | | |
| 621 | Journal of Thyroid Research | Hage M | 2011 | 0 | http://www.ncbi.nlm.nih.gov/pubmed/21785689 | | | |
| 634 | Journal of Osteoporosis | Issa C | 2011 | 0 | http://www.ncbi.nlm.nih.gov//pubmed/21772974 | | | |
| 651 | Hypertension | Kurdi M | 2011 | 0 | http://www.ncbi.nlm.nih.gov.ezproxy.aub.edu.lb/pubmed/21502563 | | | |
| 661 | J Med Liban | Matar N | 2011 | 0 | http://www.ncbi.nlm.nih.gov.ezproxy.aub.edu.lb/pubmed/22746011 | | | |
| 737 | Int J Lab Hematol | Musallam KM | 2011 | 0 | http://www.ncbi.nlm.nih.gov/pubmed/21054811 | | | |
| 1027 | Viruses | Nasr R | 2011 | 1 | http://www.ncbi.nlm.nih.gov/pubmed/21994752 | | | |
| 44 | Pediatric hematology and oncology | Al-Aridi C | 2011 | 0 | http://www.ncbi.nlm.nih.gov/pubmed/?term=Thrombosis+in+children+with+acute+lymphoblastic+leukemia+treated+at+a+tertiary+care+center+in+Lebanon%3A+revisiting+the+role+of+predictive+models | | | |
| 46 | Cancer biology & therapy | Al-Halabi R | 2011 | 0 | http://www.ncbi.nlm.nih.gov/pubmed/?term=Gallotannin+inhibits+NFkB+signaling+and+growth+of+human+colon+cancer+xenografts | | | |
| 100 | Medical hypotheses | Bou Khalil R | 2011 | 0 | http://www.ncbi.nlm.nih.gov/pubmed/?term=Tizanidine+for+alcohol+withdrawal+treatment | | | |
| 140 | J Med Liban | Daher M | 2011 | 0 | http://www.ncbi.nlm.nih.gov/pubmed/?term=Gaps+in+end-of-life+care+Daher+2011 | | | |
| 156 | Frontiers in bioscience | Deeb S J | 2011 | 0 | http://www.ncbi.nlm.nih.gov/pubmed/?term=Sage+components+enhance+cell+death+through+nuclear+factor+kappa-B+signaling | | | |
| 202 | Nutrition | El-Zein O | 2011 | 0 | http://www.ncbi.nlm.nih.gov/pubmed/?term=Pine+bark+extract+inhibits+glucose+transport+in+enterocytes+via+mitogen-activated+kinase+and+phosphoinositol+3-kinase | | | |
| 213 | Plant foods for human nutrition | Fares R | 2011 | 0 | http://www.ncbi.nlm.nih.gov/pubmed/?term=The+antioxidant+and+anti-proliferative+activity+of+the+Lebanese+Olea+europaea+extract | | | |
| 286 | Genetic testing and molecular biomarkers | Hoteit R | 2011 | 0 | http://www.ncbi.nlm.nih.gov/pubmed/?term=Proposed+algorithm+for+the+best+detection+of+different+bcr-abl+gene+fusion+transcripts+in+molecular+diagnostics+laboratories%3A+experience+of+a+major+referral+center | | | |
| 346 | Cancer management and research | Maalouf K | 2011 | 0 | http://www.ncbi.nlm.nih.gov/pubmed/?term=Kefir+induces+cell-cycle+arrest+and+apoptosis+in+HTLV-1-negative+malignant+T-lymphocytes | | | |
| 390 | BMC public health | Nakkash R T | 2011 | 0 | http://www.ncbi.nlm.nih.gov/pubmed/?term=The+rise+in+narghile+(shisha%2C+hookah)+waterpipe+tobacco+smoking%3A+a+qualitative+study+of+perceptions+of+smokers+and+non+smokers | | | |
| 484 | Journal of receptor and signal transduction research | Serhan M F | 2011 | 0 | http://www.ncbi.nlm.nih.gov/pubmed/?term=Insulin+targets+the+Na(%2B)%2FK(%2B)+ATPase+in+enterocytes+via+PI3K%2C+PKC%2C+and+MAPKS | | | |
| 565 | Exp Diabetes Res | Artinian SB | 2011 | 0 | http://www.ncbi.nlm.nih.gov/pubmed/21747829 | | | |
| 574 | Gen Physiol Biophys | Barakat GM | 2011 | 0 | http://www.ncbi.nlm.nih.gov/pubmed/21460410 | | | |
| 617 | Am J of Obstetrics & Gynecology | Ghulmiyyah LM | 2011 | 0 | http://www.ncbi.nlm.nih.gov/pubmed/21531372 | | | |
| 725 | Pharm Biol | Zeinab RA | 2011 | 0 | http://www.ncbi.nlm.nih.gov/pubmed/21777042 | | | |
| 976 | Int J Cancer | Darwiche N | 2011 | 0 | http://www.ncbi.nlm.nih.gov/pubmed/21064094 | | | |
| 1023 | Haematologica | Musallam KM | 2011 | 0 | http://www.ncbi.nlm.nih.gov/pubmed/21282714 | | | |
| 174 | Breast | El Saghir N S | 2011 | 0 | http://www.ncbi.nlm.nih.gov/pubmed/?term=Survey+of+utilization+of+multidisciplinary+management+tumor+boards+in+Arab+countries | | | |
| 314 | The Journal of international medical research | Jambart S | 2011 | 0 | http://www.ncbi.nlm.nih.gov/pubmed/?term=2011+jambart+Prevalence+of+painful+diabetic+peripheral+neuropathy+among+patients+with+diabetes+mellitus+in+the+Middle+East+region | | | |
| 344 | BMC public health | Liu A | 2011 | 0 | http://www.ncbi.nlm.nih.gov/pubmed/?term=Ethnic+differences+in+body+fat+distribution+among+Asian+pre-pubertal+children%3A+a+cross-sectional+multicenter+study | | | |
| 542 | Journal of evaluation in clinical practice | Zeitoun A A | 2011 | 0 | http://www.ncbi.nlm.nih.gov/pubmed/?term=Evaluation+of+anti-emetic+use+in+chemotherapy-induced+nausea+and+vomiting+in+a+third-world+country+(Lebanon) | | | |
| 940 | Osteoporosis international | Sibai AM | 2011 | 0 | http://www.ncbi.nlm.nih.gov/pubmed/21069293 | | | |
| 219 | Clinical breast cancer | Farhat F S | 2011 | 0 | http://www.ncbi.nlm.nih.gov/pubmed/?term=A+phase+II+study+of+lipoplatin+(liposomal+cisplatin)%2Fvinorelbine+combination+in+HER-2%2Fneu-negative+metastatic+breast+cancer | | | |
| 580 | Clinical Therapeutics | Bolliger CT | 2011 | 0 | http://www.ncbi.nlm.nih.gov/pubmed/21635992 | | | |
| 173 | Breast | El Saghir N S | 2011 | 0 | http://www.ncbi.nlm.nih.gov/pubmed/?term=Breast+cancer+management+in+low+resource+countries+(LRCs)%3A+consensus+statement+from+the+Breast+Health+Global+Initiative | | | |
| 93 | Blood | Bazarbachi A | 2011 | 0 | http://www.ncbi.nlm.nih.gov/pubmed/21673346 | | | |
| 716 | Int J Exp Pathol | Trak-Smayra V | 2011 | 0 | http://www.ncbi.nlm.nih.gov/pubmed/22118645 | | | |
| 188 | J Med Liban | El-Herte R | 2011 | 0 | http://www.ncbi.nlm.nih.gov/pubmed/?term=A+patient+with+headache%2C+right+upper+extremity+and+right+hemithorax+hypothermalgesia | | | |
| 48 | J Med Liban | Al-Rifai R | 2011 | 0 | http://www.ncbi.nlm.nih.gov/pubmed/?term=Prevalence+of+peripheral+vascular+calcifications+in+patients+on+chronic+hemodialysis+at+a+tertiary+care+center+in+Beirut%3A+a+pilot+study | | | |
| 57 | J Med Liban | Arabi M | 2011 | 0 | http://www.ncbi.nlm.nih.gov/pubmed/?term=The+status+of+pediatric+cardiology+at+a+tertiary+center+in+Lebanon | | | |
| 377 | Food and nutrition bulletin | Nabhani-Zeidan M | 2011 | 0 | http://www.ncbi.nlm.nih.gov/pubmed/?term=Dietary+intake+and+nutrition-related+knowledge+in+a+sample+of+Lebanese+adolescents+of+contrasting+socioeconomic+status | | | |
| 455 | Eastern Mediterranean health journal | Salameh P | 2011 | 0 | http://www.ncbi.nlm.nih.gov/pubmed/?term=Pattern+of+obesity+and+associated+diabetes+in+Lebanese+adolescents%3A+a+pilot+study+Pub+Year2011 | | | |
| 692 | J Med Liban | Salameh P | 2011 | 0 | http://www.ncbi.nlm.nih.gov.ezproxy.aub.edu.lb/pubmed/22746005 | | | |
| 17 | J Med Liban | Abi Haidar G | 2011 | 0 | http://www.ncbi.nlm.nih.gov/pubmed/?term=Jarrib+Baleha--a+pilot+nutrition+intervention+to+increase+water+intake+and+decrease+soft+drink+consumption+among+school+children+in+Beirut | | | |
| 463 | Clinical Gastroenterology and Hepatology | Samlani-Sebbane Z | 2011 |  | http://www.ncbi.nlm.nih.gov/pubmed/21459071 | | | |
| 503 | Journal of Palliative Medicine | Tazzi I | 2011 |  | http://www.ncbi.nlm.nih.gov/pubmed/21361832 | | | |
| 731 | Annales de Biologie Clinique | Tazi I | 2011 |  | http://www.ncbi.nlm.nih.gov/pubmed/22034668 | | | |
| 851 | Presse médicale | Seddik H | 2011 |  | http://www.ncbi.nlm.nih.gov/pubmed/21367574 | | | |
| 39 | Internal medicine | Akhaddar A | 2011 | 0 | http://www.ncbi.nlm.nih.gov/pubmed/21372479 | | | |
| 41 | Acta orthopaedica et traumatologica turcica | Alami M | 2011 | 0 | http://www.ncbi.nlm.nih.gov/pubmed/21765226 | | | |
| 52 | J Gastrointest Cancer | Arifi S | 2011 | 0 | http://www.ncbi.nlm.nih.gov/pubmed/22045276 | | | |
| 54 | The Pan African Medical Journal | Asabbane A | 2011 | 1 | http://www.ncbi.nlm.nih.gov/pubmed/22121442 | | | |
| 57 | The Pan African Medical Journal | Bahouq H | 2011 | 0 | http://www.ncbi.nlm.nih.gov/pubmed/22121447 | | | |
| 133 | Saudi Journal of Kidney Disease and Transplantation | Bouattar T | 2011 | 0 | http://www.ncbi.nlm.nih.gov/pubmed/21422634 | | | |
| 168 | Bulletin de la Société belge d'ophtalmologie | Chraibi F | 2011 | 0 | http://www.ncbi.nlm.nih.gov/pubmed/22003764 | | | |
| 183 | Annales de Dermatologie et de Vénéréologie | Droussi H | 2011 | 1 | http://www.ncbi.nlm.nih.gov/pubmed/21570568 | | | |
| 241 | The Pan African Medical Journal | Elmazghi A | 2011 | 1 | http://www.ncbi.nlm.nih.gov/pubmed/22355427 | | | |
| 253 | The Pan African Medical Journal | Fadoukhair Z | 2011 | 0 | http://www.ncbi.nlm.nih.gov/pubmed/22121457 | | | |
| 317 | Endoscopy | Ismaili Z | 2011 | 0 | http://www.ncbi.nlm.nih.gov/pubmed/21590608 | | | |
| 333 | Southern medical journal | Kaoutar Z | 2011 | 0 | http://www.ncbi.nlm.nih.gov/pubmed/21297533 | | | |
| 355 | The Pan African Medical Journal | Lakmichi MA | 2011 | 0 | http://www.ncbi.nlm.nih.gov/pubmed/22355428 | | | |
| 395 | BMC Ear Nose Throat Disord | Mesmoudi M | 2011 | 0 | http://www.ncbi.nlm.nih.gov/pubmed/21658269 | | | |
| 396 | J Med Case Rep | Mesmoudi M | 2011 | 0 | http://www.ncbi.nlm.nih.gov/pubmed/21929776 | | | |
| 403 | J Gastrointest Cancer | Mokrim M | 2011 | 0 | http://www.ncbi.nlm.nih.gov/pubmed/21894458 | | | |
| 415 | Rev Stomatol Chir Maxillofac | Nadour K | 2011 | 1 | http://www.ncbi.nlm.nih.gov/pubmed/21429542 | | | |
| 416 | Annales de Biologie Clinique | Nafil H | 2011 | 1 | http://www.ncbi.nlm.nih.gov/pubmed/22123576 | | | |
| 417 | Annales de Biologie Clinique | Nafil H | 2011 | 1 | http://www.ncbi.nlm.nih.gov/pubmed/22123569 | | | |
| 418 | Rev Stomatol Chir Maxillofac | Nafil H | 2011 | 1 | http://www.ncbi.nlm.nih.gov/pubmed/22071151 | | | |
| 429 | Eur Ann Otorhinolaryngol Head Neck Dis | Nouri H | 2011 | 0 | http://www.ncbi.nlm.nih.gov/pubmed/21602115 | | | |
| 444 | The Pan African Medical Journal | Rais G | 2011 | 1 | http://www.ncbi.nlm.nih.gov/pubmed/22121456 | | | |
| 466 | World journal of Surgical Oncology | Sbitti Y | 2011 | 0 | http://www.ncbi.nlm.nih.gov/pubmed/21955806 | | | |
| 467 | World journal of Surgical Oncology | Sbitti Y | 2011 | 0 | http://www.ncbi.nlm.nih.gov/pubmed/21682901 | | | |
| 470 | The Pan African Medical Journal | Serghini I | 2011 | 1 | http://www.ncbi.nlm.nih.gov/pubmed/22355422 | | | |
| 473 | World journal of Surgical Oncology | Smahi M | 2011 | 0 | http://www.ncbi.nlm.nih.gov/pubmed/21352593 | | | |
| 480 | Journal of Cardiovascular Disease Research | Souirti Z | 2011 | 0 | http://www.ncbi.nlm.nih.gov/pubmed/21814418 | | | |
| 490 | Annales de Biologie Clinique | Tali A | 2011 | 0 | http://www.ncbi.nlm.nih.gov/pubmed/21464001 | | | |
| 497 | North American Journal of Medical Sciences | Tazzi el M | 2011 | 0 | http://www.ncbi.nlm.nih.gov/pubmed/22540062 | | | |
| 500 | World journal of Surgical Oncology | Tazzi EM | 2011 | 0 | http://www.ncbi.nlm.nih.gov/pubmed/21752265 | | | |
| 506 | African Journal of Health Sciences | Tazzi I | 2011 | 0 | http://www.ncbi.nlm.nih.gov/pubmed/21857864 | | | |
| 519 | Internal medicine | Traibi A | 2011 | 0 | http://www.ncbi.nlm.nih.gov/pubmed/21628948 | | | |
| 523 | European journal of gynaecological oncology | Zakkouri FA | 2011 | 0 | http://www.ncbi.nlm.nih.gov/pubmed/21614920 | | | |
| 695 | The Pan African Medical Journal | BoulaÃ¢mane L | 2011 | 1 | http://www.ncbi.nlm.nih.gov/pubmed/22145060 | | | |
| 700 | Chirurgie de la Main | Chbani L | 2011 | 1 | http://www.ncbi.nlm.nih.gov/pubmed/21334949 | | | |
| 722 | Revue de Pneumologie Clinique | Tazi EM | 2011 | 0 | http://www.ncbi.nlm.nih.gov/pubmed/21665080 | | | |
| 730 | Indian Journal of Dermatology | Tazi I | 2011 | 0 | http://www.ncbi.nlm.nih.gov/pubmed/22121279 | | | |
| 733 | African Health Sciences | Tazi I | 2011 | 0 | http://www.ncbi.nlm.nih.gov/pubmed/21857864 | | | |
| 735 | Leukemia Research | Tazi I | 2011 | 0 | http://www.ncbi.nlm.nih.gov/pubmed/21247633 | | | |
| 832 | The Pan African Medical Journal | Ouadnouni Y | 2011 | 1 | http://www.ncbi.nlm.nih.gov/pubmed/22355418 | | | |
| 840 | Revue de pneumologie clinique | Zidane A | 2011 | 1 | http://www.ncbi.nlm.nih.gov/pubmed/22137283 | | | |
| 844 | Annales de Biologie Clinique | El Boukhrissi F | 2011 | 1 | http://www.ncbi.nlm.nih.gov/pubmed/22008141 | | | |
| 845 | Annales de Biologie Clinique | Benchekroun L | 2011 | 1 | http://www.ncbi.nlm.nih.gov/pubmed/22008139 | | | |
| 846 | Annales de Dermatologie et de Vénéréologie | El Bakkal A | 2011 | 1 | http://www.ncbi.nlm.nih.gov/pubmed/21978503 | | | |
| 847 | Annales de Chirurgie Plastique Esthétique | Moumine M | 2011 | 1 | http://www.ncbi.nlm.nih.gov/pubmed/21689588 | | | |
| 850 | Revue de stomatologie et de chirurgie maxillo-faciale | Belghiti H | 2011 | 1 | http://www.ncbi.nlm.nih.gov/pubmed/21388648 | | | |
| 852 | Chirurgie de la main | Amar MF | 2011 | 1 | http://www.ncbi.nlm.nih.gov/pubmed/21334956 | | | |
| 853 | Revue de stomatologie et de chirurgie maxillo-faciale | Moumine M | 2011 | 1 | http://www.ncbi.nlm.nih.gov/pubmed/21334704 | | | |
| 855 | Annales de Cardiologie et d'Angéiologie | Fettouhi H | 2011 | 1 | http://www.ncbi.nlm.nih.gov/pubmed/21276952 | | | |
| 856 | Annales Françaises d'Anesthésie et de Réanimation | Asfalou I | 2011 | 1 | http://www.ncbi.nlm.nih.gov/pubmed/21232905 | | | |
| 858 | Progrès en urologie | Tazi H | 2011 | 1 | http://www.ncbi.nlm.nih.gov/pubmed/21193150 | | | |
| 859 | The Pan African Medical Journal | El M'rabet FZ | 2011 | 1 | http://www.ncbi.nlm.nih.gov/pubmed/22384290 | | | |
| 860 | Gynécologie, obstétrique & fertilité | Guedira I | 2011 | 1 | http://www.ncbi.nlm.nih.gov/pubmed/21183391 | | | |
| 865 | Revue neurologique | Messouak M | 2011 | 1 | http://www.ncbi.nlm.nih.gov/pubmed/21051064 | | | |
| 870 | Revue neurologique | Raissouni L | 2011 | 1 | http://www.ncbi.nlm.nih.gov/pubmed/20561657 | | | |
| 6 | The Pan African Medical Journal | Abahssain H | 2011 | 1 | http://www.ncbi.nlm.nih.gov/pubmed/22121427 | | | |
| 7 | Eastern Mediterranean Health Journal | Abbass F | 2011 | 1 | http://www.ncbi.nlm.nih.gov/pubmed/22355946 | | | |
| 34 | BMC Research Notes | Akasbi Y | 2011 | 0 | http://www.ncbi.nlm.nih.gov/pubmed/22088140 | | | |
| 49 | Annales de Biologie Clinique | Amine K | 2011 | 0 | http://www.ncbi.nlm.nih.gov/pubmed/22123566 | | | |
| 141 | Bulletin Du Cancer | Boulaamane L | 2011 | 1 | http://www.ncbi.nlm.nih.gov/pubmed/21896399 | | | |
| 145 | BMC Research Notes | Bourhafour M | 2011 | 0 | http://www.ncbi.nlm.nih.gov/pubmed/21714875 | | | |
| 152 | The Pan African Medical Journal | Brahmi SA | 2011 | 0 | http://www.ncbi.nlm.nih.gov/pubmed/22187618 | | | |
| 165 | Archives of Medical Research | Cherkaoui Dekkaki I | 2011 | 0 | http://www.ncbi.nlm.nih.gov/pubmed/22227044 | | | |
| 178 | Arab Journal of Gastroenterology | Diffaa A | 2011 | 0 | http://www.ncbi.nlm.nih.gov/pubmed/21429457 | | | |
| 203 | Age and Ageing | El fakir S | 2011 | 0 | http://www.ncbi.nlm.nih.gov/pubmed/21791445 | | | |
| 204 | American Journal of health promotion | El fakir S | 2011 | 0 | http://www.ncbi.nlm.nih.gov/pubmed/21721964 | | | |
| 213 | Revue de pneumologie clinique | EL Idrissi-Raja L | 2011 | 1 | http://www.ncbi.nlm.nih.gov/pubmed/22017948 | | | |
| 230 | Public health nutrition | El Rhazi K | 2011 | 0 | http://www.ncbi.nlm.nih.gov/pubmed/20602865 | | | |
| 240 | Sante (Montrouge, France) | Elmachtani Idrissi S | 2011 | 1 | http://www.ncbi.nlm.nih.gov/pubmed/21700555 | | | |
| 242 | BMC dermatology | Errihani H | 2011 | 0 | http://www.ncbi.nlm.nih.gov/pubmed/22078023 | | | |
| 251 | International Journal of Biological Markers | Ezzikouri S | 2011 | 0 | http://www.ncbi.nlm.nih.gov/pubmed/22180176 | | | |
| 263 | The Pan African Medical Journal | Ghanem S | 2011 | 0 | http://www.ncbi.nlm.nih.gov/pubmed/22187603 | | | |
| 266 | The Pan African Medical Journal | Glaoui M | 2011 | 1 | http://www.ncbi.nlm.nih.gov/pubmed/22121443 | | | |
| 275 | Bulletin de la Société de pathologie exotique | Hali F | 2011 | 1 | http://www.ncbi.nlm.nih.gov/pubmed/21191827 | | | |
| 304 | Cancer Radiothérapie | Houjami M | 2011 | 1 | http://www.ncbi.nlm.nih.gov/pubmed/21256791 | | | |
| 321 | Actas urologicas españolas | Janane A | 2011 | 1 | http://www.ncbi.nlm.nih.gov/pubmed/21664008 | | | |
| 342 | Infectious agents and cancer | Laantri N | 2011 | 0 | http://www.ncbi.nlm.nih.gov/pubmed/21352537 | | | |
| 357 | Annales de Dermatologie et de Vénéréologie | Lamchahab FE | 2011 | 1 | http://www.ncbi.nlm.nih.gov/pubmed/22078032 | | | |
| 358 | Annals of Physical and Rehabilitation Medicine | Lamchahab FZ | 2011 | 0 | http://www.ncbi.nlm.nih.gov/pubmed/21840783 | | | |
| 388 | Eastern Mediterranean Health Journal | Maoujoud O | 2011 | 1 | http://www.ncbi.nlm.nih.gov/pubmed/21735803 | | | |
| 437 | International Journal of Endocrinology | Ould Mohamedou M M | 2011 | 0 | http://www.ncbi.nlm.nih.gov/pubmed/22114593 | | | |
| 469 | BMC Women's Health | Sbitti Y | 2011 | 1 | http://www.ncbi.nlm.nih.gov/pubmed/21668971 | | | |
| 485 | Eastern Mediterranean Health Journal | Tachfouti N | 2011 | 0 | http://www.ncbi.nlm.nih.gov/pubmed/22259887 | | | |
| 516 | Acta Neurologica Scandinavica | They-They TP | 2011 | 0 | http://www.ncbi.nlm.nih.gov/pubmed/20637011 | | | |
| 520 | Journal of Neurological Sciences | Tran J | 2011 | 0 | http://www.ncbi.nlm.nih.gov/pubmed/21669445 | | | |
| 530 | Archives of Gynecology and Obstetrics | Znati K | 2011 | 0 | http://www.ncbi.nlm.nih.gov/pubmed/20454905 | | | |
| 531 | Pathol Biol (Paris) | Benmoussa A | 2011 | 0 | http://www.ncbi.nlm.nih.gov/pubmed/19939582 | | | |
| 848 | Presse médicale | Hali F | 2011 | 1 | http://www.ncbi.nlm.nih.gov/pubmed/21684107 | | | |
| 854 | Gynécologie, obstétrique & fertilité | Bensouda Y | 2011 | 1 | http://www.ncbi.nlm.nih.gov/pubmed/21324724 | | | |
| 857 | Journal de gynécologie, obstétrique et biologie de la reproduction | Tanz R | 2011 | 1 | http://www.ncbi.nlm.nih.gov/pubmed/21227599 | | | |
| 244 | International journal of gynecological cancer : official journal of the International Gynecological | Errihani H | 2011 | 0 | http://www.ncbi.nlm.nih.gov/pubmed/21270619 | | | |
| 534 | Annals of Human Biology | Laarabi FZ | 2011 | 0 | http://www.ncbi.nlm.nih.gov/pubmed/20939750 | | | |
| 115 | Eur Ann Otorhinolaryngol Head Neck Dis | Bensouda Y | 2011 | 0 | http://www.ncbi.nlm.nih.gov/pubmed/21177151 | | | |
| 219 | La Revue de médecine interne | El Mesbahi O | 2011 | 1 | http://www.ncbi.nlm.nih.gov/pubmed/21333410 | | | |
| 278 | Indian J Hum Genet | Hamzi K | 2011 | 0 | http://www.ncbi.nlm.nih.gov/pubmed/22345995 | | | |
| 310 | World journal of Surgical Oncology | Ismaili N | 2011 | 0 | http://www.ncbi.nlm.nih.gov/pubmed/22115124 | | | |
| 311 | Orphanet journal of rare diseases | Ismaili N | 2011 | 0 | http://www.ncbi.nlm.nih.gov/pubmed/22078012 | | | |
[truncated: 151,304 more chars]
